# Supplementary material for: Scalable total synthesis of (+)-aniduquinolone A and its acid-catalyzed rearrangement to aflaquinolones
Source: Commun Chem. 2022 Mar 17;5:35. doi: 10.1038/s42004-022-00655-x (PMC9814574; doi:10.1038/s42004-022-00655-x)
Supplement: Supplementary file 1 — Description of Additional Supplementary Files [file 42004_2022_655_MOESM1_ESM.pdf]

Description of Additional Supplementary Files

File Name: Supplementary Data 1

Description: CCDC 2122872 (Compound 1)

File Name: Supplementary Data 2

Description: CCDC 2122875 (Compound 14)

File Name: Supplementary Data 3

Description: CCDC 2122876 (Compound 29)
