# Supplementary material for: Scalable total synthesis of (+)-aniduquinolone A and its acid-catalyzed rearrangement to aflaquinolones
Source: Commun Chem. 2022 Mar 17;5:35. doi: 10.1038/s42004-022-00655-x (PMC9814574; doi:10.1038/s42004-022-00655-x)
Supplement: Supplementary file 5 — Supplementary Information [file 42004_2022_655_MOESM5_ESM.pdf]

Supplementary Information

Scalable total synthesis of (+)-aniduquinolone A and its acid-catalyzed rearrangement to aflaquinolones

Feng-Wei Guo,[1,2,6] Xiao-Feng Mou,[1,3,6] Yong Qu,[1,2] Mei-Yan Wei,[1] Guang-Ying Chen,[4] Chang-Yun Wang,[1,2] Yu-Cheng Gu,[5] and Chang-Lun Shao*[1,2]

[1] Key Laboratory of Marine Drugs, The Ministry of Education of China, School of Medicine and Pharmacy, Ocean University of China, Qingdao 266003, China.

[2] Laboratory for Marine Drugs and Bioproducts, Pilot National Laboratory for Marine Science and Technology (Qingdao), Qingdao 266200, China.

[3] School of Pharmacy, Yantai University, Yantai, 264005, China.

[4] College of Chemistry and Chemical Engineering, Hainan Normal University, Haikou 571158, China.

[5] Syngenta Jealott's Hill International Research Centre, Bracknell, Berkshire, RG42 6EY, UK.

[6] These authors contributed equally: Feng-Wei Guo, Xiao-Feng Mou.

E-mail: shaochanglun@163.com

Table of Contents

Supplementary Methods 3

Supplementary Notes 4

1. Experimental Procedures and Spectroscopic Data 4

Supplementary Tables 21

1. **Comparison of NMR Spectra of Isolated and Synthetic Compounds** 21

Supplementary Figures 25

1. **X-ray Crystallographic Data** 25
2. **Experimental ECD Spectra** 29
3. **Comparison of the NMR Spectral Data of Compound 1 and Its Stereoisomers** .. 30
4. **NMR Spectra of the Synthesized Compounds in This Article** 32

Supplementary References 99

Supplementary Methods

General Information

All reactions were performed under nitrogen or argon in vacuum-dried glassware using dry solvents and standard syringe techniques. Reagents were purchased at the highest commercial quality (>95%) and used without further purification, unless otherwise stated. Anhydrous tetrahydrofuran (THF), toluene (PhMe) were distilled from sodium-benzophenone, dichloromethane (CH_2Cl_2) and dimethylformamide (DMF) were distilled from calcium hydride. Column chromatography was carried out by using silica gel (200−300 mesh). Yields refer to chromatographically, unless otherwise specified. Optical rotations were measured on a JASCO P-1020 digital polarimeter (JASCO Ltd., Tokyo, Japan). IR spectrawere recorded on a Nicolet-Nexus-470 spectrometer (Perkin Elmer Ltd., Boston, MA, USA) using KBr pellets. NMR spectra were recorded on a JEOL JEM-ECP NMR spectrometer (JEOL Ltd., Tokyo, Japan; 500MHz for ^{1}H and 125MHz for ^{13}C), using TMS as internal standard. The ESI-MS spectra were obtained from a Micromass Q-TOF spectrometer (Waters Ltd., Boston, MA, USA). Semi-preparative HPLC was performed on a Hitachi L-2000 system (Hitachi Ltd., Tokyo, Japan) using a C18 column (Kromasil (Eka Ltd., Bohus, Sweden) 250 × 10 mm, 5 μm, 2.0 mL/min). Diastereomeric mixtures were resolved on a Chiralpak IC column (5 μm, 4.6 mm × 250 mm, hexane−2-propanol eluent, 0.5 mL/min). UPLC-MS was performed on Waters UPLC® system (Waters Ltd., Massachusetts, America) using a C_{18} column [(Waters Ltd., Massachusetts, America) ACQUITY UPLC® BEH C18, 2.1 × 50 mm, 1.7 μm; 0.5 mL/min] and ACQUITY QDa ESIMS scan from 150 to 1000 Da. Silica gel (Qingdao Haiyang Chemical Group Co., Qingdao, China; 200–300 mesh), octadecylsilyl silica gel (YMC Co., Ltd., Tokyo, Japan; 45–60 μm), and Sephadex LH-20 (GE Ltd., Hartford, CT, USA) were used for column chromatography (CC). Precoated silica gel plates (Yantai Zhifu Chemical Group Co., Yantai, China; G60, F-254) were used for thin layer chromatography.

Abbreviations used: Et_3N = triethylamine, EtOAc = ethyl acetate, CH_2Cl_2 = dichloromethane, THF = tetrahydrofuran, MeOH = methanol, Na_2SO_4 = sodium sulfate, HCl = hydrochloric acid, NH_4Cl = ammonium chloride, $NaHCO_3$ = sodium hydrogencarbonate (sodium bicarbonate), $NaBH_4$ = sodium borohydride, *m*CPBA = *m*-chloroperoxybenzoic acid

Supplementary Notes

1. Experimental Procedures and Spectroscopic Data

Preparation of Compound 18:

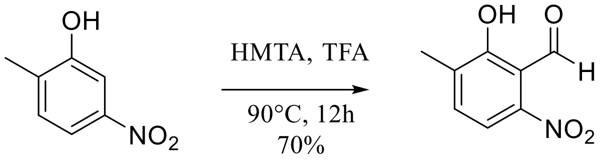

2-methyl-5-nitrophenol (**15**) **18**

A solution of 2-methyl-5-nitrophenol (60.0 g, 392 mmol) in F_3CCO_2H (600 mL) was treated with HMTA (65.9 g, 470 mmol) and the mixture was heated at 90 ºC for 12 h. The reaction was poured over ice-water (900 mL). The resultant mixture was stirred for 30 min and then extracted with EtOAc (3 × 600 mL). The organic layers were washed with brine (600 mL), dried under Na_2SO_4 and concentrated in vacuo. Chromatography of the oily residue afforded **18** (49.7 g, 70%), as a yellow solid.

Compound **18**: **R_f** 0.5 (4:1 petroleum ether: ethyl acetate); **^{1}H NMR** (400 MHz, acetone-d_6) δ 12.39 (s, 1H), 10.35 (s, 1H), 7.69 (d, J = 8.1 Hz, 1H), 7.60 (d, J = 8.1 Hz, 1H), 2.33 (s, 3H). **^{13}C NMR** (100 MHz, acetone-d_6) δ 196.1, 161.9, 150.5, 137.0, 135.3, 116. 7, 112.5, 15.8.

Preparation of Compound 19:

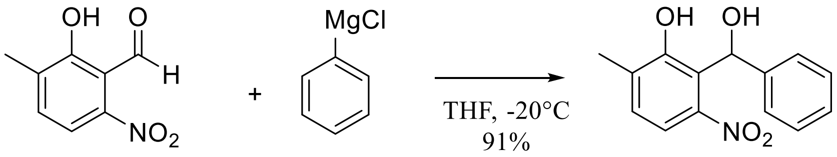

18 **19**

To a stirred solution of compound **18** (47.5 g, 262 mmol) in THF (500 mL) at -20°C was added dropwise of a 2 mol•L^{-1} phenylmagnesium chloride (262 mL, 524 mmol) solution in dry THF, and stirred at -20°C for 20 min. The reaction was quenched with a saturated aqueous solution of NH_4Cl (500 mL), and the aqueous layer was extracted with EtOAc (3×500 mL). The combined organic layers were washed successively with water (500 mL) and brine (500 mL), dried over anhydrous Na_2SO_4 and evaporated under reduced pressure. The residue was purified by flash chromatography on silica gel (eluent: EtOAc/PE, 1/5 to 1/4) to give compound **19** (62.0 g, yield: 91%) as a white solid.

Compound **19**: **R_f** 0.3 (3:1 petroleum ether: ethyl acetate); **^{1}H NMR** (400 MHz, $CDCl_3$) δ 9.22 (s, 1H), 7.40 (d, J = 8.3 Hz, 1H), 7.38 – 7.28 (m, 5H), 7.20 (d, J = 8.3 Hz, 1H), 6.63 (s, 1H), 3.43 (s, 1H), 2.30 (s, 3H). **^{13}C NMR** (100 MHz, $CDCl_3$) δ 155.7, 147.3, 140.1, 133.7, 130.3, 128.9, 128.6, 126.8, 119.9, 116.1, 72.9, 16.7.

Preparation of Compound 14:

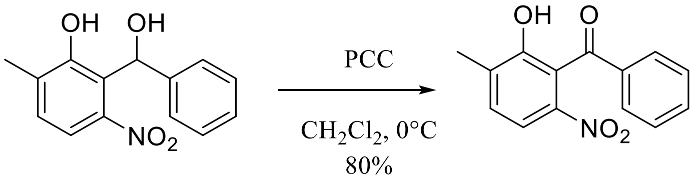

19 **14**

To a stirred solution of compound **19** (40.0 g, 154 mmol) in dry CH_2Cl_2 (500 mL), PCC (40.0 g, 185 mmol) was added at 0 °C[1]. The mixture was allowed to react at 0°C for 4 h and filtered with diatomite to remove insoluble solid. The volatiles were then removed under reduced pressure. The residue was dissolved in CH_2Cl_2 (200 mL), washed sequentially with water (200 mL) and brine (200 mL), dried over anhydrous Na_2SO_4, filtered and concentrated under reduced pressure. The residue was purified by flash chromatography on silica gel (eluent: EtOAc/PE, 1/5 to 1/3) to afford compound **14** (31.8 g, yield: 80%) as a white solid.

Compound **14**: **Rf** 0.4 (3:1 petroleum ether : ethyl acetate); **IR** (KBr) vmax (cm^{-1}) 3407, 1669, 1596, 1519, 1466, 1351, 1320, 1284, 1245, 1211, 1174, 953; **^{1}H NMR** (400 MHz, $CDCl_3$) δ 7.73-7.62 (m, 2H), 7.58-7.55 (m, 2H), 7.47-7.36 (m, 3H), 2.39 (s, 3H). **^{13}C NMR** (100 MHz, $CDCl_3$) δ 196.2, 156.3, 148.1, 137.7, 134.6, 133.9, 133.1, 129.0, 128.2, 116.4, 115.8, 16.7; **HRMS (ESI)**: m/z calcd for $C_{14}H_{12}O_4N$ $[M+H]^+$: 258.0761; found: 258.0762.

Preparation of Compound 20:

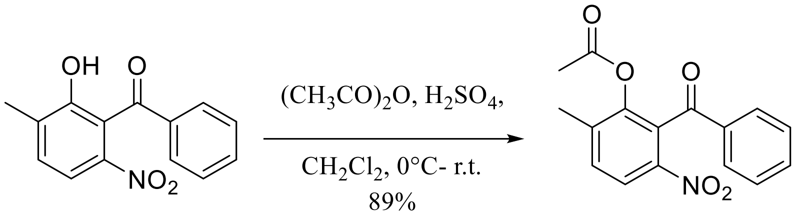

14 **20**

To a stirred solution of compound **14** (28.0 g, 109 mmol) in CH_2Cl_2 (200 mL) and acetic anhydride (100 mL) at 0℃, was added concentrated H_2SO_4 solution (3.35 mL). Then the reaction was stirred at room temperature for 12 h. After completion, the organic solvent was removed in vacuo and the resulting mixture was diluted with H_2O (300 mL). The aqueous layer was extracted with EtOAc (3×300 mL). The combined organic layers were washed with brine (300 mL), dried over anhydrous Na_2SO_4, filtered and concentrated under reduced pressure. The residue was purified by flash chromatography on silica gel (eluent: EtOAc/PE, 1/10 to 1/8) to give compound **20** (29.0 g, yield: 89%) as a white solid.

Compound **20**: **R_f** 0.2 (5:1 petroleum ether: ethyl acetate); **IR** (KBr) vmax (cm^{-1}) 1778, 1682, 1594, 1527, 1452, 1349, 1316, 1278, 1249, 1203, 1181, 1012, 959, 880, 714;

^{1}H NMR (400 MHz, $CDCl_3$) δ 8.09 (d, J = 8.5 Hz, 1H), 7.75 (d, J = 7.2 Hz, 2H), 7.57 (t, J = 7.4 Hz, 1H), 7.50 (d, J = 8.5 Hz, 1H), 7.43 (t, J = 7.8 Hz, 2H), 2.27 (s, 3H), 1.96 (s, 3H). **^{13}C NMR** (100 MHz, $CDCl_3$) δ 190.9, 167.7, 147.1, 145.0, 140.3, 135.8, 134.0, 132.0, 129.8, 129.0, 128.8, 122.1, 20.0, 17.0; **HRMS (ESI)**: m/z calcd for $C_{16}H_{14}O_5N$ $[M+H]^+$: 300.0866; found: 300.0861.

Preparation of Compound 21:

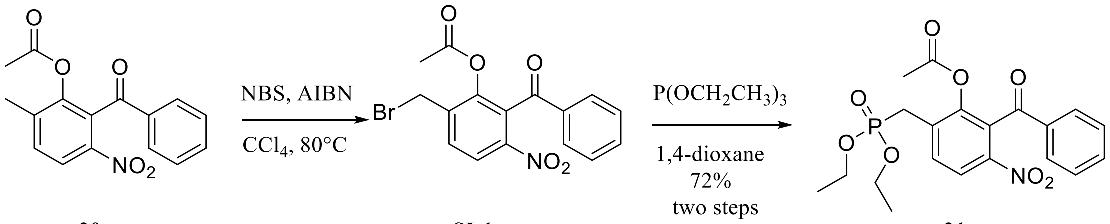

20 **SI-1** **21**

To a solution of compound **20** (14.5 g, 48.5 mmol) in CCl_4 (250 mL) was added N-bromosuccinimide (8.62 g, 48.5 mmol) and azobisisobutyronitrile (0.40 g, 2.42 mmol). The mixture was allowed to react at 80°C for 24 h. The solvent was evaporated under reduced pressure to yield product **SI-1**and the crude material was used in the next reaction without purification.

To a stirred solution of crude **SI-1** in 1,4-dioxane (200 mL) was added triethyl phosphite (32.2 g, 194 mmol), and the mixture was heated and stirred at 120 °C for 8 h. After completion, the organic solvent was removed in vacuo. The residue was purified by flash chromatography on silica gel (eluent: EtOAc/PE, 3/1 to 5/1) to give compound **21** (15.2 g, yield: 72%) as a yellow oil.

Compound **21**: $\mathbf{R_f}$ 0.2 (1:3 petroleum ether : ethyl acetate); **IR** (KBr) vmax (cm^{-1}) 2985, 1779, 1682, 1529, 1452, 1367, 1347, 1313, 1278, 1252, 1182, 1165, 1050, 1025, 962, 857; **^{1}H NMR** (400 MHz, $CDCl_3$) δ 8.16 (d, J = 8.6 Hz, 1H), 7.77 (overlapped, 3H), 7.59 (t, J = 7.4 Hz, 1H), 7.44 (t, J = 7.8 Hz, 2H), 4.08 (overlapped, 4H), 3.14 (d, J = 22.2 Hz, 2H), 1.99 (s, 3H), 1.28 (t, J = 7.0 Hz, 6H). **^{13}C NMR** (100 MHz, $CDCl_3$) δ 190.4, 168.0, 147.2, 146.0, 135.8, 134.5, 134.2, 132.5, 130.3, 129.0, 128.9, 122.2, 62.8, 29.4, 28.1, 20.2, 16.5; **HRMS (ESI)**: m/z calcd for $C_{20}H_{23}O_8NP$ $[M+H]^+$: 436.1156; found: 436.1148.

Preparation of Compound 12:

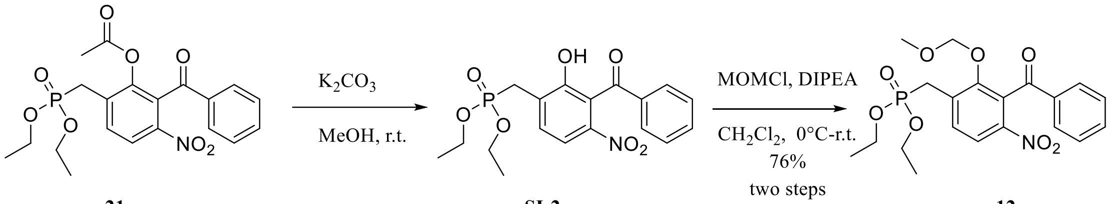

To a stirred solution of compound **21** (9.00 g, 20.7 mmol) in MeOH (150 mL) was added K_2CO_3 (3.43 g, 24.8 mmol) at room temperature and stirred for 1 h. After completion, the organic solvent was removed in vacuo and the resulting mixture was diluted with H_2O (150 mL), the aqueous layer extracted with EtOAc (3 ×150 mL). The combined organic layers were washed with brine (150 mL), dried over anhydrous Na_2SO_4, filtered, and concentrated under reduced pressure to yield product **SI-2**. The product was used without further purification.

To a stirred solution of compound **SI-2** and DIPEA (5.34 g, 41.3 mmol) in dry CH_2Cl_2 (150 mL) was added MOMCl (2.50 g, 31.0 mmol) at 0℃. The reaction was removed from the ice bath and stirred at room temperature for 8 h. The reaction was quenched with saturated aqueous NH_4Cl (150 mL), and then the aqueous layer was extracted with CH_2Cl_2 (3 × 150 mL). The combined organic layers were washed with brine (150 mL), dried over anhydrous Na_2SO_4, filtered and concentrated under reduced pressure. The residue was purified by flash chromatography on silica gel (eluent: EtOAc/PE, 1/1 to 3/1) to give compound **12** (6.87 g, yield: 76%) as a yellow oil.

Compound **12**: $\mathbf{R_f}$ 0.2 (1:3 petroleum ether : ethyl acetate); **IR** (KBr) vmax (cm^{-1}) 3422, 1650, 1051, 1028, 1005, 825, 764, 625, 424; **^{1}H NMR** (400 MHz, $CDCl_3$) δ 8.04 (d, J = 8.6 Hz, 1H), 7.75 (dt, J = 3.1, 2.0 Hz, 3H), 7.57 (dd, J = 10.6, 4.3 Hz, 1H), 7.43 (t, J = 7.7 Hz, 2H), 4.93 (s, 2H), 4.24 – 3.97 (m, 4H), 3.42 (d, J = 22.5 Hz, 2H), 3.35 (s, 3H), 1.28 (t, J = 7.1 Hz, 6H). **^{13}C NMR** (100 MHz, $CDCl_3$) δ 192.1, 154.1, 154.0, 145.7, 136.5, 135.3, 135.2, 133.9, 132.5, 132.4, 131.4, 128.9, 128.9, 120.5, 102.0, 62.6, 62.6, 57.8, 28.6, 27.2, 16.5; **HRMS (ESI)**: m/z calcd for $C_{20}H_{25}O_8NP$ $[M+H]^+$: 438.1312; found: 438.1307.

Preparation of Compound SI-4:

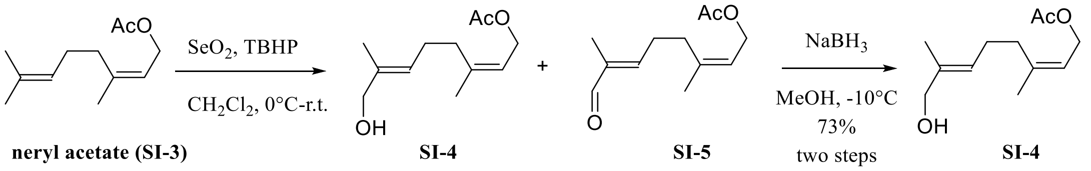

To a solution of selenium dioxide (4.52 g, 40.8 mmol) in CH_2Cl_2 (300 mL) was added tert-butyl hydroperoxide (5 M in decane, 81.5 mL, 408 mmol) and neryl acetate (**SI-3**) (40.0 g, 204 mmol). After the mixture was stirred for 24 h at room temperature, water (600 mL) and CH_2Cl_2 (300 mL) were added, and the reaction was transferred to a separatory funnel. The layers were separated, and the organic layer was washed successively with water (2 × 300 mL), saturated aqueous $NaHCO_3$ (300 mL), and brine (300 mL). The organic layer was dried over Na_2SO_4, filtered and concentrated under reduced pressure to yield product **SI-4** and **SI-5**. The product was used without further purification.

To a stirred solution of crude **SI-4** and **SI-5** in MeOH (300 mL) was added $NaBH_4$ (6.86 g, 186 mmol) at -15℃and stirred for 1 h. After completion, the organic solvent was removed in vacuo and the resulting mixture was diluted with saturated aqueous NH_4Cl (300 mL). The aqueous layer was extracted with EtOAc (3 ×300 mL) and the combined organic layers were washed with brine (300 mL), dried over anhydrous Na_2SO_4, filtered and concentrated under reduced pressure. The residue was purified by flash chromatography on silica gel (eluent: EtOAc/PE, 1/6 to 1/3) to give compound **SI-4** (31.6 g, yield: 73%) as a colorless oil. Spectral data matched the reported literature values.[1]
Compound **SI-4**: $\mathbf{R_f}$ 0.2 (4:1 petroleum ether: ethyl acetate); **^{1}H NMR** (400 MHz, $CDCl_3$) δ 5.34 (overlapped, 2H), 4.52 (d, J = 7.2 Hz, 2H), 3.94 (s, 2H), 2.11 (overlapped, 4H), 2.01 (s, 3H), 1.73 (s, 3H), 1.62 (s, 3H). **^{13}C NMR** (100 MHz, $CDCl_3$) δ 171.3, 142.0, 135.7, 124.7, 119.5, 68.6, 61.2, 31.8, 26.0, 23.4, 21.1, 13.7.

Preparation of Compound SI-6:

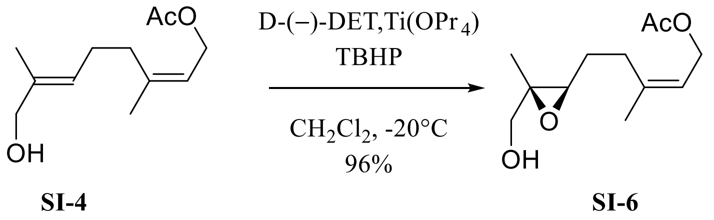

To a suspension of activated molecular sieves (4 Å) in CH_2Cl_2 (450 mL) at –20 °C was added titanium tetraisopropoxide (20.9 g, 73.5 mmol), D-(-)-diethyl tartrate (16.4 g, 79.6 mmol) and and TBHP (49.0 mL, 245 mmol, 5M in decane). After 20 min, a solution of **SI-4** (26.0 g, 123 mmol) in dry CH_2Cl_2 (100 mL) was added dropwise over 0.5 h. Upon completion of the reaction, as judged by TLC analysis, the mixture was quenched with 300 mL of distilled water and allowed to warm to rt. After 30 min of vigorous stirring, the solution was filtered through Celite. The organic phase was separated and the aqueous phase was extracted with CH_2Cl_2 (3 × 300 mL). The combined organic layers were washed with brine (300 mL), dried over anhydrous Na_2SO_4, filtered and concentrated under reduced pressure, the residue was purified by flash chromatography on silica gel (eluent: EtOAc/PE, 1/3 to 1/2) to afford compound **SI-6** (26.8 g, yield: 96%) as a colorless oil. Spectral data matched the reported literature values.[1]

Compound **SI-6**: **R$_f$** 0.3 (3:1 petroleum ether: ethyl acetate); **^{1}H NMR** (400 MHz, CDCl$_3$) δ 5.34 (t, J = 7.2 Hz, 1H), 4.52 (d, J = 7.3 Hz, 2H), 3.59 (d, J = 12.2 Hz, 1H), 3.50 (d, J = 11.8 Hz, 1H), 2.95 (t, J = 6.3 Hz, 1H), 2.50 (s, 1H), 2.20 (t, J = 7.5 Hz, 2H), 1.98 (s, 3H), 1.72 (s, 3H), 1.62 (m, 2H), 1.23 (s, 3H). **^{13}C NMR** (100 MHz, CDCl$_3$) δ 171.1, 141.3, 120.0, 65.5, 61.2, 60.9, 59.6, 28.7, 26.7, 23.3, 21.0, 14.2.

Preparation of Compound SI-7:

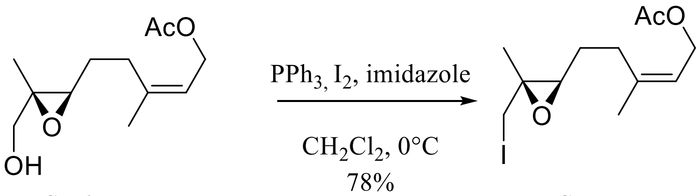

SI-6 **SI-7**

To a stirred solution of **SI-6** (26.0g, 114 mmol) in CH_2Cl_2 (400 mL) at 0°C was added imidazole (23.3 g, 341 mmol). When the imidazole was totally dissolved, PPh_3 (47.8 g, 182 mmol) was added in one portion. When the solution became homogeneous, I_2 beads (46.3 g, 182 mmol) were added and the solution was stirred vigorously at 0 °C for 30 min. Upon completion of the reaction, as judged by TLC analysis, the reaction mixture was concentrated under reduced pressure. The residue was dissolved in Et_2O (300 mL) and washed with saturated aqueous $Na_2S_2O_3$. The Et_2O solution was separated, dried over Na_2SO_4 and concentrated under reduced pressure. The residue was purified by flash chromatography on silica gel (eluent: EtOAc/PE, 1/20 to 1/15) to afford compound **SI-7** (30.0 g, yield: 78%) as a yellow oil. Spectral data matched the reported literature values.[1]

Compound **SI-7**: **R$_f$** 0.5 (15:1 petroleum ether: ethyl acetate); **^{1}H NMR** (400 MHz, CDCl$_3$) δ 5.40 (t, J = 7.2 Hz, 1H), 4.57 (d, J = 7.3 Hz, 2H), 3.21 (d, J = 10Hz, 1H), 3.10 (d, J = 10Hz, 1H), 2.88 (t, J = 6.3 Hz, 1H), 2.26 (t, J = 8.2 Hz, 2H), 2.03 (s, 3H), 1.78 (s, 3H), 1.64 (m, 2H), 1.45 (s, 3H). **^{13}C NMR** (100 MHz, CDCl$_3$) δ 170.6, 140.9, 120.0, 65.6, 60.6, 59.8, 28.5, 27.5, 23.2, 20.9, 16.0, 13.9.

Preparation of Compound SI-8:

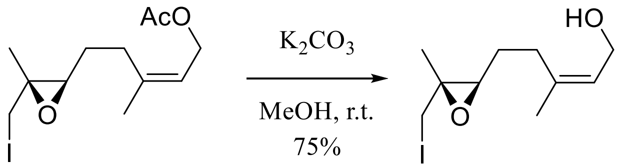

SI-7 **SI-8**

To a stirred solution of compound **SI-7** (25.0g, 73.9 mmol) in MeOH (300 mL) was added K_2CO_3 (5.11g, 37.0 mmol) at 0 °C, and stirred for 2 h. After completion, the organic solvent was removed in vacuo and the resulting mixture was diluted with H_2O (200 mL), the aqueous layer extracted with EtOAc (3 ×200 mL). The combined organic layers were washed with brine (200 mL), dried over anhydrous Na_2SO_4, filtered and concentrated under reduced pressure and the residue was purified by flash chromatography on silica gel (eluent: EtOAc/PE, 1/10 to 1/8) to afford compound **SI-8** (16.4 g, yield: 75%) as a yellow oil. Spectral data matched the reported literature values.[1]

Compound **SI-8**: **R$_f$** 0.2 (15:1 petroleum ether: ethyl acetate); **^{1}H NMR** (400 MHz, CDCl$_3$) δ 5.48 (t, J = 7.1 Hz, 1H), 4.13 (m, 2H), 3.21 (d, J = 9.9 Hz, 1H), 3.10 (d, J = 9.9 Hz, 1H), 2.89 (t, J = 9.9

Hz, 1H), 2.26 (m, 2H), 2.01 (s, 1H), 1.76 (s, 3H), 1.65 (m, 2H), 1.45 (s, 3H). **^{13}C NMR** (100 MHz, $CDCl_3$) δ 138.1, 125.5, 66.0, 60.4, 58.8, 28.4, 27.4, 23.3, 16.2, 13.9.

Preparation of Compound SI-9:

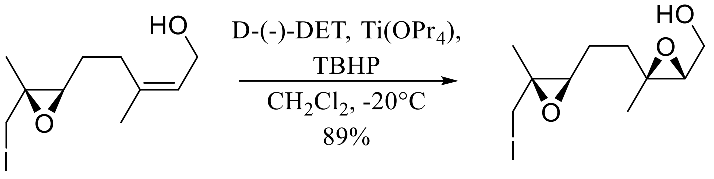

SI-8 **SI-9**

To 35.00 g of activated 4 Å molecular sieves, suspended in dry CH_2Cl_2 (200 mL), cooled to -10℃ internal, was added in order D-(-)-diethyl tartrate (10.1 g, 49.0 mmol), Ti(O-^{i}Pr)$_4$ (12.9 g, 45.3 mmol), and TBHP (30.1mL, 151 mmol, 5M in decane). After 20 min the mixture was cooled to -20℃, and a solution of **SI-8** (16.0 g, 54.0 mmol) in dry CH_2Cl_2 (100 mL) was added over 0.5 h. Upon completion of the reaction, as judged by TLC analysis, the mixture was quenched with 200 mL of distilled water and allowed to warm to rt. After 30 min of vigorous stirring, the solution was filtered through Celite. The organic phase was separated and the aqueous phase was extracted with CH_2Cl_2 (3 × 200 mL). The combined organic phases were washed with brine (200 mL), dried over anhydrous Na_2SO_4, filtered and concentrated under reduced pressure, the residue was purified by flash chromatography on silica gel (eluent: EtOAc/PE, 1/3 to 1/1) to afford compound **SI-9** (15.0 g, yield: 89%) as a colorless oil. Spectral data matched the reported literature values.[1]

Compound **SI-9**: **R$_f$** 0.3 (2:1 petroleum ether: ethyl acetate); **^{1}H NMR** (400 MHz, $CDCl_3$) δ 3.81-3.70 (m, 2H), 3.22 (d, J = 9.9 Hz, 1H), 3.10 (d, J = 9.9 Hz, 1H), 2.98 (t, J = 5.7 Hz, 1H), 2.90 (m, 1H), 2.57 (brs, 1H), 1.80-1.60 (m, 4H), 1.47 (s, 3H), 1.33 (s, 3H). **^{13}C NMR** (100 MHz, $CDCl_3$) δ 66.1, 63.9, 61.0, 60.7, 60.4, 29.5, 25.1, 21.9, 16.0, 13.4.

Preparation of Compound 16:

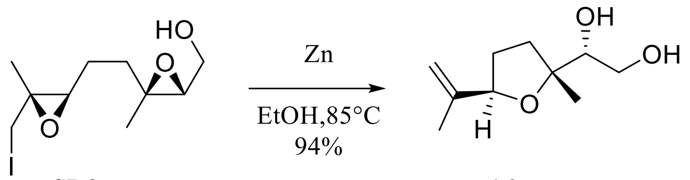

SI-9 **16**

To a stirred solution of **SI-9** (15.0 g, 48.1 mmol) in EtOH (300 mL) was added zinc powder (31.4 g, 481 mmol). The mixture was allowed to react at 85℃for 12 h. After completion, the reaction mixture was allowed to cool to room temperature. The mixture was diluted with Et_2O (200 mL) and filtered through Celite. The filtrate was washed with saturated aqueous $Na_2S_2O_3$ (200 mL). The Et_2O solution was separated, dried over Na_2SO_4 and concentrated under reduced pressure. The residue was purified by flash chromatography on silica gel (eluent: EtOAc/PE, 1/1 to 2/1) to afford compound **16** (8.41 g, yield: 94%) as a colorless oil. Spectral data matched the reported literature values.[1]

Compound **16**: **R$_f$** 0.2 (1:1 petroleum ether: ethyl acetate) **^{1}H NMR** (500 MHz, $CDCl_3$) δ 4.98 (s, 1H), 4.79 (s, 1H), 4.34(t, J = 7.3 Hz, 1H), 3.72 (m, 1H), 3.64 (m, 1H),3.55 (m, 1H), 3.00 (d, J = 5.5

Hz, 1H), 2.87 (d, J = 8.3 Hz, 1H), 2.16-2.01 (m, 2H), 1.78 (m, 1H), 1.69 (s, 3H), 1.67 (m, 1H),1.17 (s, 3H). **^{13}C NMR** (125 MHz, $CDCl_3$) δ 145.2, 110.6, 85.0, 83.6, 76.3, 63.2, 34.5, 30.9, 22.9, 17.7

Preparation of Compound 13:

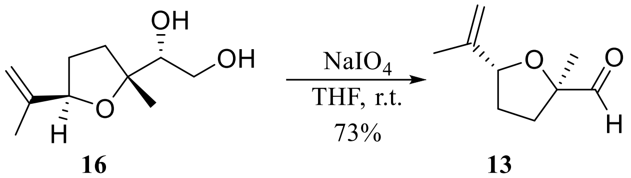

To a solution of **16** (8.0 g, 43.0 mmol) in THF: H_2O (2:1; 150 mL), $NaIO_4$ (11.0 g， 51.5 mmol) was added at 0 °C and stirred at room temperature for 1 h. The reaction mixture was filtered and washed with CH_2Cl_2 (3 × 100 mL). It was dried (Na_2SO_4) and evaporated to give aldehyde **13** (4.83 g, yield: 73%) as a yellow oil. The product was used without further purification.

Preparation of Compound 11:

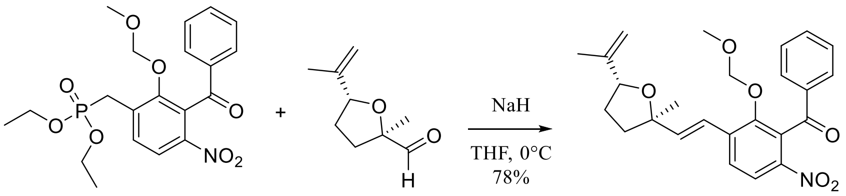

To a stirred solution of **12** (5.00 g, 11.4 mmol) in THF (150 mL) was added NaH (549 mg, 13.7 mmol) at 0 °C, and stirred for 0.5 h. A solution of **13** (2.64 g, 17.2 mmol) in THF (50 mL) was then added, and the resulting mixture was stirred for 2 h at the same temperature before being quenched with saturated aqueous NH_4Cl (150 mL) and extracted with EtOAc (3 × 150 mL). The extract was successively washed with brine (150 mL), dried (Na_2SO_4), filtered and concentrated under reduced pressure. The residue was purified by flash chromatography on silica gel (eluent: EtOAc/PE, 1/30 to 1/20) to afford compound **11** (3.90 g, yield: 78%) as a yellow oil.

Compound **11**: **R_f** 0.3 (10:1 petroleum ether : ethyl acetate); **IR** (KBr) νmax (cm^{-1}) 2971, 2931, 1682, 1523, 1453, 1387, 1342, 1316, 1287, 1254, 1163, 1100, 1086, 1000, 968, 901, 712; **^{1}H NMR** (400 MHz, $CDCl_3$) δ 8.03 (d, J = 8.7 Hz, 1H), 7.80 – 7.78 (m, 2H), 7.72 (d, J = 8.8 Hz, 1H), 7.58 (m, 1H), 7.45 (t, J = 7.7 Hz, 2H), 6.94 (d, J = 16.1 Hz, 1H), 6.52 (d, J = 16.1 Hz, 1H), 5.03 (s, 1H), 4.90 (d, J = 1.2 Hz, 2H), 4.82 (s, 1H), 4.46 (t, J = 6.9 Hz, 1H), 3.31 (s, 3H), 2.12 – 1.97 (m, 2H), 1.93 – 1.80 (m, 2H), 1.74 (s, 3H), 1.46 (s, 3H). **^{13}C NMR** (100 MHz, $CDCl_3$) δ 192.1, 152.6, 145.7, 145.2, 142.7, 138.9, 136.6, 133.8, 131.8, 128.9, 128.9, 127.7, 120.7, 120.3, 110.7, 101.2, 83.3, 82.5, 58.0, 37.7, 30.8, 27.1, 18.2; **HRMS (ESI)**: m/z calcd for $C_{25}H_{28}O_6N$ [M+H]$^+$: 438.1911; found: 438.1904.

Preparation of Compound 22:

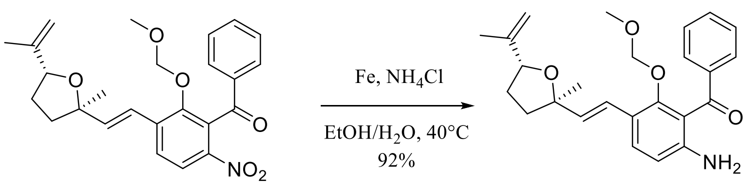

To a solution of **11** (3.85 g, 8.80 mmol) in EtOH/H_2O (80 mL/80 mL) was added NH_4Cl (1.88 g, 35.2 mol) and reduced iron powder (1.97 g, 35.2 mol). The mixture was allowed to react at 40℃for 5 h and filtered with diatomite to remove insoluble solid. The volatiles were then removed under reduced pressure. The residue was resolved in EtOAc (150 mL), washed sequentially with water (150 mL) and brine (150 mL), dried over anhydrous Na_2SO_4, filtered and concentrated under reduced pressure. The residue was purified by flash chromatography on silica gel (eluent: EtOAc/PE, 1/20 to 1/15) to afford compound **22** (3.30 g, yield: 92%) as a yellow oil.

Compound **22**: **R_f** 0.2 (10:1 petroleum ether : ethyl acetate); **IR** (KBr) vmax (cm^{-1}) 3374, 2970, 1649, 1616, 1575, 1542, 1521, 1484, 1451, 1426, 1396, 1372, 1320, 1279, 1214, 1158, 1120, 1076, 1013, 974, 925, 869, 816, 715; **^{1}H NMR** (400 MHz, $CDCl_3$) δ 7.83 (d, J = 7.1, 2H), 7.54 (m, 1H), 7.45 – 7.41 (m, 3H), 6.73 (d, J = 16.1 Hz, 1H), 6.54 (d, J = 8.5 Hz, 1H), 6.13 (d, J = 16.2 Hz, 1H), 5.02 (s, 1H), 4.80 (s, 1H), 4.69 (s, 2H), 4.51 (s, 2H), 4.43 (t, J = 6.8 Hz, 1H), 3.16 (s, 3H), 2.10 – 1.96 (m, 2H), 1.86 – 1.76 (m, 2H), 1.73 (s, 3H), 1.43 (s, 3H). **^{13}C NMR** (100 MHz, $CDCl_3$) δ 197.6, 154.3, 147.0, 146.2, 138.9, 134.3, 133.1, 129.9, 129.7, 128.4, 121.2, 120.3, 117.9, 113.2, 110.4, 100.4, 83.4, 82.2, 57.7, 37.7, 31.0, 27.4, 18.2; **HRMS (ESI)**: m/z calcd for $C_{25}H_{30}O_4N$ $[M+H]^+$: 408.2169; found: 408.2169.

Preparation of Compound 23 and 23a:

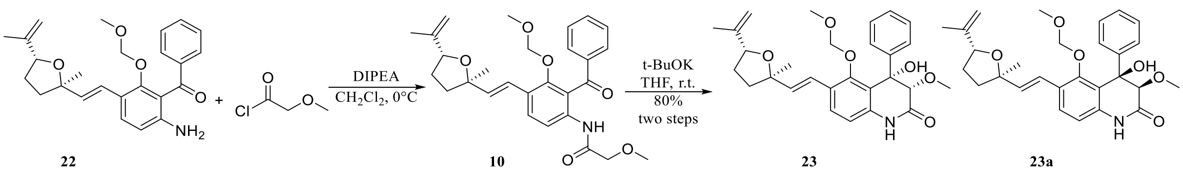

To a stirred solution of compound **22** (3.20 g, 7.85 mmol) and DIPEA (2.03 g，15.7 mmol) in dry CH_2Cl_2 (100 mL) was added methoxyacetyl chloride (1.28 g, 11.8 mmol) at 0°C. The reaction was removed from the ice bath and stirred at room temperature for 8 h. The mixture reaction was quenched with saturated aqueous NH_4Cl (100 mL), CH_2Cl_2 (100 mL) was added. The reaction mixture was partitioned between saturated NH_4Cl and CH_2Cl_2, and the aqueous layer extracted with CH_2Cl_2 (3 × 100 mL). The combined organic layers were washed with brine (100 mL), dried over anhydrous Na_2SO_4, filtered and concentrated under reduced pressure to yield product **10**. The product was used without further purification.

To a stirred solution of compound **10** in THF(100 mL) was added KO^{t}Bu (8.82 g, 78.6 mmol) at 0℃, and stirred for 4 h. The mixture reaction was quenched with saturated aqueous NH_4Cl (100 mL), EtOAc (100 mL) was added. The reaction mixture was partitioned between saturated NH_4Cl and EtOAc and the aqueous layer extracted with EtOAc (3 × 100 mL). The combined organic layers were washed with brine (100 mL), dried over anhydrous Na_2SO_4, filtered and concentrated under reduced pressure. The residue was purified by flash chromatography on silica gel (eluent: EtOAc/PE,

1/2 to 1/1) to give compound **23** and **23a** (3.00 g, yield: 80%) as a white solid.

Compound **23**: **R$_f$** 0.3 (2:3 petroleum ether : ethyl acetate); **[α]$_D$^{25}**= +22 (c = 0.7, MeOH);**IR** (KBr) vmax (cm^{-1}) 3968, 1699, 1600, 1485, 1452, 1391, 1375, 1164, 1103, 1057, 1048, 932; **^{1}H NMR** (500 MHz, acetone-*d*$_6$) *δ* 9.32 (s, 1H), 7.57 (d, *J* = 8.4 Hz, 1H), 7.40 (m, 2H), 7.32 (m, 2H), 7.27 (m, 1H), 6.87 (overlapped, 2H), 6.26 (d, *J* = 16.1 Hz, 1H), 5.19 (s, 1H), 4.98 (s, 1H), 4.74 (s, 1H), 4.65 (d, *J* = 2.1 Hz, 2H), 4.42 (t, J = 6.9 Hz, 1H), 3.74 (d, *J* = 0.7 Hz, 1H), 3.40 (s, 3H), 3.27 (s, 3H), 2.12 – 2.07 (m, 1H), 2.03 – 1.98 (m, 1H), 1.85 – 1.80 (m, 1H),1.79 – 1.72 (m, 1H), 1.70 (s, 3H), 1.39 (s, 3H). **^{13}C NMR** (125 MHz, acetone-*d*$_6$) *δ* 167.5, 155.1, 147.5, 143.9, 138.8, 136.6, 128.9, 128.4, 128.0, 127.9, 127.0, 122.1, 122.0, 113.1, 110.0, 101.2, 86.5, 83.9, 82.6, 78.8, 60.1, 57.9, 38.2, 31.5, 27.7, 18.3; **HRMS (ESI)**: m/z calcd for $C_{28}H_{34}O_6N$ [M+H]$^+$: 480.2381; found: 480.2372.

Compound **23a**: **R$_f$** 0.3 (2:3 petroleum ether : ethyl acetate); **[α]$_D$^{25}**= -19 (c = 0.7, MeOH);**IR** (KBr) vmax (cm^{-1}) 3968, 1699, 1600, 1485, 1452, 1391, 1375, 1164, 1103, 1057, 1048, 932; **^{1}H NMR** (500 MHz, acetone-*d*$_6$) *δ* 9.32 (s, 1H), 7.57 (d, *J* = 8.4 Hz, 1H), 7.40 (m, 2H), 7.32 (m, 2H), 7.27 (d, J = 7.2 Hz, 1H), 6.87 (overlapped, 2H), 6.26 (d, *J* = 16.1 Hz, 1H), 5.18 (s, 1H), 4.98 (s, 1H), 4.74 (s, 1H), 4.66 (d, *J* = 2.6 Hz, 2H), 4.44 (t, *J* = 6.9 Hz, 1H), 3.76 (s, 1H), 3.40 (s, 3H), 3.27 (s, 3H), 2.12 – 2.07 (m, 1H), 2.03 – 1.98 (m, 1H), 1.85 – 1.80 (m, 1H),1.79 – 1.72 (m, 1H), 1.70 (s, 3H), 1.38 (s, 3H). **^{13}C NMR** (125 MHz, acetone-*d*$_6$) *δ* 167.5, 155.1, 147.5, 143.9, 138.8, 136.5, 128.9, 128.4, 127.9, 127.8, 127.0, 122.1, 122.0, 113.1, 110.0, 101.2, 86.5, 83.9, 82.6, 78.7, 60.1, 57.9, 38.2, 31.5, 27.7, 18.3; **HRMS (ESI)**: m/z calcd for $C_{28}H_{34}O_6N$ [M+H]$^+$: 480.2381; found: 480.2372.

Preparation of (+)-aniduquinolone A (1):

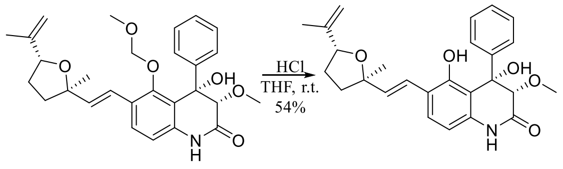

23 **1**

To a stirred solution of compound **23** (1.50 g) in THF (100 mL) was added concentrated HCl (3 mL) at room temperature, the mixture was stirred for 1 h. The solution was neutralized with $NaHCO_3$ and the aqueous layer was extracted with EtOAc (3×100 mL). The combined organic layers were washed with brine (100 mL), dried over anhydrous Na_2SO_4, filtered and concentrated in vacuo. The residue was purified by flash chromatography on silica gel (eluent: EtOAc/PE, 1/3 to 1/1) to give (+)-aniduquinolone A (**1**) (735 mg, yield: 54%) as a white solid.

(+)-aniduquinolone A (**1**): **R$_f$** 0.4 (2:3 petroleum ether : ethyl acetate); **[α]$_D$^{25}**= +56 (c = 0.7, MeOH); **IR** (KBr) vmax (cm^{-1}) 3288, 2968, 2936, 1690, 1617, 1601, 1507, 1494, 1451, 1420, 1375, 1343, 1318, 1293, 1223, 1188, 1180, 1101, 1081, 1029, 977, 902, 808, 699; **^{1}H NMR** (500 MHz, acetone-*d*$_6$) *δ* 9.60 (s, 1H), 9.34 (s, 1H), 7.43 (d, *J* = 8.3 Hz, 1H), 7.35 (m, 5H), 6.81 (d, *J* = 16.2 Hz, 1H), 6.58 (d, *J* = 8.3 Hz, 1H), 6.34 (s, 1H), 6.28 (d, *J* = 16.2 Hz, 1H), 4.99 (s, 1H), 4.74 (s, 1H), 4.40 (t, *J* = 6.8 Hz, 1H), 3.67 (d, *J* = 1.4 Hz, 1H), 3.52 (s, 3H), 2.14-2.05 (m, 1H), 2.02-1.93 (m, 1H), 1.86-1.78 (m, 1H), 1.78-1.72 (m, 1H), 1.71 (s, 3H), 1.37 (s, 3H). **^{13}C NMR** (125 MHz, acetone-*d*$_6$) *δ* 166.2, 156.3, 147.6, 140.1, 137.1, 135.3, 129.7, 129.5, 128.1, 127.4, 121.6, 121.4, 112.1, 109.9, 107.7, 84.0, 82.5, 80.0, 58.9, 38.3, 31.6, 27.9, 18.3. **^{1}H NMR** (600 MHz, DMSO-*d*$_6$) *δ* 10.30 (s, 1H), 7.34 (m, 4H), 7.20 (dd, *J* = 7.9, 1.7 Hz, 2H), 6.65 (d, *J* = 16.2 Hz, 1H), 6.44 (d, *J* = 8.3 Hz, 1H), 6.24 (d, *J* = 16.2 Hz, 1H), 4.96 (s, 1H), 4.75 (s, 1H), 4.33 (t, *J* = 6.8 Hz, 1H), 3.60 (d, *J* = 1.3 Hz, 1H), 3.44 (s, 3H), 2.03-1.98 (m, 1H), 1.94-1.90 (m, 1H), 1.77-1.73 (m, 1H), 1.72-1.68 (m, 1H), 1.67

(s, 3H), 1.33 (s, 3H). **^{13}C NMR** (150 MHz, DMSO-d_6) δ 166.1, 154.7, 146.2, 139. 8, 136.0, 134.2, 128.6, 128.6, 126.8, 126.2, 120.1, 119.4, 111.1, 109.7, 106.9, 84.2, 82.9, 81.2, 78.6, 58.4, 37.3, 30.3, 27.4, 18.0; **HRMS (ESI)**: m/z calcd for $C_{26}H_{30}O_5N$ $[M+H]^+$: 436.2118; found: 436.2111.

Preparation of SI-11:

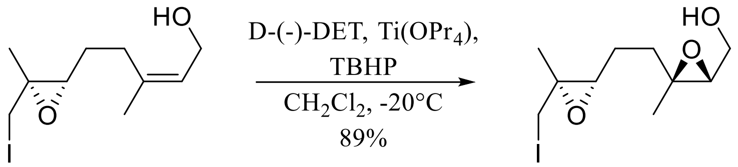

SI-10 **SI-11**

To 25.0 g of activated 4 Å molecular sieves, suspended in dry CH_2Cl_2 (150 mL), cooled to -10℃ internal, was added in order D-(-)-diethyl tartrate (5.24 g, 25.4 mmol), Ti(O-^{i}Pr)$_4$ (6.67 g, 23.5 mmol), and TBHP (15.6 mL, 78.2 mmol, 5M in decane). After 20 min the mixture was cooled to -20℃, and a solution of **SI-10** (8.30 g, 28.0 mmol) in dry CH_2Cl_2 (50 mL) was added over 0.5 h. Upon completion of the reaction, as judged by TLC analysis, the mixture was quenched with 200 mL of distilled water and allowed to warm to rt. After 30 min of vigorous stirring, the solution was filtered through Celite. The organic phase was separated and the aqueous phase was extracted with CH_2Cl_2 (3 × 100 mL). The combined organic phases were washed with brine (100 mL), dried over anhydrous Na_2SO_4, filtered and concentrated under reduced pressure, the residue was purified by flash chromatography on silica gel (eluent: EtOAc/PE, 1/3 to 1/1) to afford compound **SI-11** (7.79 g, yield: 89%) as a colorless oil. Spectral data matched the reported literature values.[1]
Compound **SI-11**: **R$_f$** 0.3 (2:1 petroleum ether: ethyl acetate); **^{1}H NMR** (400 MHz, $CDCl_3$) δ 3.77 (m, 2H), 3.25 (d, J = 9.9 Hz, 1H), 3.09 (d, J = 9.9 Hz, 1H), 3.00 (t, J = 5.7 Hz, 1H), 2.93 (t, J = 5.9 Hz, 1H), 2.48 (s, 1H), 1.83-1.63 (m, 4H), 1.48 (s, 3H), 1.37 (s, 3H). **^{13}C NMR** (100 MHz, $CDCl_3$) δ 65.3, 63.9, 60.7, 60.7, 60.6, 29.1, 25.0, 21.9, 15.9, 13.3.

Preparation of 16a:

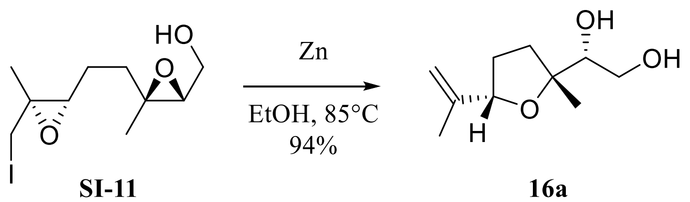

SI-11 **16a**

To a stirred solution of **SI-11** (5.50 g, 27.2 mmol) in EtOH (80 mL) was added zinc powder (17.8 g, 272 mmol). The mixture was allowed to react at 85℃for 12 h. After completion, the reaction mixture was allowed to cool to room temperature. The mixture was diluted with Et_2O (100 mL) and filtered through Celite. The filtrate was washed with saturated aqueous $Na_2S_2O_3$ (100 mL). The Et_2O solution was separated, dried over Na_2SO_4 and concentrated under reduced pressure. The residue was purified by flash chromatography on silica gel (eluent: EtOAc/PE, 1/1 to 2/1) to afford compound **16a** (4.76 g, yield: 94%) as a colorless oil. Spectral data matched the reported literature values.[1]

Compound **216a**: **R$_f$** 0.2 (1:1 petroleum ether: ethyl acetate) **^{1}H NMR** (500 MHz, $CDCl_3$) δ 4.94 (s, 1H), 4.79 (s, 1H), 4.36(t, J = 7.3 Hz, 1H), 3.65 (m, 2H), 3.54 (m, 1H), 2.98 (d, J = 5.5 Hz, 1H), 2.89 (d, J = 8.3 Hz, 1H), 2.10-2.01 (m, 2H), 1.76-1.61 (overlapped, 5H), 1.18 (s, 3H). **^{13}C NMR** (125 MHz, $CDCl_3$) δ 144.9, 110.9, 85.0, 81.9, 77.0, 63.5, 35.0, 31.4, 22.48, 18.2.

Preparation of Compound 13a:

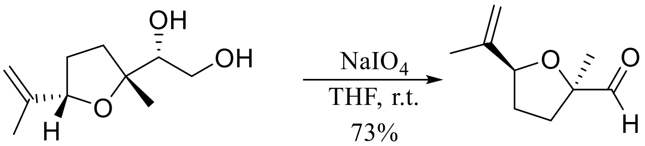

16a **13a**

To a solution of **16a** (2.80 g, 15.0 mmol) in THF: H_2O (2:1; 80 mL), $NaIO_4$ (3.86 g，18.0 mmol) was added at 0 °C and the reaction mixture was stirred at room temperature for 1 h. Upon completion, the reaction mixture was filtered and extracted with CH_2Cl_2 (3 × 100 mL). The organic layers were dried (Na_2SO_4) and evaporated to give aldehyde **13a** (1.70 g, yield: 73%) as a yellow oil. The product was used without further purification.

Preparation of Compound SI-12:

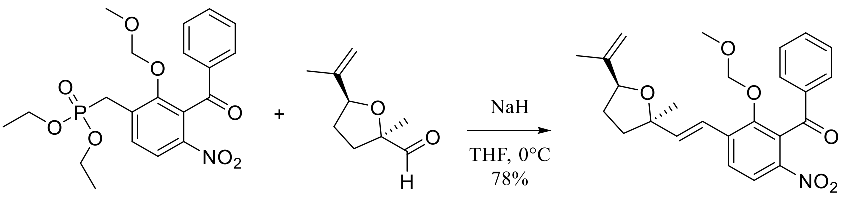

11 **13a** **SI-12**

To a stirred solution of **11** (600 mg, 1.37 mmol) in THF (20 mL) was added NaH (65.8 mg, 1.65 mmol) at 0 °C and stirred for 0.5 h[2]. A solution of **13a** (317 mg, 2.06 mmol) in THF (5 mL) was then added, and the resulting mixture was stirred for 2 h at the same temperature before being quenched with saturated aqueous NH_4Cl (50 mL) and extracted with EtOAc (3 × 50 mL). The extracts were successively washed with brine (50 mL), dried (Na_2SO_4), filtered and concentrated under reduced pressure. The residue was purified by flash chromatography on silica gel (eluent: EtOAc/PE, 1/30 to 1/20) to afford compound **SI-12** (470 mg, yield: 78%) as a yellow oil.

Compound **SI-12**: **R$_f$** 0.3 (10:1 petroleum ether : ethyl acetate); **IR** (KBr) vmax (cm^{-1}) 2971, 2931, 1682, 1523, 1453, 1387, 1342, 1316, 1287, 1254, 1163, 1100, 1086, 1000, 968, 901, 712; **^{1}H NMR** (400 MHz, $CDCl_3$) δ 8.03 (d, J = 8.8 Hz, 1H), 7.79 (m, 2H), 7.71 (d, J = 8.8 Hz, 1H), 7.58 (t, J = 7.4 Hz, 1H), 7.45 (t, J = 7.7 Hz, 2H), 6.93 (d, J = 16.2 Hz, 1H), 6.57 (d, J = 16.2 Hz, 1H), 5.08 (s, 1H), 4.89 (s, 2H), 4.83 (s, 1H), 4.48 (dd, J = 8.4, 6.3 Hz, 1H), 3.25 (s, 3H), 2.08 (m, 2H), 1.94 (m, 1H), 1.75 (m, 4H), 1.45 (s, 3H). **^{13}C NMR** (100 MHz, $CDCl_3$) δ 192.0, 152.6, 145.3, 143.1, 139.0, 136.7, 133.8, 129.0, 129.0, 128.9, 127.8, 120.7, 120.5, 110.7, 101.0, 82.9, 82.4, 77.4, 58.0, 38.5, 31.1, 26.9, 18.7; **HRMS (ESI)**: m/z calcd for $C_{25}H_{28}O_6N$ $[M+H]^+$: 438.1911; found: 438.1904.

Preparation of Compound SI-13:

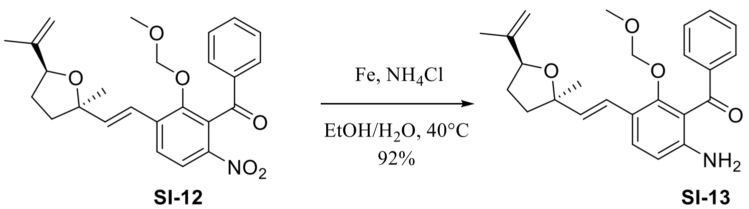

To a solution of **SI-12** (450 mg, 1.03 mmol) in $EtOH/H_2O$ (40 mL/40 mL) was added NH_4Cl (220 mg, 4.11 mmol) and reduced iron powder (230 mg, 4.11 mmol). The mixture was allowed to react at 40℃ for 5 h and filtered with diatomite to remove insoluble solid. The volatiles were then removed under reduced pressure. The residue was resolved in EtOAc (50 mL), washed sequentially with water (50 mL) and brine (50 mL), dried over anhydrous Na_2SO_4, filtered and concentrated under reduced pressure. The residue was purified by flash chromatography on silica gel (eluent: EtOAc/PE, 1/20 to 1/15) to afford compound **SI-13** (380 mg, yield: 91%) as a yellow oil.

Compound **SI-13**: **R_f** 0.2 (10:1 petroleum ether: ethyl acetate); **IR** (KBr) vmax (cm^{-1}) 3374, 2970, 1649, 1616, 1575, 1542, 1521, 1484, 1451, 1426, 1396, 1372, 1320, 1279, 1214, 1158, 1120, 1076, 1013, 974, 925, 869, 816, 715; **^{1}H NMR** (400 MHz, $CDCl_3$) δ 7.83 (m, 2H), 7.54 (m, 1H), 7.43 (m, 3H), 6.74 (d, J = 16.3 Hz, 1H), 6.54 (d, J = 8.5 Hz, 1H), 6.20 (d, J = 16.3 Hz, 1H), 5.07 (s, 1H), 4.80 (s, 1H), 4.68 (s, 2H), 4.48 (overlapped, 3H), 3.13 (s, 3H), 2.06 (m, 2H), 1.87 (m, 1H), 1.74 (overlapped, 4H), 1.42 (s, 3H). **^{13}C NMR** (100 MHz, $CDCl_3$) δ 197.6, 154.3, 146.9, 145.8, 138.8, 134.8, 133.1, 129.8, 128.4, 121.1, 120.4, 118.0, 113.2, 110.5, 100.3, 83.1, 82.2, 77.4, 57.7, 38.6, 31.3, 26.9, 18.5; **HRMS (ESI)**: m/z calcd for $C_{25}H_{30}O_4N$ $[M+H]^+$: 408.2169; found: 408.2169.

Preparation of Compound 24 and 24a:

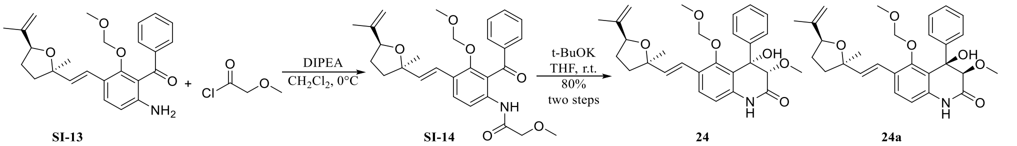

To a stirred solution of compound **SI-13** (370 mg, 0.908 mmol) and DIPEA (235 mg， 1.82 mmol) in dry CH_2Cl_2 (50 mL) was added methoxyacetyl chloride (148 mg, 1.36 mmol) at 0°C. The reaction was removed from the ice bath and stirred at room temperature for 8 h. The reaction mixture was quenched with saturated aqueous NH_4Cl (50 mL), CH_2Cl_2 (50 mL) was added. The reaction mixture was partitioned between saturated NH_4Cl and CH_2Cl_2 and the aqueous layer extracted with CH_2Cl_2 (3 × 50 mL). The combined organic layers were washed with brine (50 mL), dried over anhydrous Na_2SO_4, filtered and concentrated under reduced pressure to yield product **SI-14**. The product was used without further purification.

To a stirred solution of compound **SI-14** in THF(50 mL) was added KO^{t}Bu (1.02 g, 9.08 mmol) at 0℃, and stirred for 4 h. The mixture reaction was quenched with saturated aqueous NH_4Cl (50 mL), EtOAc (50 mL) was added. The reaction mixture was partitioned between saturated NH_4Cl and EtOAc and the aqueous layer extracted with EtOAc (3 × 50 mL). The combined organic layers were washed with brine (40 mL), dried over anhydrous Na_2SO_4, filtered and concentrated under reduced pressure. The residue was purified by flash chromatography on silica gel (eluent: EtOAc/PE, 1/2 to 1/1) to give compound **24** and **24a** (330 mg, yield: 76%) as a white solid.

Compound **24**: $\mathbf{R_f}$ 0.3 (2:3 petroleum ether : ethyl acetate); $[\alpha]_D^{25}$= +39 (c = 0.7, MeOH); **IR** (KBr) vmax (cm^{-1}) 3968, 1699, 1600, 1485, 1452, 1391, 1375, 1164, 1103, 1057, 1048, 932; ^{1}**H NMR** (400 MHz, acetone-d_6) δ 9.31 (s, 1H), 7.55 (d, *J* = 8.3 Hz, 1H), 7.38 (m, 2H), 7.30 (m, 3H), 6.87(overlapped, 2H), 6.33 (d, *J* = 16.2 Hz, 1H), 5.20 (s, 1H), 5.04 (s, 1H), 4.75 (s, 1H), 4.67 (d, *J* = 2.1 Hz, 2H), 4.43 (t, *J* = 6.9 Hz, 1H), 3.74 (s, 1H), 3.40 (s, 3H), 3.23 (s, 3H), 2.17 – 2.09 (m, 1H), 2.05 – 2.01 (m, 1H), 1.95 – 1.85 (m, 1H), 1.78 (m, 1H), 1.72 (s, 3H), 1.37 (s, 3H). ^{13}**C NMR** (100 MHz, acetone-d_6) δ 167.5, 155.2, 147.1, 143.9, 138.9, 137.4, 128.9, 128.4, 128.0, 128.0, 127.1, 122.2, 121.9, 113.1, 110.1, 101.2, 86.5, 83.5, 82.5, 78.8, 60.1, 57.9, 39.0, 31.9, 27.1, 18.7; **HRMS (ESI)**: m/z calcd for $C_{28}H_{34}O_6N$ $[M+H]^+$: 480.2381; found: 480.2372.

Compound **24a**: $\mathbf{R_f}$ 0.3 (2:3 petroleum ether : ethyl acetate); $[\alpha]_D^{25}$= -8 (c = 0.7, MeOH);**IR** (KBr) vmax (cm^{-1}) 3968, 1699, 1600, 1485, 1452, 1391, 1375, 1164, 1103, 1057, 1048, 932; ^{1}**H NMR** (400 MHz, acetone-d_6) δ 9.30 (s, 1H), 7.55 (d, *J* = 8.4 Hz, 1H), 7.38 (m, 2H), 7.32 (m, 3H), 6.87 (overlapped, 2H), 6.33 (d, *J* = 16.2 Hz, 1H), 5.23 (s, 1H), 5.05 (s, 1H), 4.76 (s, 1H), 4.65 (s, 2H), 4.43 (t, *J* = 6.9 Hz, 1H), 3.72 (s, 1H), 3.41 (s, 3H), 3.24 (s, 3H), 2.16 – 2.08 (m, 1H), 2.04 – 1.99 (m, 1H), 1.91 (m, 1H), 1.76 (m, 1H), 1.73 (s, 3H), 1.37 (s, 3H). ^{13}**C NMR** (101 MHz, acetone-d_6) δ 167.4, 155.1, 147.1, 143.8, 138.9, 137.4, 129.0, 128.5, 128.0, 128.0, 127.1, 122.2, 121.9, 113.1, 110.2, 101.1, 86.6, 83.6, 82.6, 78.9, 60.0, 57.9, 39.0, 31.8, 27.1, 18.7; **HRMS (ESI)**: m/z calcd for $C_{28}H_{34}O_6N$ $[M+H]^+$: 480.2381; found: 480.2372.

Preparation of 25:

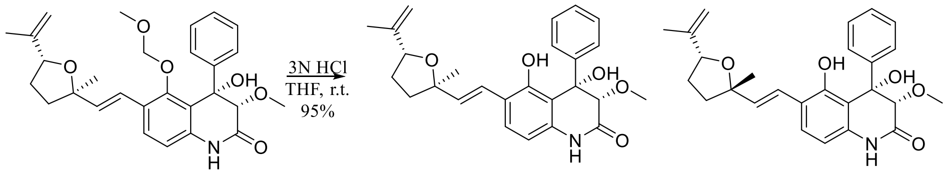

23 (+)-**1** **25**

To a stirred solution of compound **23** (70.0 mg, 0.146 mmol) in THF (10 mL) was added concentrated 3N HCl (0.3 mL) at room temperature, the mixture was stirred for 1 h. The solution was neutralized with $NaHCO_3$ and the aqueous layer was extracted with EtOAc (3×50 mL). The combined organic layers were washed with brine (50 mL), dried over anhydrous Na_2SO_4, filtered and concentrated in vacuo. The residue was purified by flash chromatography on silica gel (eluent: EtOAc/PE, 1/3 to 1/1) to give (+)-aniduquinolone A (**1**) and **25** and (59.0 mg, yield: 93%) as a white solid.

19-*epi*-aniduquinolone A (**25**): $\mathbf{R_f}$ 0.4 (2:3 petroleum ether: ethyl acetate); $[\alpha]_D^{25}$= +32 (c = 0.7, MeOH); **IR** (KBr) vmax (cm^{-1}) 3288, 2968, 2936, 1690, 1617, 1601, 1507, 1494, 1451, 1420, 1375, 1343, 1318, 1293, 1223, 1188, 1180, 1101, 1081, 1029, 977, 902, 808, 699; ^{1}**H NMR** (500 MHz, acetone-d_6) δ 9.58 (s, 1H), 9.34 (s, 1H), 7.42 (d, *J* = 8.3 Hz, 1H), 7.35 (m, 5H), 6.87 (d, *J* = 16.2 Hz, 1H), 6.57 (d, *J* = 8.3 Hz, 1H), 6.35 (s, 1H), 6.34 (d, *J* = 16.2 Hz, 1H), 5.05 (s, 1H), 4.74 (s, 1H), 4.42 (t, *J* = 7.2 Hz, 1H), 3.66 (d, *J* = 1.4 Hz, 1H), 3.51 (s, 3H), 2.11-2.06 (m, 1H), 2.02-1.97 (m, 1H), 1.91-1.86 (m, 1H), 1.77-1.70 (m, 1H), 1.72 (s, 3H), 1.35 (s, 3H). ^{13}**C NMR** (125 MHz, acetone-d_6) δ 166.2, 156.3, 147.2, 140.1, 137.1, 136.0, 129.7, 129.5, 127.9, 127.4, 121.6, 112.1, 110.2, 107.7, 85.7, 83.6, 82.7, 80.0, 58.9, 39.2, 31.9, 27.4, 18.6; **HRMS (ESI)**: m/z calcd for $C_{26}H_{30}O_5N$ $[M+H]^+$: 436.2118; found: 436.2111.

Preparation of 26 and 27:

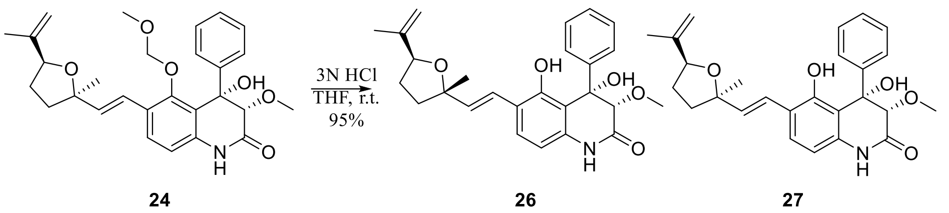

To a stirred solution of compound **24** (80.0 mg, 0.167 mmol) in THF (10 mL) was added concentrated 3N HCl (0.3 mL) at room temperature, the mixture was stirred for 1 h. The solution was neutralized with $NaHCO_3$ and the aqueous layer was extracted with EtOAc (3×50 mL). The combined organic layers were washed with brine (50 mL), dried over anhydrous Na_2SO_4, filtered and concentrated in vacuo. The residue was purified by flash chromatography on silica gel (eluent: EtOAc/PE, 1/3 to 1/1) to give **26** and **27** and (68.0 mg, yield: 94%) as a white solid.

Compound **26**: $\mathbf{R_f}$ 0.4 (2:3 petroleum ether : ethyl acetate); $[\alpha]_D^{25}$= +50 (c = 0.7, MeOH); **IR** (KBr) vmax (cm^{-1}) 3288, 2968, 2936, 1690, 1617, 1601, 1507, 1494, 1451, 1420, 1375, 1343, 1318, 1293, 1223, 1188, 1180, 1101, 1081, 1029, 977, 902, 808, 699; **^{1}H NMR** (500 MHz, acetone-d_6) δ 9.60 (s, 1H), 9.34 (s, 1H), 7.43 (d, J = 8.3 Hz, 1H), 7.35 (m, 5H), 6.81 (d, J = 16.2 Hz, 1H), 6.58 (d, J = 8.3 Hz, 1H), 6.34 (s, 1H), 6.27 (d, J = 16.2 Hz, 1H), 4.99 (s, 1H), 4.74 (s, 1H), 4.40 (t, J = 6.8 Hz, 1H), 3.67 (d, J = 1.4 Hz, 1H), 3.52 (s, 3H), 2.14-2.05 (m, 1H), 2.02-1.93 (m, 1H), 1.86-1.78 (m, 1H), 1.78-1.72 (m, 1H), 1.70 (s, 3H), 1.37 (s, 3H). **^{13}C NMR** (125 MHz, acetone-d_6) δ 166.2, 156.3, 147.6, 140.2, 137.1, 135.3, 129.7, 129.5, 128.0, 127.4, 121.49, 121.47, 112.1, 109.9, 107.7, 85.7, 84.0, 82.5, 80.0, 58.9, 38.3, 31.6, 27.9, 18.3; **HRMS (ESI)**: m/z calcd for $C_{26}H_{30}O_5N$ $[M+H]^+$: 436.2118; found: 436.2111.

Compound **27**: $\mathbf{R_f}$ 0.4 (2:3 petroleum ether : ethyl acetate); $[\alpha]_D^{25}$= +70 (c = 0.7, MeOH);**IR** (KBr) vmax (cm^{-1}) 3288, 2968, 2936, 1690, 1617, 1601, 1507, 1494, 1451, 1420, 1375, 1343, 1318, 1293, 1223, 1188, 1180, 1101, 1081, 1029, 977, 902, 808, 699; **^{1}H NMR** (500 MHz, acetone-d_6) δ 9.58 (s, 1H), 9.33 (s, 1H), 7.41 (d, J = 8.3 Hz, 1H), 7.35 (m, 5H), 6.87 (d, J = 16.2 Hz, 1H), 6.57 (d, J = 8.3 Hz, 1H), 6.35 (d, J = 16.3 Hz, 2H), 6.33 (overlapped, 1H), 5.05 (s, 1H), 4.75 (s, 1H), 4.42 (t, J = 7.2 Hz, 1H), 3.67 (d, J = 1.5 Hz, 1H), 3.52 (s, 3H), 2.11-2.06 (m, 1H), 2.02-1.97 (m, 1H), 1.91-1.86 (m, 1H), 1.77-1.70 (m, 1H), 1.73 (s, 3H), 1.35 (s, 3H). **^{13}C NMR** (125 MHz, acetone-d_6) δ 166.2, 156.3, 147.2, 140.1, 137.1, 136.0, 129.7, 129.5, 127.9, 127.4, 121.7, 121.6, 112.1, 110.2, 107.7, 85.7, 83.6, 82.7, 80.0, 58.9, 39.3, 31.9, 27.4, 18.7; **HRMS (ESI)**: m/z calcd for $C_{26}H_{30}O_5N$ $[M+H]^+$: 436.2118; found: 436.2111.

Preparation of 28 and 29:

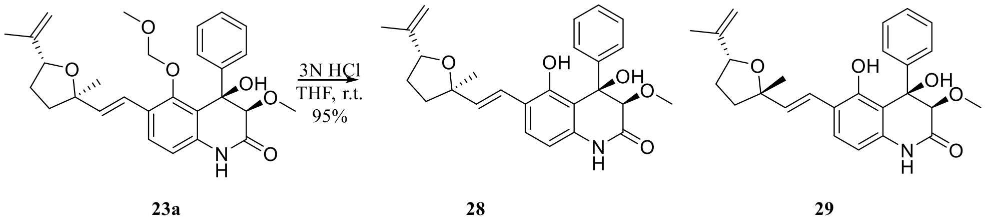

To a stirred solution of compound **23a** (70.0 mg, 0.146 mmol) in THF (10 mL) was added concentrated 3N HCl (0.3 mL) at room temperature, the mixture was stirred for 1 h. The solution was neutralized with $NaHCO_3$ and the aqueous layer was extracted with EtOAc (3×50 mL). The

combined organic layers were washed with brine (50 mL), dried over anhydrous Na_2SO_4, filtered and concentrated in vacuo. The residue was purified by flash chromatography on silica gel (eluent: EtOAc/PE, 1/3 to 1/1) to give **28** and **29** (58.0 mg, yield: 91%) as a white solid.

Compound **28**: $\mathbf{R_f}$ 0.4 (2:3 petroleum ether : ethyl acetate); $[\alpha]_D^{25}$= -60 (c = 0.7, MeOH); **IR** (KBr) vmax (cm^{-1}) 3288, 2968, 2936, 1690, 1617, 1601, 1507, 1494, 1451, 1420, 1375, 1343, 1318, 1293, 1223, 1188, 1180, 1101, 1081, 1029, 977, 902, 808, 699; **^{1}H NMR** (500 MHz, acetone-d_6) δ 9.60 (s, 1H), 9.34 (s, 1H), 7.43 (d, J = 8.3 Hz, 1H), 7.35 (m, 5H), 6.81 (d, J = 16.2 Hz, 1H), 6.58 (d, J = 8.3 Hz, 1H), 6.34 (s, 1H), 6.27 (d, J = 16.2 Hz, 1H), 4.99 (s, 1H), 4.74 (s, 1H), 4.40 (t, J = 6.8 Hz, 1H), 3.67 (d, J = 1.4 Hz, 1H), 3.52 (s, 3H), 2.14-2.05 (m, 1H), 2.02-1.93 (m, 1H), 1.86-1.78 (m, 1H), 1.78-1.72 (m, 1H), 1.70 (s, 3H), 1.37 (s, 3H). **^{13}C NMR** (125 MHz, acetone-d_6) δ 166.2, 156.3, 147.6, 140.2, 137.1, 135.3, 129.7, 129.5, 128.0, 127.4, 121.49, 121.47, 112.1, 109.9, 107.7, 85.7, 84.0, 82.5, 80.0, 58.9, 38.3, 31.6, 27.9, 18.3; **HRMS (ESI)**: m/z calcd for $C_{26}H_{30}O_5N$ $[M+H]^+$: 436.2118; found: 436.2111.

Compound **29**: $\mathbf{R_f}$ 0.4 (2:3 petroleum ether : ethyl acetate); $[\alpha]_D^{25}$= -56 (c = 0.7, MeOH); **IR** (KBr) vmax (cm^{-1}) 3288, 2968, 2936, 1690, 1617, 1601, 1507, 1494, 1451, 1420, 1375, 1343, 1318, 1293, 1223, 1188, 1180, 1101, 1081, 1029, 977, 902, 808, 699; **^{1}H NMR** (500 MHz, acetone-d_6) δ 9.58 (s, 1H), 9.33 (s, 1H), 7.41 (d, J = 8.3 Hz, 1H), 7.35 (m, 5H), 6.87 (d, J = 16.2 Hz, 1H), 6.57 (d, J = 8.3 Hz, 1H), 6.35 (d, J = 16.3 Hz, 2H), 6.33 (overlapped, 1H), 5.05 (s, 1H), 4.75 (s, 1H), 4.42 (t, J = 7.2 Hz, 1H), 3.67 (d, J = 1.5 Hz, 1H), 3.52 (s, 3H), 2.11-2.06 (m, 1H), 2.02-1.97 (m, 1H), 1.91-1.86 (m, 1H), 1.77-1.70 (m, 1H), 1.73 (s, 3H), 1.35 (s, 3H). **^{13}C NMR** (125 MHz, acetone-d_6) δ 166.2, 156.3, 147.2, 140.1, 137.1, 136.0, 129.7, 129.5, 127.9, 127.4, 121.7, 121.6, 112.1, 110.2, 107.7, 85.7, 83.6, 82.7, 80.0, 58.9, 39.3, 31.9, 27.4, 18.7; **HRMS (ESI)**: m/z calcd for $C_{26}H_{30}O_5N$ $[M+H]^+$: 436.2118; found: 436.2111.

Preparation of 30 and 31:

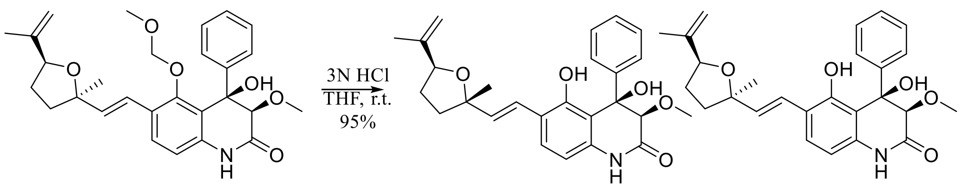

24a **30** **31**

To a stirred solution of compound **24a** (80.0 mg, 0.167 mmol) in THF (10 mL) was added concentrated 3N HCl (0.3 mL) at room temperature, the mixture was stirred for 1 h. The solution was neutralized with $NaHCO_3$ and the aqueous layer was extracted with EtOAc (3×50 mL). The combined organic layers were washed with brine (50 mL), dried over anhydrous Na_2SO_4, filtered and concentrated in vacuo. The residue was purified by flash chromatography on silica gel (eluent: EtOAc/PE, 1/3 to 1/1) to give **30** and 3**1** and (68.0 mg, yield: 94%) as a white solid.

Compound **30**: $\mathbf{R_f}$ 0.4 (2:3 petroleum ether : ethyl acetate); $[\alpha]_D^{25}$= -65 (c = 0.7, MeOH); **IR** (KBr) vmax (cm^{-1}) 3288, 2968, 2936, 1690, 1617, 1601, 1507, 1494, 1451, 1420, 1375, 1343, 1318, 1293, 1223, 1188, 1180, 1101, 1081, 1029, 977, 902, 808, 699; **^{1}H NMR** (500 MHz, acetone-d_6) δ 9.60 (s, 1H), 9.34 (s, 1H), 7.43 (d, J = 8.3 Hz, 1H), 7.35 (m, 5H), 6.81 (d, J = 16.2 Hz, 1H), 6.58 (d, J = 8.3 Hz, 1H), 6.34 (s, 1H), 6.28 (d, J = 16.2 Hz, 1H), 4.99 (s, 1H), 4.74 (s, 1H), 4.40 (t, J = 6.8 Hz, 1H), 3.67 (d, J = 1.4 Hz, 1H), 3.52 (s, 3H), 2.14-2.05 (m, 1H), 2.02-1.93 (m, 1H), 1.86-1.78 (m, 1H), 1.78-1.72 (m, 1H), 1.71 (s, 3H), 1.37 (s, 3H). **^{13}C NMR** (125 MHz, acetone-d_6) δ 166.2, 156.3,

147.6, 140.1, 137.1, 135.3, 129.7, 129.5, 128.1, 127.4, 121.6, 121.4, 112.1, 109.9, 107.7, 84.0, 82.5, 80.0, 58.9, 38.3, 31.6, 27.9, 18.3; **HRMS (ESI)**: m/z calcd for $C_{26}H_{30}O_5N$ $[M+H]^+$: 436.2118; found: 436.2111.

Compound **31**: **R_f** 0.4 (2:3 petroleum ether : ethyl acetate); **$[\alpha]_D^{25}$**= -31 (c = 0.7, MeOH); **IR** (KBr) vmax (cm^{-1}) 3288, 2968, 2936, 1690, 1617, 1601, 1507, 1494, 1451, 1420, 1375, 1343, 1318, 1293, 1223, 1188, 1180, 1101, 1081, 1029, 977, 902, 808, 699; **^{1}H NMR** (500 MHz, acetone-*d*$_6$) δ 9.58 (s, 1H), 9.34 (s, 1H), 7.42 (d, J = 8.3 Hz, 1H), 7.35 (m, 5H), 6.87 (d, J = 16.2 Hz, 1H), 6.57 (d, J = 8.3 Hz, 1H), 6.35 (s, 1H), 6.34 (d, J = 16.2 Hz, 1H), 5.05 (s, 1H), 4.74 (s, 1H), 4.42 (t, J = 7.2 Hz, 1H), 3.66 (d, J = 1.4 Hz, 1H), 3.51 (s, 3H), 2.11-2.06 (m, 1H), 2.02-1.97 (m, 1H), 1.91-1.86 (m, 1H), 1.77-1.70 (m, 1H), 1.72 (s, 3H), 1.35 (s, 3H). **^{13}C NMR** (125 MHz, acetone-*d*$_6$) δ 166.2, 156.3, 147.2, 140.1, 137.1, 136.0, 129.7, 129.5, 127.9, 127.4, 121.6, 112.1, 110.2, 107.7, 85.7, 83.6, 82.7, 80.0, 58.9, 39.2, 31.9, 27.4, 18.6; **HRMS (ESI)**: m/z calcd for $C_{26}H_{30}O_5N$ $[M+H]^+$: 436.2118; found: 436.2111.

Preparation of aflaquinolones A, C and D (4, 5 and 6):

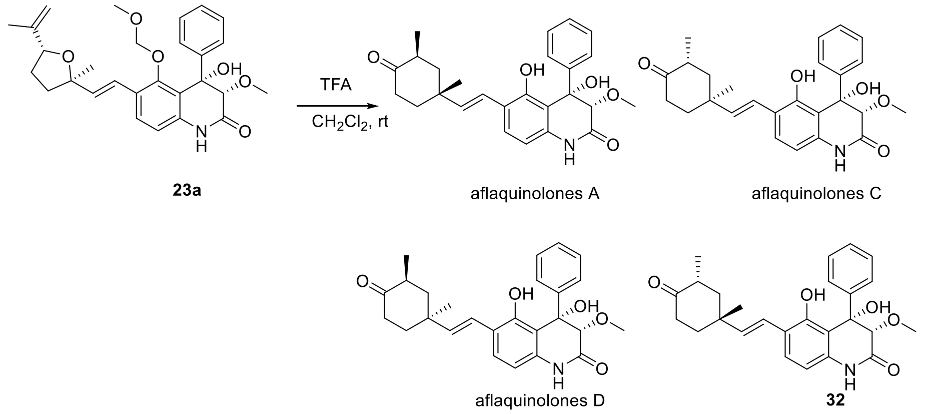

To a stirred solution of compound **23a** (100 mg) in CH_2Cl_2 (10 mL) was added concentrated TFA (0.3 mL) at room temperature, the mixture was stirred for 8 h. The solution was neutralized with $NaHCO_3$ and the aqueous layer was extracted with EtOAc (3×50 mL). The combined organic layers were washed with brine (50 mL), dried over anhydrous Na_2SO_4, filtered and concentrated in vacuo. The residue was purified by flash chromatography on silica gel (eluent: EtOAc/PE, 1/3 to 1/1) to give aflaquinolones A, C, D and 19,21-*epi*-aflaquinolone D (**32**) (64.0 mg, yield: 70%) as a white solid.

Aflaquinolone A: **R_f** 0.4 (2:3 petroleum ether : ethyl acetate); **$[\alpha]_D^{25}$**= +24 (c = 0.3, MeOH); **^{1}H NMR** (400 MHz, $CDCl_3$) δ**^{13}C NMR** (100 MHz, $CDCl_3$) δ 213.7, 165.0, 154.9, 137.1, 135.8, 134.2, 129.2, 128.8, 127.2, 126.2, 122.4, 122.2, 110.7, 106.7, 84.0, 78.8, 58.8, 47.4, 41.2, 38.5, 38.3, 37.2, 30.4, 14.3. **HRMS (ESI)**: m/z calcd for $C_{26}H_{30}O_5N$ $[M+H]^+$: 436.2118; found: 436.2113.

Aflaquinolone C: **R_f** 0.4 (2:3 petroleum ether : ethyl acetate); **$[\alpha]_D^{25}$**= +11 (c = 0.2, MeOH); **^{1}H NMR** (400 MHz, $CDCl_3$) δ 9.10 (s, 1H), 7.41 (d, J = 8.3 Hz, 1H), 7.30 (m, 5H), 6.77 (d, J = 16.6 Hz, 1H), 6.35 (d, J = 8.2 Hz, 1H), 6.27 (d, J = 16.6 Hz, 1H), 4.61 (s, 1H), 3.68 (d, J = 1.4 Hz, 1H), 3.60 (s, 3H), 2.53 (m, 1H), 2.49 (m, 1H), 2.24 (m, 1H), 2.12 (m, 2H), 1.71 (td, J = 13.9, 4.3 Hz, 1H), 1.46 (t, J = 13.5 Hz, 1H), 1.09 (s, 3H), 0.97 (d, J = 6.5 Hz, 3H). **^{13}C NMR** (100 MHz, $CDCl_3$) δ 213.7, 164.9, 154.8, 137.1, 135.8, 134.2, 129.2, 128.8, 127.2, 126.2, 122. 3, 122.2, 110.7, 106.7, 84.0, 78.8, 58.8, 47.4, 41.2, 38.4, 38.2, 37. 2, 30.4, 14.3. **HRMS (ESI)**: m/z calcd for $C_{26}H_{30}O_5N$ $[M+H]^+$: 436.2118; found: 436.2113.

Aflaquinolone D: **R_f** 0.4 (2:3 petroleum ether : ethyl acetate); **^{1}H NMR** (400 MHz, $CDCl_3$) δ 9.09 (s, 1H), 7.36 (d, J = 8.3 Hz, 1H), 7.30 (m, 5H), 6.62 (d, J = 16.4 Hz, 1H), 6.34 (d, J = 8.3 Hz, 1H), 6.14 (d, J = 16.4 Hz, 1H), 4.61 (s, 1H), 3.69 (d, J = 1.5 Hz, 1H), 3.61 (s, 3H), 2.61 (m, 1H), 2.52 (m, 1H), 2.34 (m, 1H), 1.86 (m, 3H), 1.59 (overlapped, 1H), 1.40 (s, 3H), 1.02 (d, J = 6.5 Hz, 3H). **^{1}H NMR** (400 MHz, acetone-d_6)δ 9.58 (s, 1H), 9.33 (s, 1H), 7.43 (d, J = 8.3 Hz, 1H), 7.34 (m, 5H), 6.64 (d, J = 16.5 Hz, 1H), 6.57 (d, J = 8.3 Hz, 1H), 6.36 (s, 1H), 6.24 (d, J = 16.5 Hz, 1H), 3.67 (d, J = 1.4 Hz, 1H), 3.51 (s, 3H), 2.69 (m, 1H), 2.61 (m, 1H), 2.20 (m, 1H), 1.86 (m, 3H), 1.54 (t, J = 13.2 Hz, 1H), 1.43 (s, 3H), 0.95 (d, J = 6.5 Hz, 3H). **^{13}C NMR** (100 MHz, $CDCl_3$) δ 213.5, 165.2, 155.1, 139.8, 137.4, 134.3, 129.4, 129.1, 127.4, 126.4, 122.5, 119.8, 110.9, 106.9, 84.2, 79.1, 59.1, 46.9, 40.8, 38.2, 38.0, 36.4, 22.6, 14.7. **HRMS (ESI)**: m/z calcd for $C_{26}H_{30}O_5N$ $[M+H]^+$: 436.2118; found: 436.2113.

19,21-*epi*-aflaquinolone D (**32**): **R_f** 0.4 (2:3 petroleum ether : ethyl acetate); **^{1}H NMR** (500 MHz, $CDCl_3$) δ 9.09 (s, 1H), 7.36 (d, J = 8.3 Hz, 1H), 7.30 (m, 5H), 6.62 (d, J = 16.4 Hz, 1H), 6.34 (d, J = 8.3 Hz, 1H), 6.14 (d, J = 16.4 Hz, 1H), 4.61 (s, 1H), 3.69 (d, J = 1.4 Hz, 1H), 3.61 (s, 3H), 2.61 (m, 1H), 2.52 (m, 1H), 2.34 (m, 1H), 1.86 (m, 3H), 1.59 (overlapped, 1H), 1.40 (s, 3H), 1.02 (d, J = 6.5 Hz, 3H). **^{13}C NMR** (125 MHz, $CDCl_3$) δ 213.5, 165.2, 155.1, 139.7, 137.4, 134.3, 129.4, 129.1, 127.4, 126.4, 122.5, 119.8, 110.9, 106.9, 84.2, 79.0, 59.1, 46.9, 40.8, 38.2, 38.0, 36.4, 22.6, 14.7. **HRMS (ESI)**: m/z calcd for $C_{26}H_{30}O_5N$ $[M+H]^+$: 436.2118; found: 436.2113.

Supplementary Tables

1. Comparison of NMR Spectra of Isolated and Synthetic Compounds

Supplementary Table 1. ^{1}H NMR comparison of isolated (+)-Aniduquinolone A[2,3,4] and synthetic (+)-Aniduquinolone A (**1**).

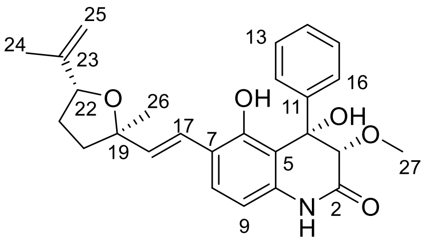

(+)-Aniduquinolone A (1)

^{1}H NMR Data	Synthetic (600 MHz) [a]	Synthetic (500 MHz) [b]	Synthetic (400 MHz) [c]	Natural (500 MHz) [a]	Natural (600 MHz) [c]
Position			δ_H (J in Hz)		
1-NH	10.30, br s	9.34, br s		10.29, br s	
2					
3	3.60, d (1.3)	3.67, d (1.3)	3.64, s	3.60, s	3.61, s
4					
5					
6					
7					
8	7.37, d (8.3)	7.43, d (8.3)	7.38, d (8.3)	7.35, m	7.38, d (8.3)
9	6.44, d (8.3)	6.58, d (8.3)	6.45, d (8.3)	6.44, d (8.3)	6.45, d (8.3)
10					
11					
12/16	7.20, m	7.35, m	7.28, m	7.22, d (7.6)	7.28, m
13/15	7.34, m	7.35, m	7.31, m	7.35, m	7.32, m
14	7.34, m	7.35, m	7.31, m	7.35, m	7.32, m
17	6.65, d (16.2)	6.81, d (16.2)	6.80, d (16.2)	6.67, d (16.2)	6.80, d (16.2)
18	6.24, d (16.2)	6.27, d (16.2)	6.24, d (16.2)	6.24, d (16.2)	6.24, d (16.2)
19					
20	1.92, m	1.98, m	2.03, m	1.92, m	1.92, m
	1.75, m	1.81, m	1.85, m	1.76, m	1.83, m
21	1.70, m	2.07, m	1.81, m	1.72, m	1.83, m
	2.01, m	1.75, m	2.11, m	2.01, m	2.04, m
22	4.33, t (6.7)	4.40, t (6.7)	4.49, t (6.7)	4.33, t (6.7)	4.49, t (6.9)
23					
24	1.67, s	1.70, s	1.74, s	1.67, s	1.73, s
25	4.75, s	4.99, s	4.80, s	4.75, s	4.82, s
	4.96, s	4.74, s	5.02, s	4.96, s	5.01, s
26	1.33, s	1.37, s	1.43, s	1.34, s	1.43, s
27	3.44, s	3.52, s	3.55, s	3.45, s	3.55, s

[a] Measured in DMSO-d_6. [b] Measured in acetone-d_6. [c] Measured in methanol-d_4

Supplementary Table 2. ^{13}C NMR comparison of isolated (+)-Aniduquinolone A[2,3,4] and synthetic (+)-Aniduquinolone A (**1**).

^{13}C NMR Data	Synthetic (150 MHz) [a]	Synthetic (125 MHz) [b]	Synthetic (100 MHz) [c]	Natural (125 MHz) [a]	Natural (150 MHz) [c]
Position	δ_C				
1					
2	166.1	166.2	168.7	166.1	168.7
3	84.2	85.7	86.1	84.2	86.0
4	78.6	80.0	79.9	78.6	79.9
5	111.1	112.1	112.5	111.1	112.2
6	154.7	156.3	156.4	155.0	156.2
7	119.4	121.5	122.2	119.5	122.2
8	126.8	128.0	127.9	126.7	127.9
9	106.9	107.7	107.9	106.6	107.8
10	136.0	137.1	136.8	136.0	136.8
11	139.8	140.2	140.2	140.0	140.3
12/16	126.2	129.5	127.4	126.1	127.1
13/15	128.6	127.4	129.5	128.5	129.6
14	128.6	129.7	129.7	128.5	129.6
17	120.1	121.5	122.0	120.3	122.2
18	134.2	135.3	134.7	134.0	134.4
19	82.9	84.0	84.9	82.9	84.8
20	37.3	38.3	38.3	37.2	38.7
21	30.3	31.6	31.4	30.3	31.6
22	81.2	82.5	83.4	81.1	83.4
23	146.2	147.6	147.1	146.2	147.0
24	18.0	18.3	17.9	17.9	17.9
25	109.7	109.9	110.7	109.6	110.7
26	27.4	27.9	27.3	27.3	27.2
27	58.4	58.9	59.0	58.3	58.8

[a] Measured in DMSO-d_6. [b] Measured in acetone-d_6. [c] Measured in methanol-d_4

Supplementary Table 3. ^{1}H NMR ($CDCl_3$) and ^{13}C NMR ($CDCl_3$) comparison of isolated Aflaquinolone A[4,5] and synthetic Aflaquinolone A.

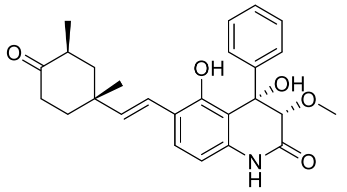

Aflaquinolone A

^{1}H NMR Data	Synthetic (400 MHz)	Natural (600 MHz)	^{13}C NMR Data	Synthetic (100 MHz)	Natural (150 MHz)
Position	δ_H (J in Hz)		Position	δ_C	
1-NH	7.52, br s	7.52, br s	1		
2			2	165.0	165.1
3	3.68, d (1.5)	3.67, d (1.5)	3	84.0	84.0
4			4	78.8	79.0
5			5	110.7	110.9
6			6	154.9	155.0
7			7	122.2	122.3
8	7.40, d (8.3)	7.40, d (8.3)	8	127.2	127.2
9	6.36, d (8.3)	6.35, d (8.3)	9	106.7	106.9
10			10	134.2	134.3
11			11	137.1	137.2
12/16	7.26-7.33, m	7.26-7.33, m	12/16	128.8	128.9
13/15	7.26-7.33, m	7.26-7.33, m	13/15	126.2	126.2
14	7.30, m	7.30, m	14	129.2	129.2
17	6.77, d (17)	6.77, d (17)	17	122.4	122.5
18	6.26, d (17)	6.26, d (17)	18	135.8	135.9
19			19	37.2	37.3
20	2.09, m<br>1.45, t (13)	2.09, m<br>1.45, t (13)	20	47.4	47.5
21	2.53, dpent (13, 6.0)	2.53, dpent (13, 6.0)	21	41.2	41.3
22			22	213.7	213.7
23	2.47, ddd (14, 14, 5.8)	2.47, ddd (14, 14, 5.8)	23	38.5	38.6
23	2.22, ddd (14, 4.5 2.5)	2.22, ddd (14, 4.5 2.5)	23		
24	2.13, m	2.13, m	24	38.3	38.4
24	1.71, td (14, 4.5)	1.71, td (14, 4.5)	24		
25	1.09. s	1.09, s	25	30.4	30.4
26	0.97, d (6.5)	0.96, d (6.4)	26	14.3	14.4
27	3.60, s	3.60, s	27	58.8	58.9

Supplementary Table 4. ^{1}H NMR ($CDCl_3$) and ^{13}C NMR ($CDCl_3$) comparison of isolated Aflaquinolone C[4,5] and synthetic Aflaquinolone C.

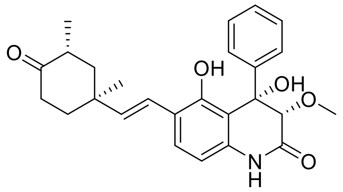

Aflaquinolone C

^{1}H NMR Data	Synthetic (400 MHz)	Natural (600 MHz)	^{13}C NMR Data	Synthetic (100 MHz)	Natural (150 MHz)
Position	δ_H (J in Hz)		Position	δ_C	
1-NH	9.10, br s	7.52, br s	1		
2			2	164.9	165.1
3	3.68, d (1.5)	3.67, d (1.5)	3	84.0	84.0
4			4	78.8	79.0
5			5	110.7	110.9
6			6	154.8	155.0
7			7	122.2	122.3
8	7.41, d (8.3)	7.40, d (8.3)	8	127.2	127.2
9	6.35, d (8.3)	6.35, d (8.3)	9	106.7	106.9
10			10	134.2	134.3
11			11	137.1	137.2
12/16	7.26-7.33, m	7.26-7.33, m	12/16	128.8	128.9
13/15	7.26-7.33, m	7.26-7.33, m	13/15	126.2	126.2
14	7.30, m	7.30, m	14	129.2	129.2
17	6.77, d (17)	6.77, d (17)	17	122.3	122.5
18	6.27, d (17)	6.26, d (17)	18	135.8	135.9
19			19	37.2	37.3
20	2.12, m	2.09, m	20	47.4	47.5
	1.46, t (13)	1.45, t (13)			
21	2.53, dpent (13, 6.0)	2.53, dpent (13, 6.0)	21	41.2	41.3
22			22	213.7	213.7
23	2.49, ddd (14, 14, 5.8)	2.47, ddd (14, 14, 5.8)	23	38.4	38.6
23	2.24, ddd (14, 4.5 2.5)	2.22, ddd (14, 4.5 2.5)	23		
24	2.12, m	2.13, m	24	38.2	38.4
24	1.71, td (14, 4.5)	1.71, td (14, 4.5)	24		
25	1.09, s	1.09, s	25	30.4	30.4
26	0.97, d (6.5)	0.96, d (6.4)	26	14.3	14.4
27	3.60, s	3.60, s	27	58.8	58.9

Supplementary Figures

1. X-ray Crystallographic Data

The crystal structure and X-ray crystallographic data of compound **14** (CCDC **2122875**) was described as follows:

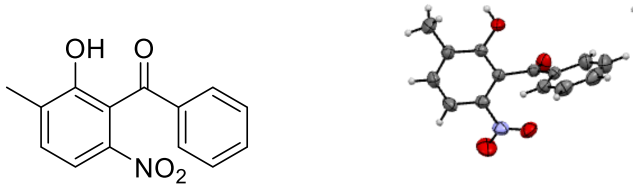

Supplementary Figure 1. X-ray crystal structure of **14**

Crystal data and structure refinement for 14

Identification code	11368	
Empirical formula	$C_{14}H_{11}N_1O_4$	
Formula weight	514.47	
Temperature	293(2) K	
Wavelength	1.54184 A	
Crystal system, space group	Monoclinic, C2/c	
Unit cell dimensions	a = 25.2201(4) A	alpha = 90 deg.
	b = 8.11224(16) A	beta = 91.0221(16) deg.
	c = 25.2396(4) A	gamma = 90 deg.
Volume	5163.00(16) A^3	
Z, Calculated density	8, 1.324 Mg/m^3	
Absorption coefficient	0.823 mm^-1	
F(000)	2144	
Crystal size	0.120 x 0.110 x 0.110 mm	
Theta range for data collection	3.503 to 67.237 deg.	
Limiting indices	-30<=h<=29, -9<=k<=9, -20<=l<=30	
Reflections collected / unique	16921 / 4623 [R(int) = 0.0254]	
Completeness to theta = 67.237	99.9 %	
Refinement method	Full-matrix least-squares on F^2	
Data / restraints / parameters	4623 / 0 / 348	
Goodness-of-fit on F^2	1.046	
Final R indices [I>2sigma(I)]	R1 = 0.0440, wR2 = 0.1246	
R indices (all data)	R1 = 0.0513, wR2 = 0.1319	

Extinction coefficient	0.00123(8)
Largest diff. peak and hole	0.530 and -0.249 e.A^-3

The crystal structure and X-ray crystallographic data of compound **1** (CCDC **2122872**) was described as follows:

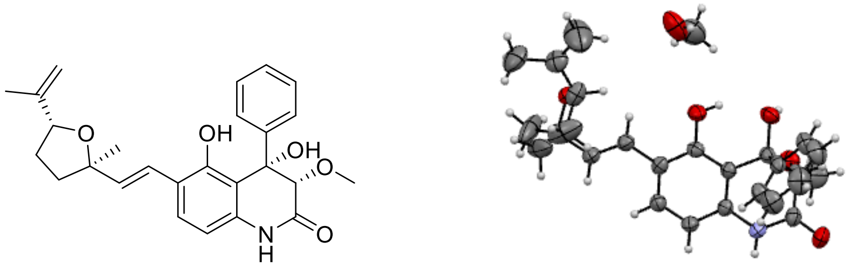

Supplementary Figure 2. X-ray crystal structure of **1**

Crystal data and structure refinement for 1.

Identification code	11646
Empirical formula	$C_{27}H_{33}NO_6$
Formula weight	467.54
Temperature	293(2) K
Wavelength	1.54178 A
Crystal system, space group	Monoclinic, P2(1)
Unit cell dimensions	a = 8.3402(3) A alpha = 90 deg.
	b = 13.8121(7) A beta = 94.737(4) deg.
	c = 11.1111(5) A gamma = 90 deg.
Volume	1275.58(10) A^3
Z, Calculated density	2, 1.217 Mg/m^3
Absorption coefficient	0.697 mm^-1
F(000)	500
Crystal size	0.160 x 0.120 x 0.110 mm
Theta range for data collection	3.992 to 67.190 deg.
Limiting indices	-9<=h<=9, -14<=k<=16, -13<=l<=12
Reflections collected / unique	4580 / 3258 [R(int) = 0.0188]
Completeness to theta = 67.190	99.6 %
Refinement method	Full-matrix least-squares on F^2
Data / restraints / parameters	3258 / 2 / 315
Goodness-of-fit on F^2	1.031
Final R indices [I>2sigma(I)]	R1 = 0.0386, wR2 = 0.0924

R indices (all data)	R1 = 0.0432, wR2 = 0.0969
Absolute structure parameter	0.19(15)
Extinction coefficient	0.0079(7)
Largest diff. peak and hole	0.167 and -0.152 e.A^-3

The crystal structure and X-ray crystallographic data of compound **29** (CCDC **2122876**) was described as follows:

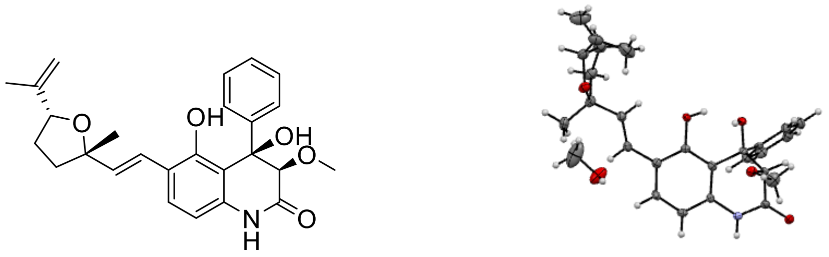

Supplementary Figure 3. X-ray crystal structure of **29**

Crystal data and structure refinement for 29

Identification code	11756
Empirical formula	$C_{27}H_{33}NO_6$
Formula weight	467.54
Temperature	293(2) K
Wavelength	1.54184 A
Crystal system, space group	Monoclinic, P2(1)
Unit cell dimensions	a = 10.5614(4) A alpha = 90 deg.
	b = 7.9969(3) A beta = 97.576(4) deg.
	c = 15.0682(7) A gamma = 90 deg.
Volume	1261.52(10) A^3
Z, Calculated density	2, 1.231 Mg/m^3
Absorption coefficient	0.705 mm^-1
F(000)	500
Crystal size	0.090 x 0.080 x 0.080 mm
Theta range for data collection	2.958 to 67.249 deg.
Limiting indices	-12<=h<=12, -6<=k<=9, -17<=l<=18
Reflections collected / unique	4471 / 3143 [R(int) = 0.0289]
Completeness to theta = 67.249	99.8 %
Refinement method	Full-matrix least-squares on F^2
Data / restraints / parameters	3143 / 1 / 314
Goodness-of-fit on F^2	1.036
Final R indices [I>2sigma(I)]	R1 = 0.0416, wR2 = 0.1027
R indices (all data)	R1 = 0.0462, wR2 = 0.1067
Absolute structure parameter	0.0(2)
Extinction coefficient	n/a
Largest diff. peak and hole	0.208 and -0.270 e.A^-3

2. Experimental ECD Spectra

Supplementary Figure 4. Experimental ECD spectra of **23** and **23a**

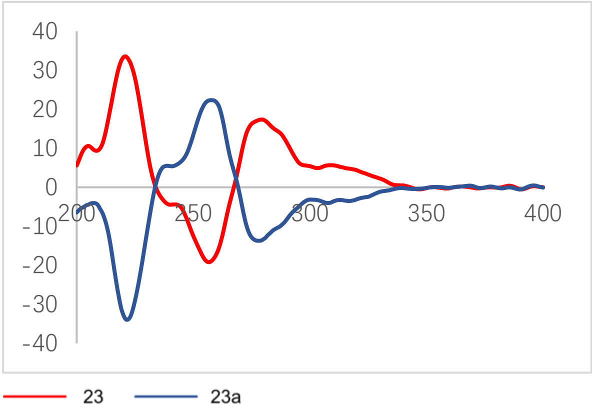

Supplementary Figure 5. Experimental ECD spectra of **24** and **24a**

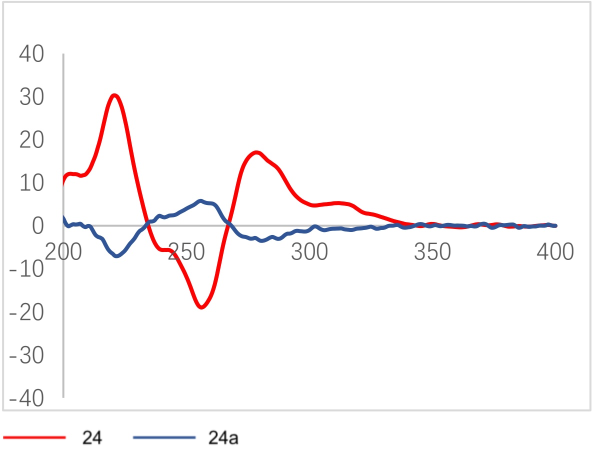

Supplementary Figure 6. Experimental ECD spectra of **1** and its stereoisomers

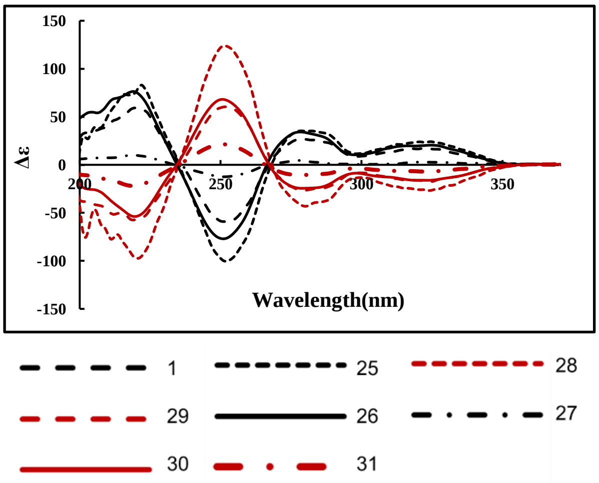

3. Comparison of the NMR Spectral Data of Compound 1 and Its Stereoisomers

Supplementary Figure 7. ^{1}H NMR spectra of compound **1** and its stereoisomers

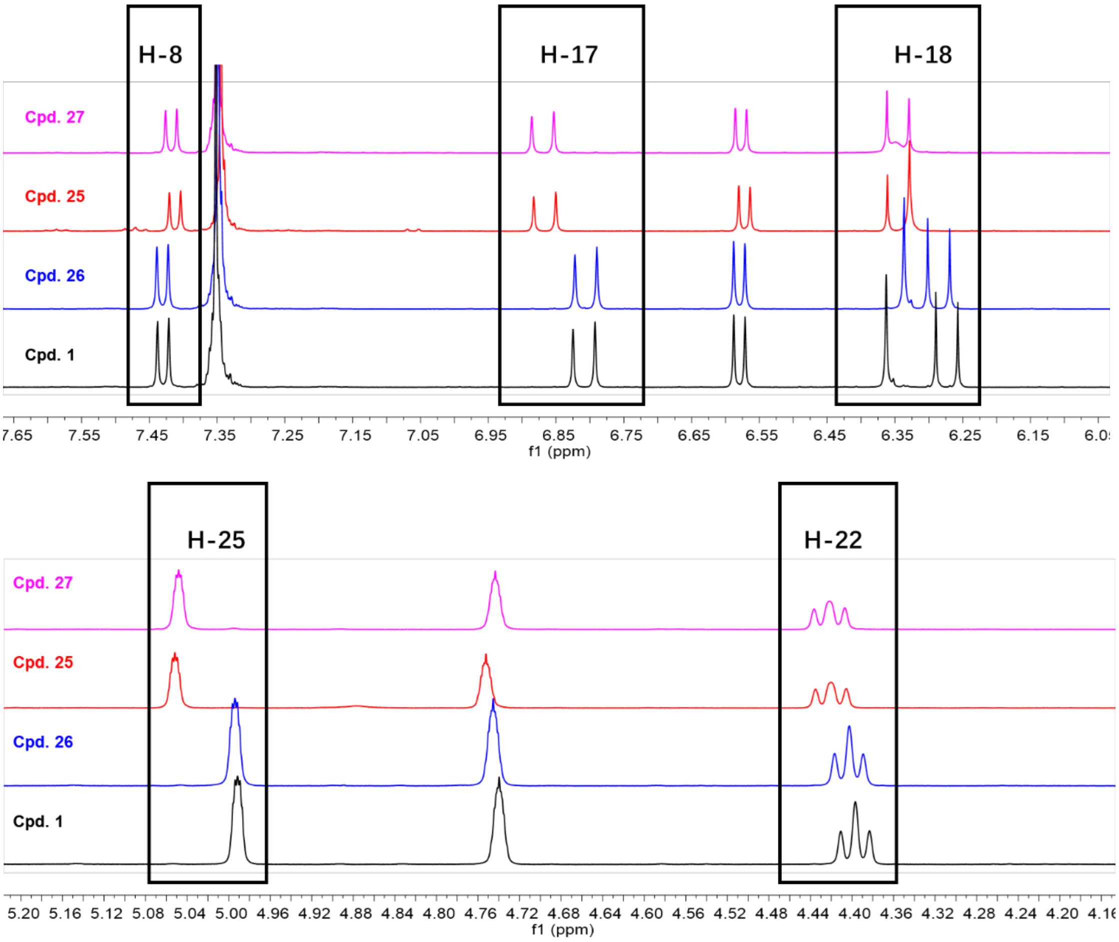

Supplementary Figure 8. ^{13}C NMR spectra of compound **1** and its stereoisomers

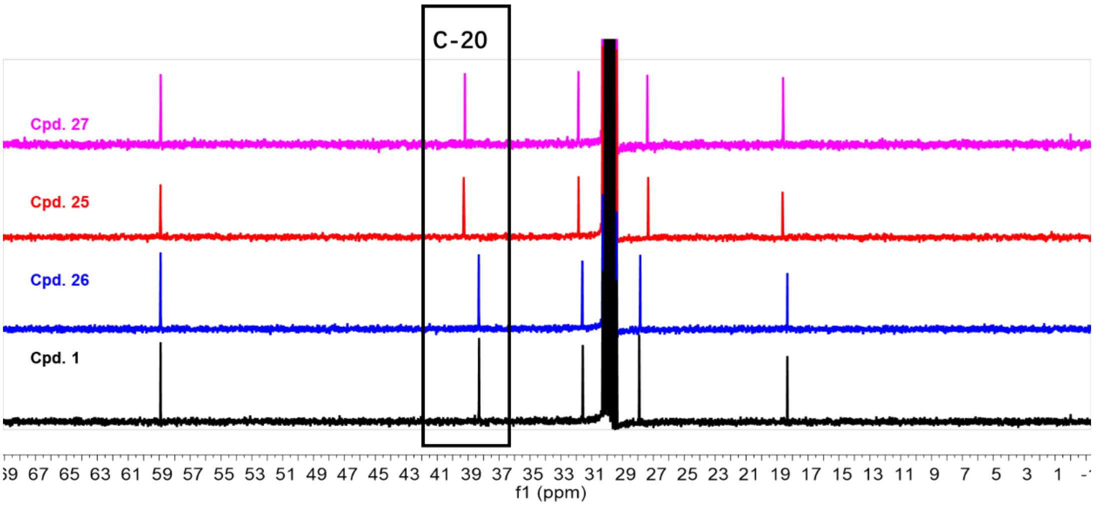

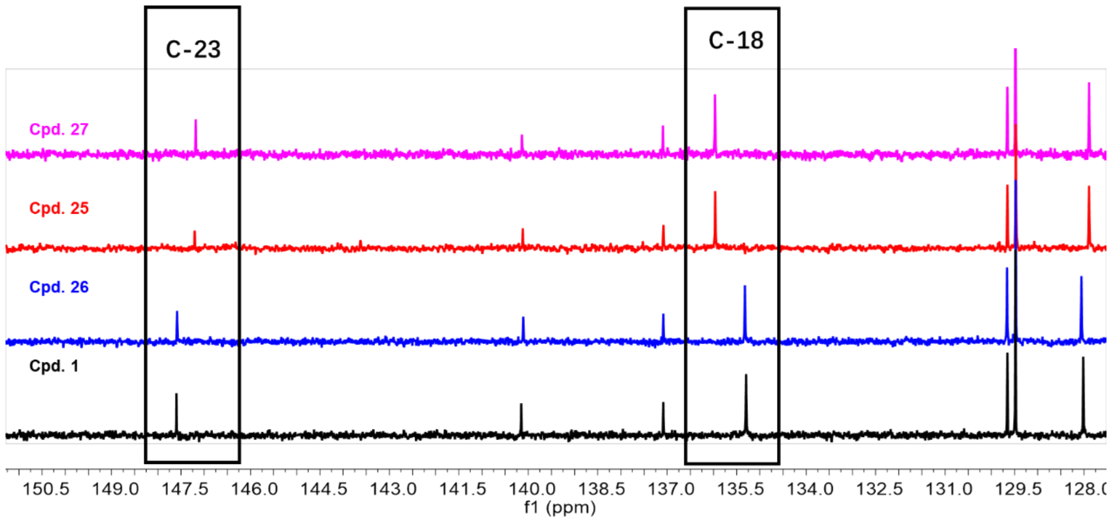

Supplementary Figure 9. The values of Δ*δ* H-20 of **1**, **25**, **26** and **27** are shown

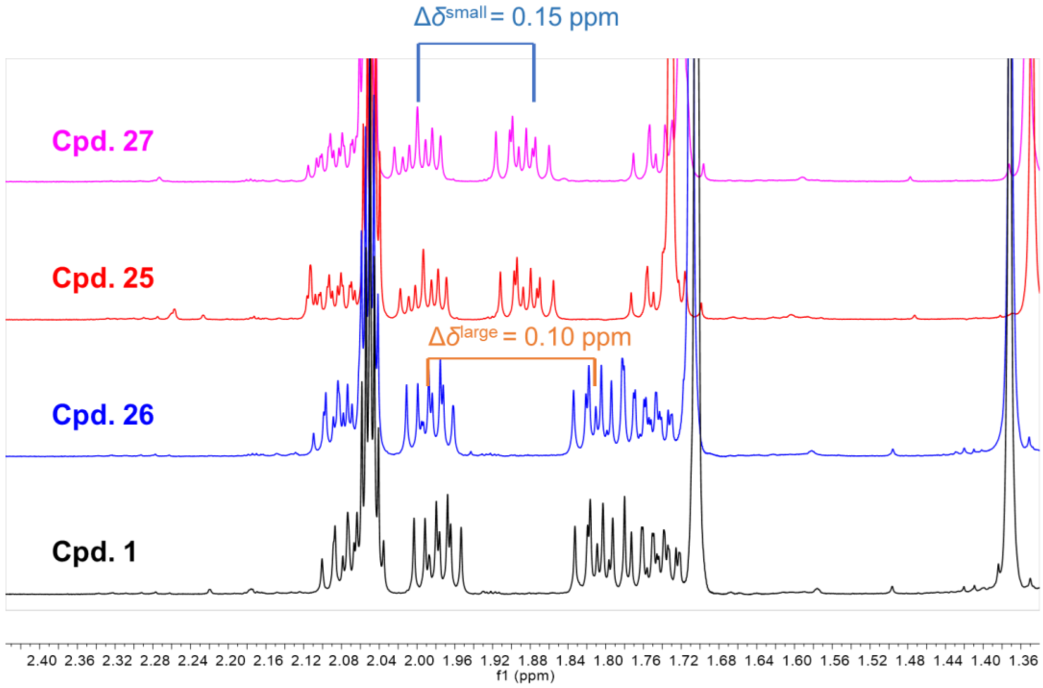

4. NMR Spectra of the Synthesized Compounds in This Article

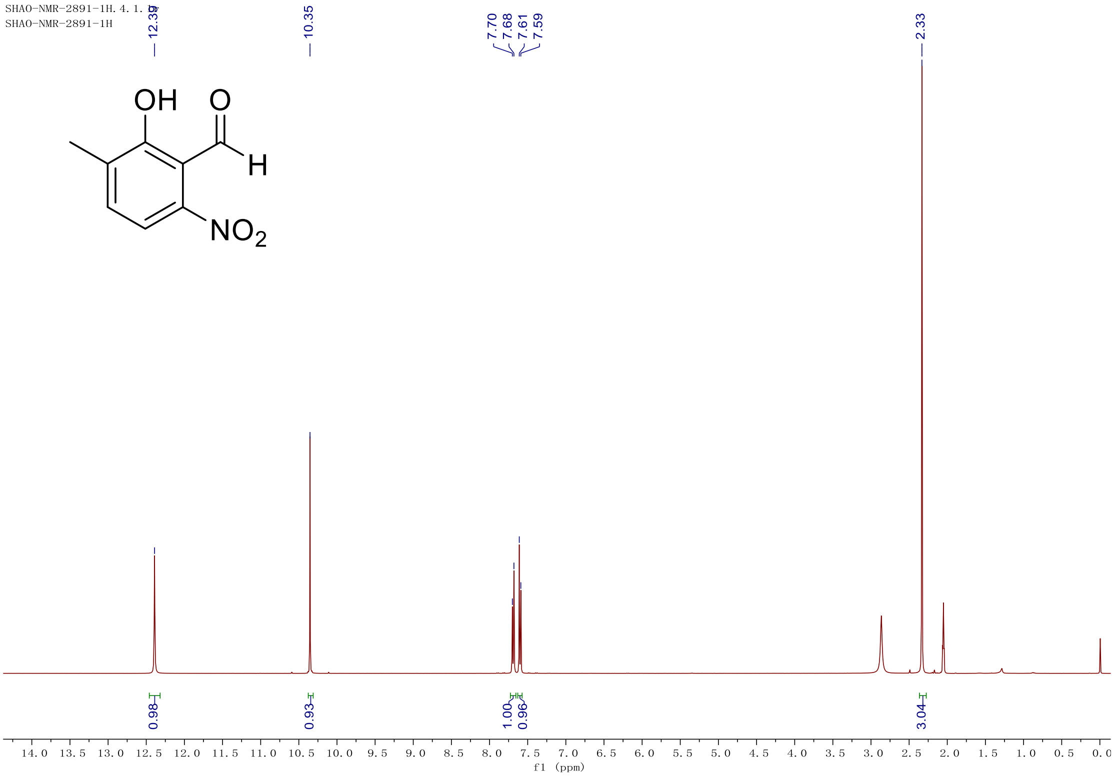

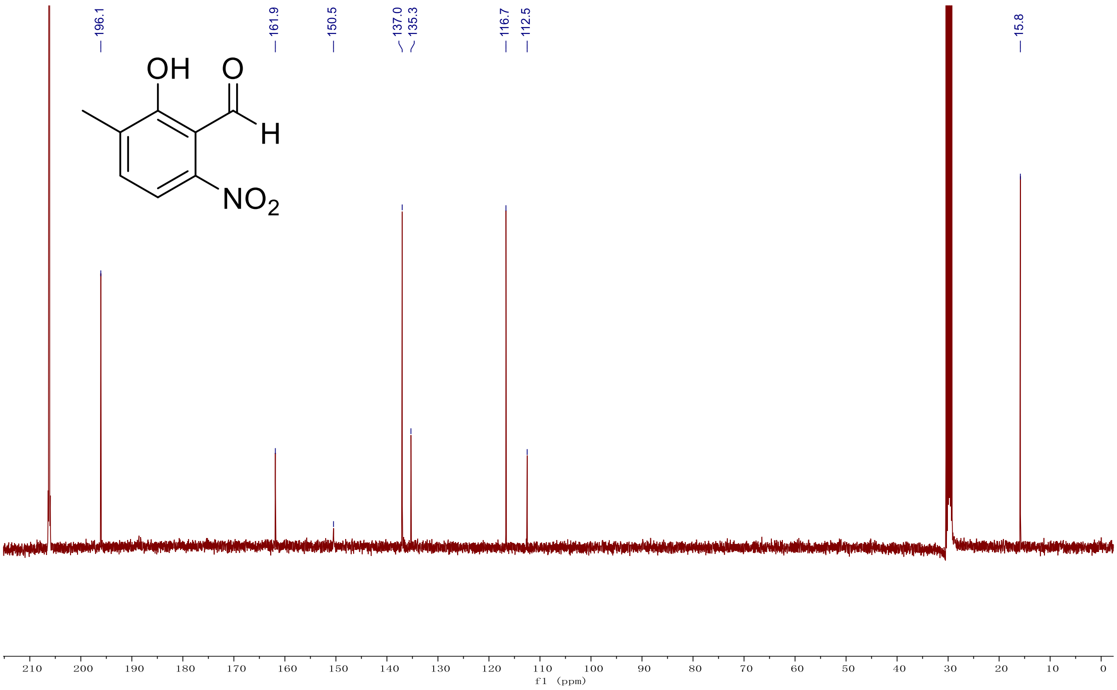

Supplementary Figure 10. ^{1}H (400 MHz, $CDCl_3$) and ^{13}C NMR (100 MHz, $CDCl_3$) spectra of compound **18**

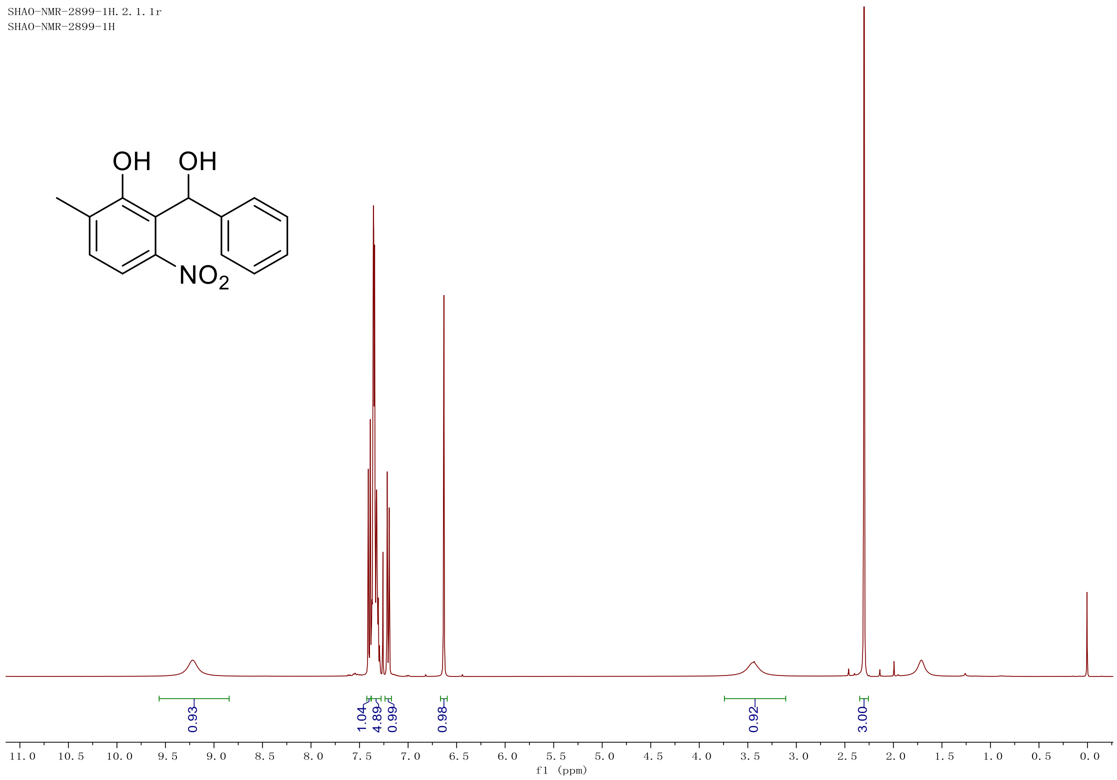

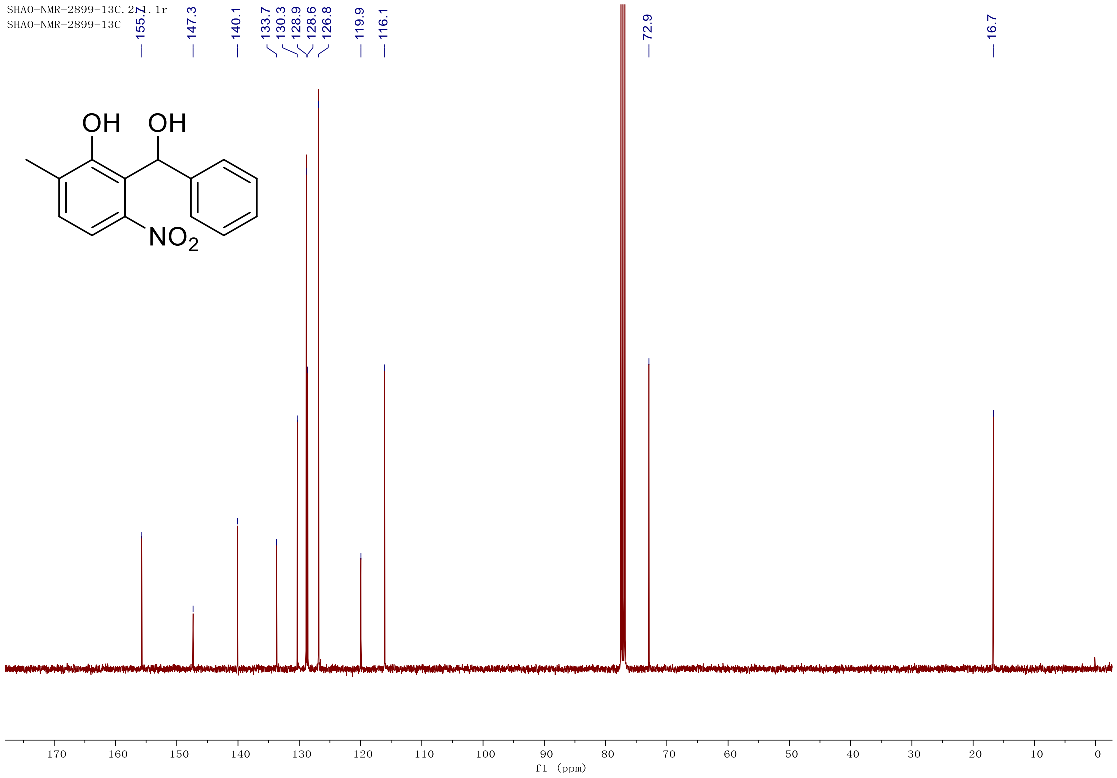

Supplementary Figure 11. ^{1}H (400 MHz, $CDCl_3$) and ^{13}C NMR (100 MHz, $CDCl_3$) spectra of compound **19**

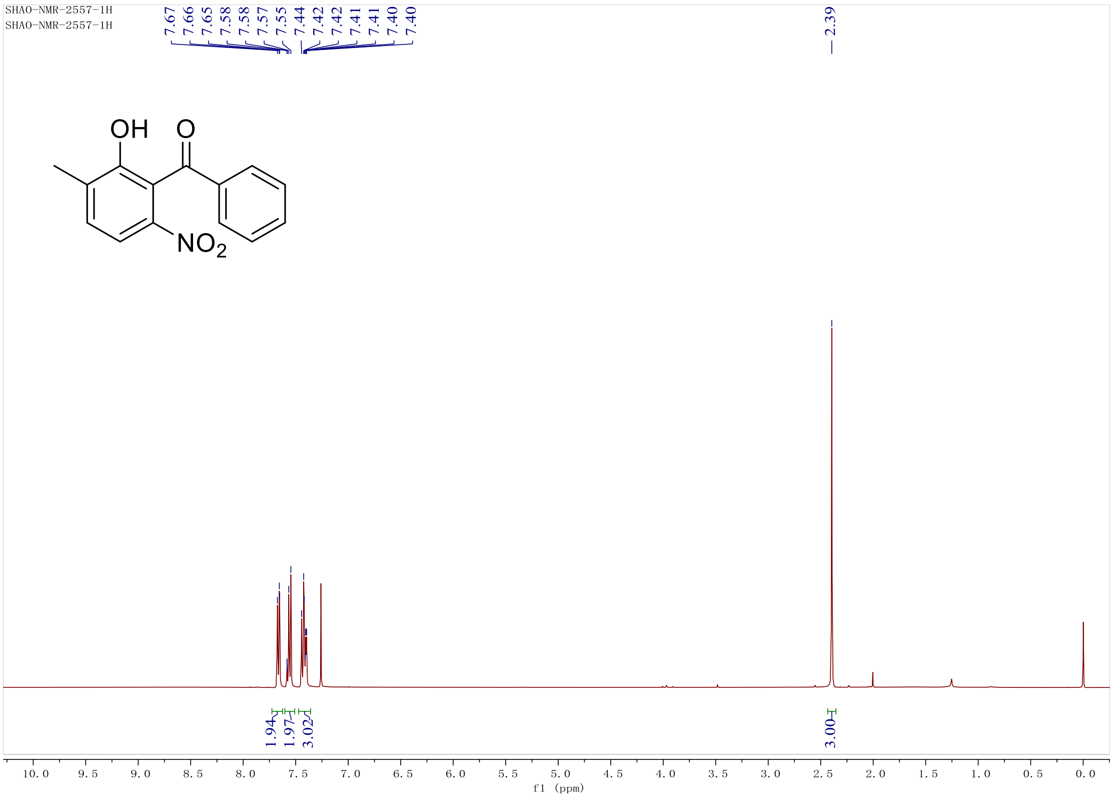

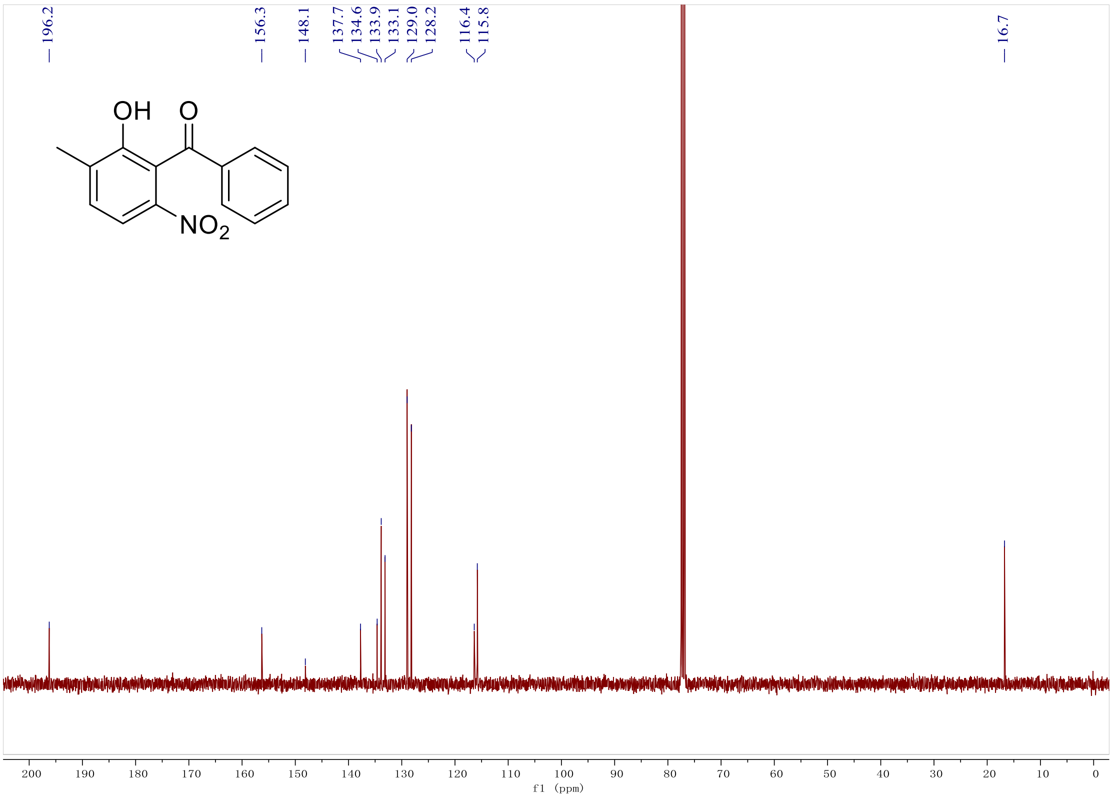

Supplementary Figure 12. ^{1}H (400 MHz, $CDCl_3$) and ^{13}C NMR (100 MHz, $CDCl_3$) spectra of compound **14**

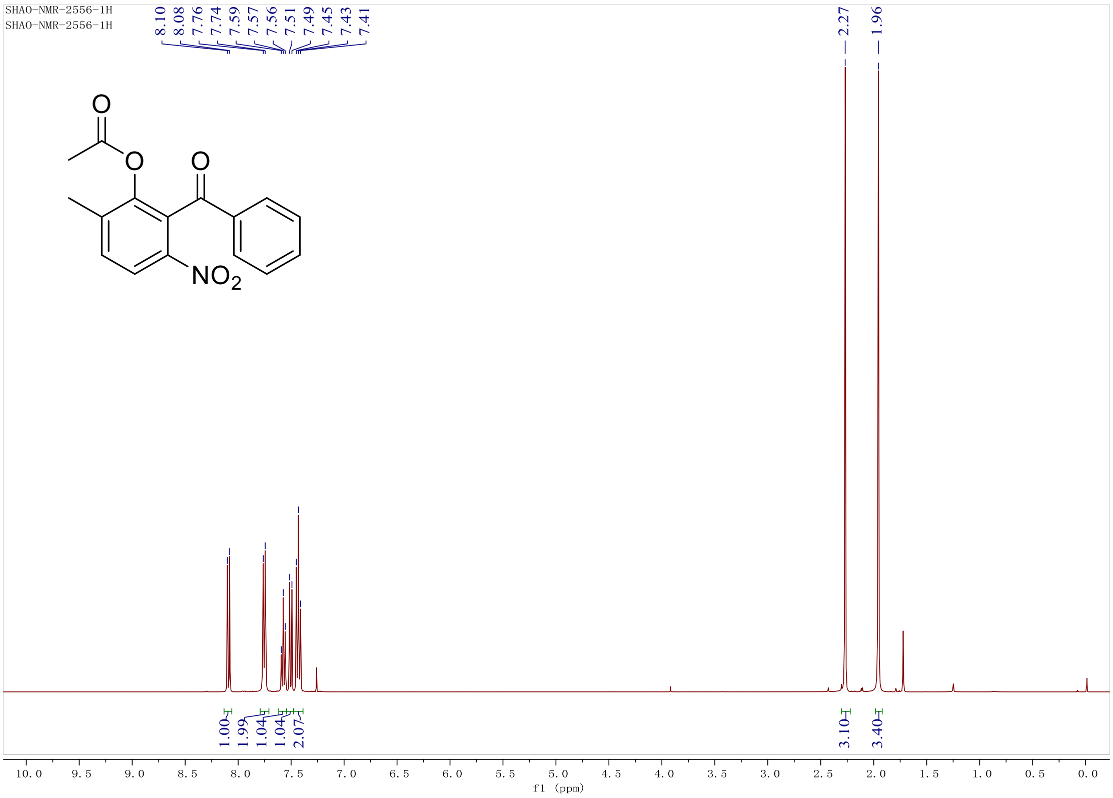

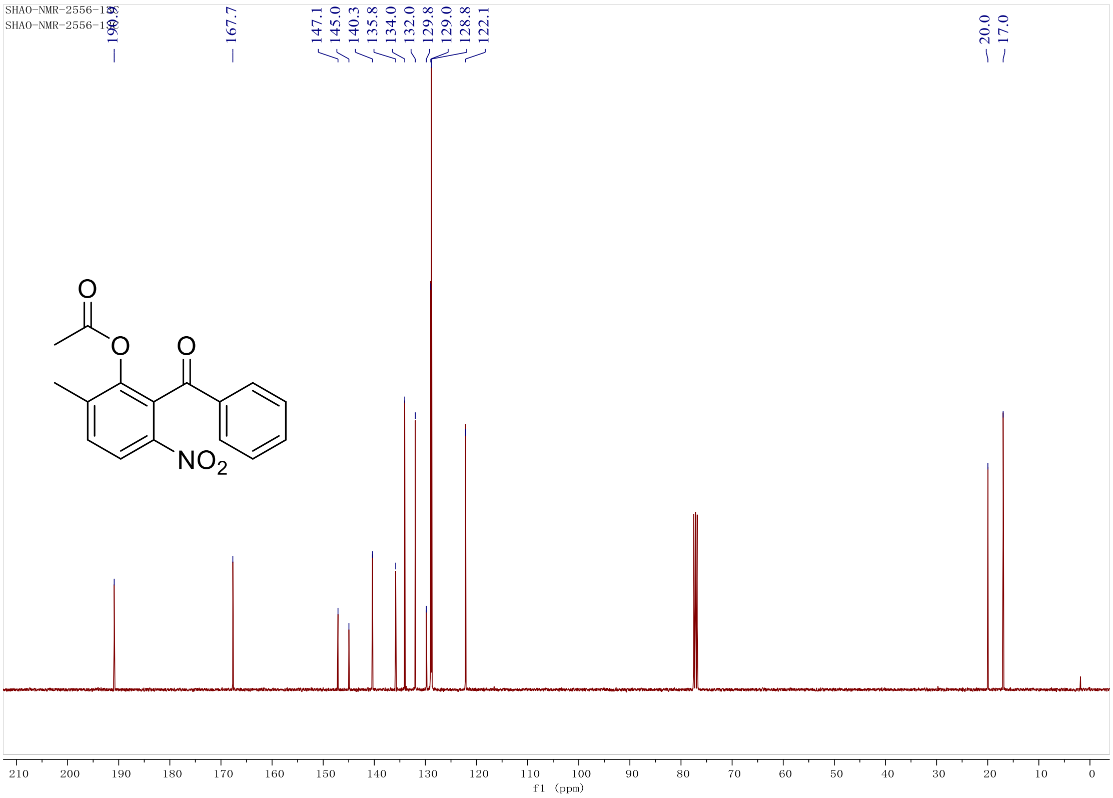

Supplementary Figure 13. ^{1}H (400 MHz, $CDCl_3$) and ^{13}C NMR (100 MHz, $CDCl_3$) spectra of compound **20**

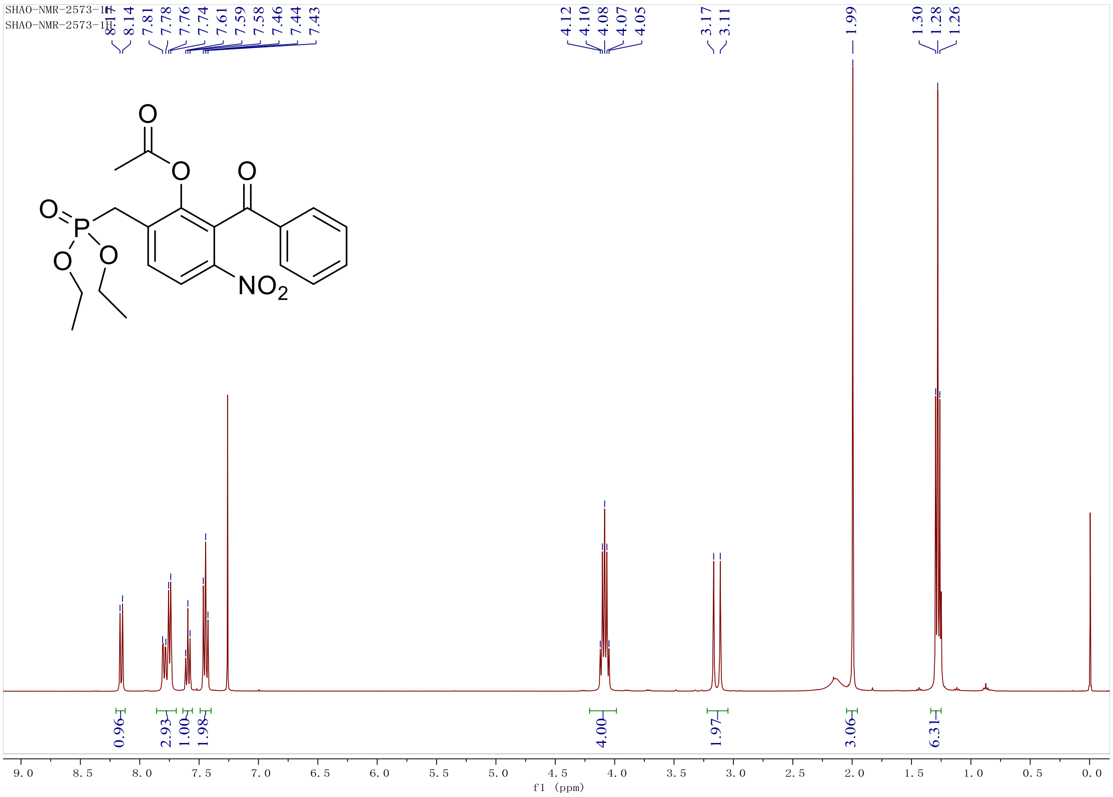

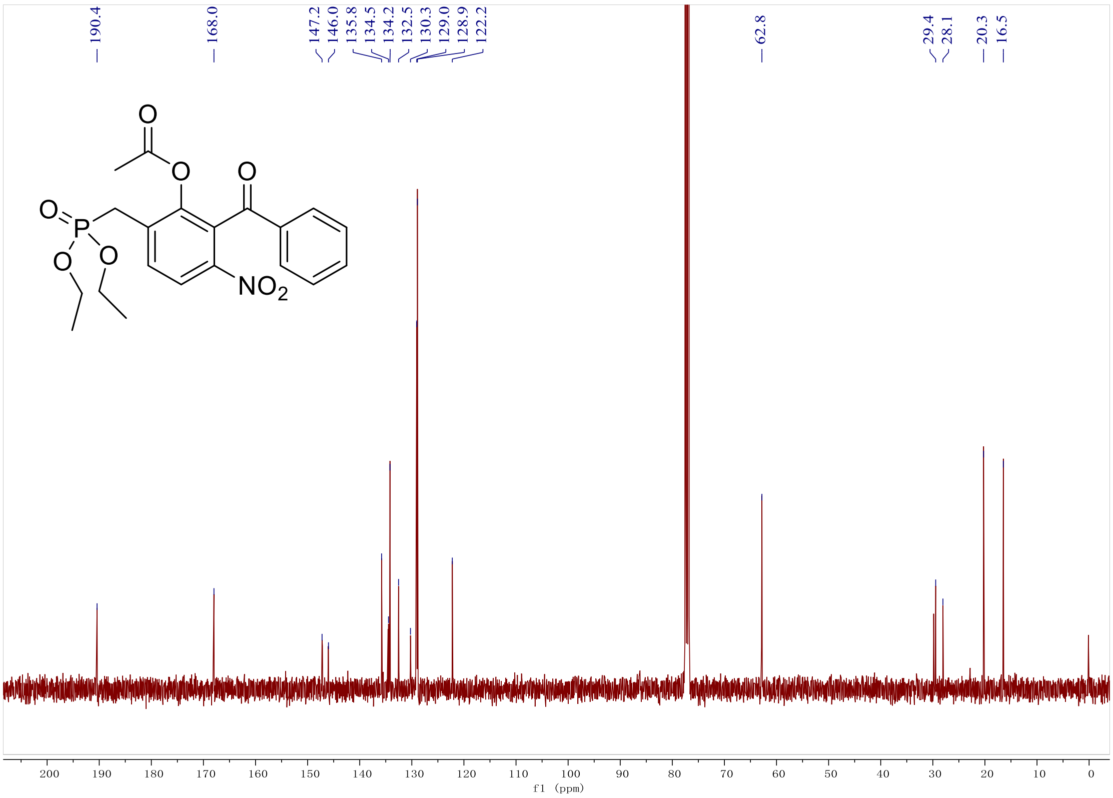

Supplementary Figure 14. ^{1}H (400 MHz, $CDCl_3$) and ^{13}C NMR (100 MHz, $CDCl_3$) spectra of compound **21**

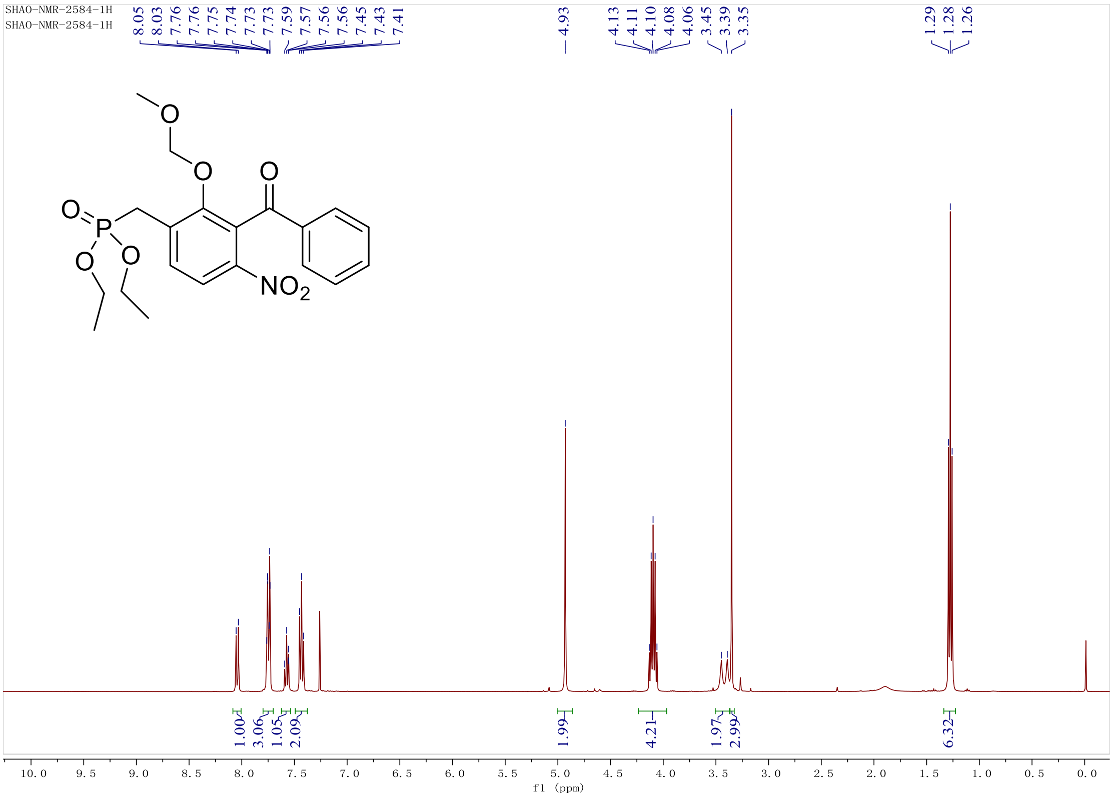

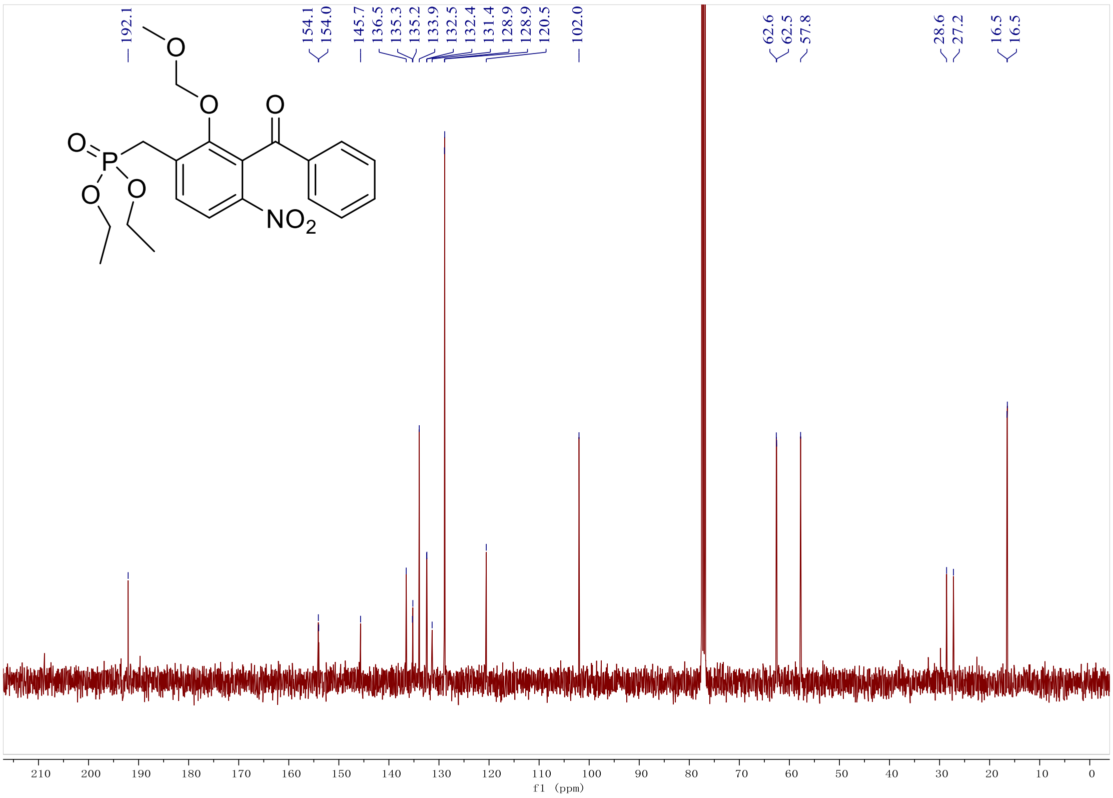

Supplementary Figure 15. ^{1}H (400 MHz, $CDCl_3$) and ^{13}C NMR (100 MHz, $CDCl_3$) spectra of compound **12**

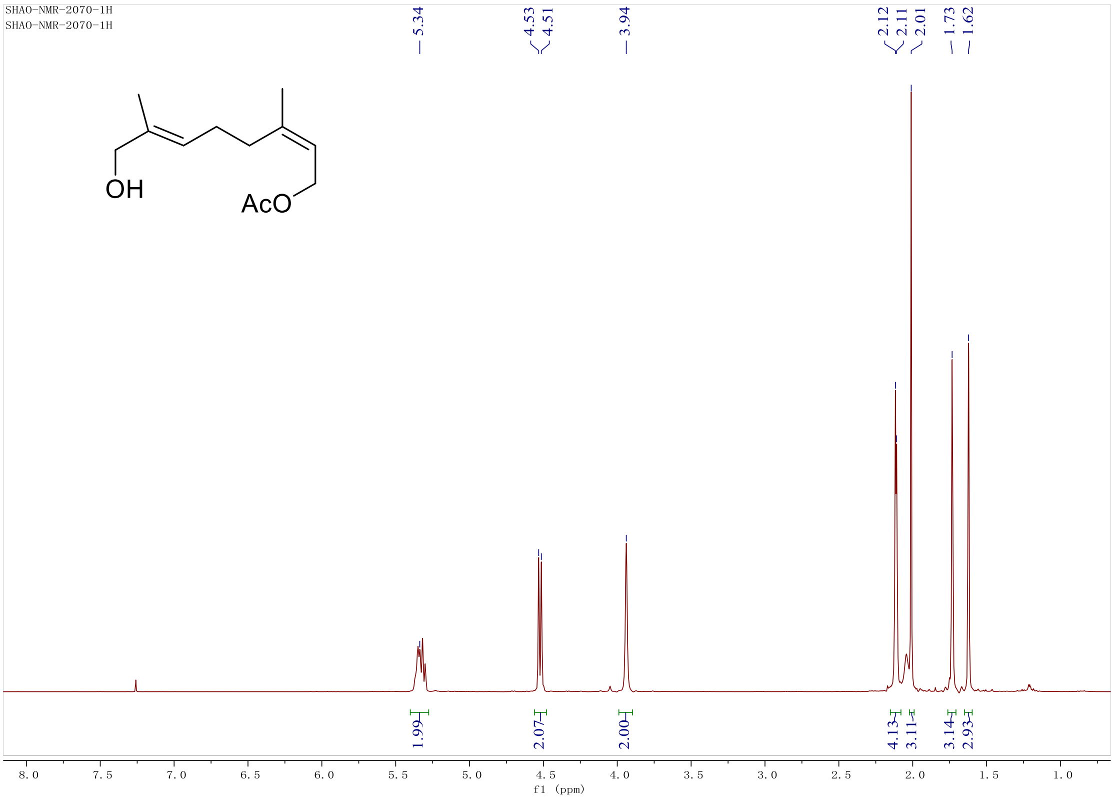

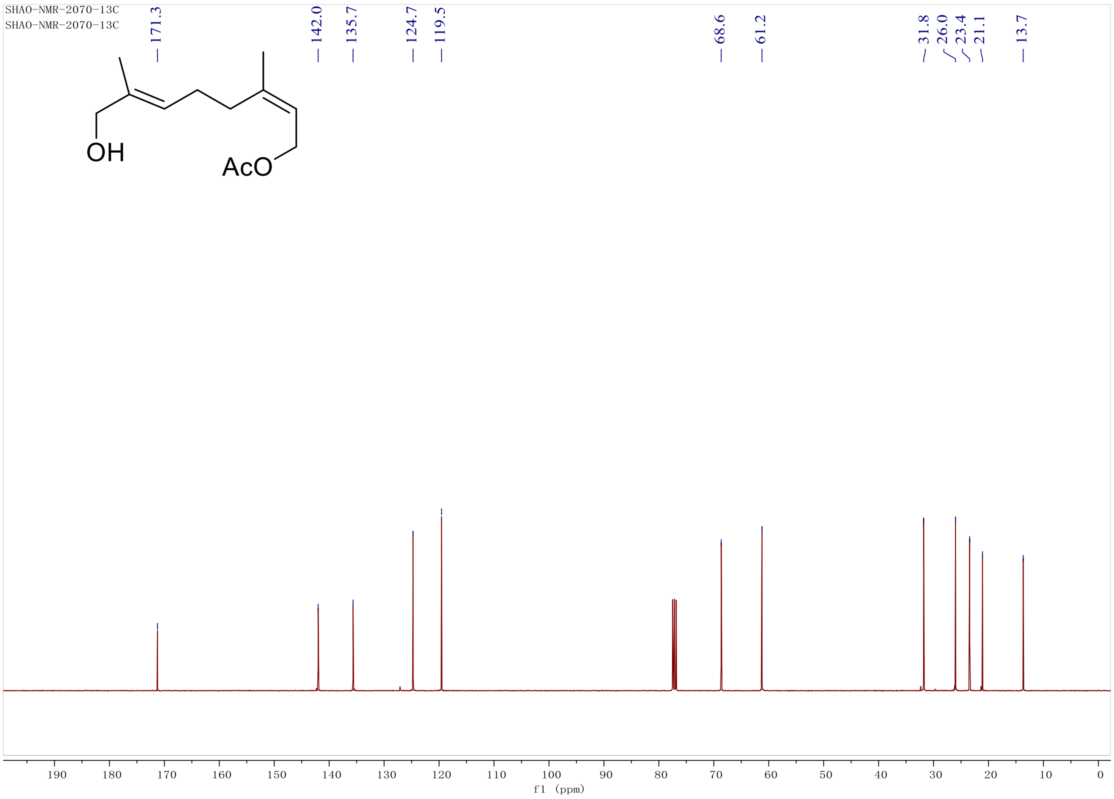

Supplementary Figure 16. ^{1}H (400 MHz, $CDCl_3$) and ^{13}C NMR (100 MHz, $CDCl_3$) spectra of compound **SI-4**

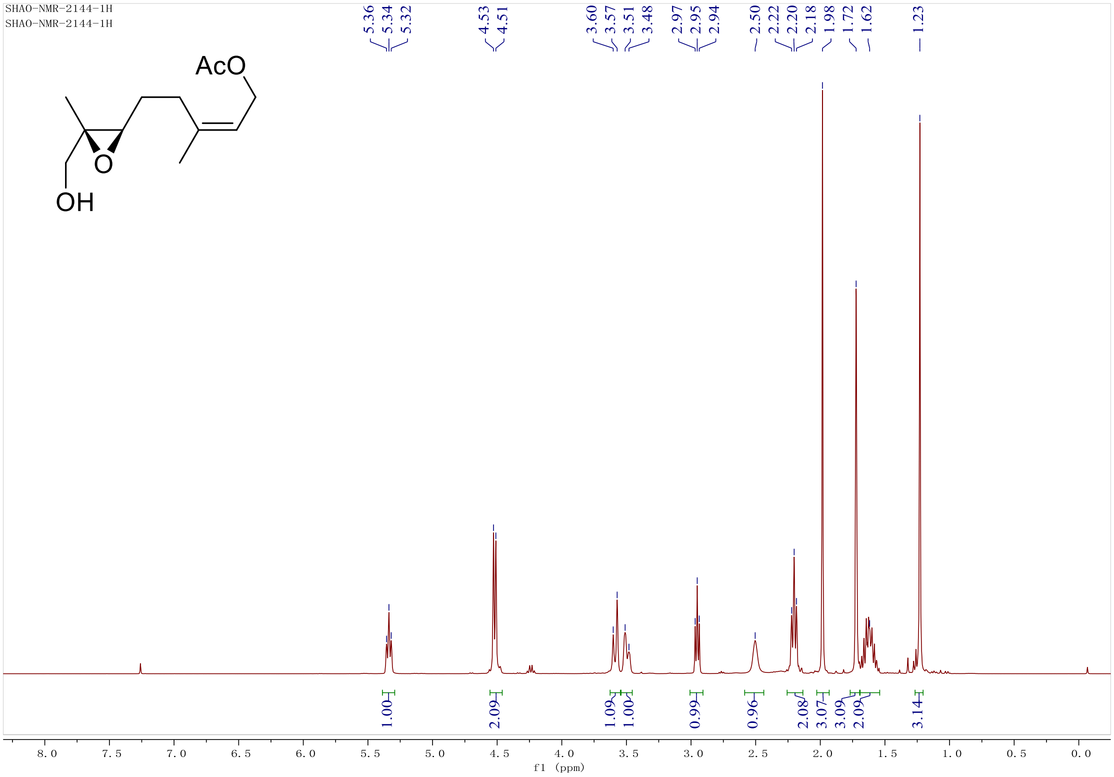

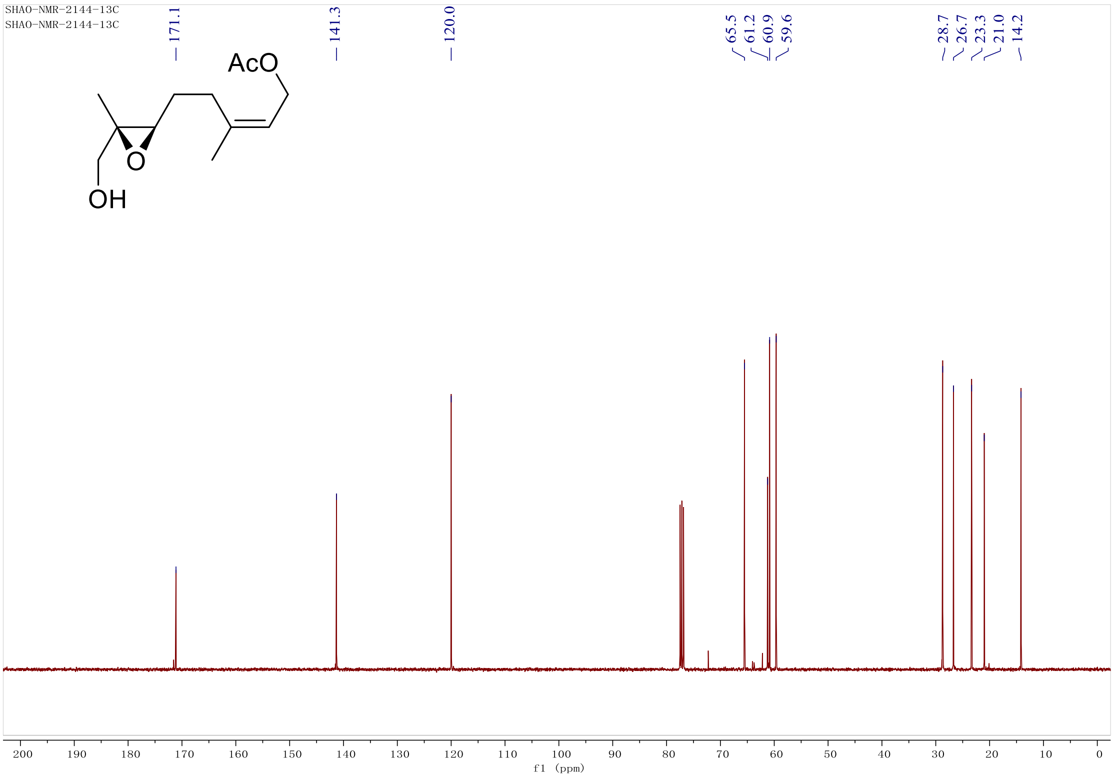

Supplementary Figure 17. ^{1}H (400 MHz, $CDCl_3$) and ^{13}C NMR (100 MHz, $CDCl_3$) spectra of compound **SI-6**

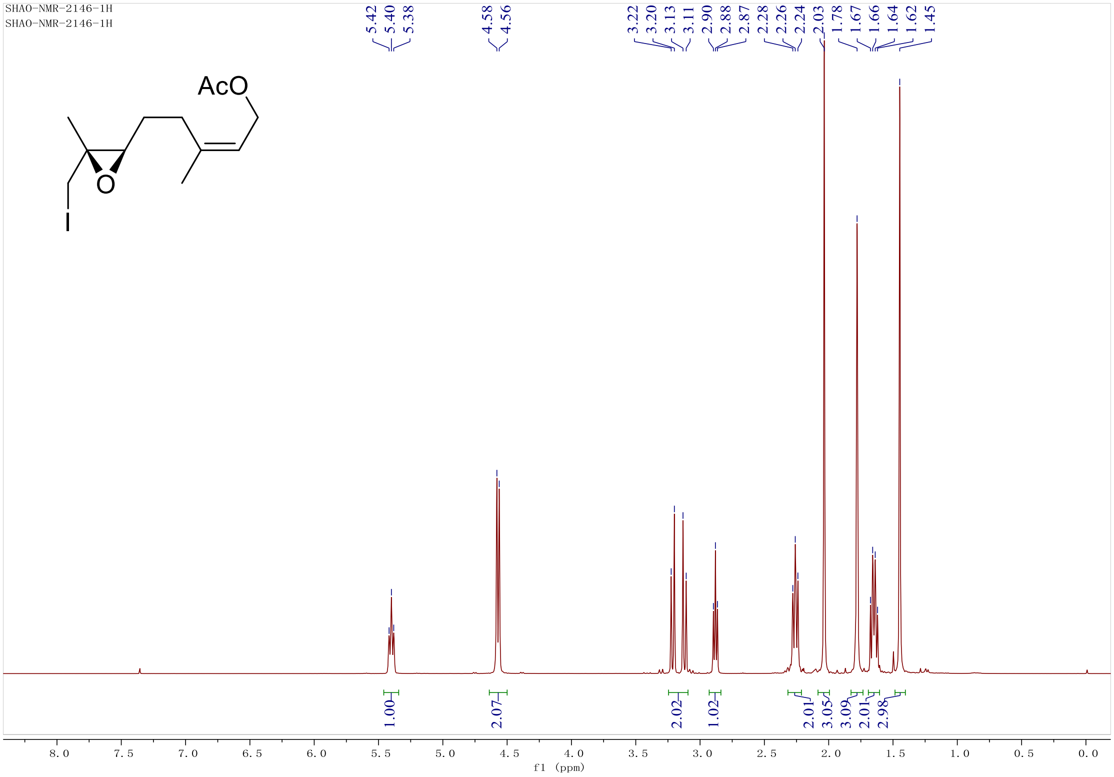

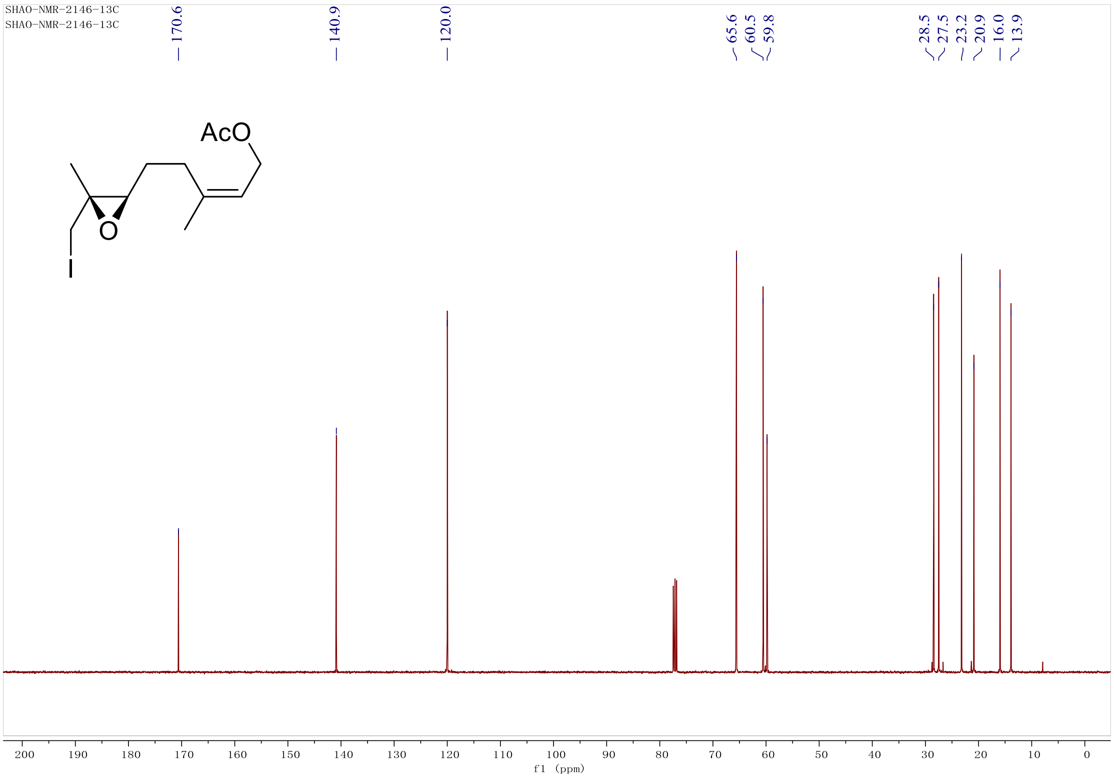

Supplementary Figure 18. ^{1}H (400 MHz, $CDCl_3$) and ^{13}C NMR (100 MHz, $CDCl_3$) spectra of compound **SI-7**

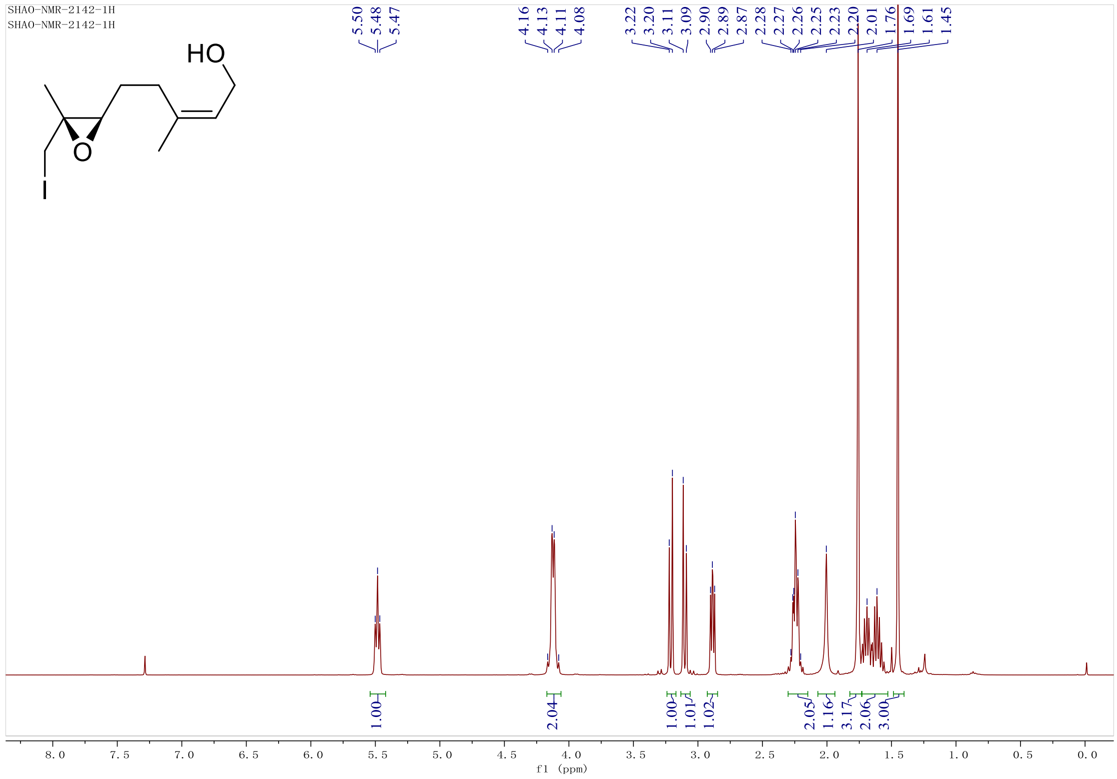

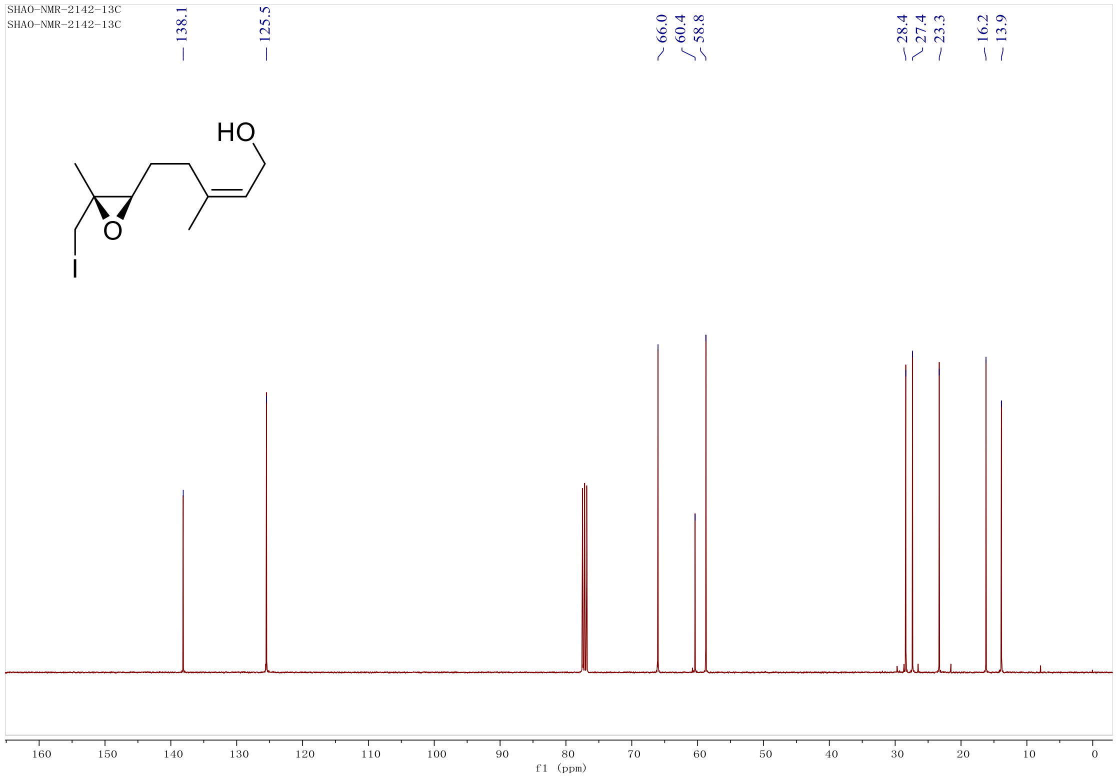

Supplementary Figure 19. ^{1}H (400 MHz, $CDCl_3$) and ^{13}C NMR (100 MHz, $CDCl_3$) spectra of compound **SI-8**

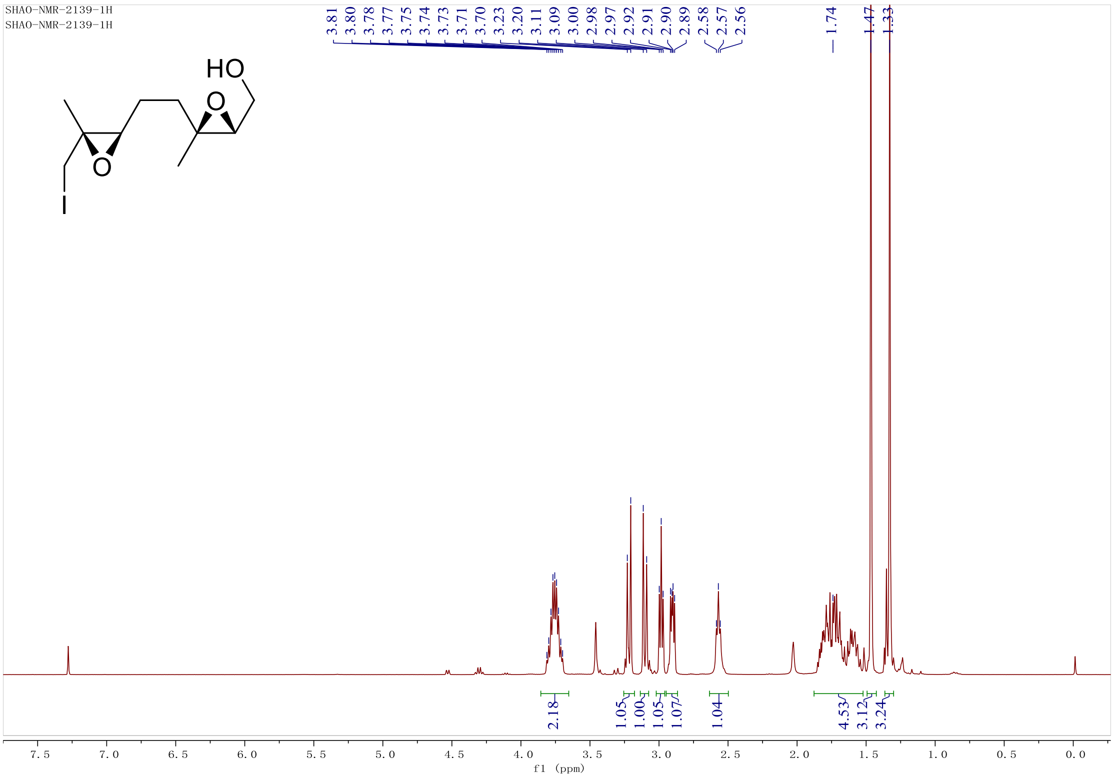

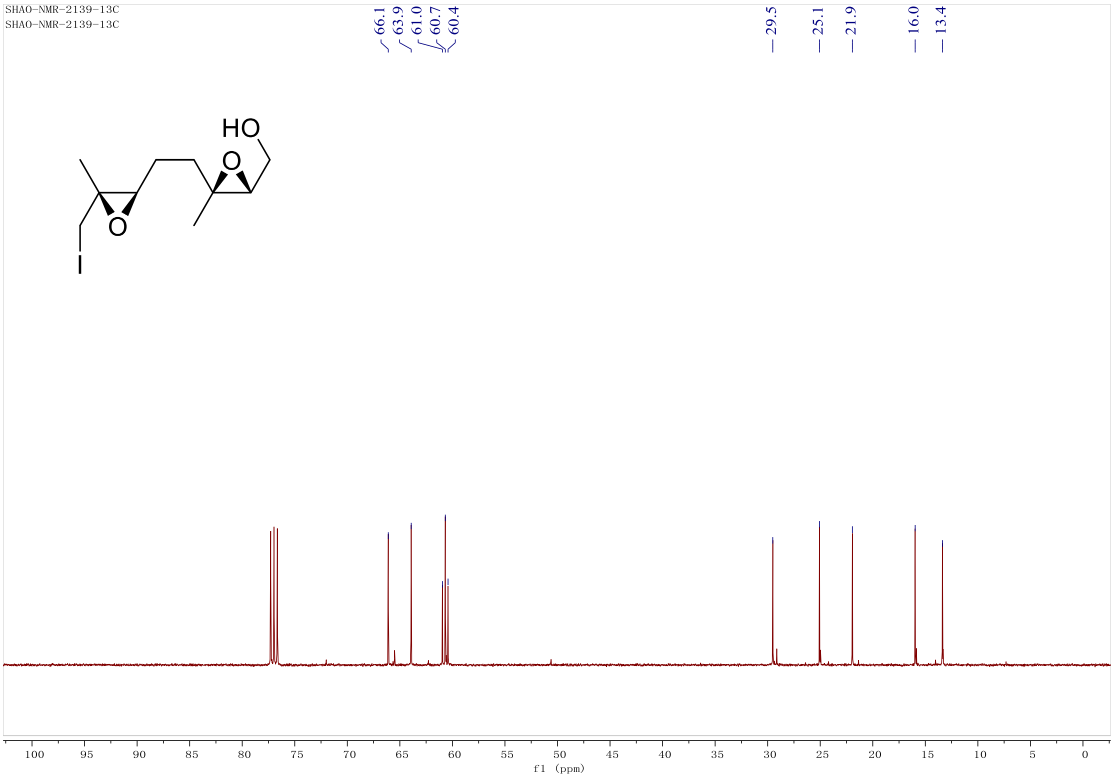

Supplementary Figure 20. ^{1}H (400 MHz, $CDCl_3$) and ^{13}C NMR (100 MHz, $CDCl_3$) spectra of compound **SI-9**

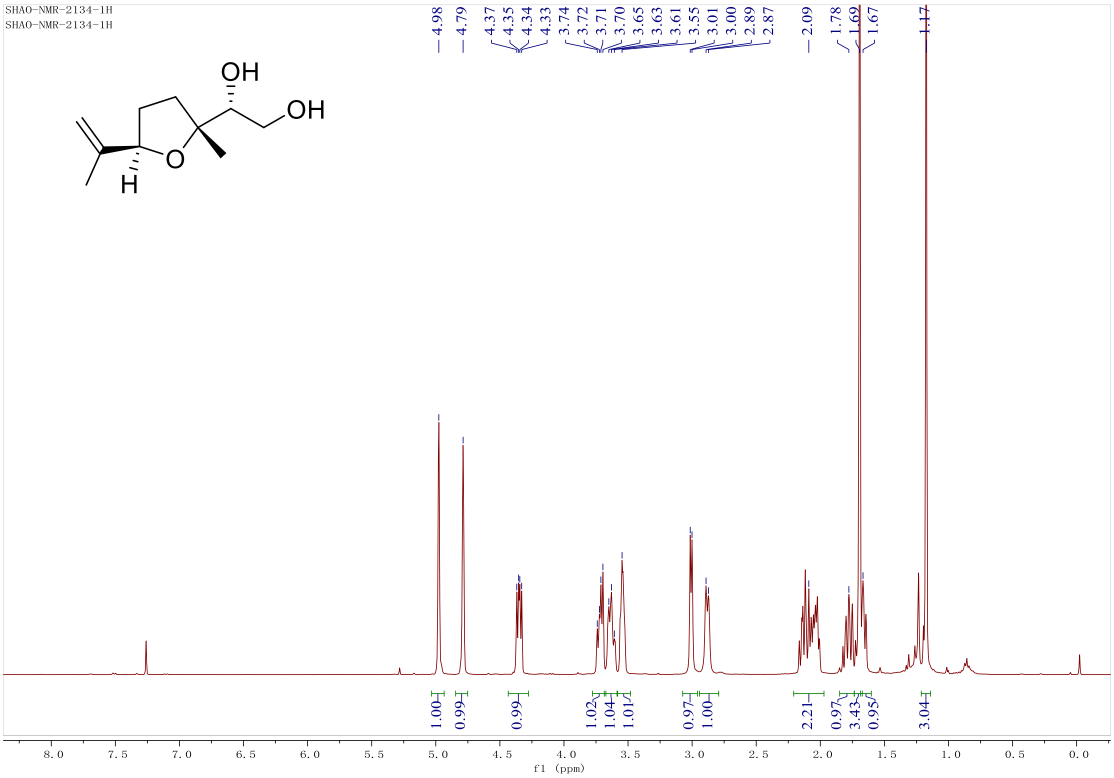

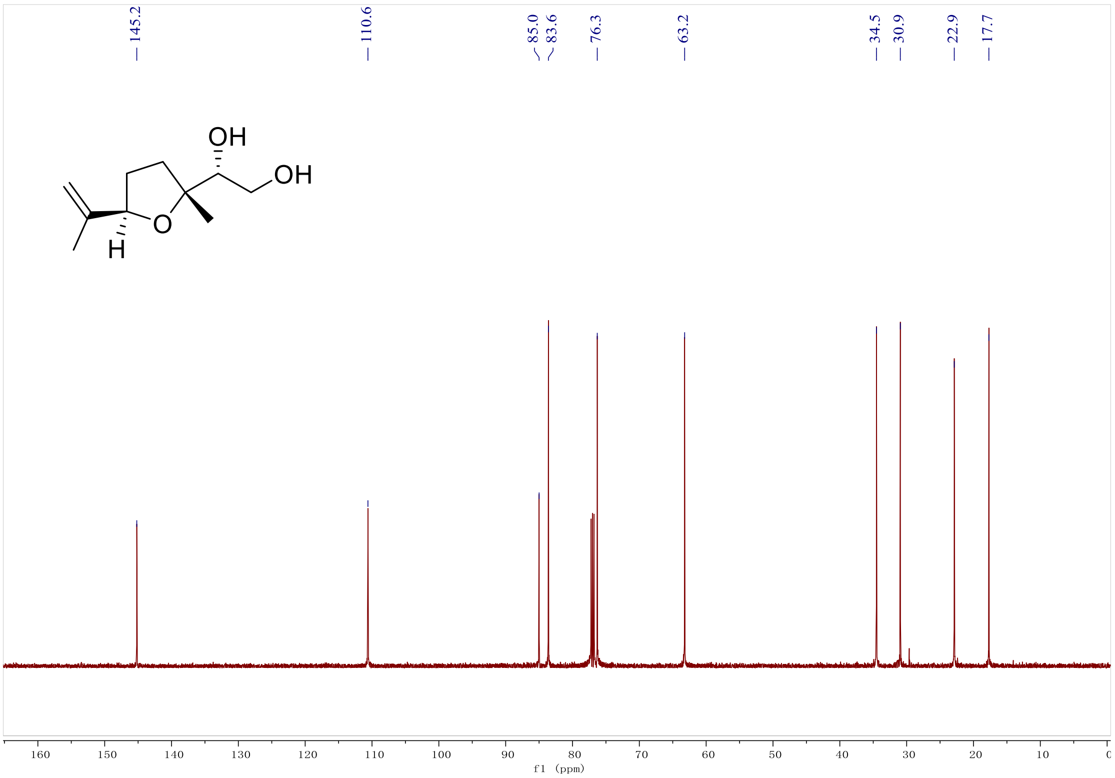

Supplementary Figure 21. ^{1}H (400 MHz, $CDCl_3$) and ^{13}C NMR (100 MHz, $CDCl_3$) spectra of compound **16**

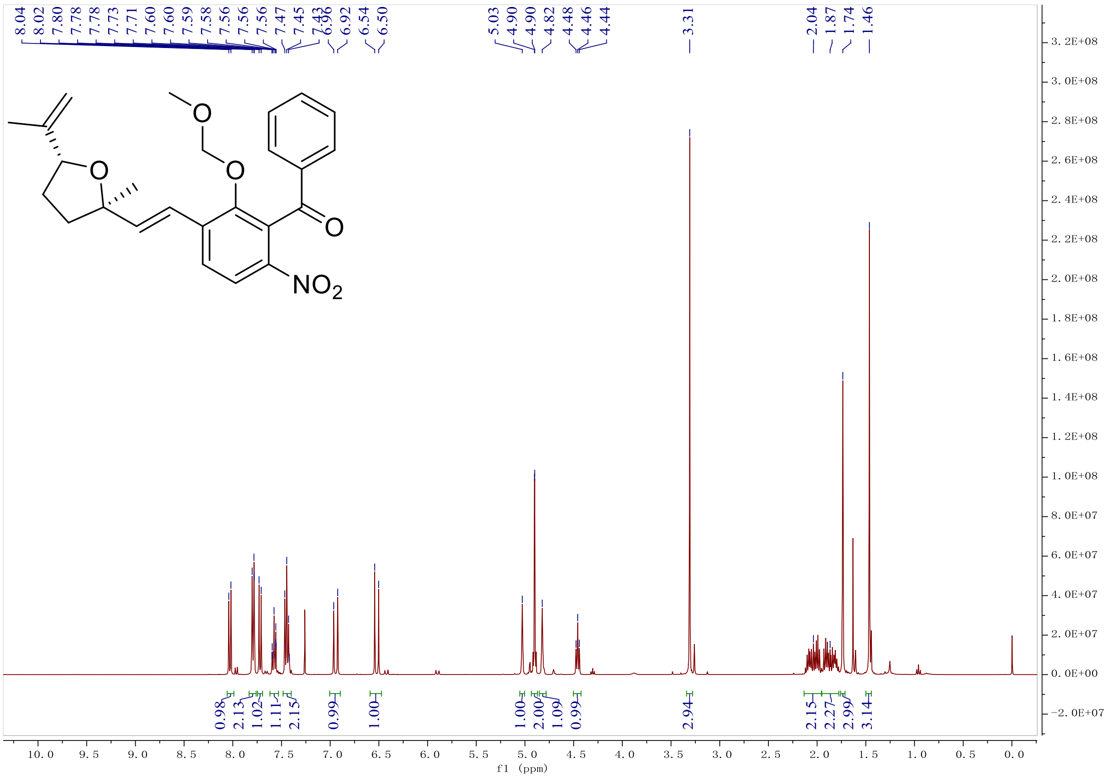

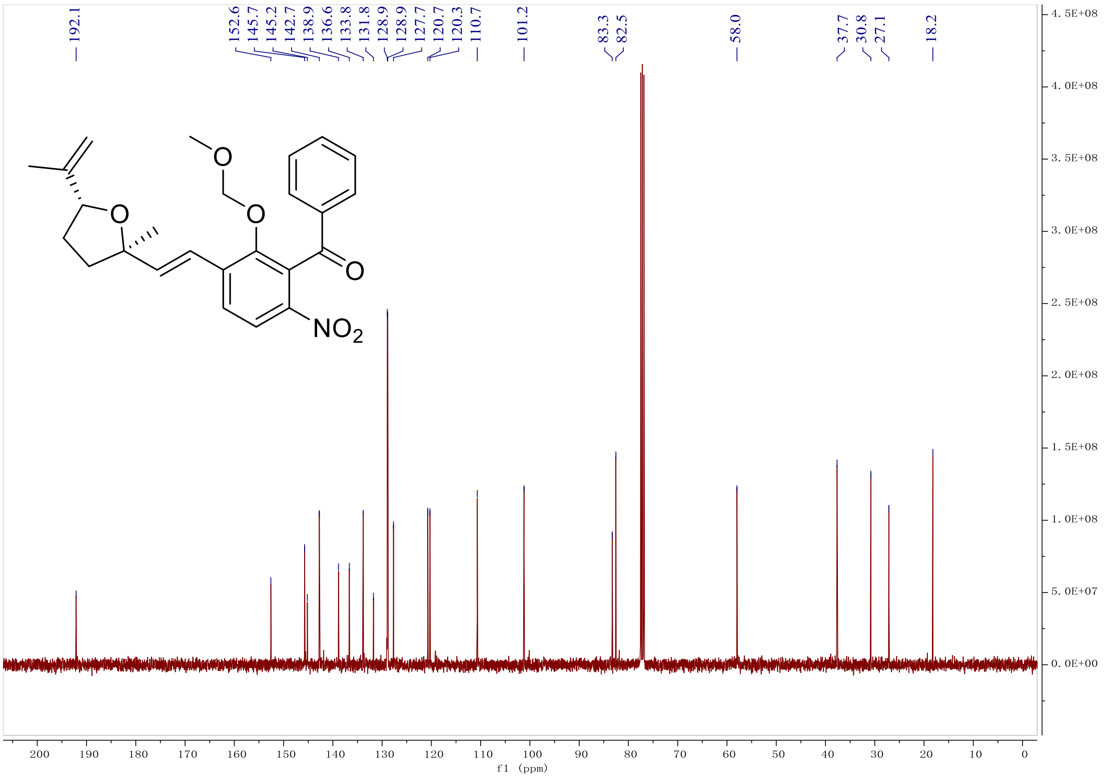

Supplementary Figure 22. ^{1}H (400 MHz, $CDCl_3$) and ^{13}C NMR (100 MHz, $CDCl_3$) spectra of compound **11**

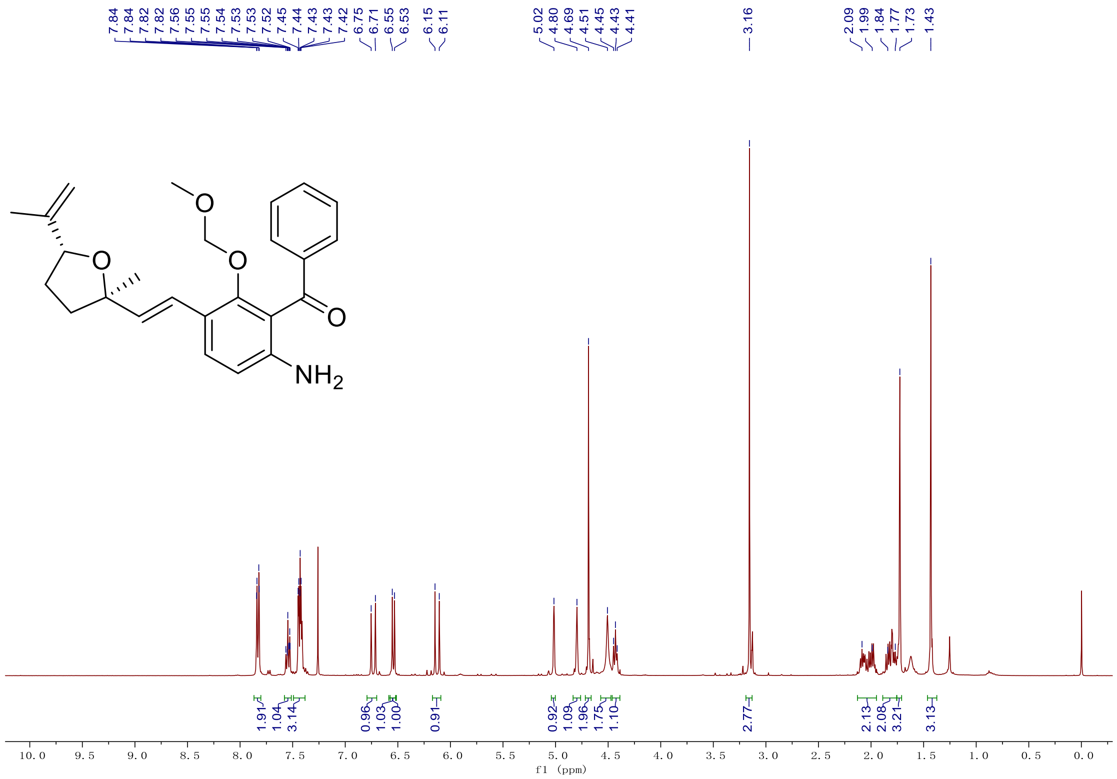

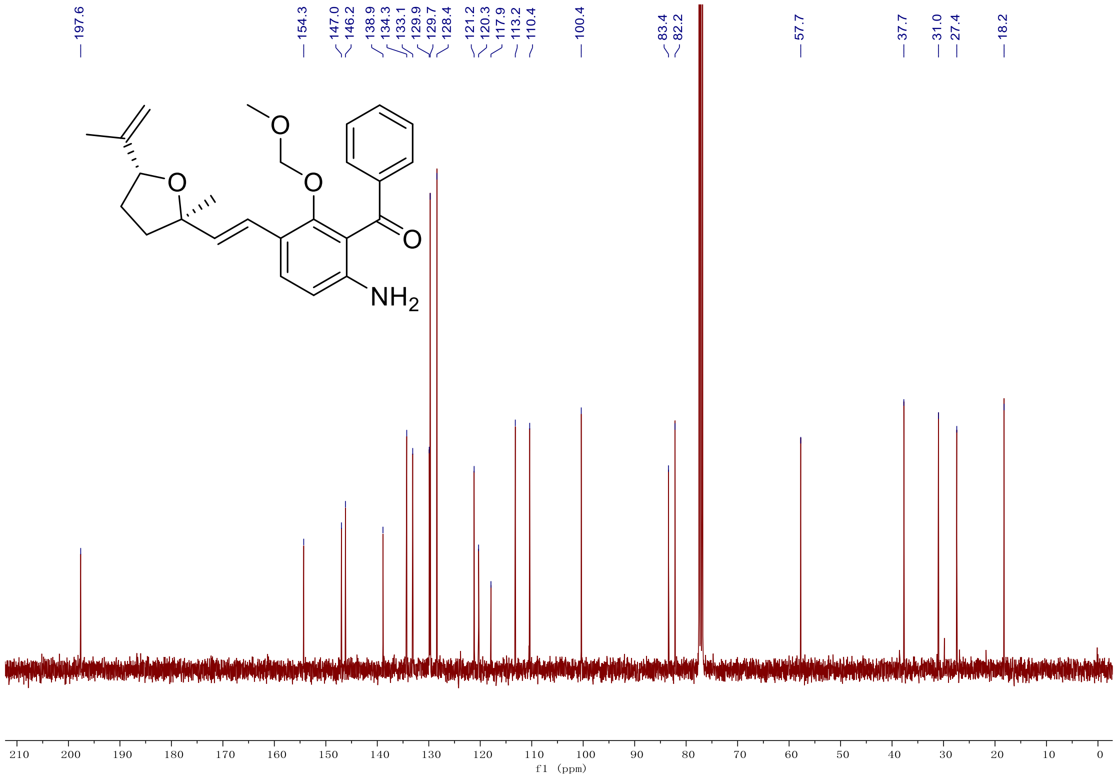

Supplementary Figure 23. ^{1}H (400 MHz, $CDCl_3$) and ^{13}C NMR (100 MHz, $CDCl_3$) spectra of compound **22**

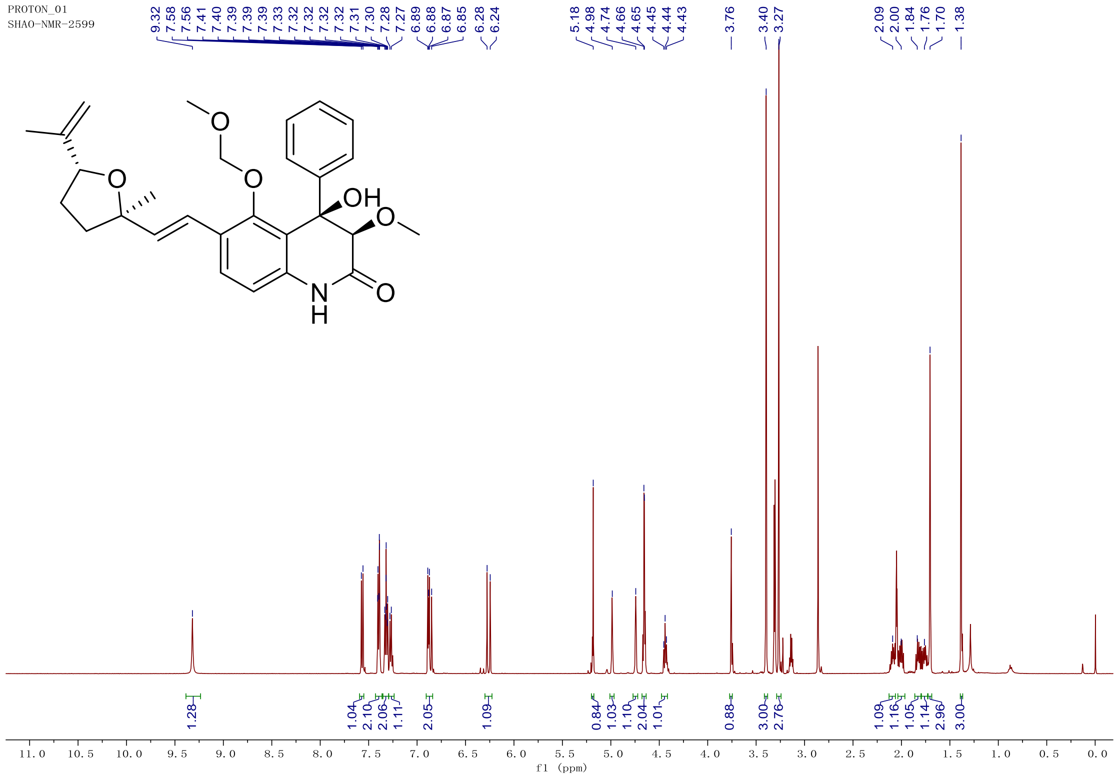

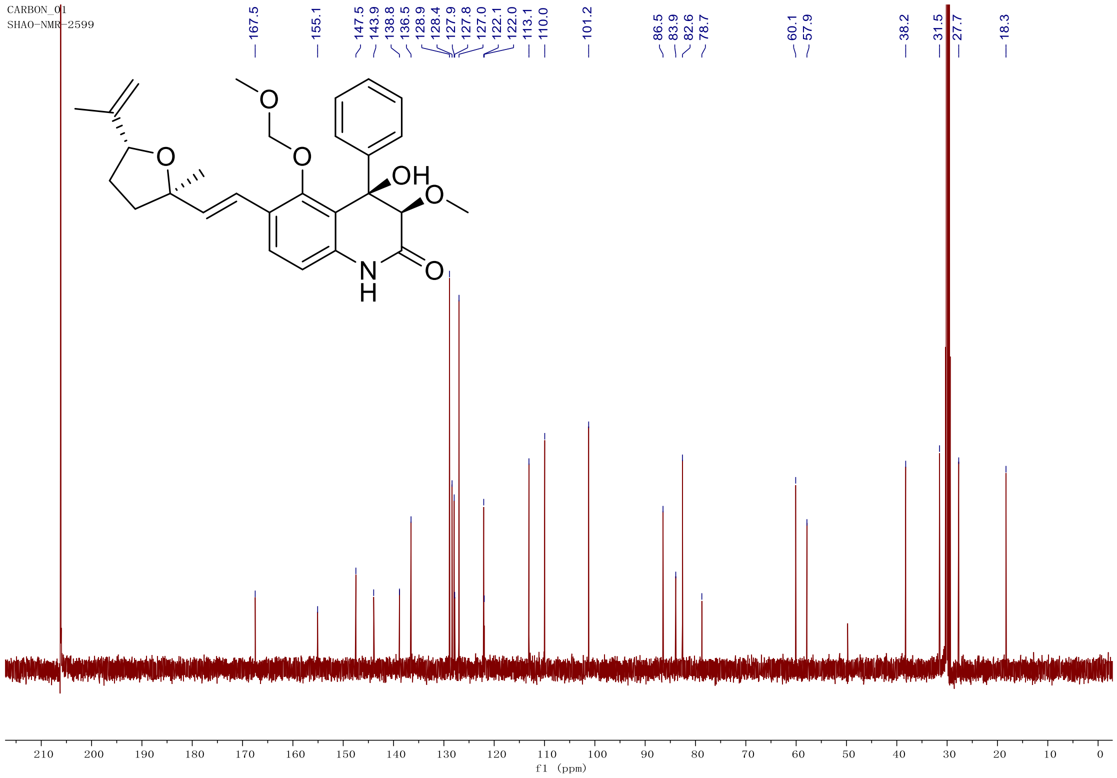

Supplementary Figure 24. ^{1}H (500 MHz, acetone-d_6) and ^{13}C NMR (125 MHz, acetone-d_6) spectra of compound **23a**

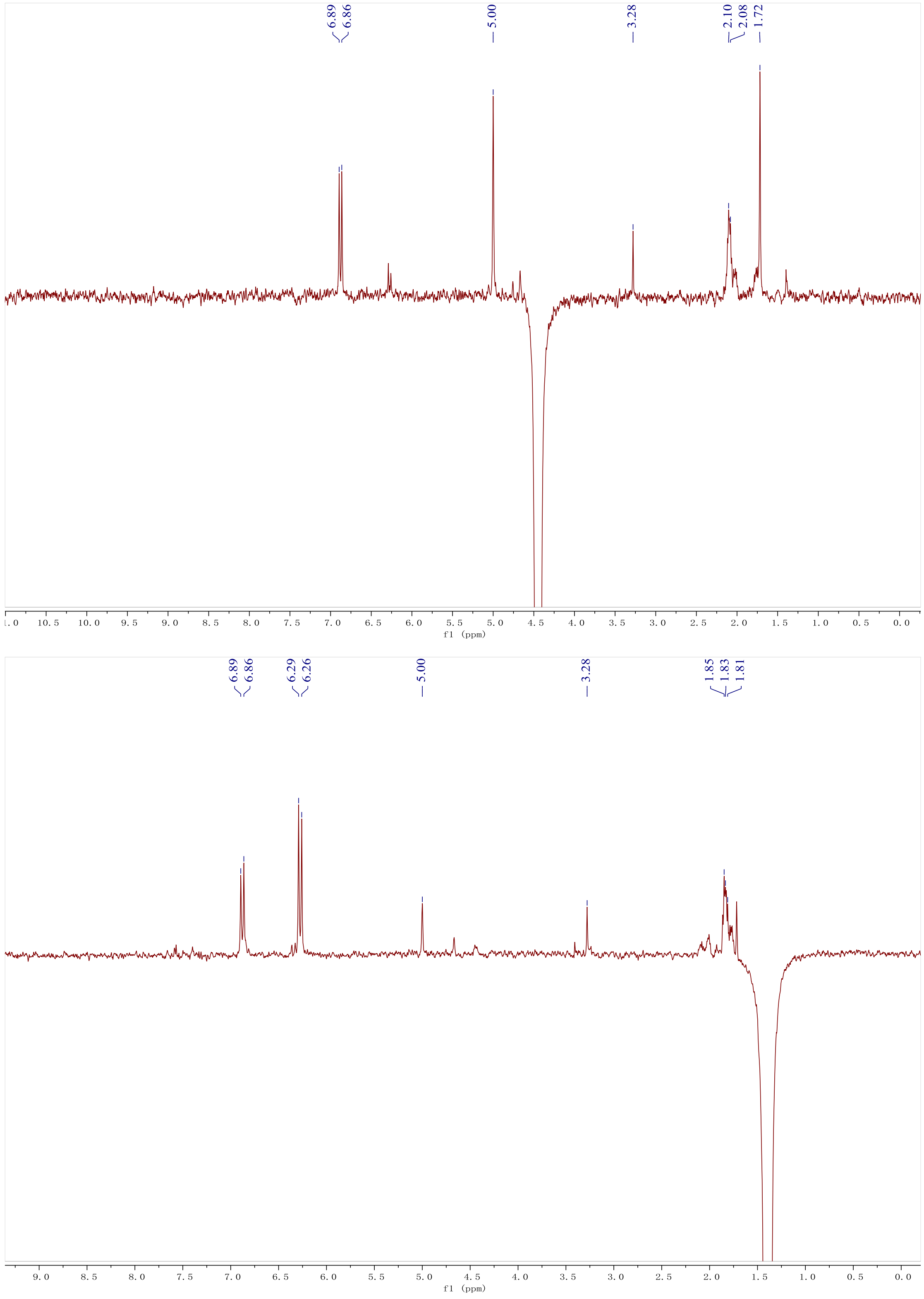

Supplementary Figure 25. NOE (500 MHz, acetone-d_6) spectra of compound **23a**

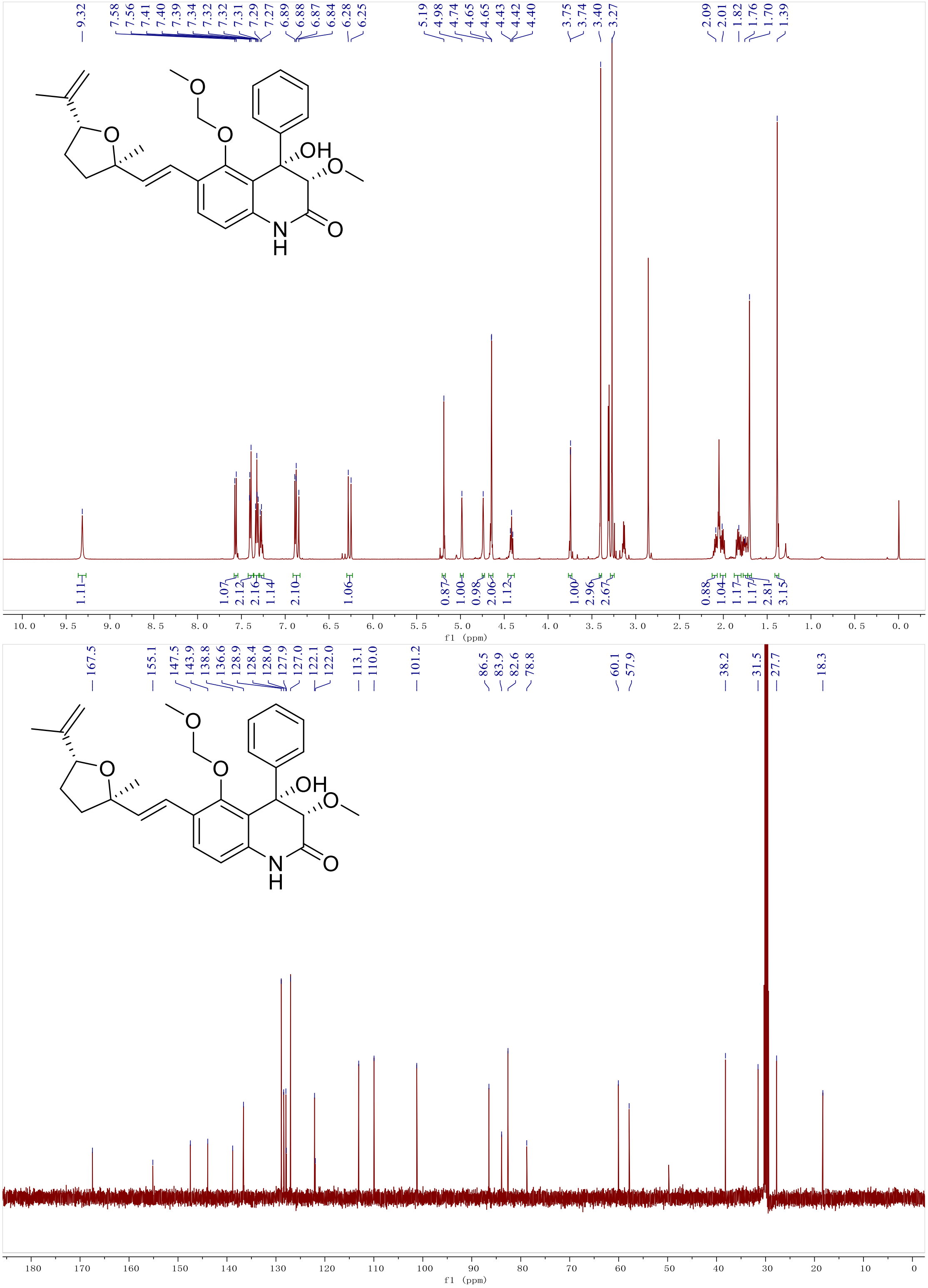

Supplementary Figure 26. ^{1}H (500 MHz, acetone-d_6) and ^{13}C NMR (125 MHz, acetone-d_6) spectra of compound **23**

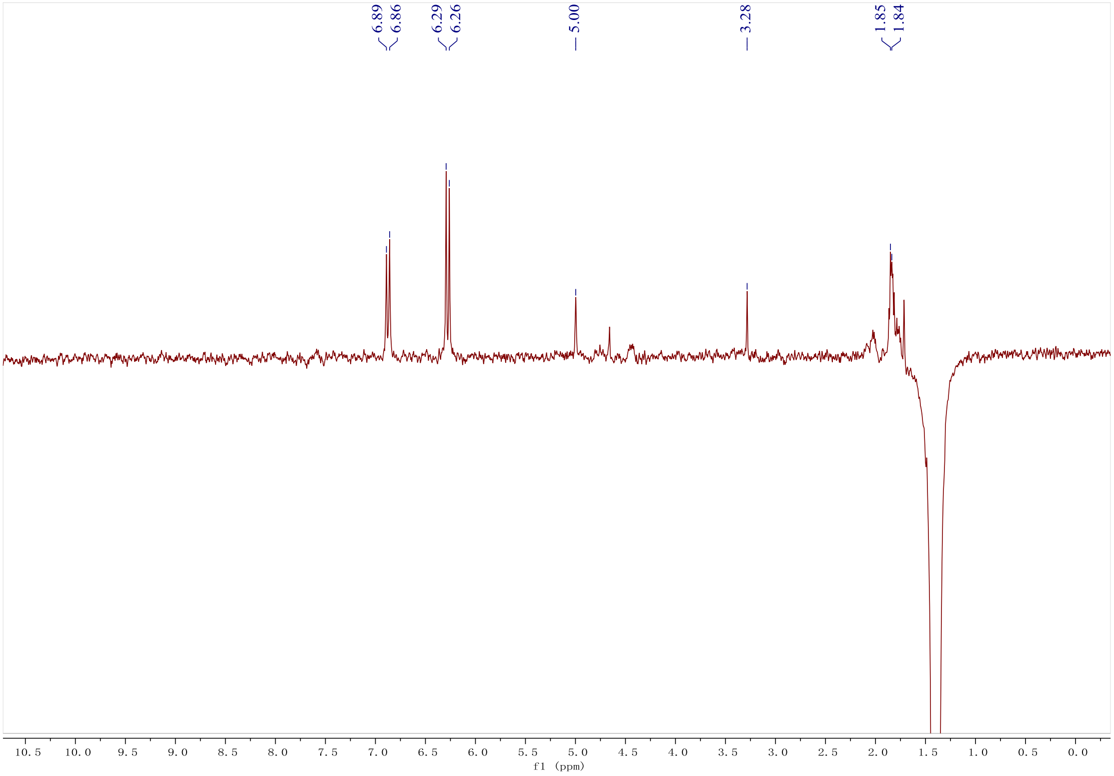

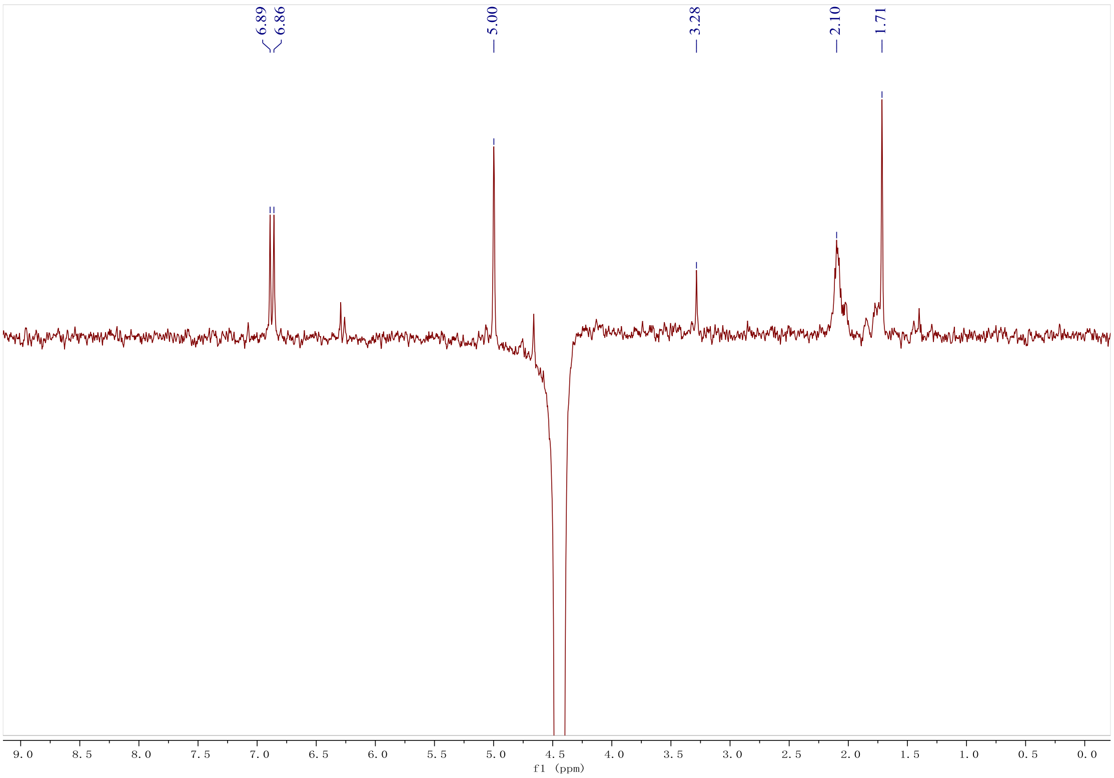

Supplementary Figure 27. NOE (500 MHz, acetone-d_6) spectra of compound **23**

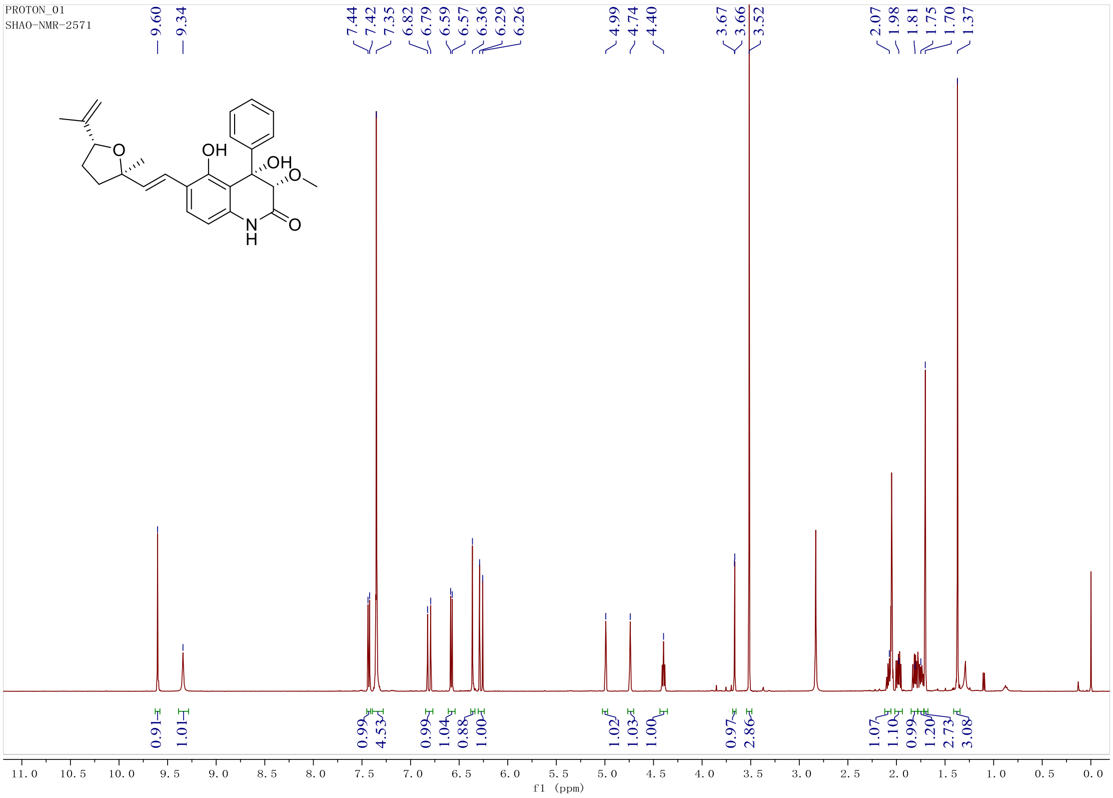

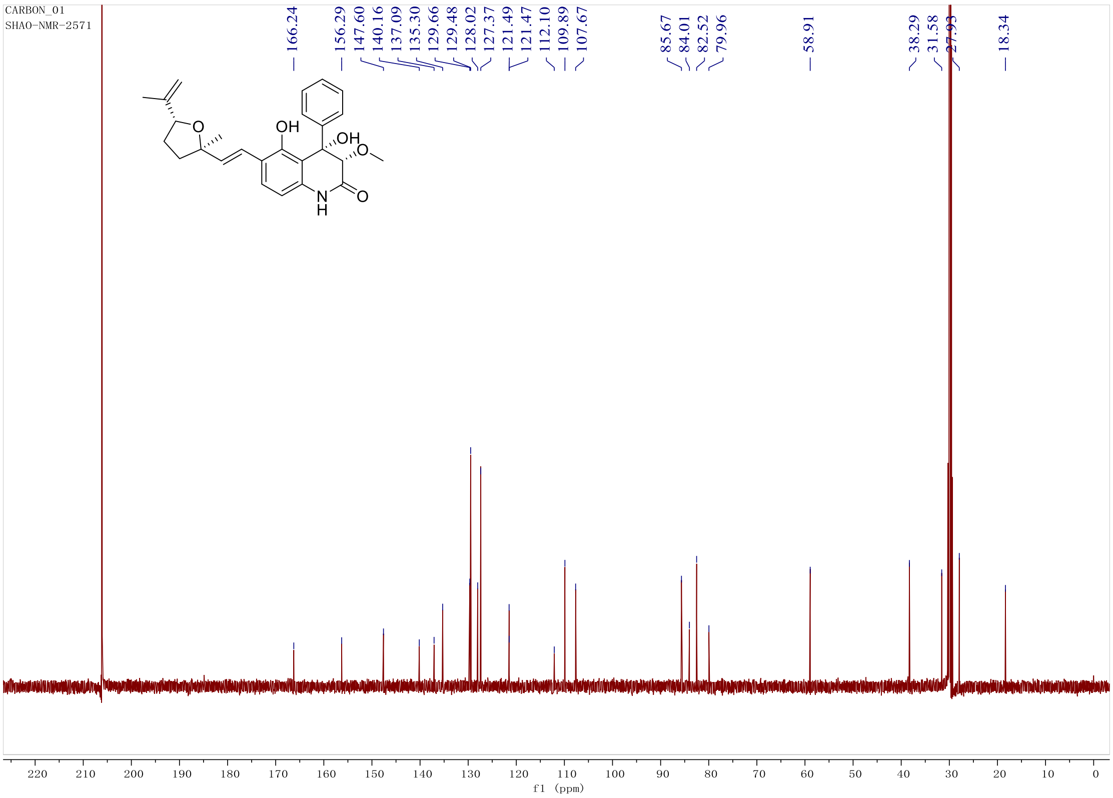

Supplementary Figure 28. ^{1}H (500 MHz, acetone-d_6) and ^{13}C NMR (125 MHz, acetone-d_6) spectra of compound **1**

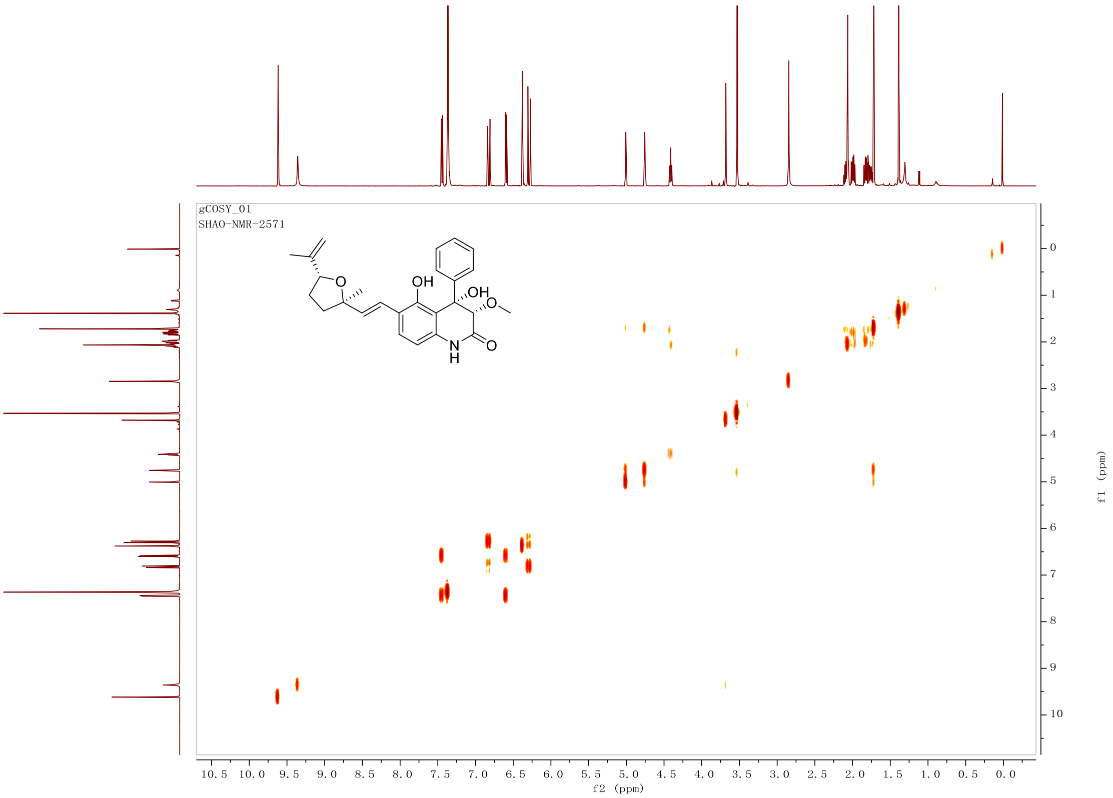

Supplementary Figure 29. ^{1}H-^{1}H COSY (500 MHz, acetone-d_6) spectra of compound **1**

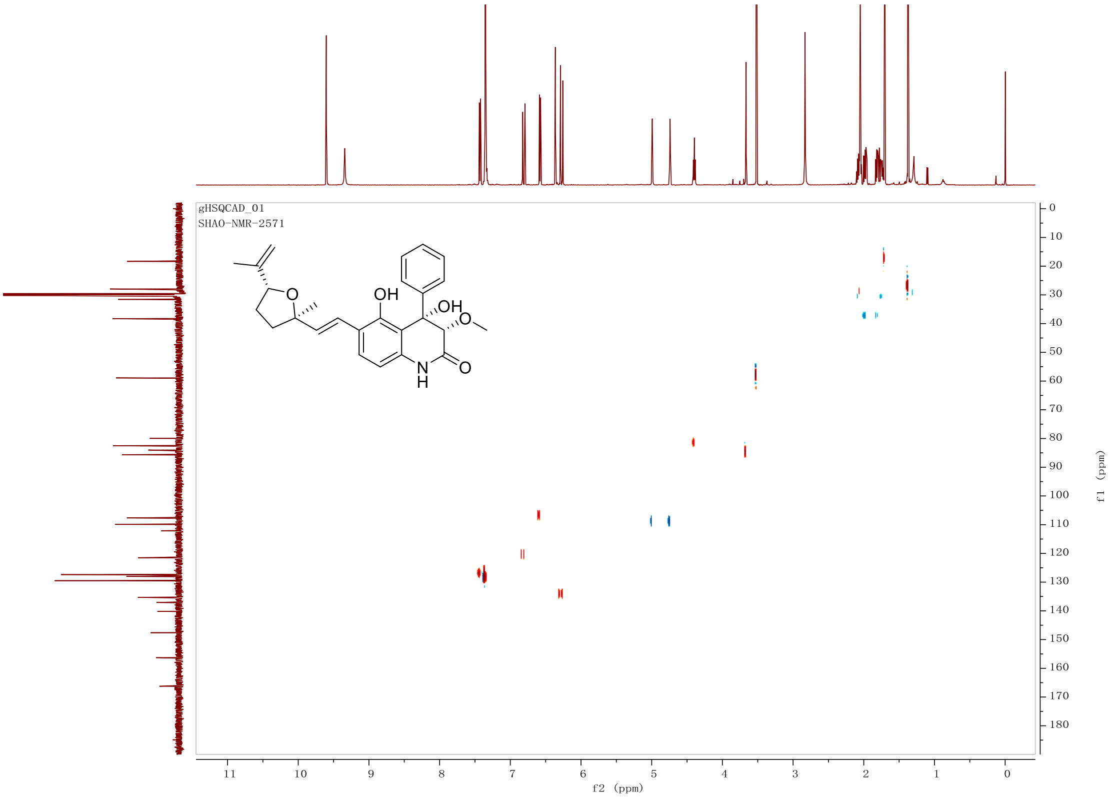

Supplementary Figure 30. HSQC (500 MHz, acetone-d_6) spectra of compound **1**

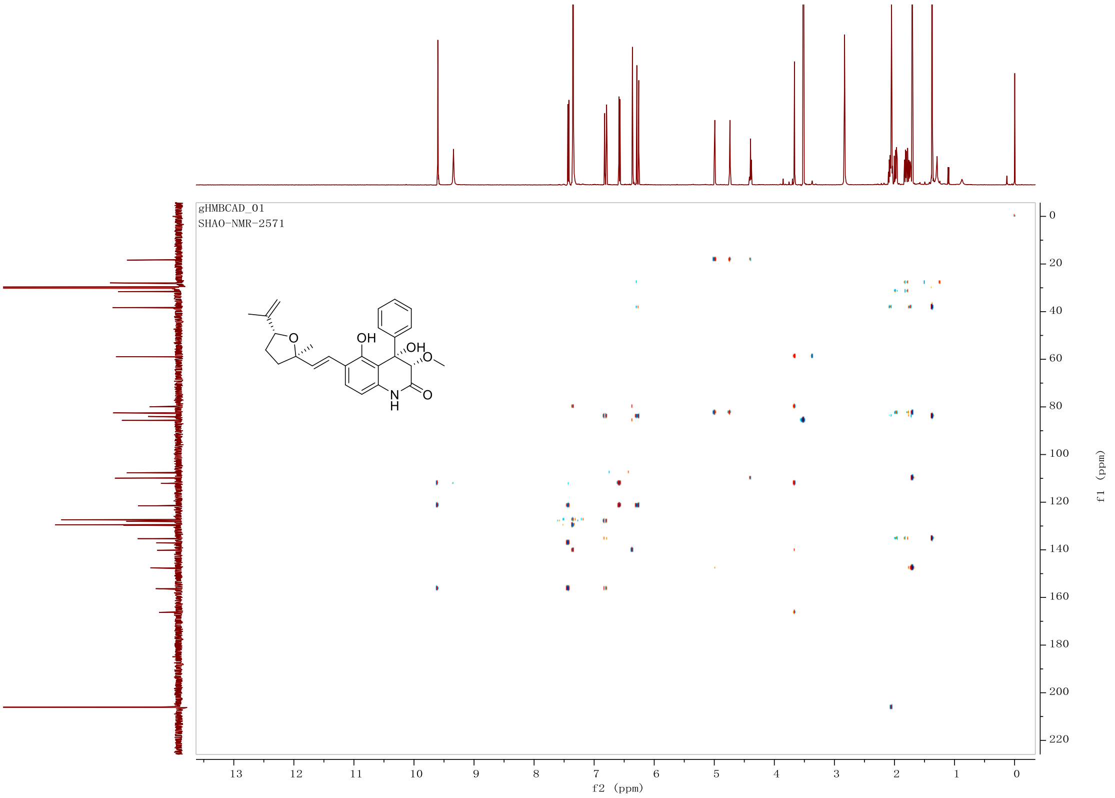

Supplementary Figure 31. HMBC (500 MHz, acetone-d_6) spectra of compound **1**

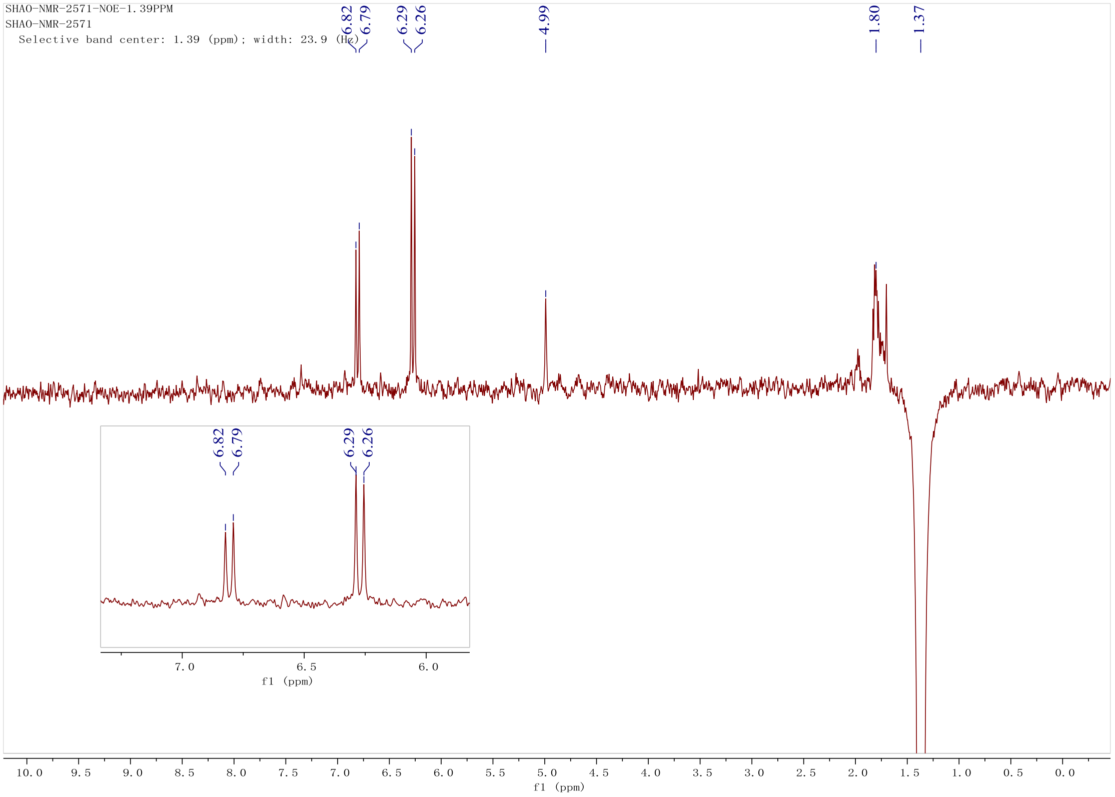

Supplementary Figure 32. NOE (500 MHz, acetone-d_6) spectra of compound **1**

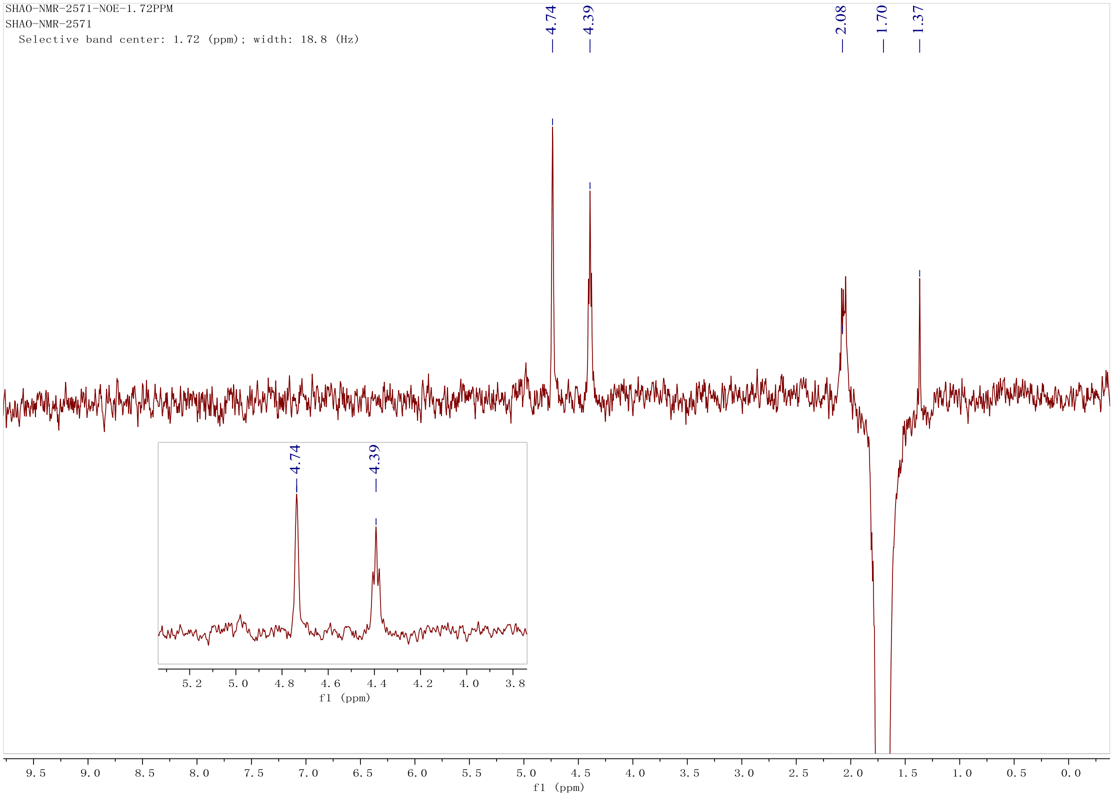

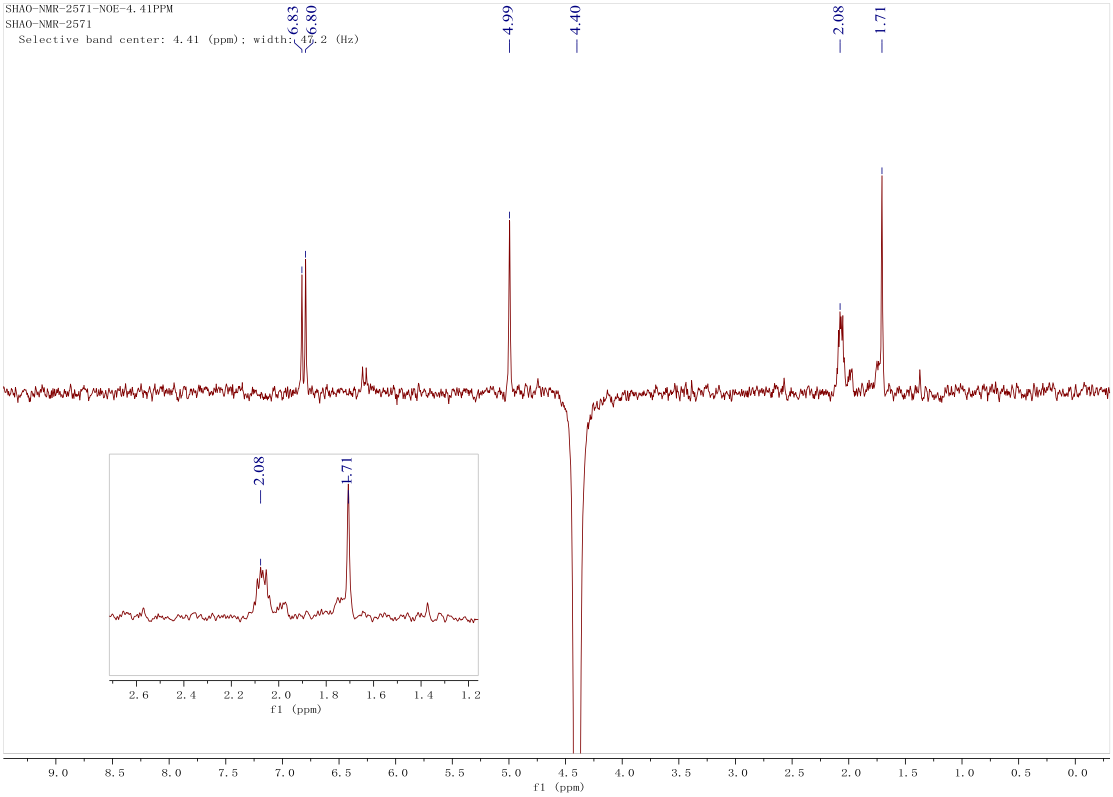

Supplementary Figure 33. NOE (500 MHz, acetone-d_6) spectra of compound **1**

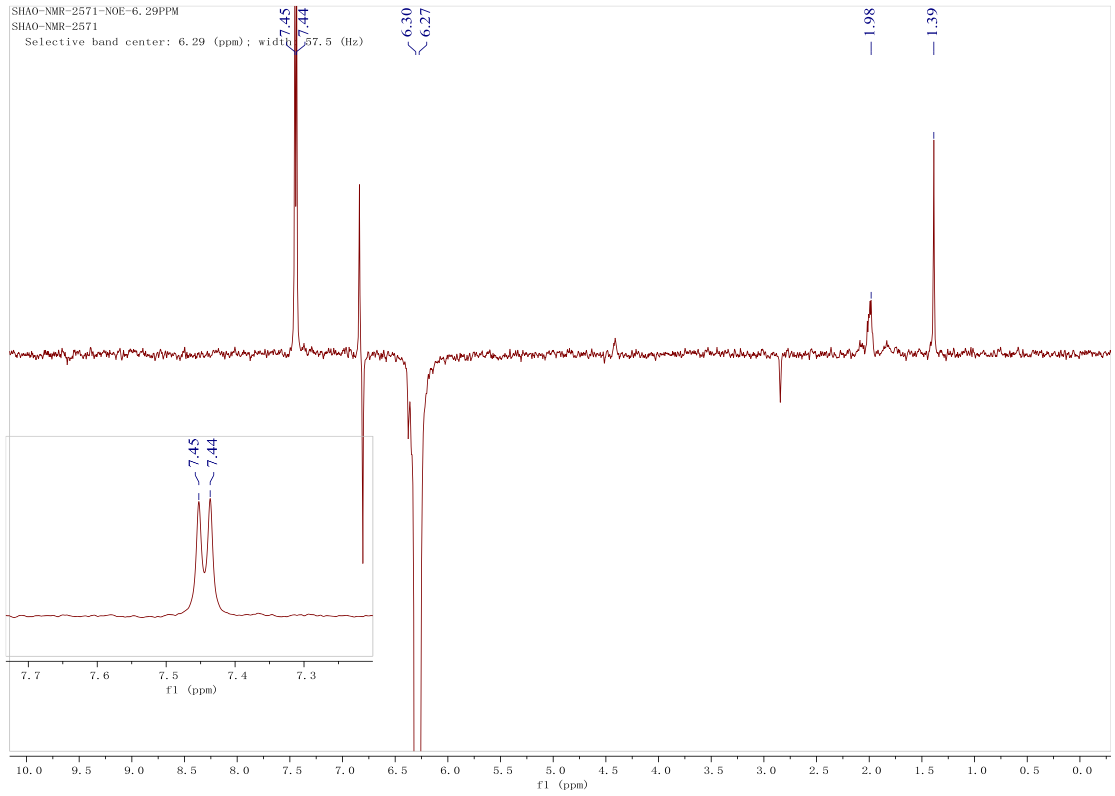

Supplementary Figure 34. NOE (500 MHz, acetone-d_6) spectra of compound **1**

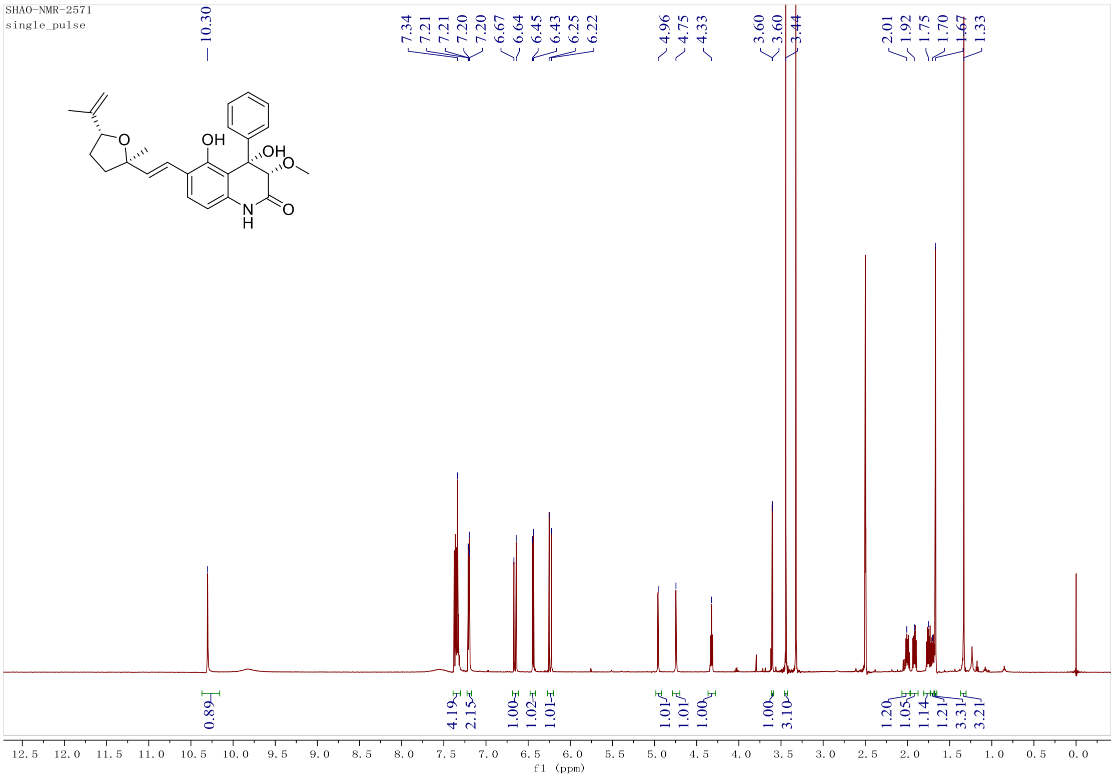

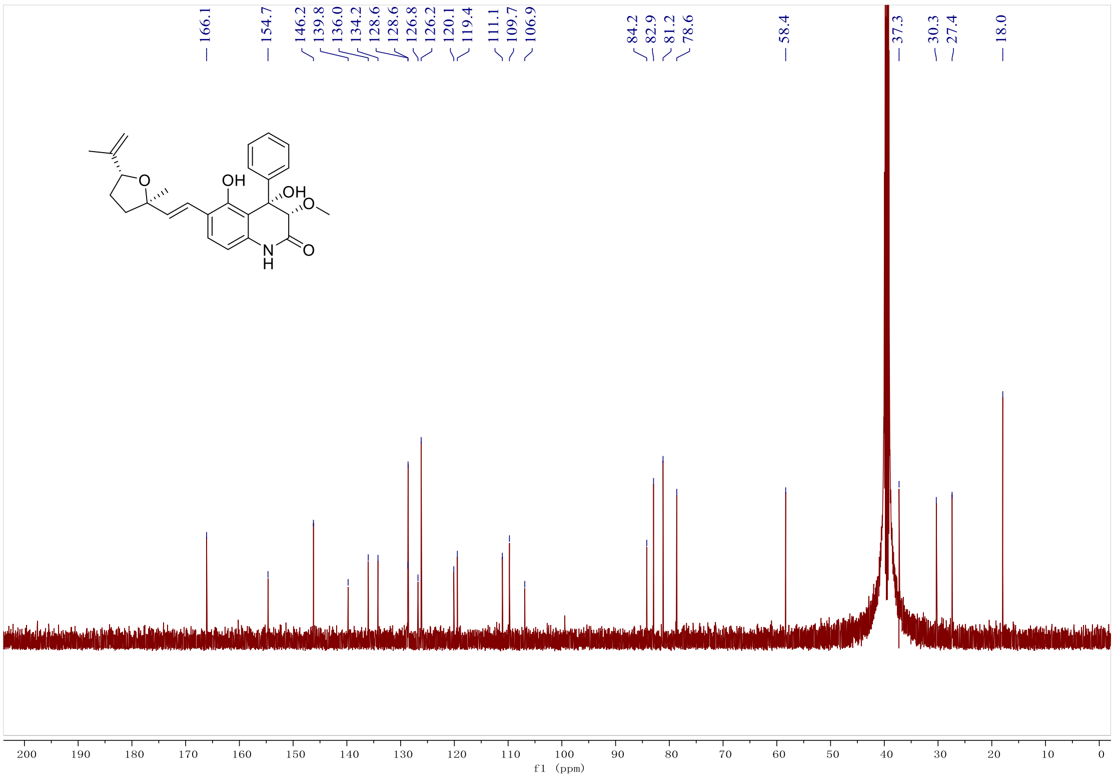

Supplementary Figure 35. ^{1}H NMR (600 MHz, DMSO-d_6) and ^{13}C NMR (150 MHz, DMSO-d_6) spectra of compound **1**

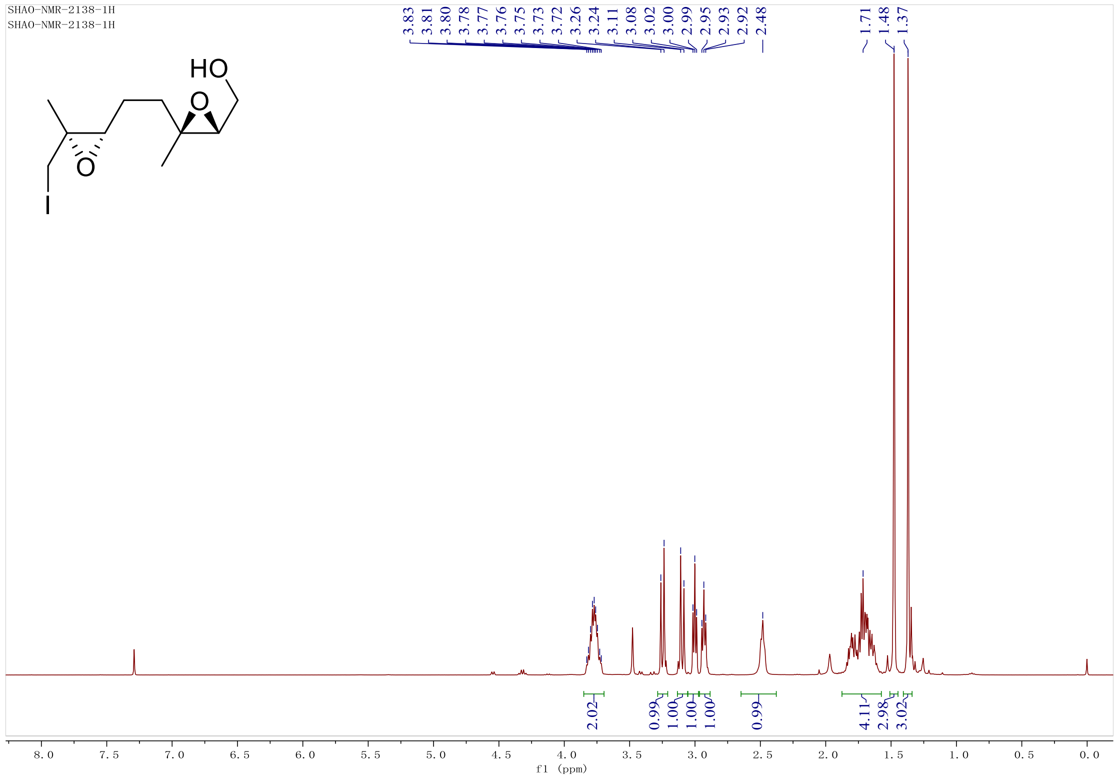

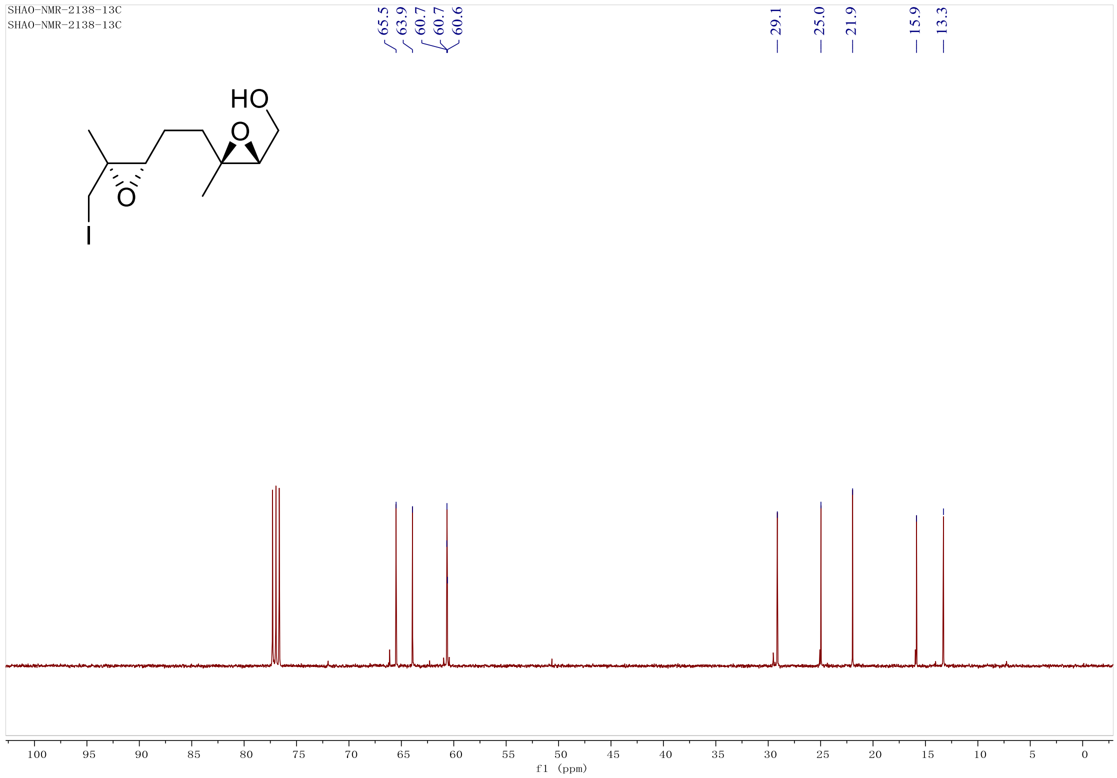

Supplementary Figure 36. ^{1}H (400 MHz, $CDCl_3$) and ^{13}C NMR (100 MHz, $CDCl_3$) spectra of compound **SI-11**

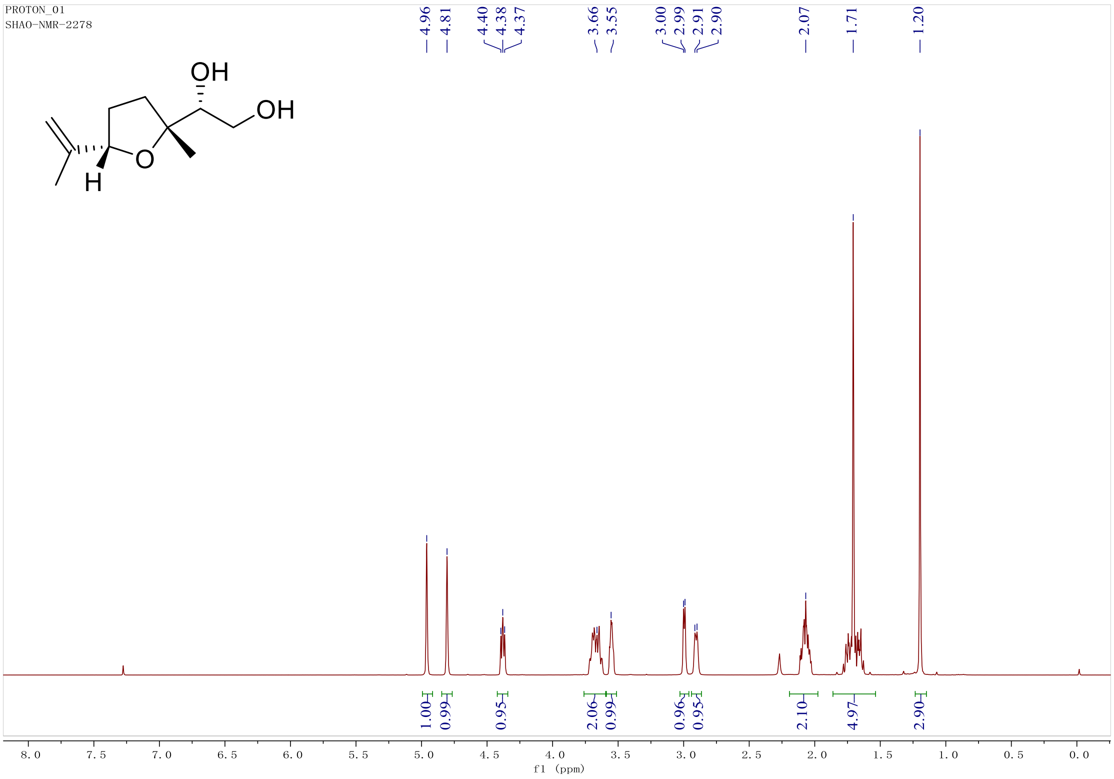

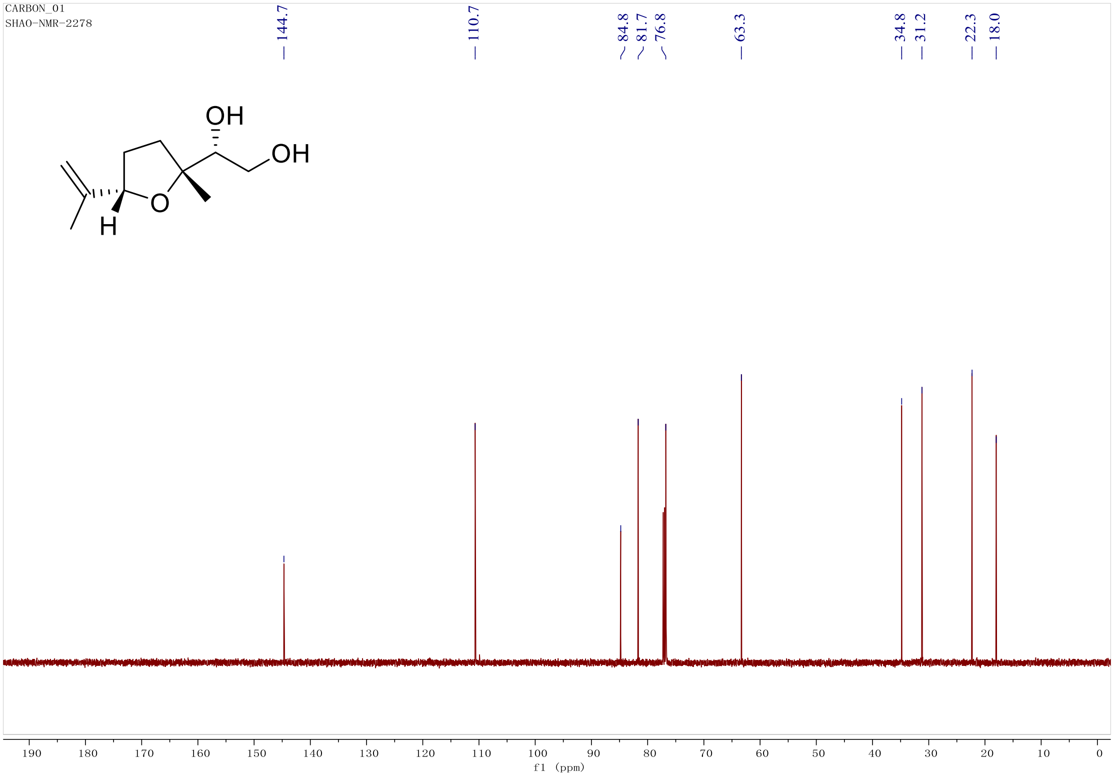

Supplementary Figure 37. ^{1}H (400 MHz, $CDCl_3$) and ^{13}C NMR (100 MHz, $CDCl_3$) spectra of compound **16a**

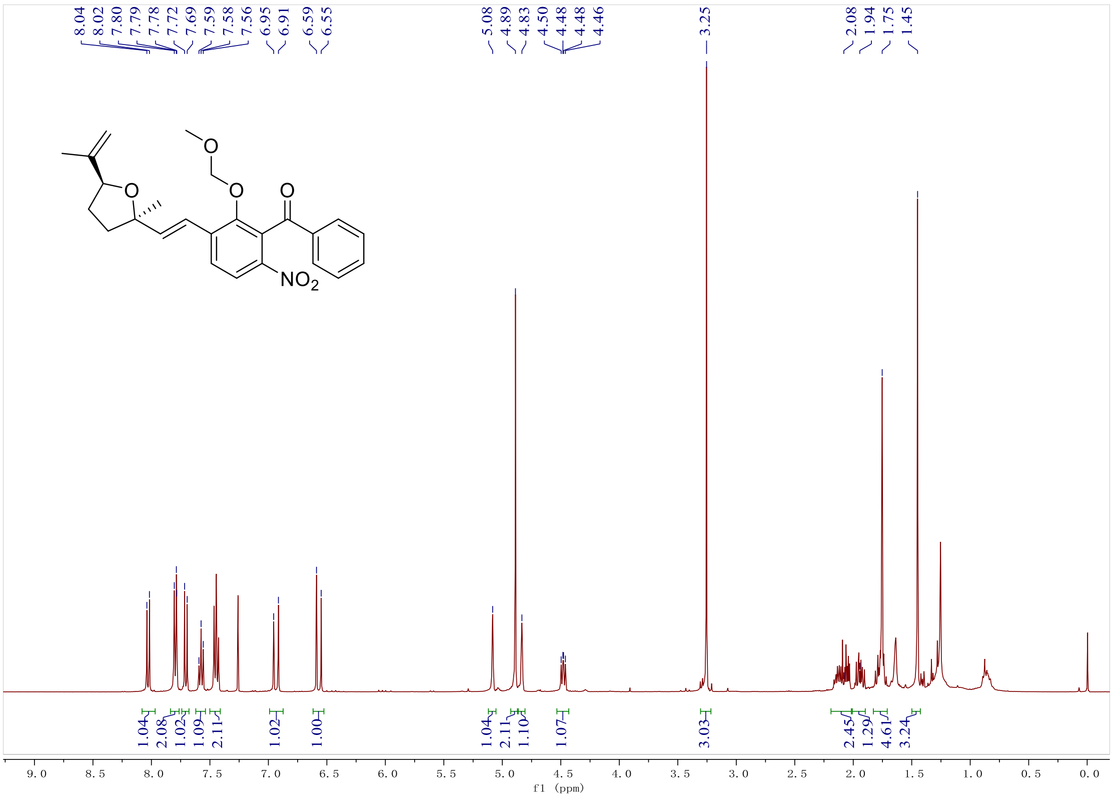

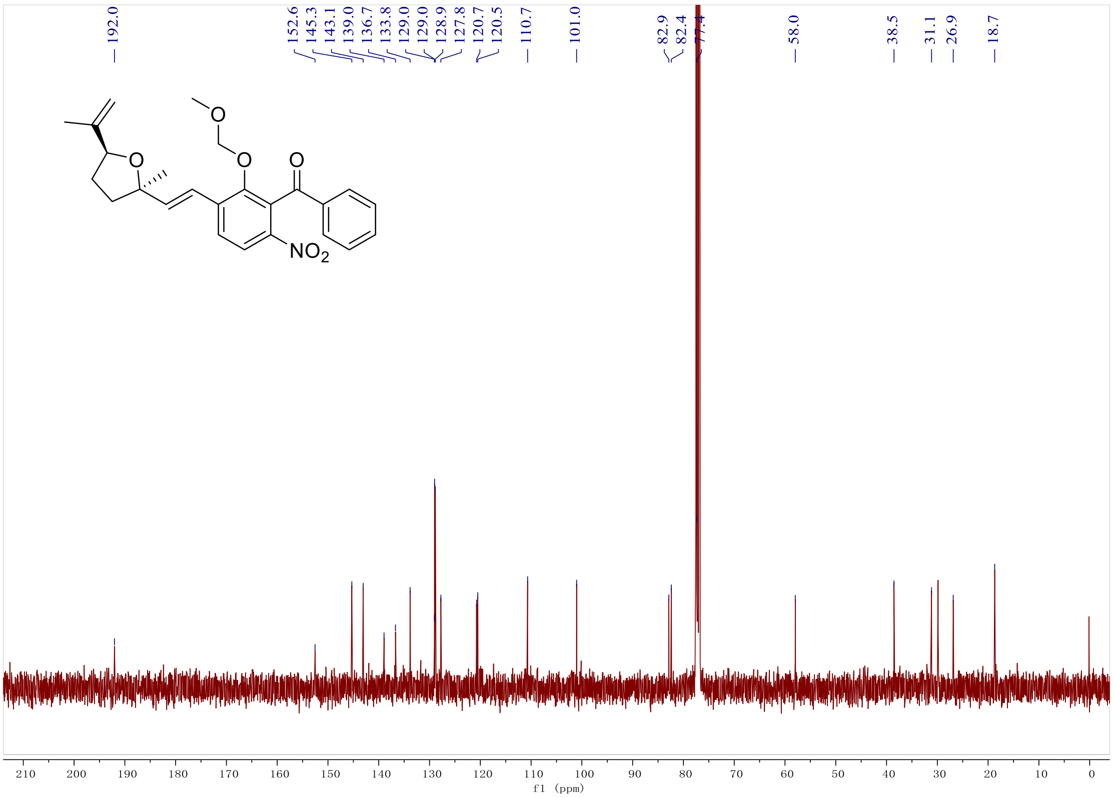

Supplementary Figure 38. ^{1}H (400 MHz, $CDCl_3$) and ^{13}C NMR (100 MHz, $CDCl_3$) spectra of compound **SI-12**

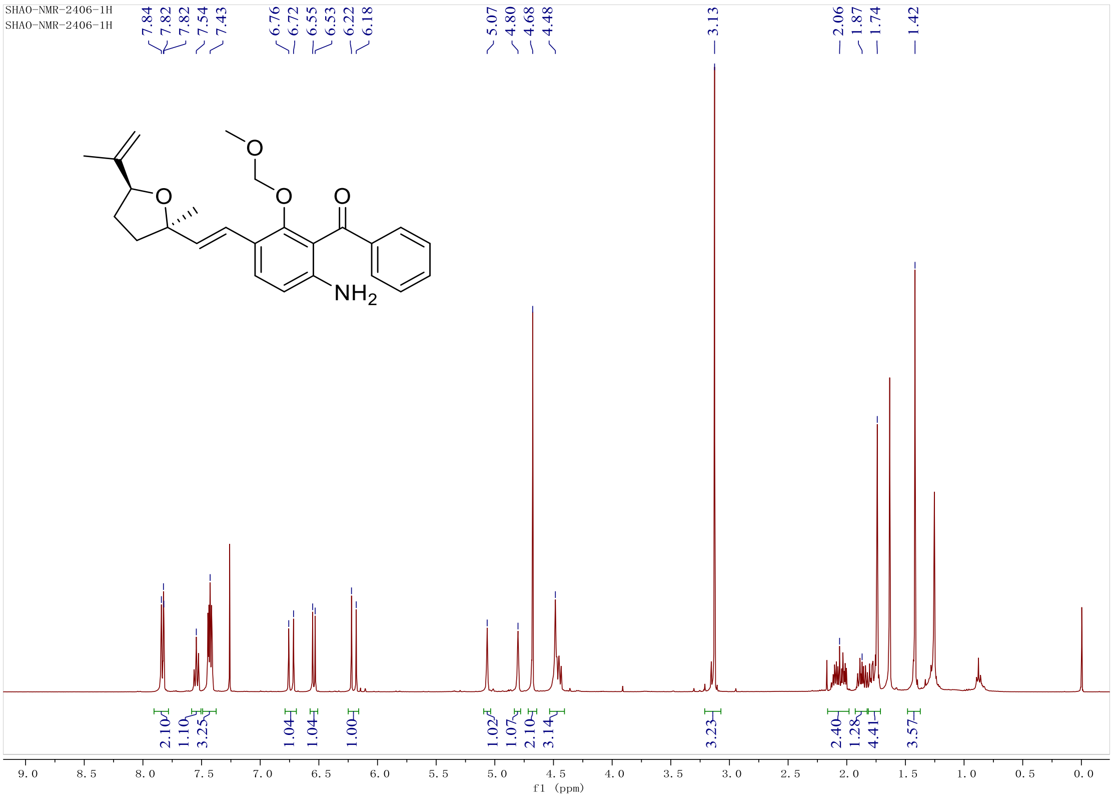

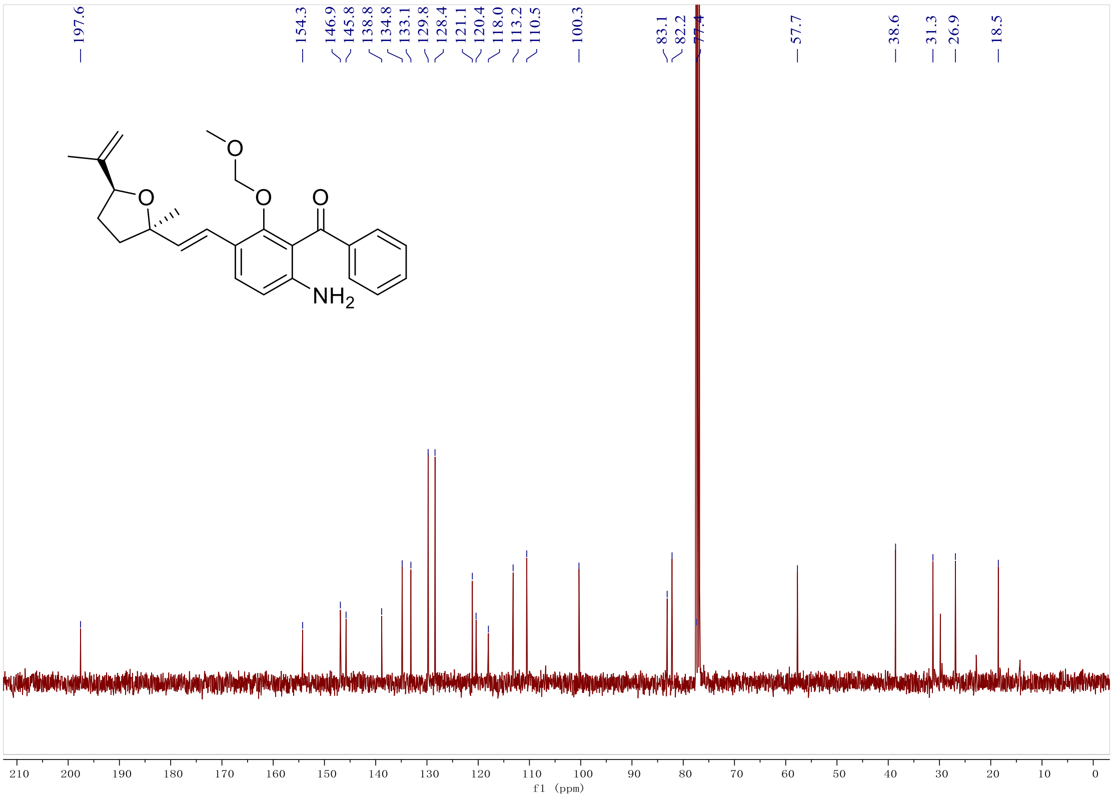

Supplementary Figure 39. ^{1}H (400 MHz, $CDCl_3$) and ^{13}C NMR (100 MHz, $CDCl_3$) spectra of compound **SI-13**

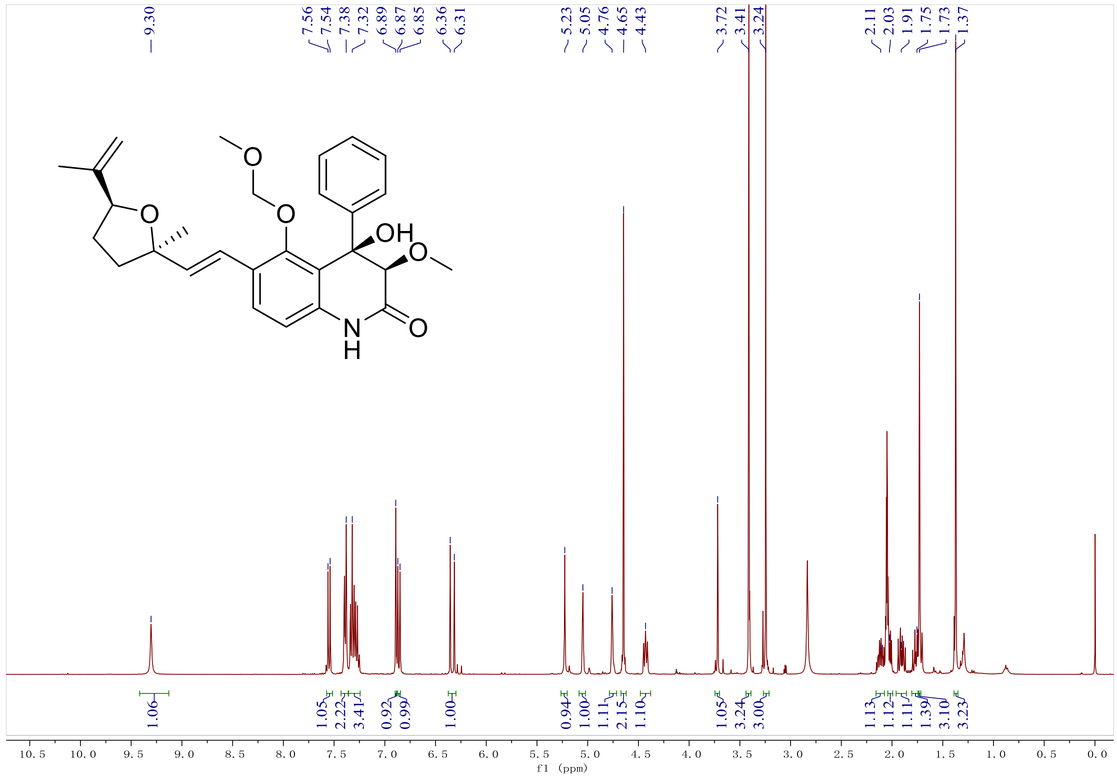

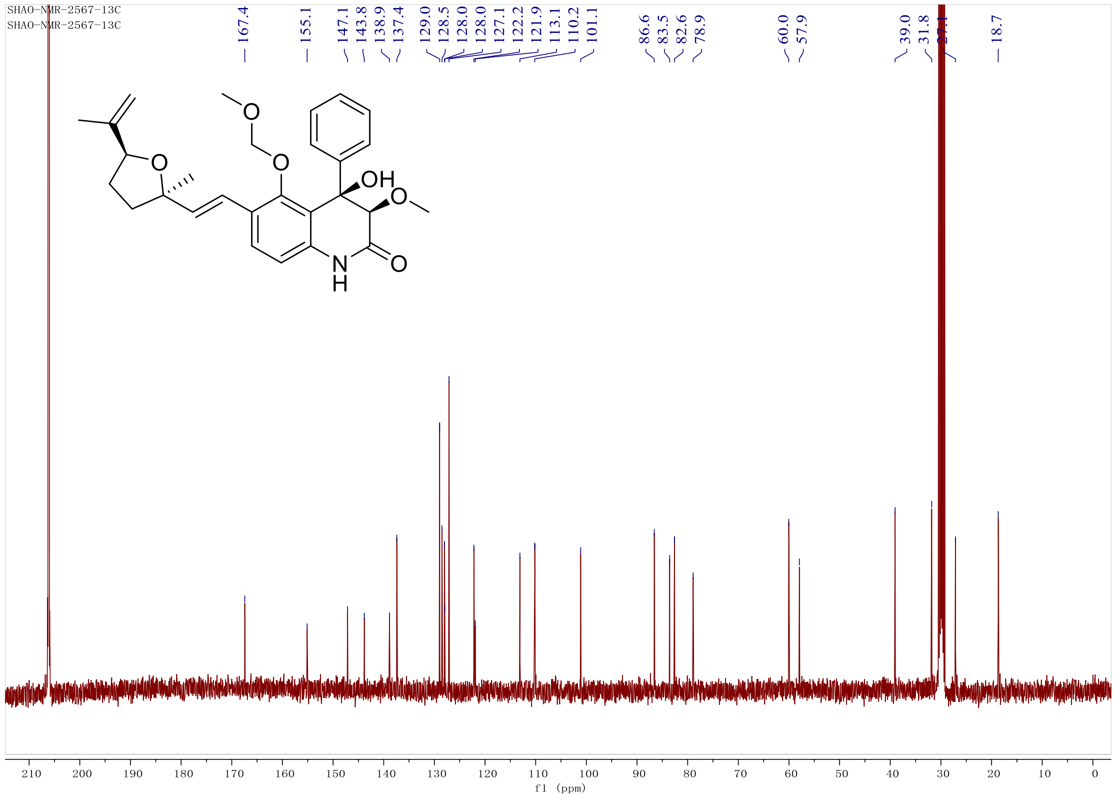

Supplementary Figure 40. ^{1}H NMR (400 MHz, acetone-d_6) and ^{13}C NMR (100 MHz, acetone-d_6) spectra of compound **24a**

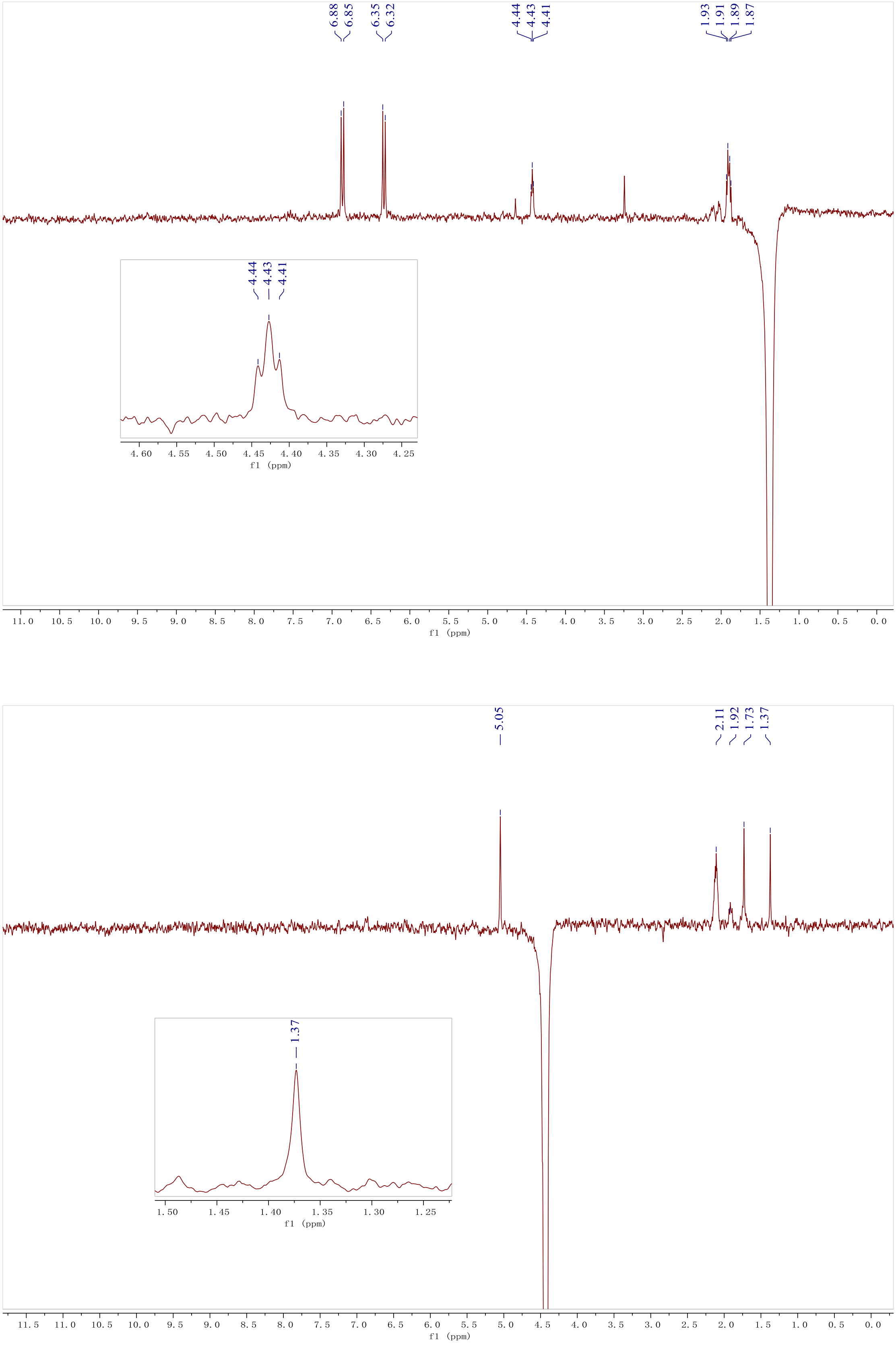

Supplementary Figure 41. NOE (400 MHz, acetone-d_6) spectra of compound **24a**

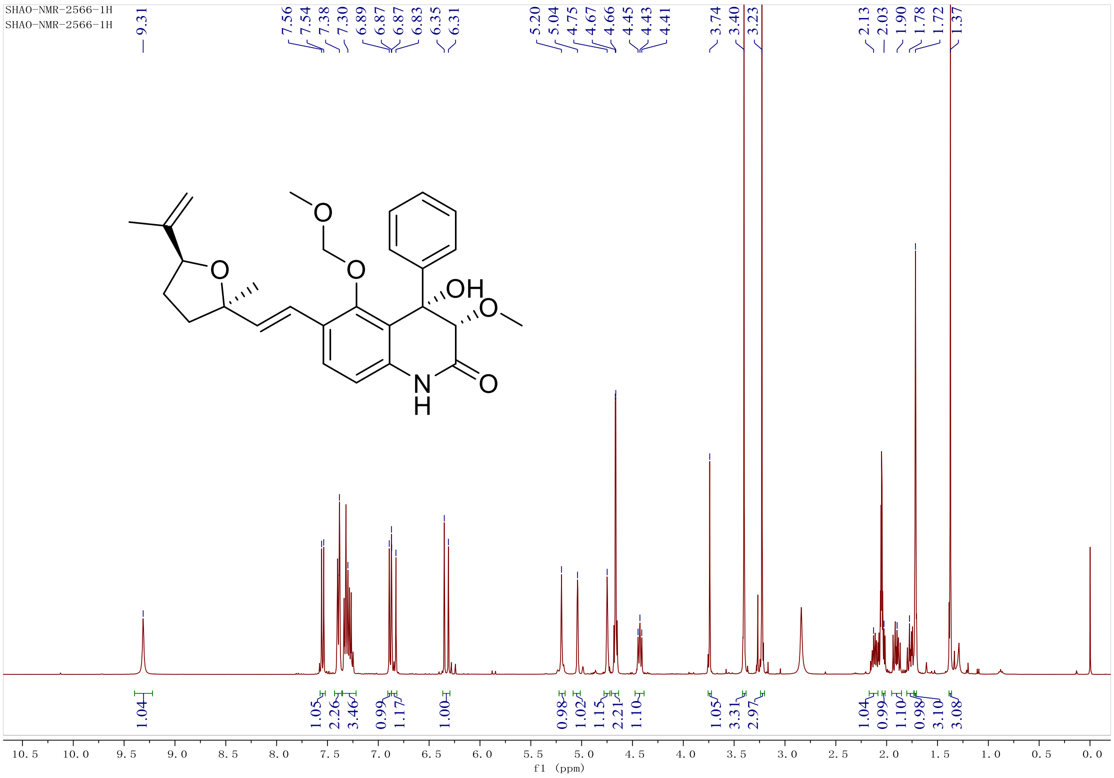

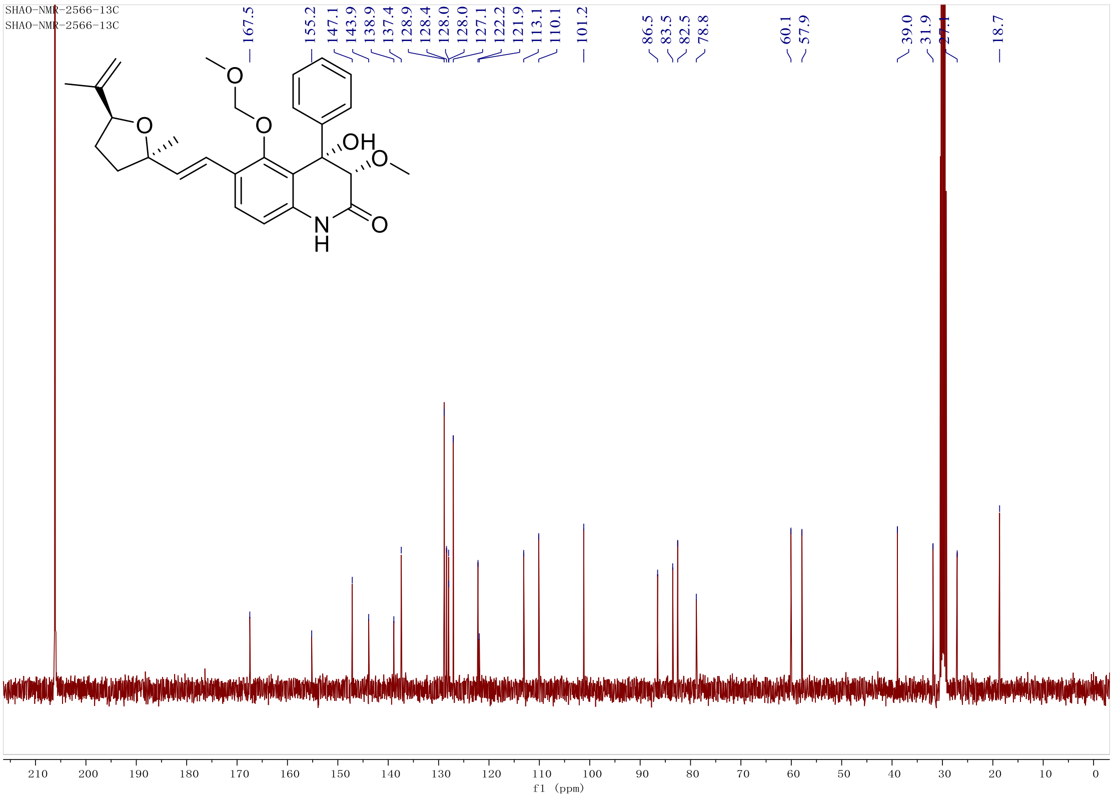

Supplementary Figure 42. ^{1}H NMR (400 MHz, acetone-d_6) and ^{13}C NMR (100 MHz, acetone-d_6) spectra of compound **24**

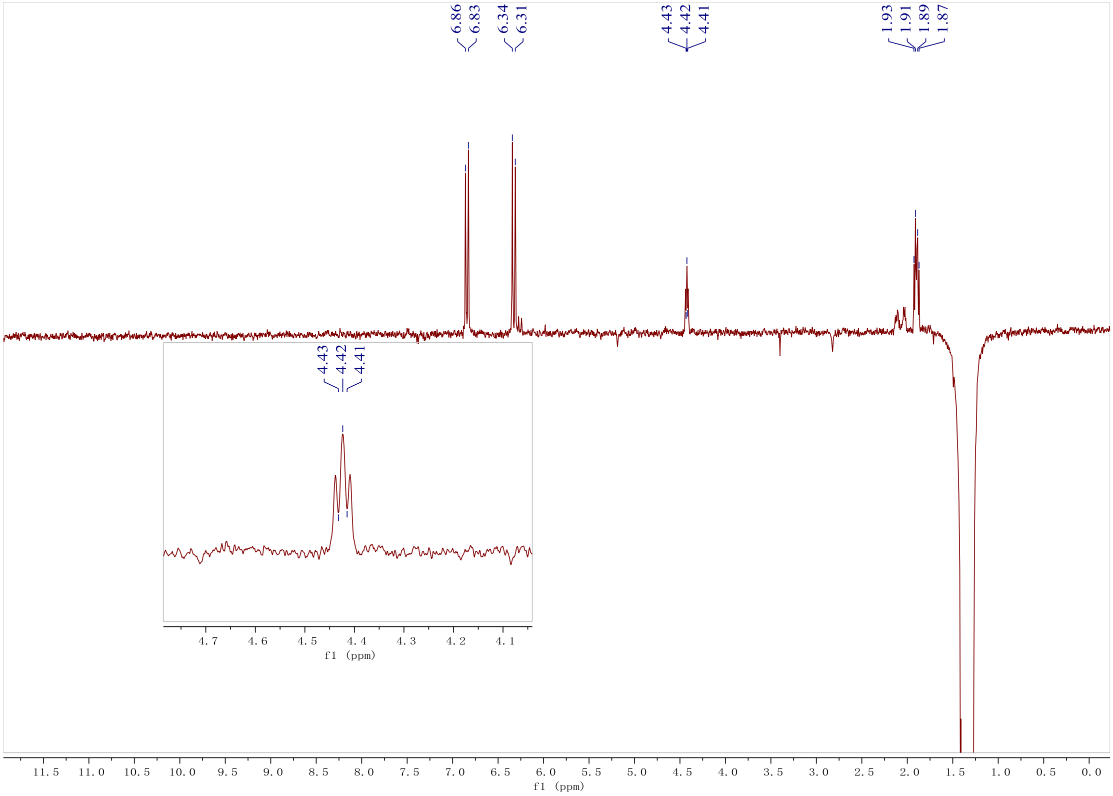

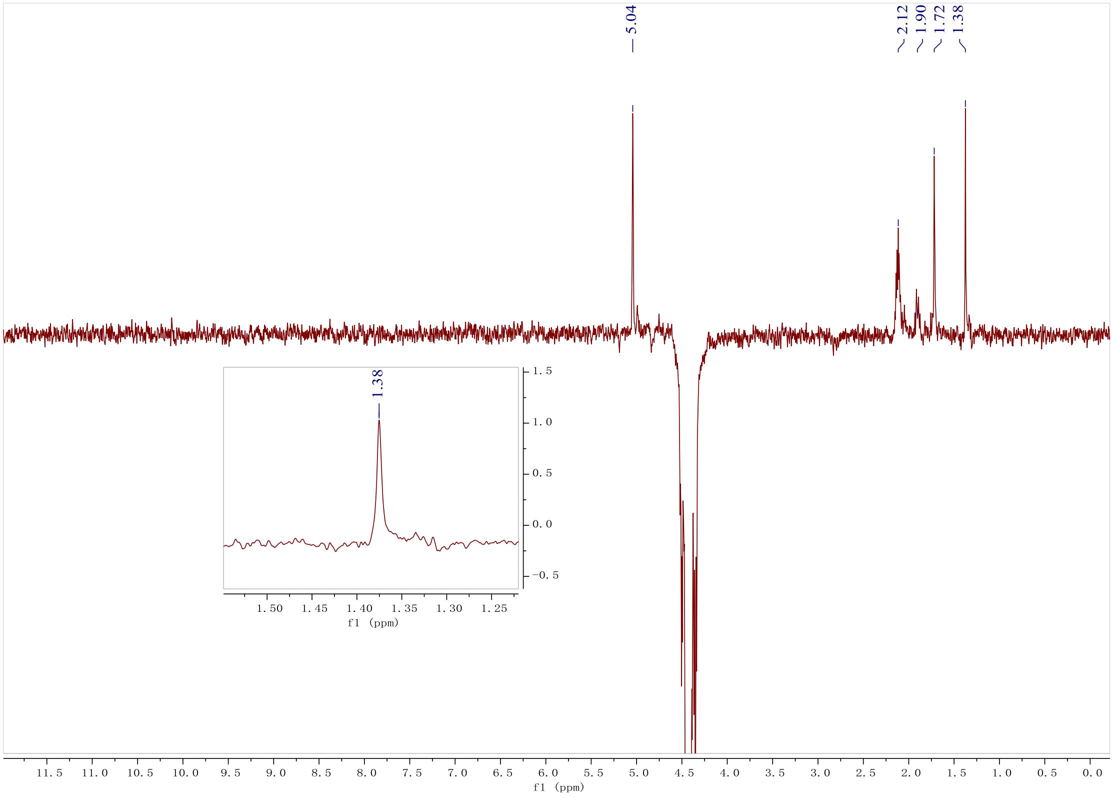

Supplementary Figure 43. NOE (400 MHz, acetone-d_6) spectra of compound **24**

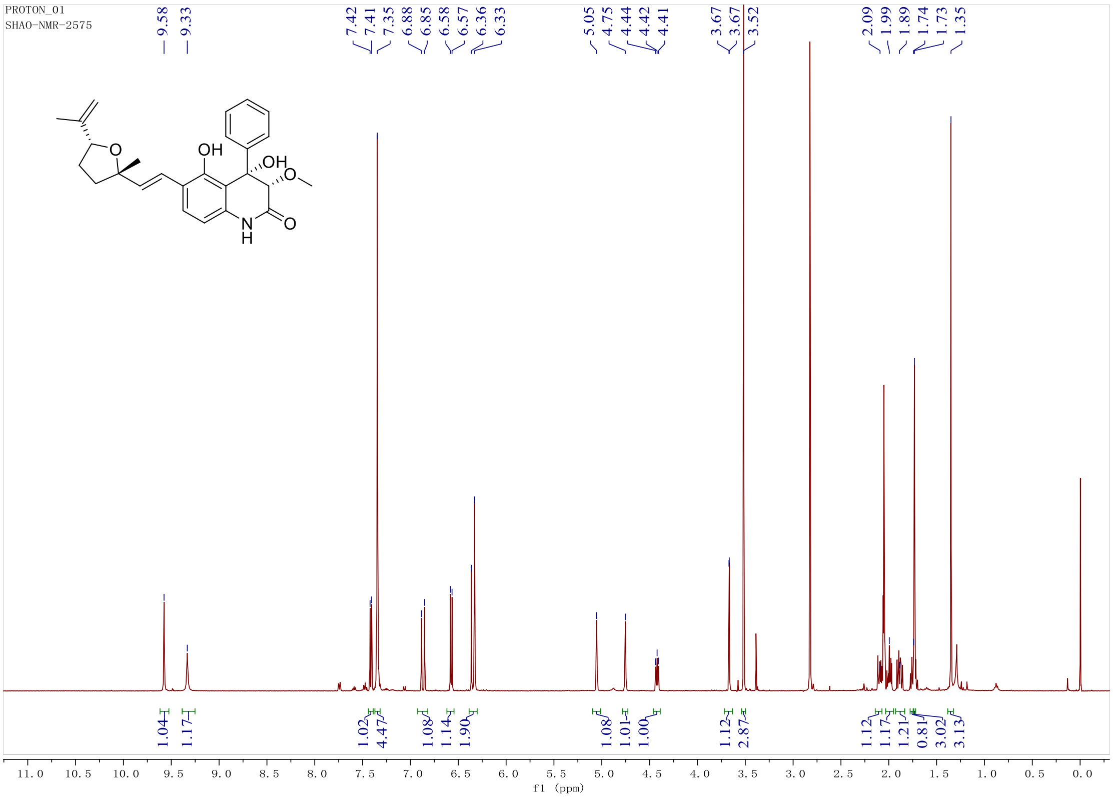

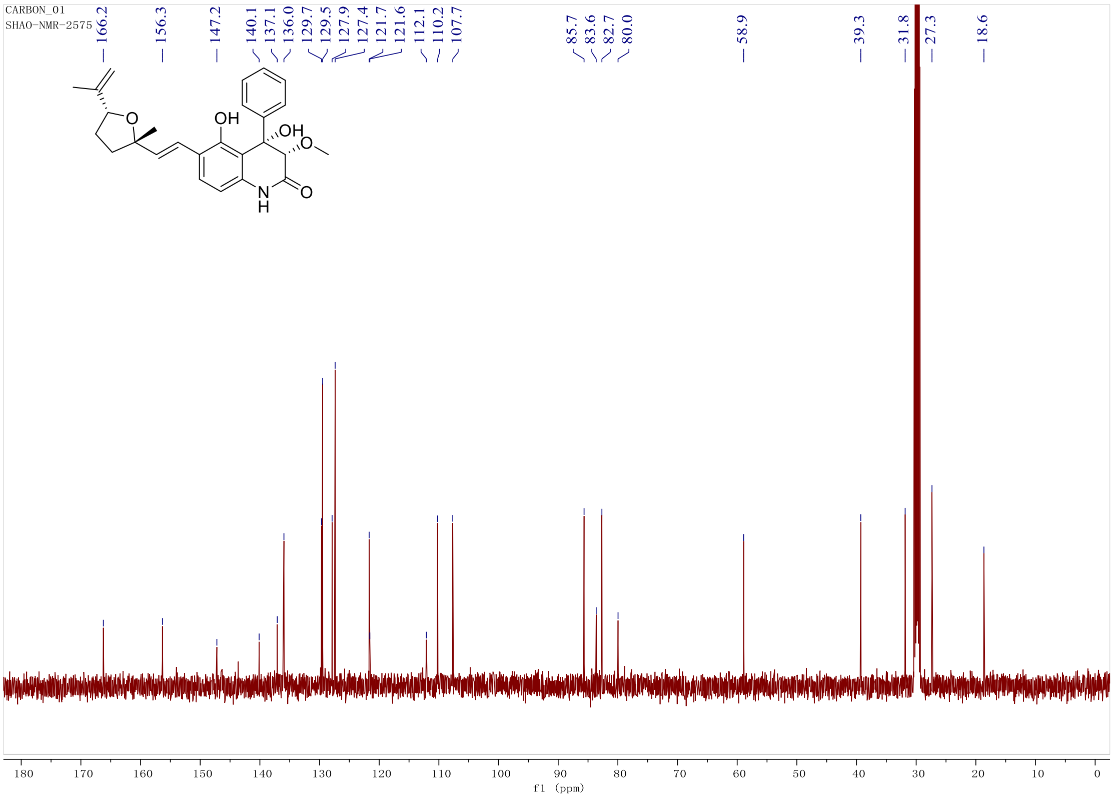

Supplementary Figure 44. ^{1}H NMR (500 MHz, acetone-d_6) and ^{13}C NMR (125 MHz, acetone-d_6) spectra of compound **25**

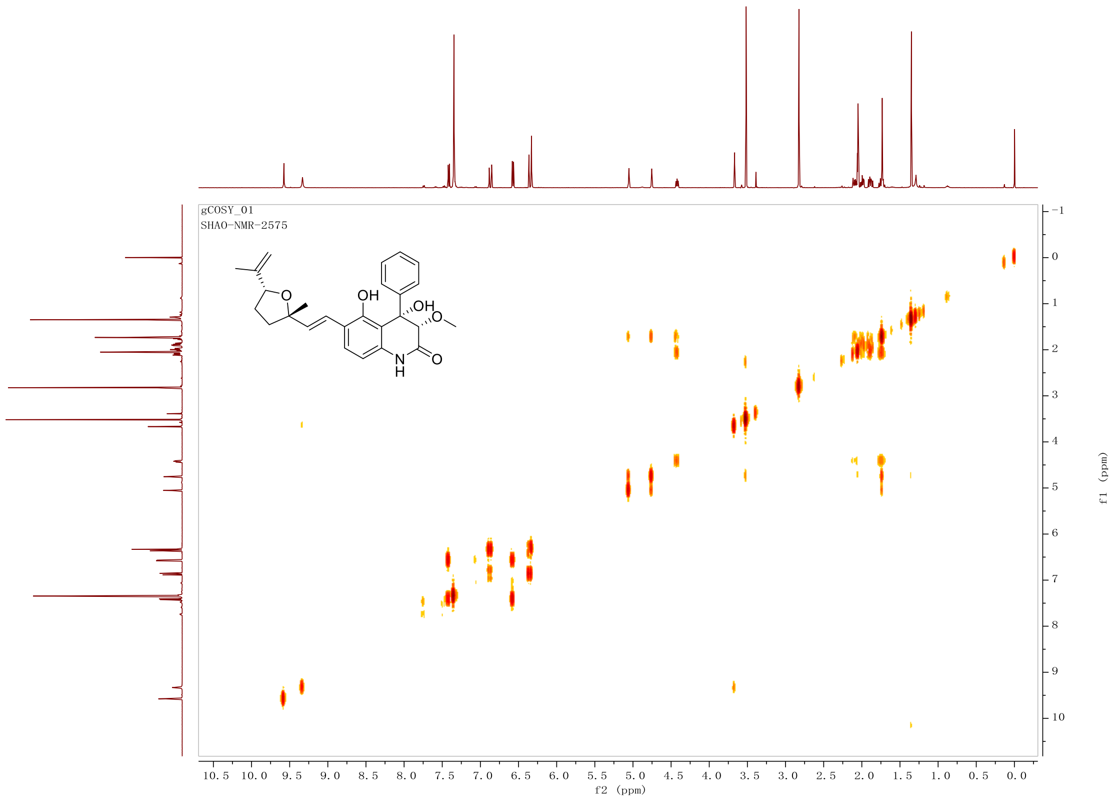

Supplementary Figure 45. ^{1}H-^{1}H COSY (500 MHz, acetone-d_6) spectra of compound **25**

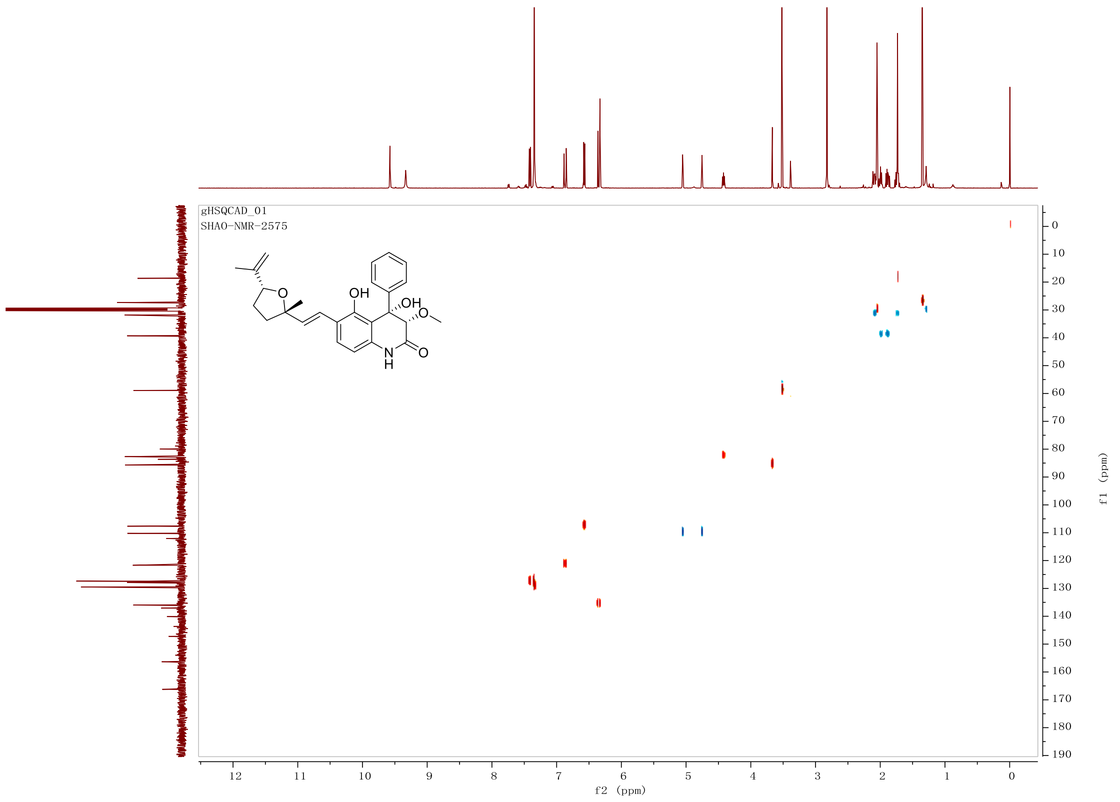

Supplementary Figure 46. HSQC (500 MHz, acetone-d_6) spectra of compound **25**

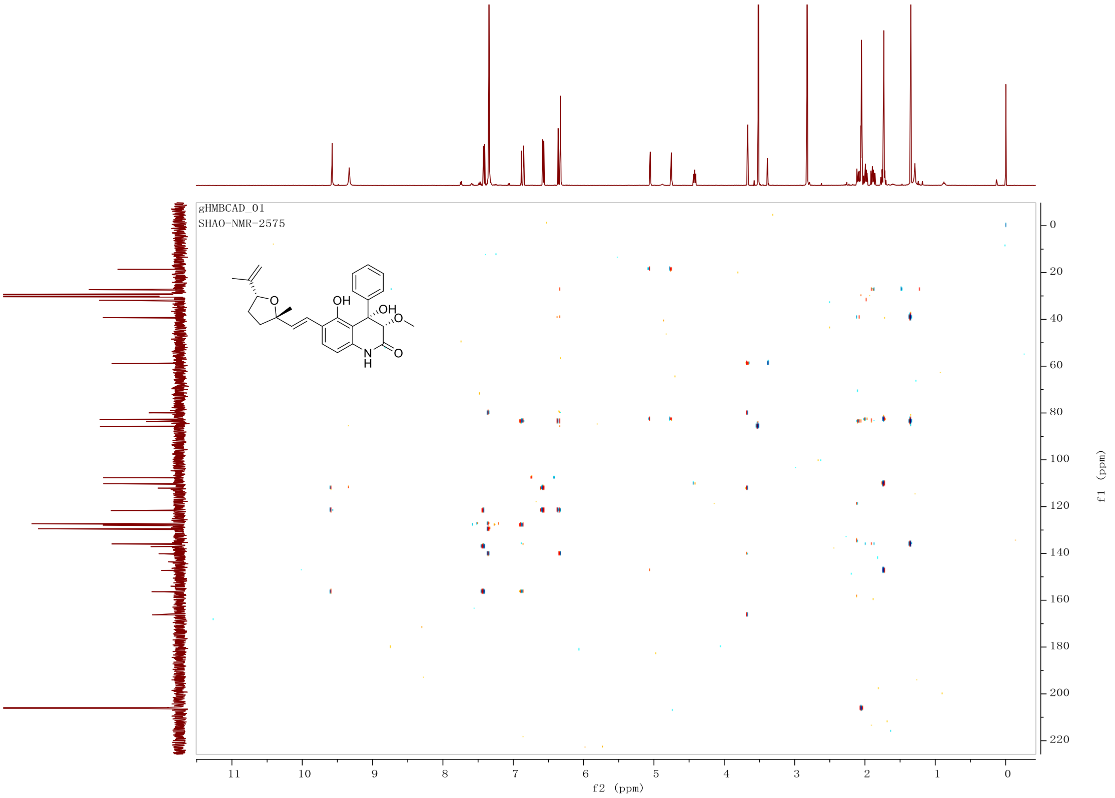

Supplementary Figure 47. HMBC (500 MHz, acetone-d_6) spectra of compound **25**

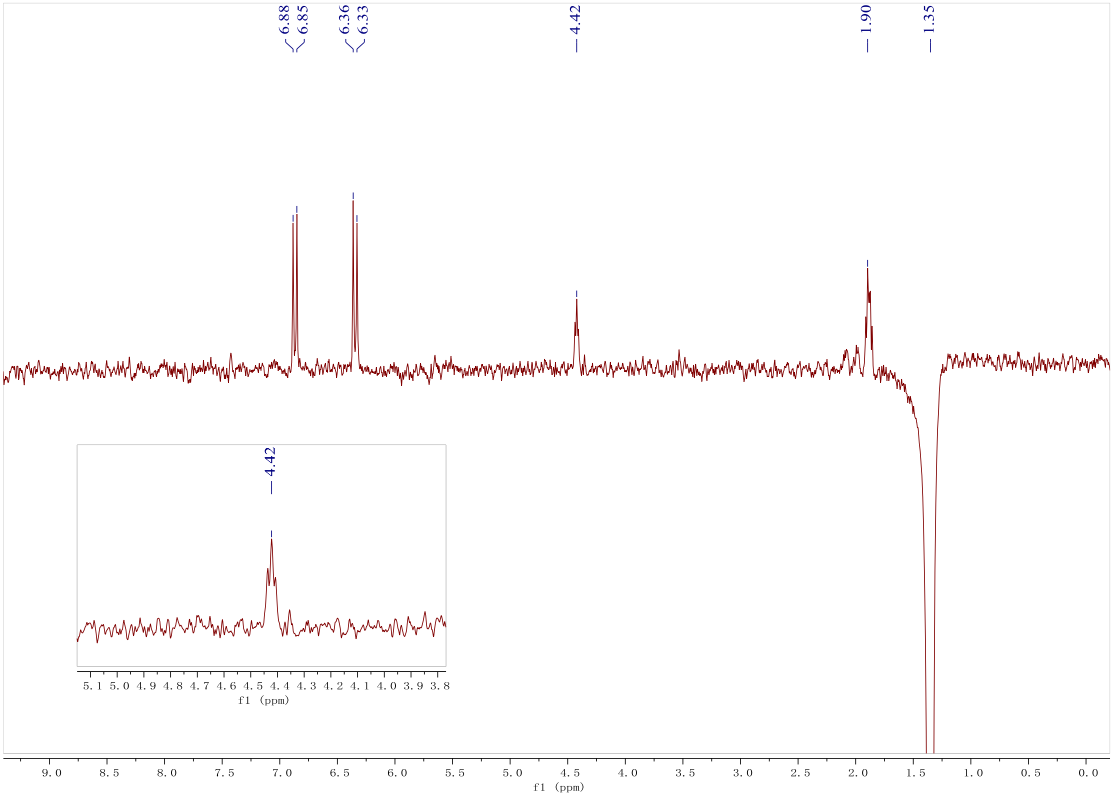

Supplementary Figure 48. NOE (500 MHz, acetone-d_6) spectra of compound **25**

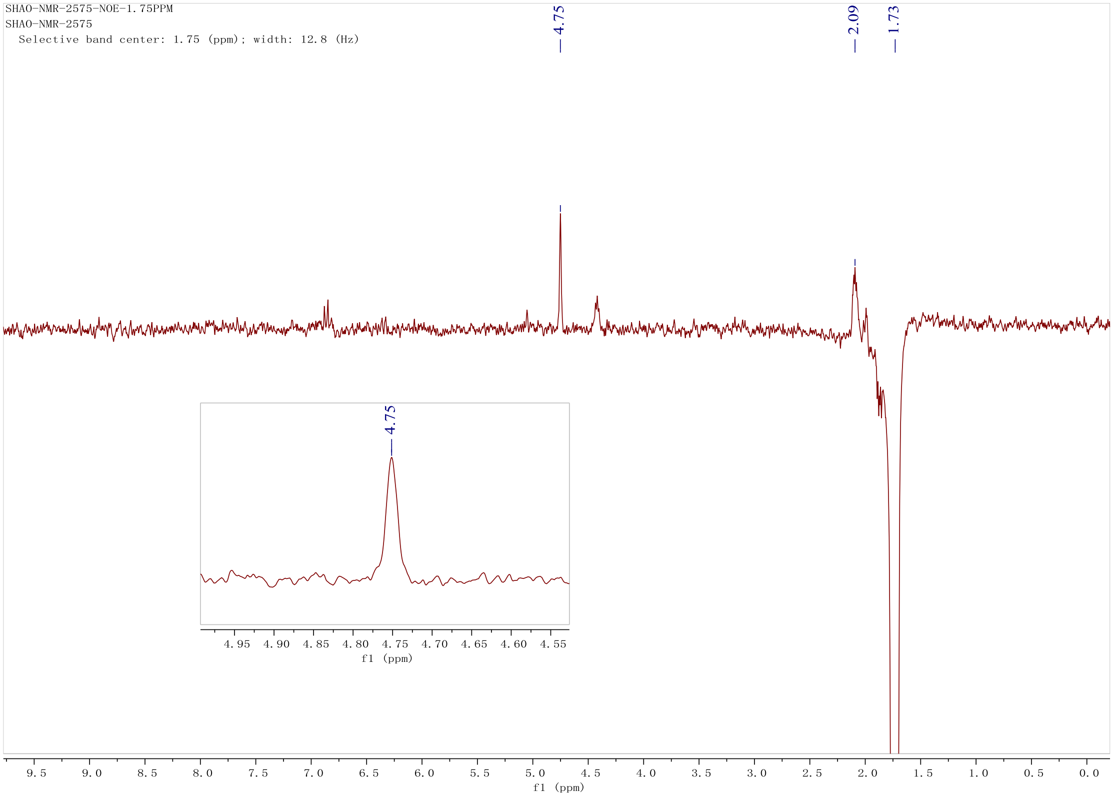

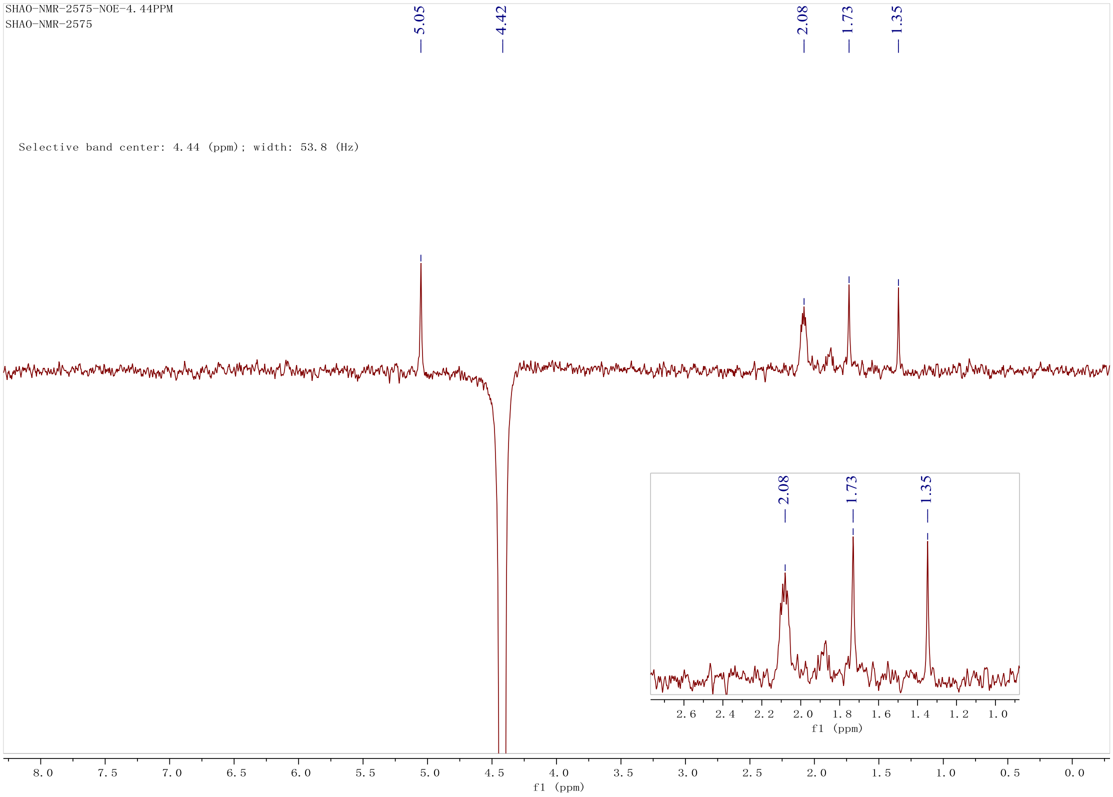

Supplementary Figure 49. NOE (500 MHz, acetone-d_6) spectra of compound **25**

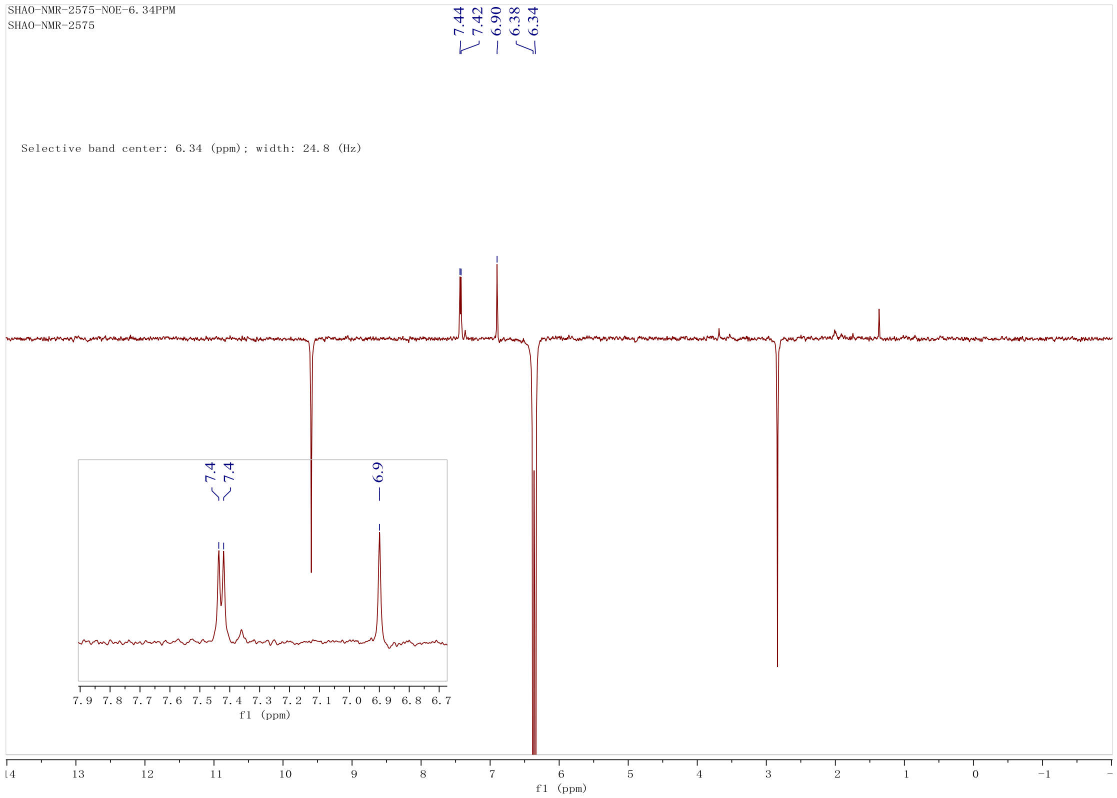

Supplementary Figure 50. NOE (500 MHz, acetone-d_6) spectra of compound **25**

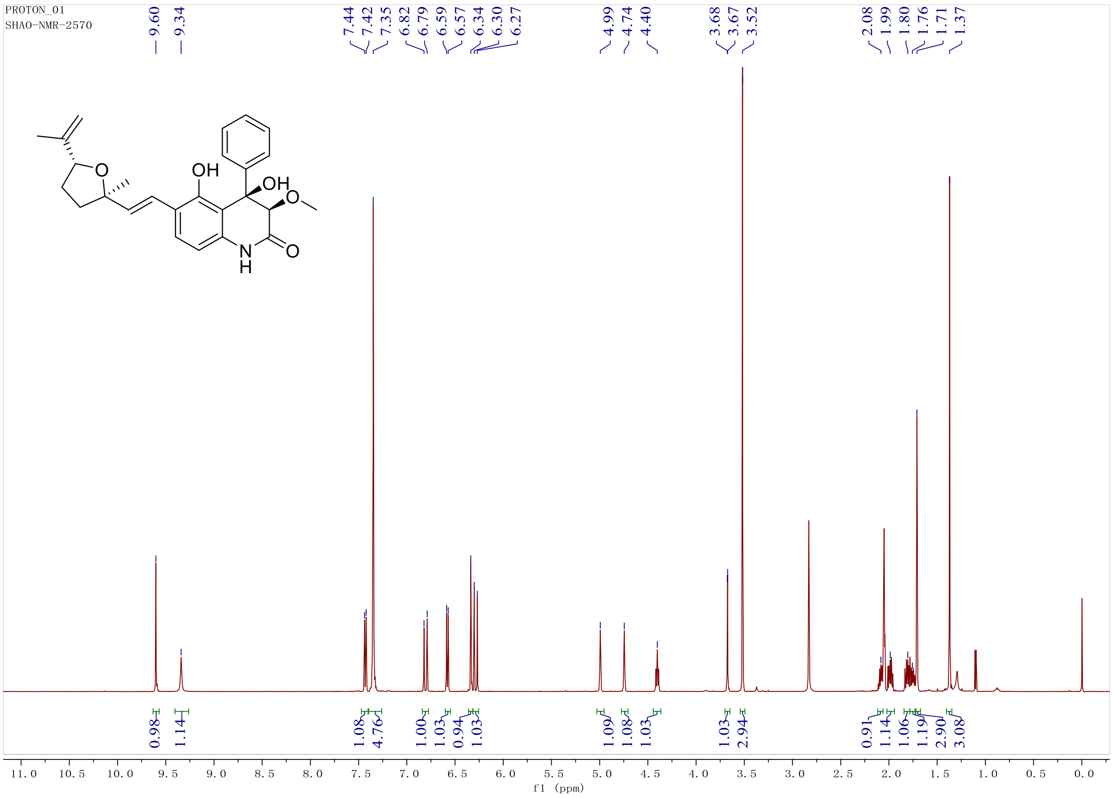

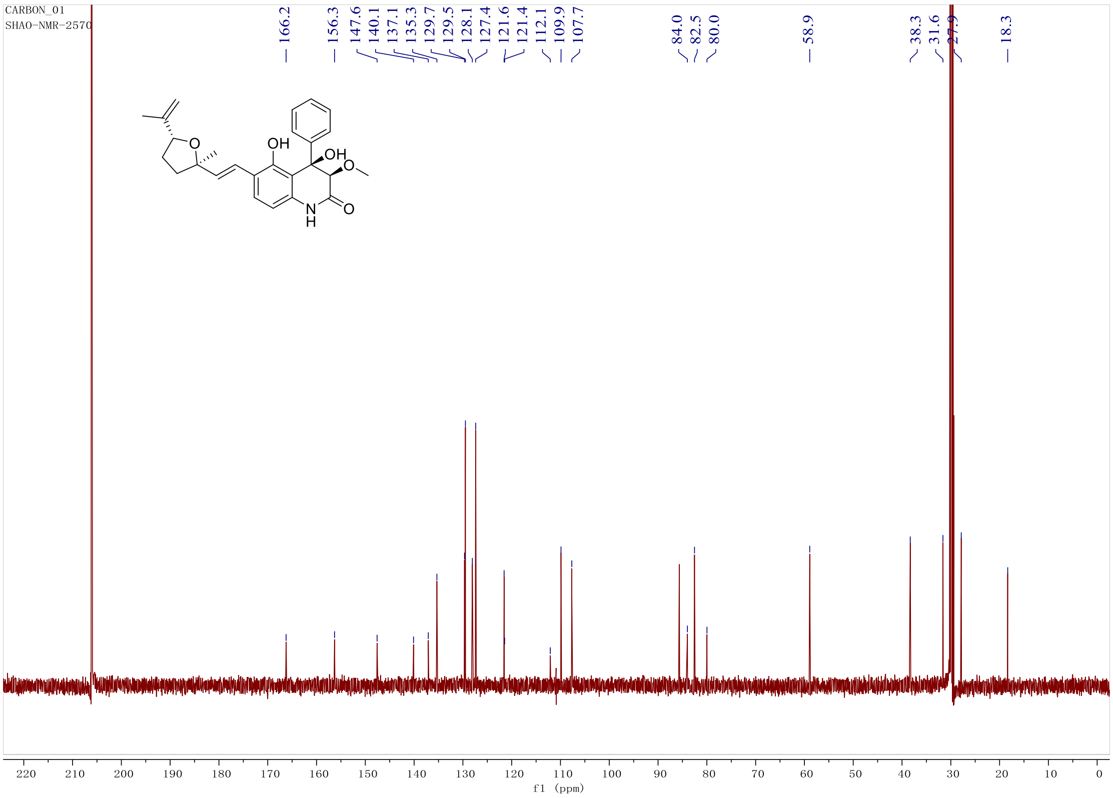

Supplementary Figure 51. ^{1}H NMR (500 MHz, acetone-d_6) and ^{13}C NMR (125 MHz, acetone-d_6) spectra of compound **28**

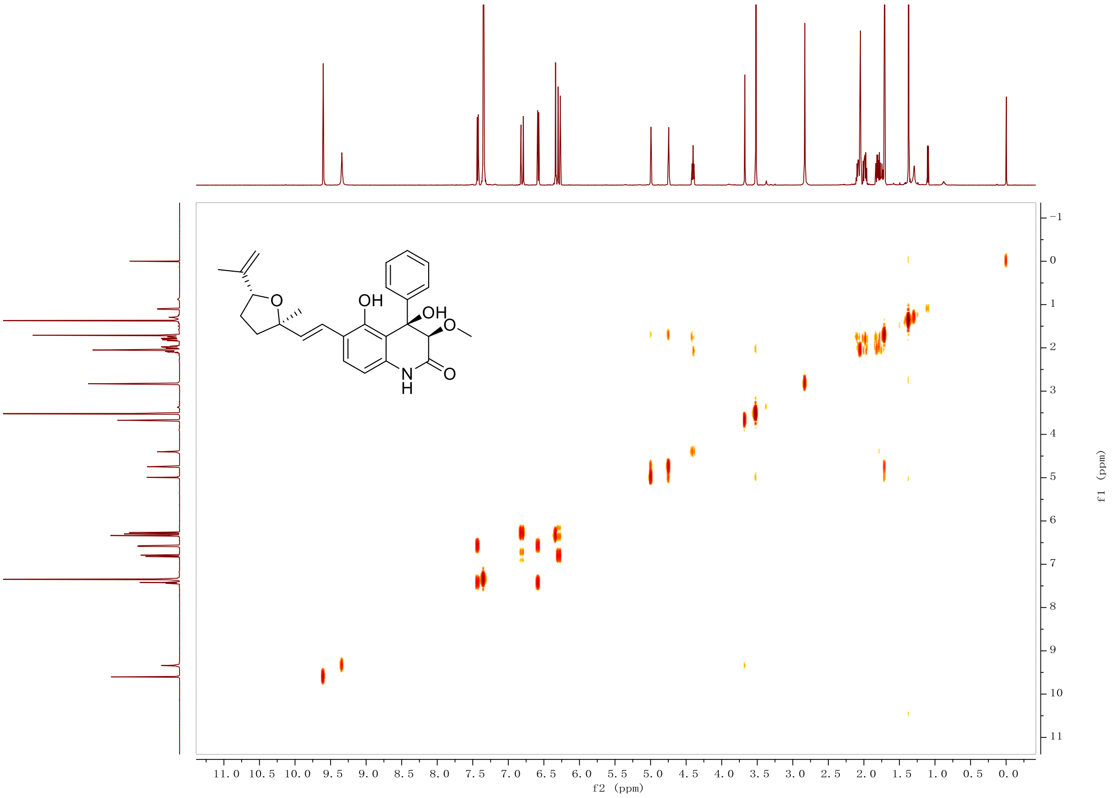

Supplementary Figure 52. ^{1}H-^{1}H COSY (500 MHz, acetone-d_6) spectra of compound **28**

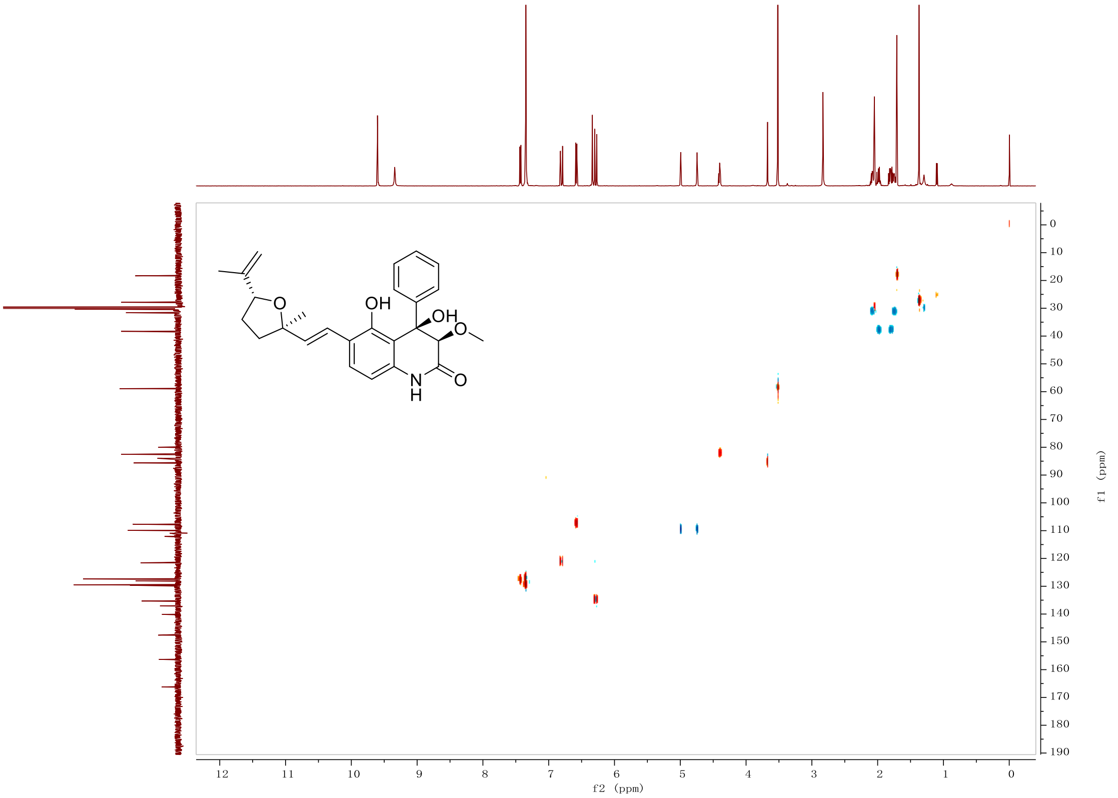

Supplementary Figure 53. HSQC (500 MHz, acetone-d_6) spectra of compound **28**

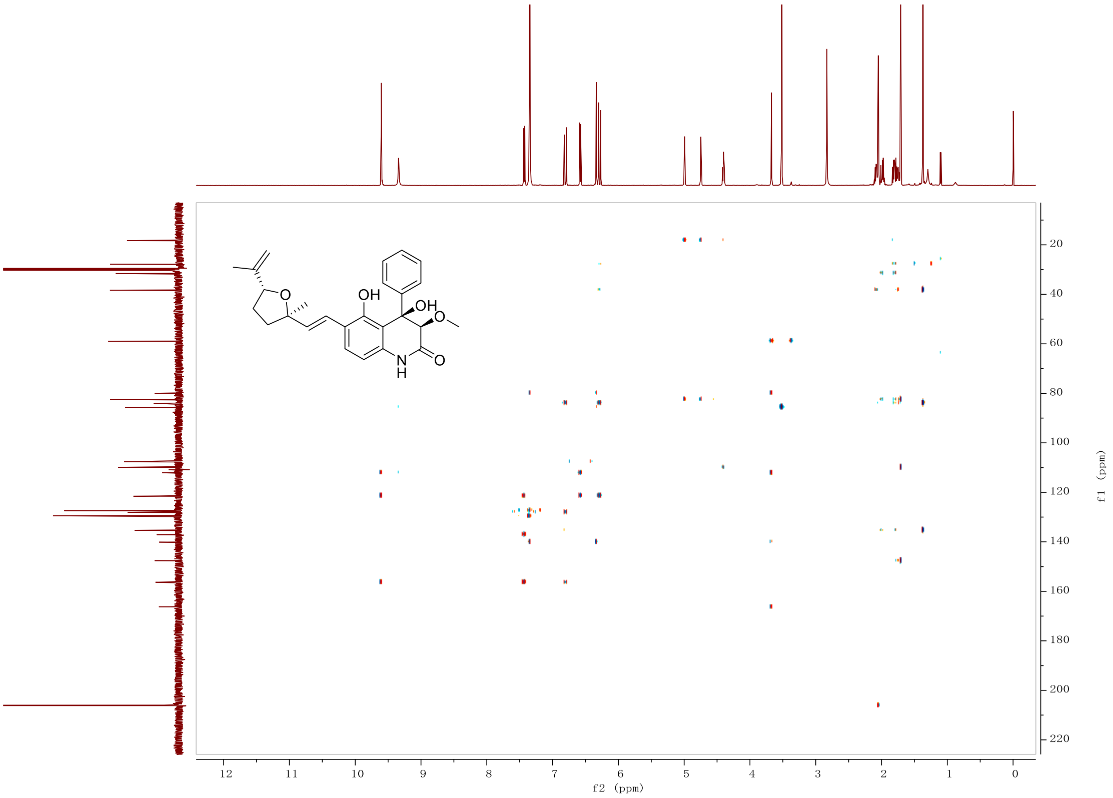

Supplementary Figure 54. HMBC (500 MHz, acetone-d_6) spectra of compound **28**

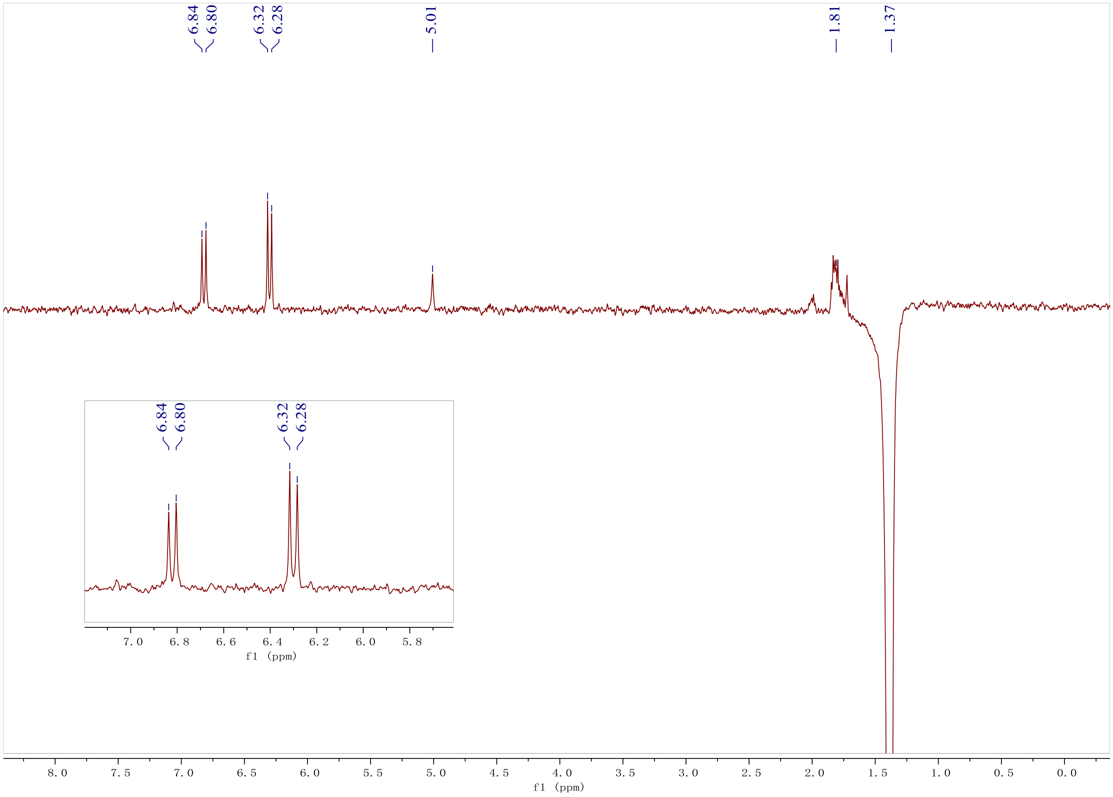

Supplementary Figure 55. NOE (500 MHz, acetone-d_6) spectra of compound **28**

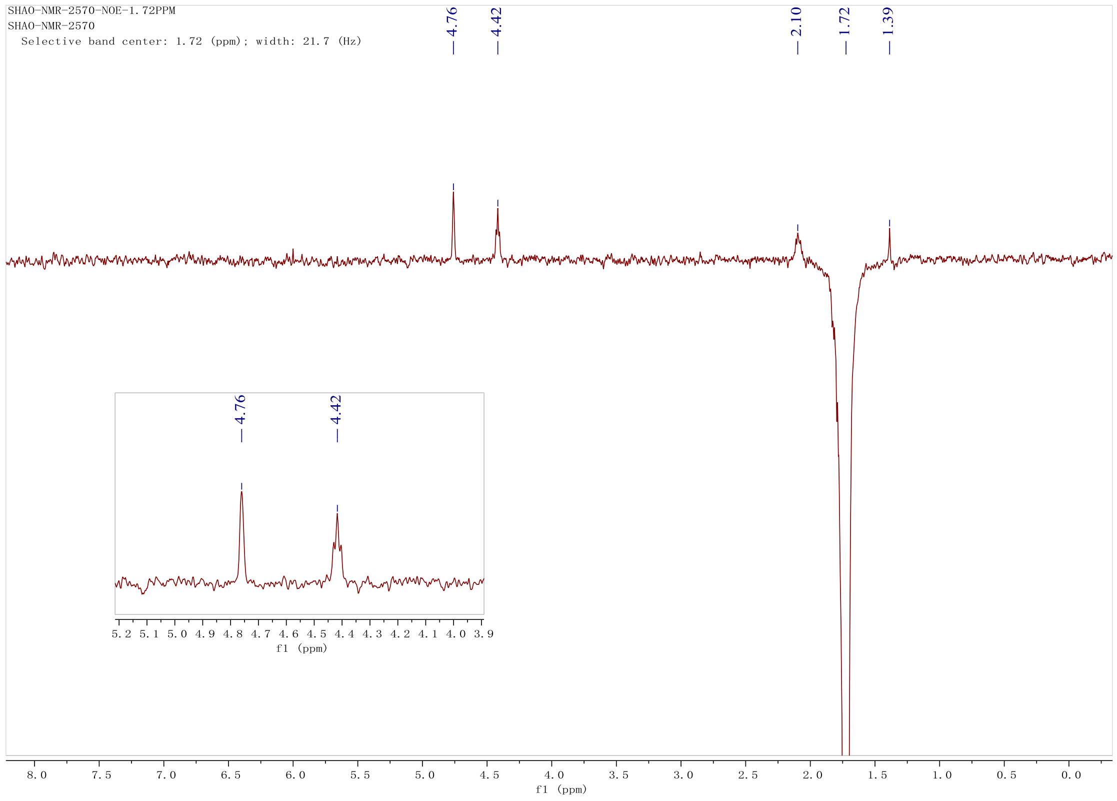

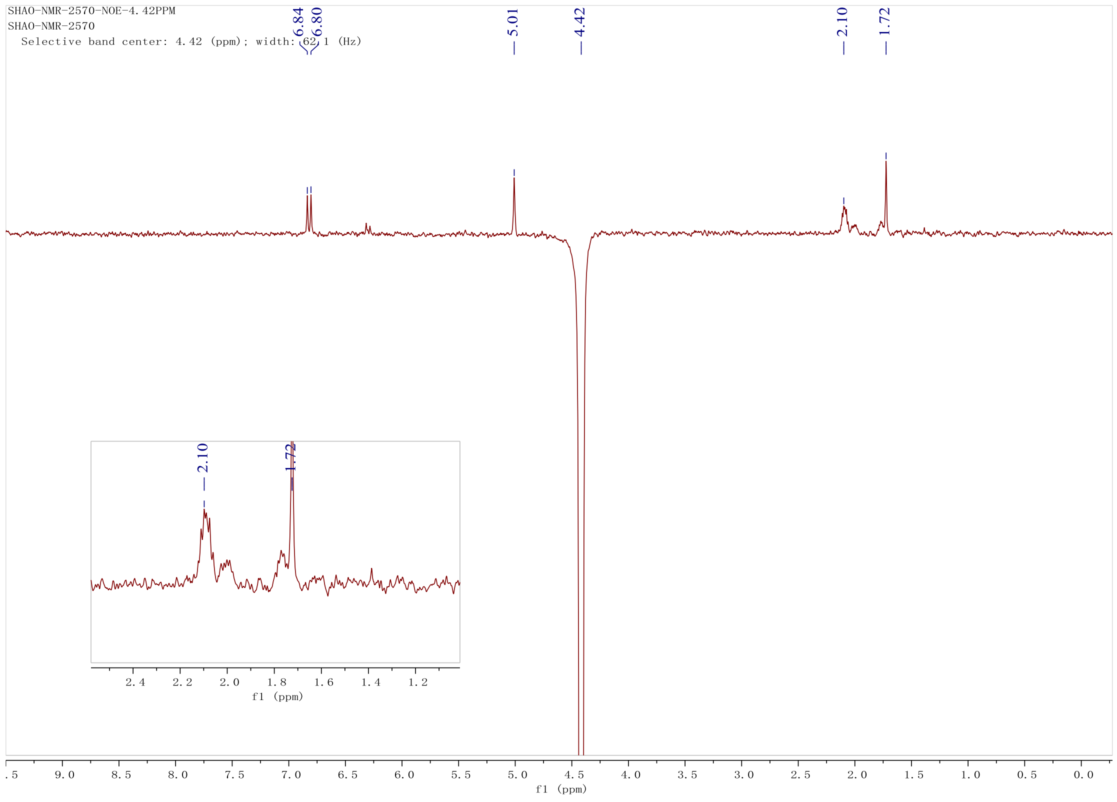

Supplementary Figure 56. NOE (500 MHz, acetone-d_6) spectra of compound **28**

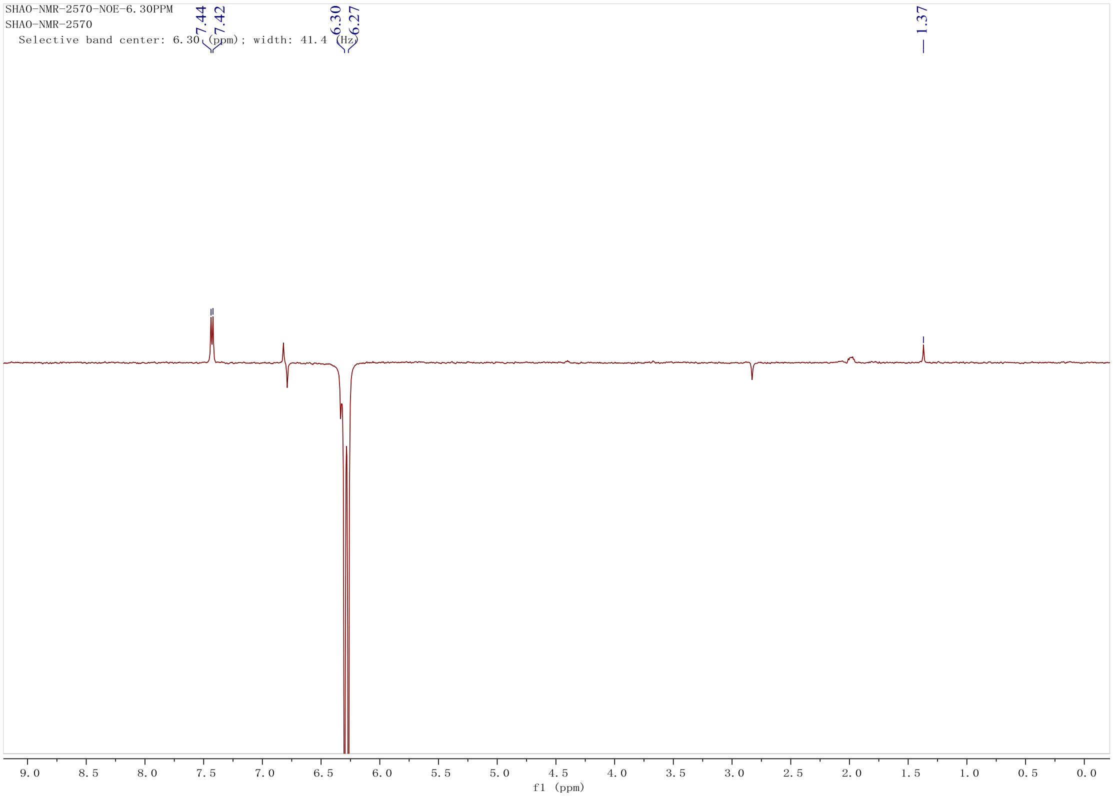

Supplementary Figure 57. NOE (500 MHz, acetone-d_6) spectra of compound **28**

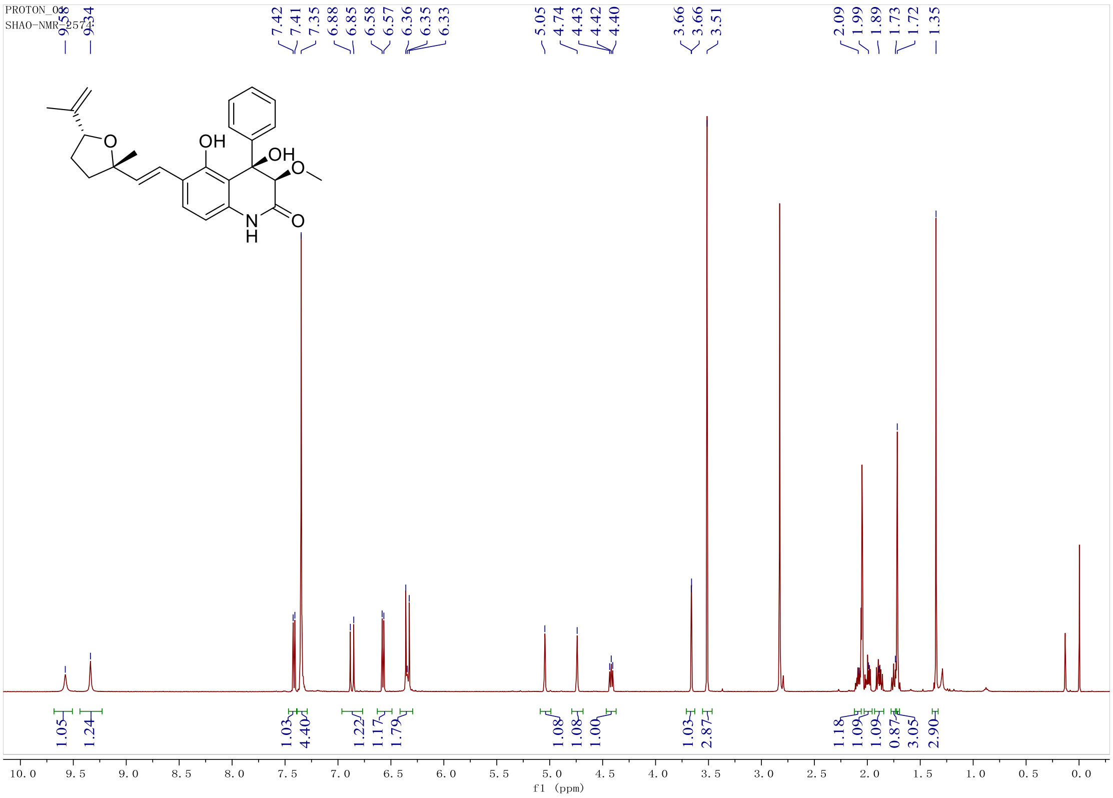

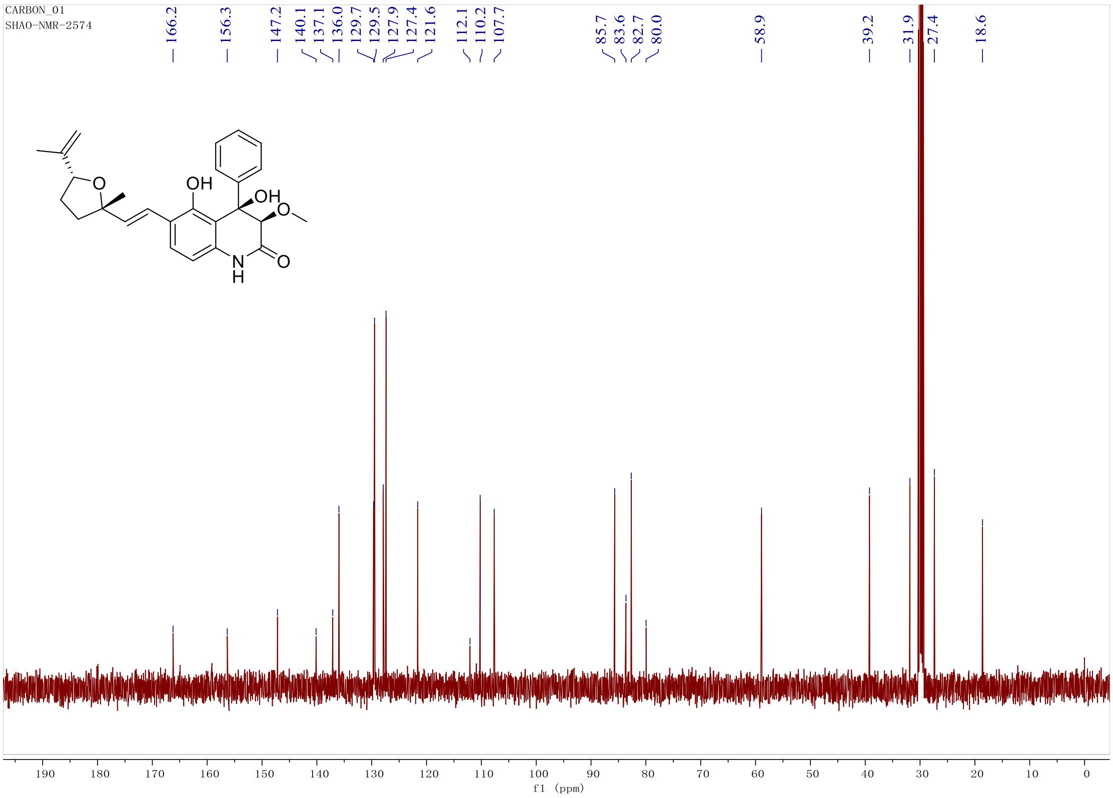

Supplementary Figure 58. ^{1}H NMR (500 MHz, acetone-d_6) and ^{13}C NMR (125 MHz, acetone-d_6) spectra of compound **29**

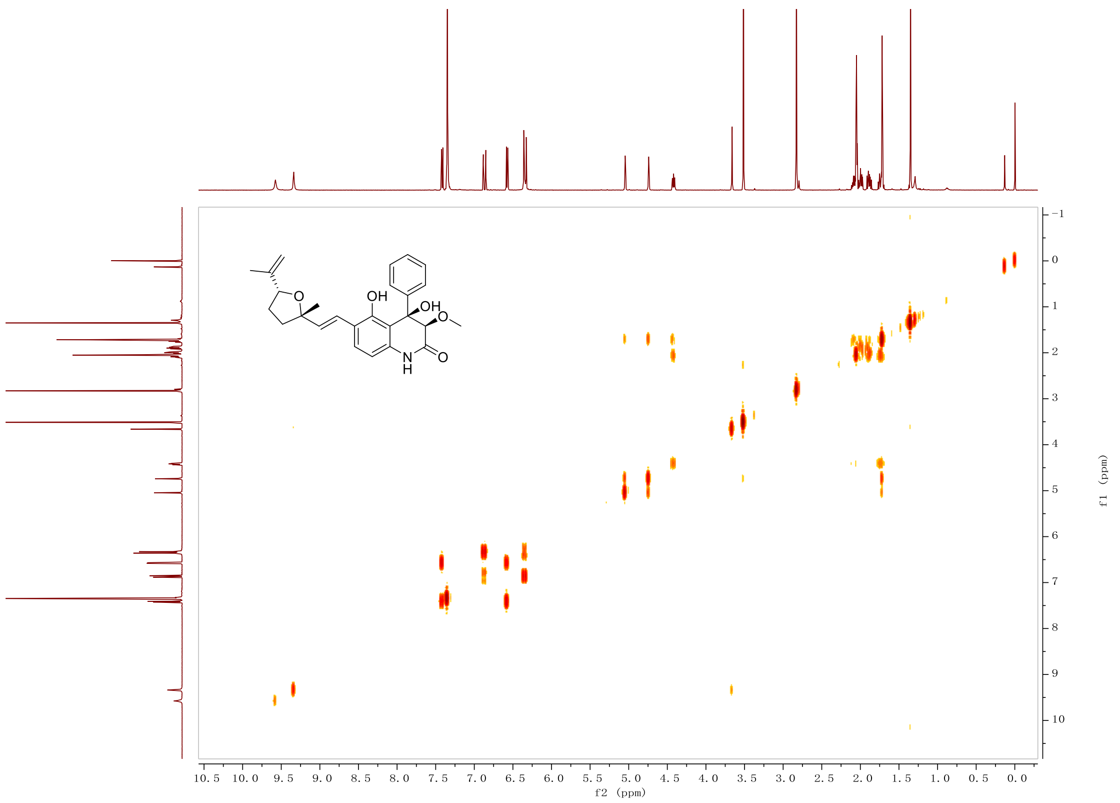

Supplementary Figure 59. ^{1}H-^{1}H COSY (500 MHz, acetone-d_6) spectra of compound **29**

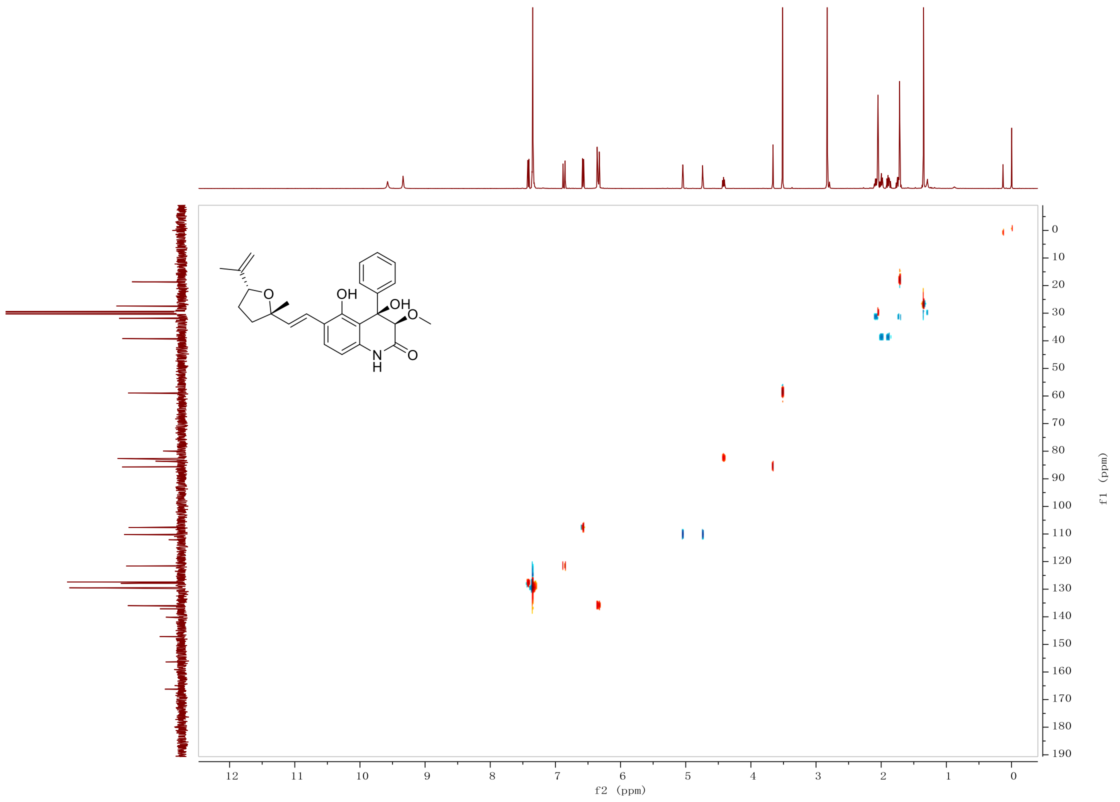

Supplementary Figure 60. HSQC (500 MHz, acetone-d_6) spectra of compound **29**

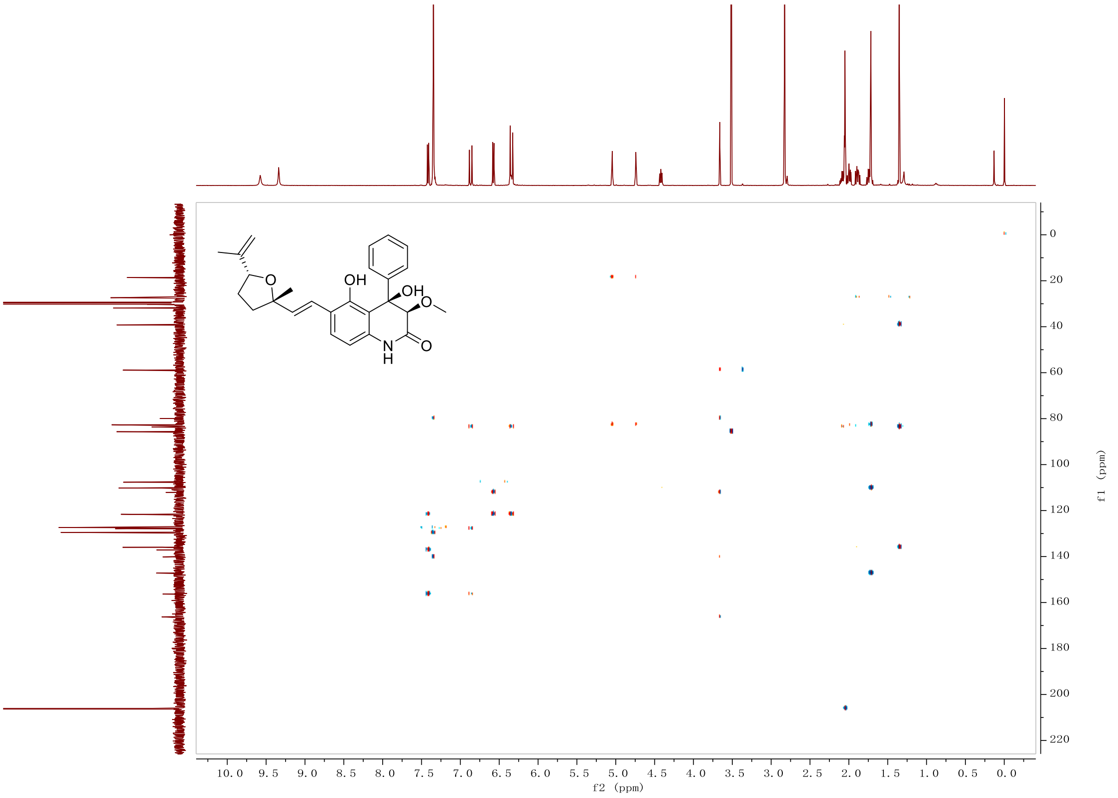

Supplementary Figure 61. HMBC (500 MHz, acetone-d_6) spectra of compound **29**

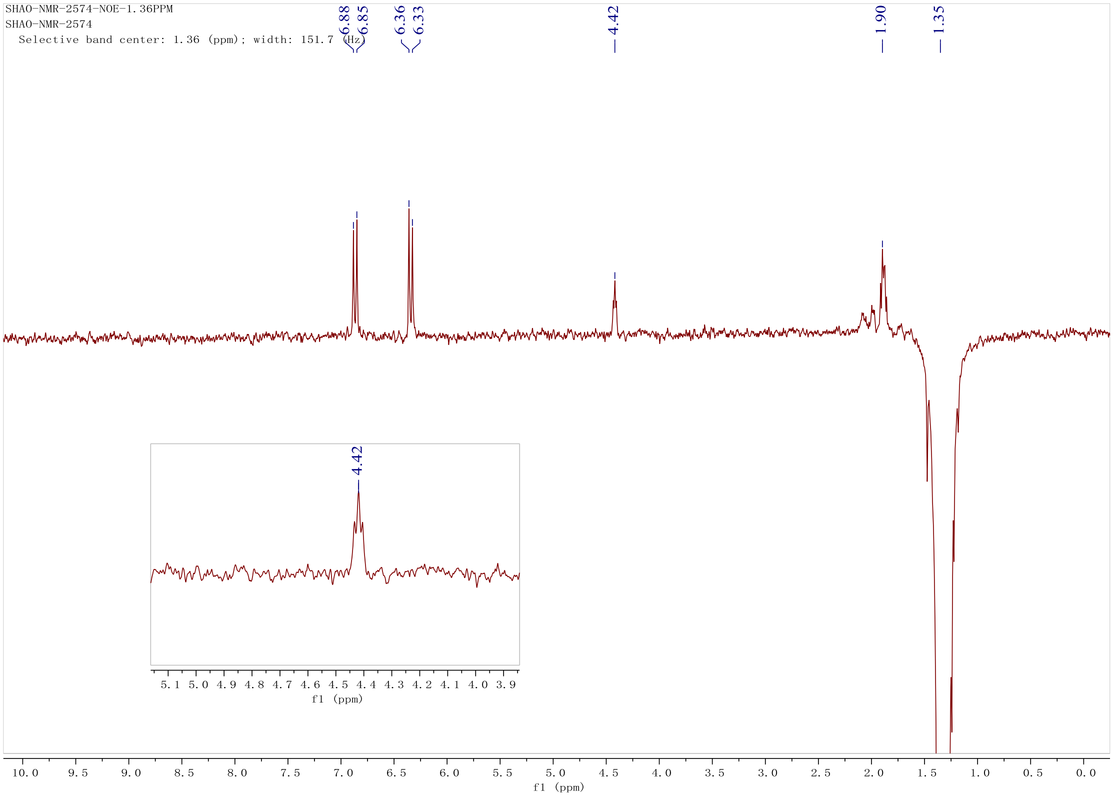

Supplementary Figure 62. NOE (500 MHz, acetone-d_6) spectra of compound **29**

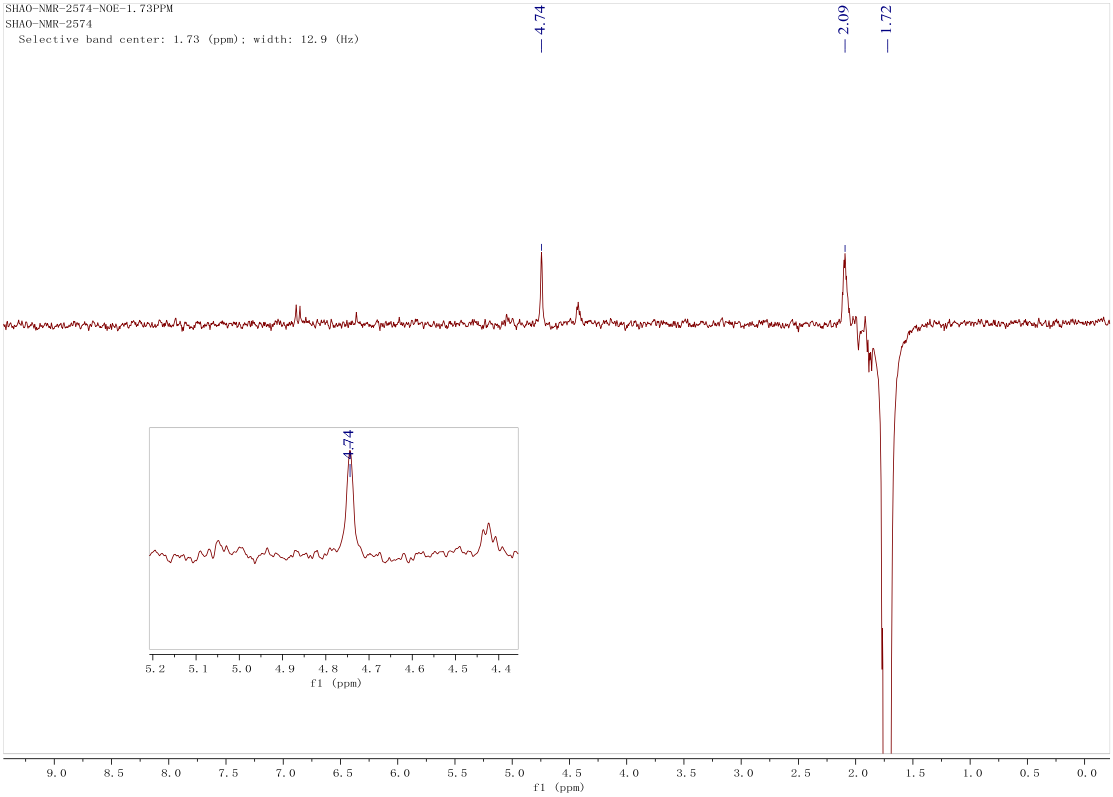

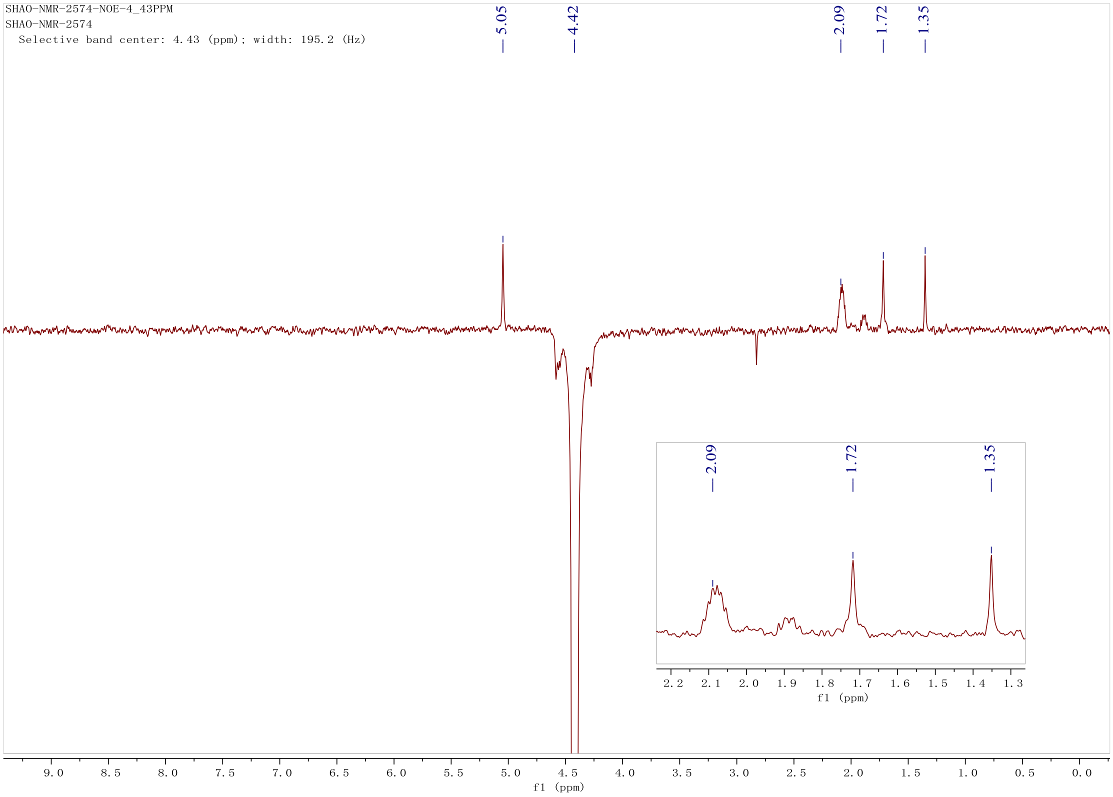

Supplementary Figure 63. NOE (500 MHz, acetone-d_6) spectra of compound **29**

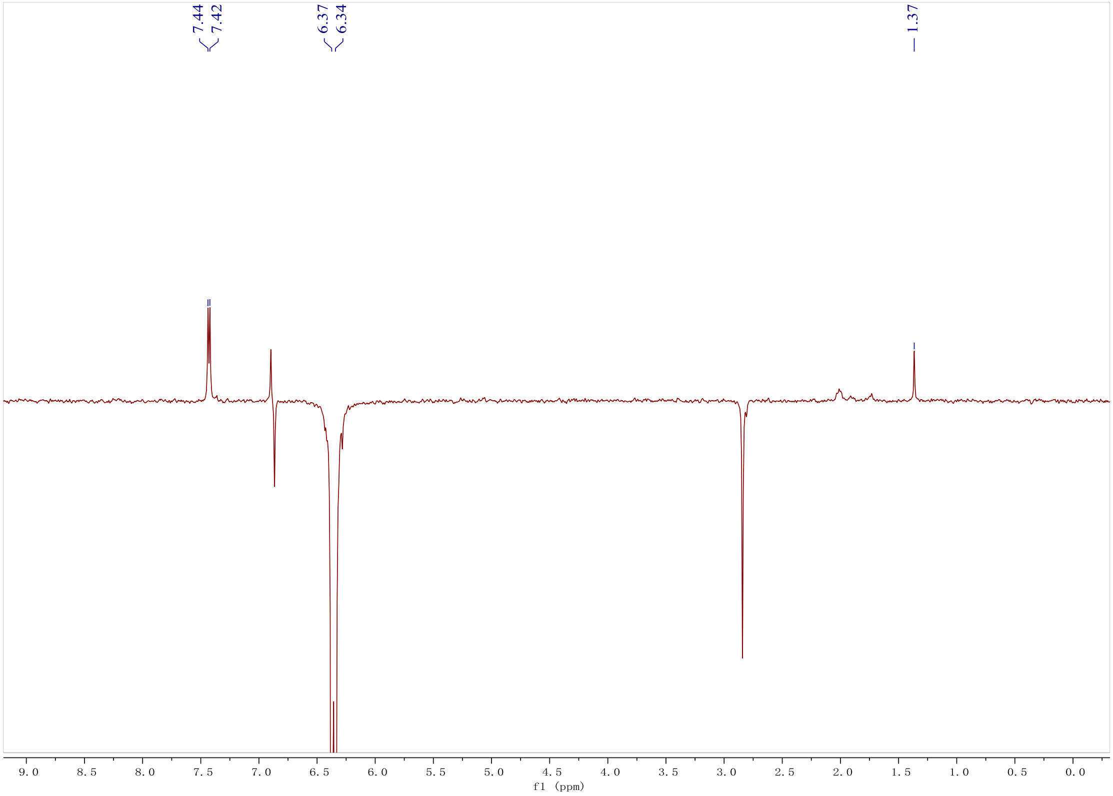

Supplementary Figure 64. NOE (500 MHz, acetone-d_6) spectra of compound **29**

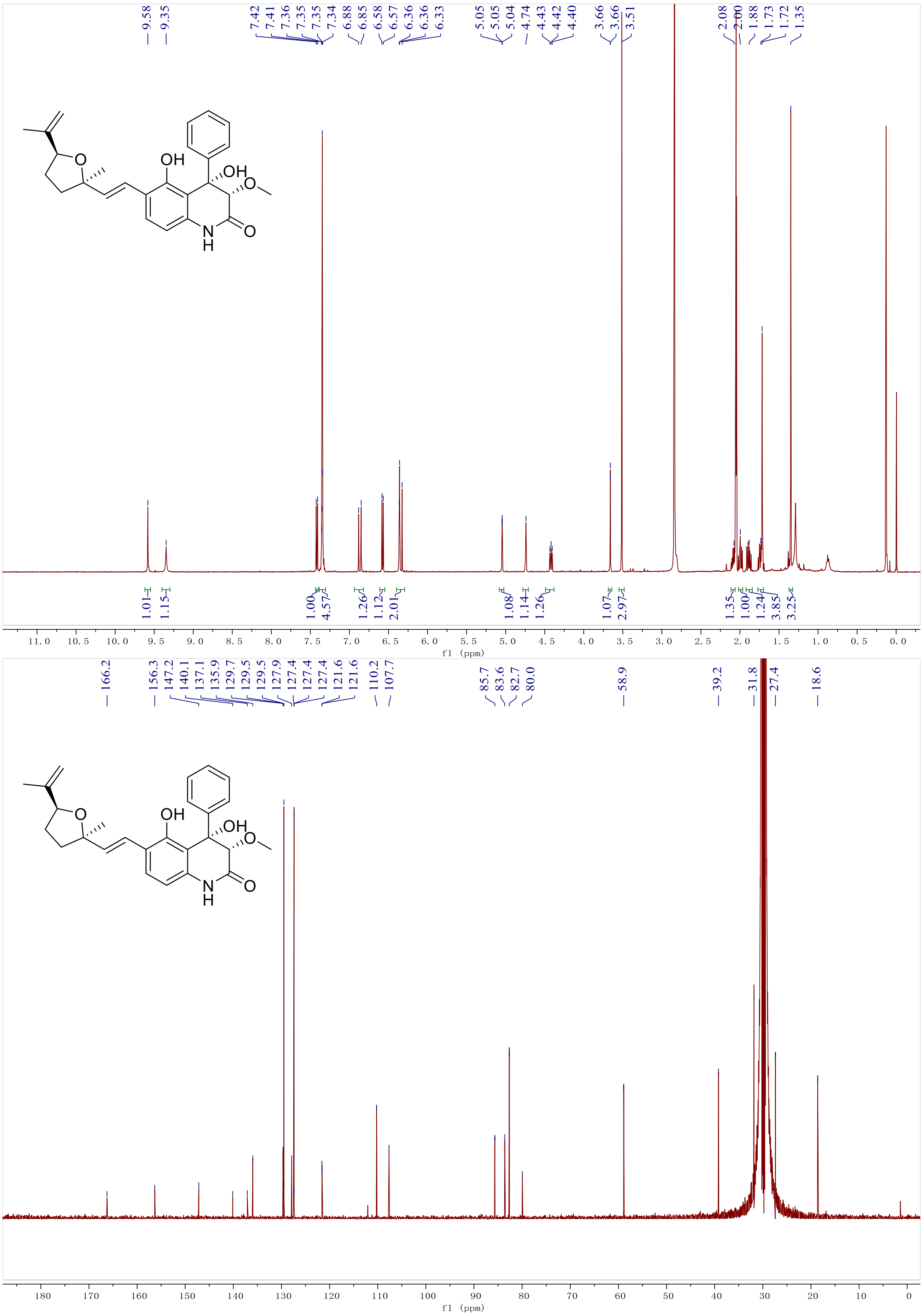

Supplementary Figure 65. ^{1}H NMR (500 MHz, acetone-d_6) and ^{13}C NMR (125 MHz, acetone-d_6) spectra of compound **27**

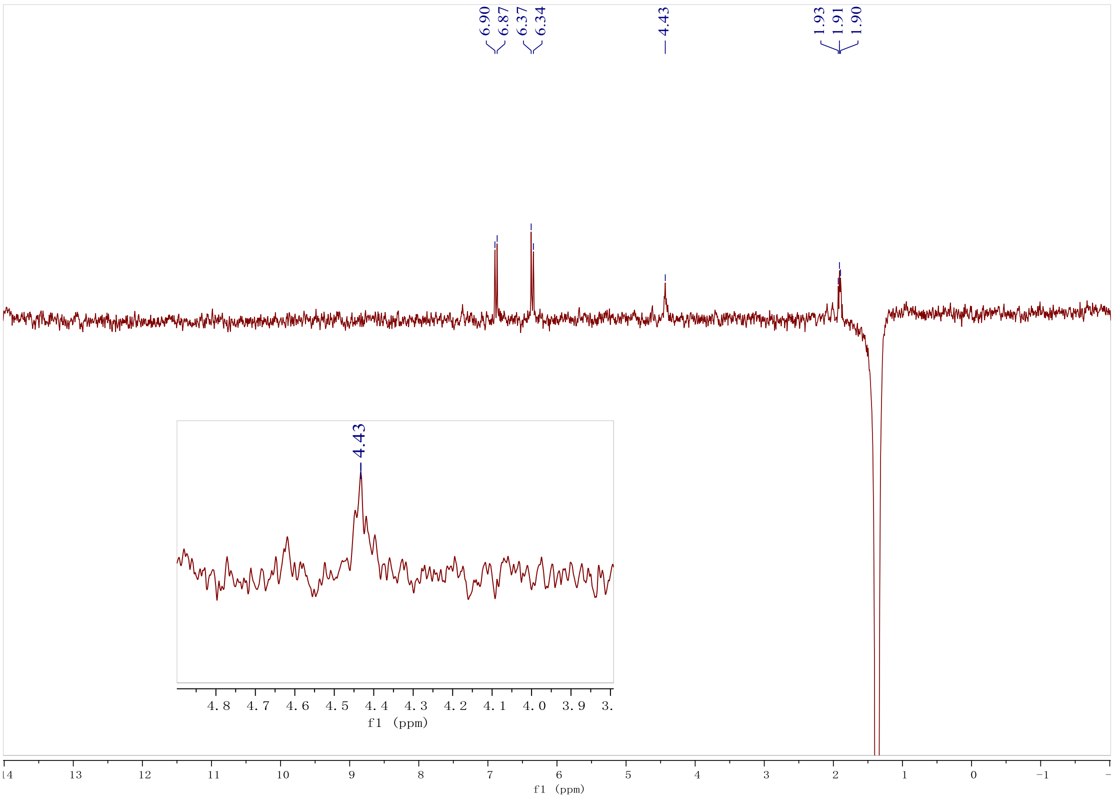

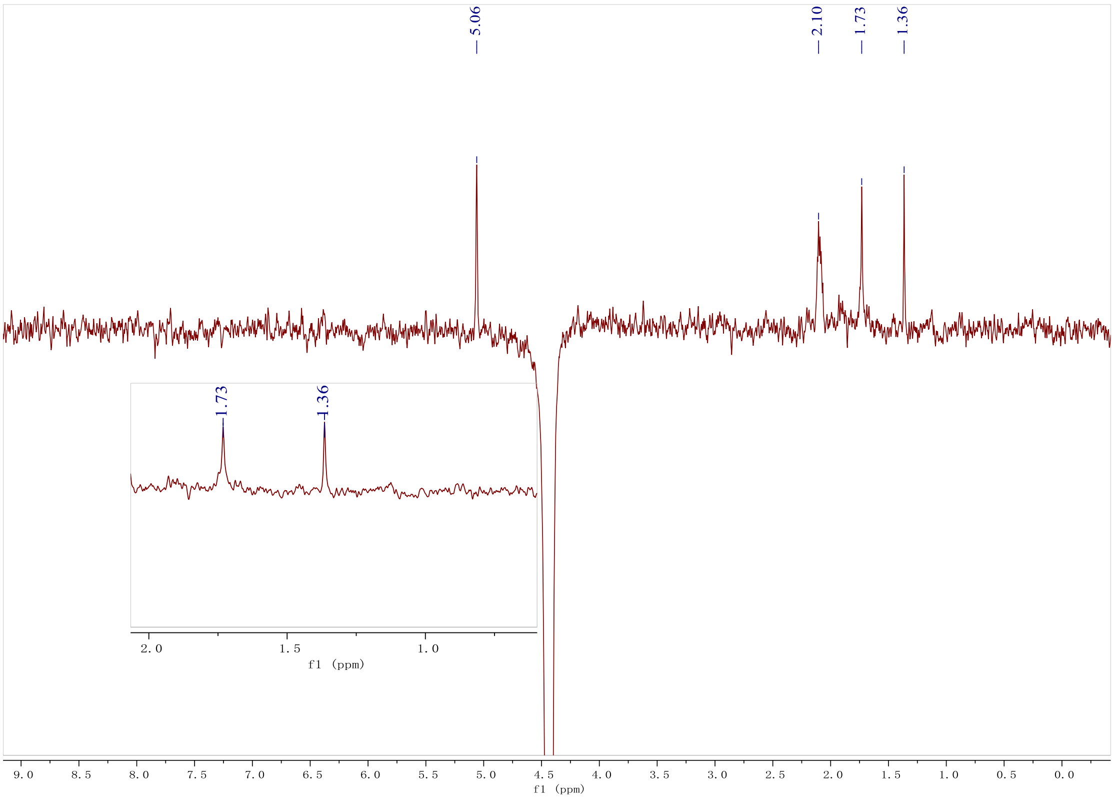

Supplementary Figure 66. NOE (500 MHz, acetone-d_6) spectra of compound **27**

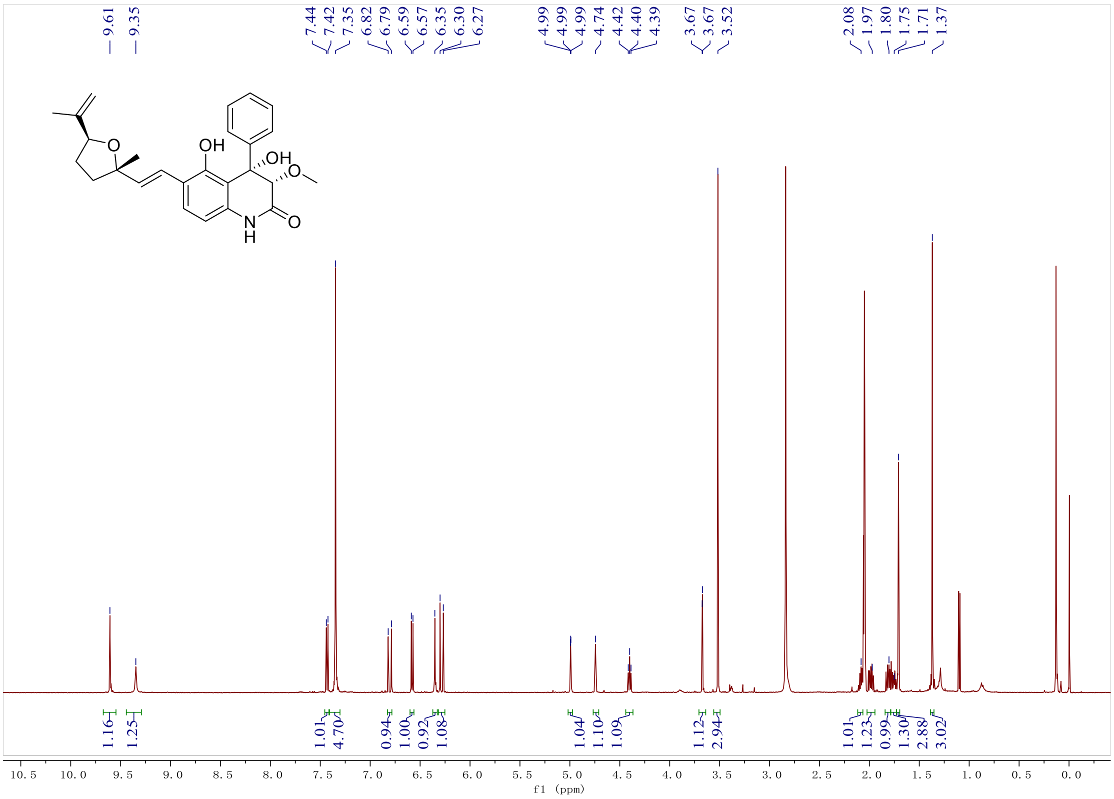

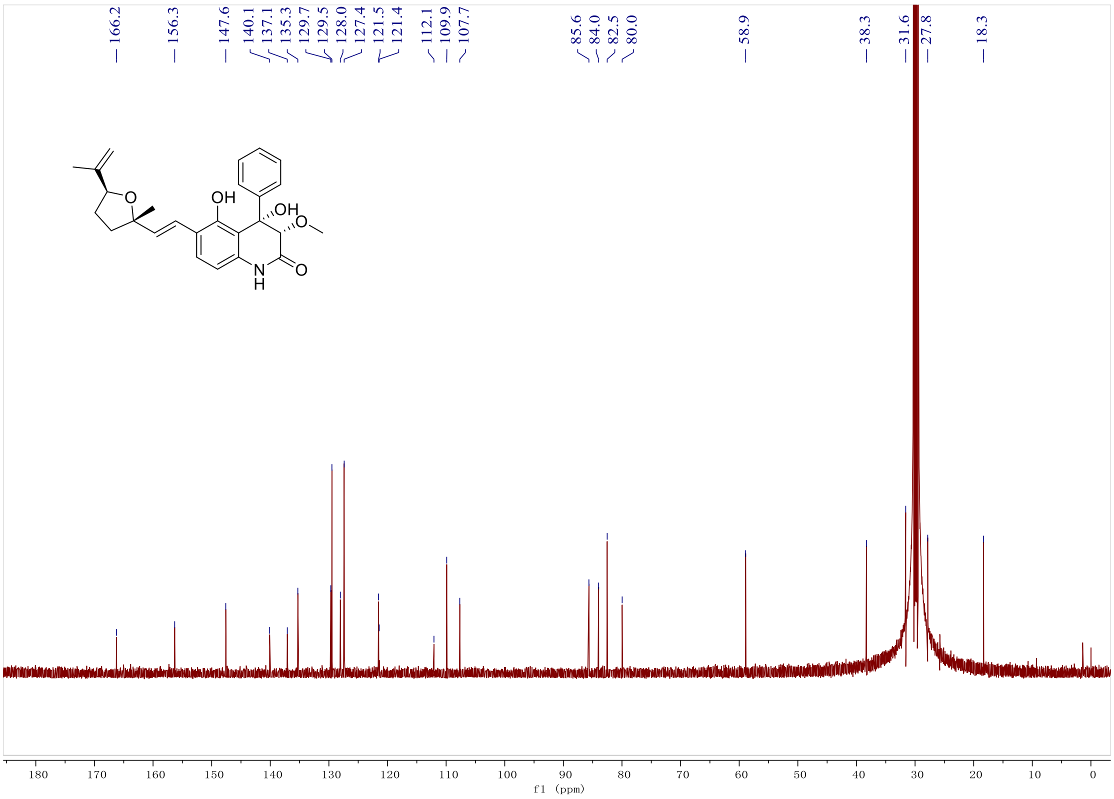

Supplementary Figure 67. ^{1}H NMR (500 MHz, acetone-d_6) and ^{13}C NMR (125 MHz, acetone-d_6) spectra of compound **26**

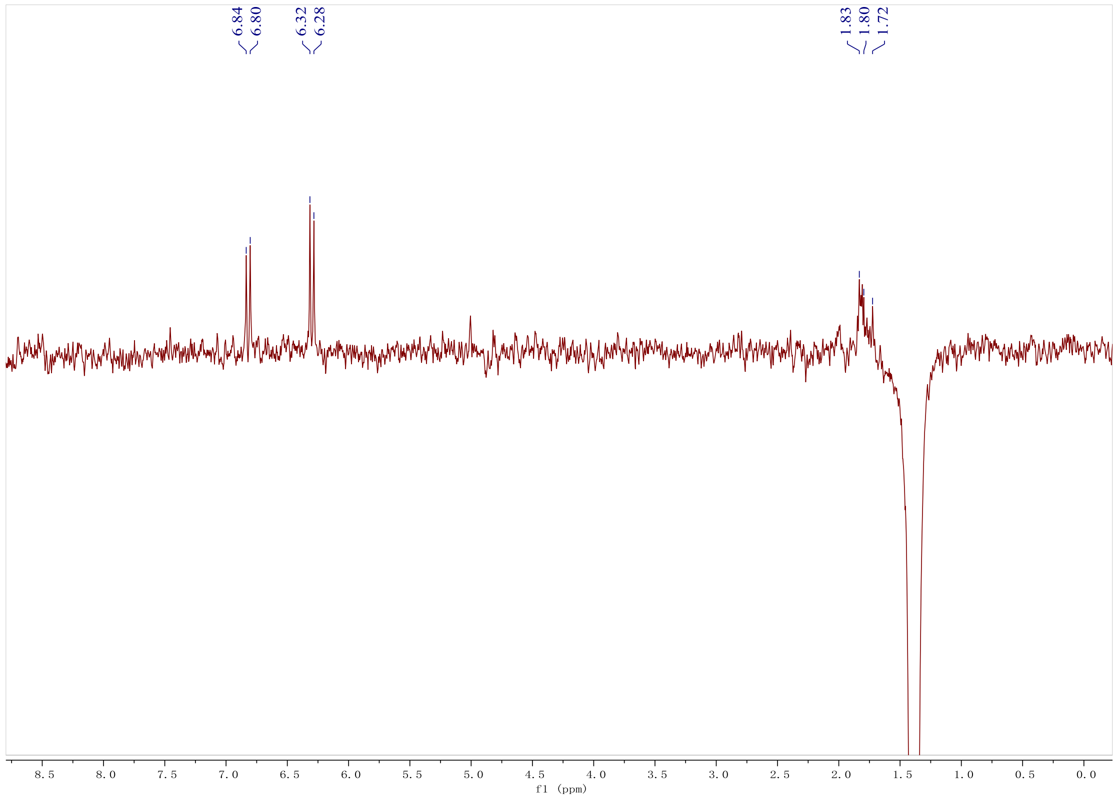

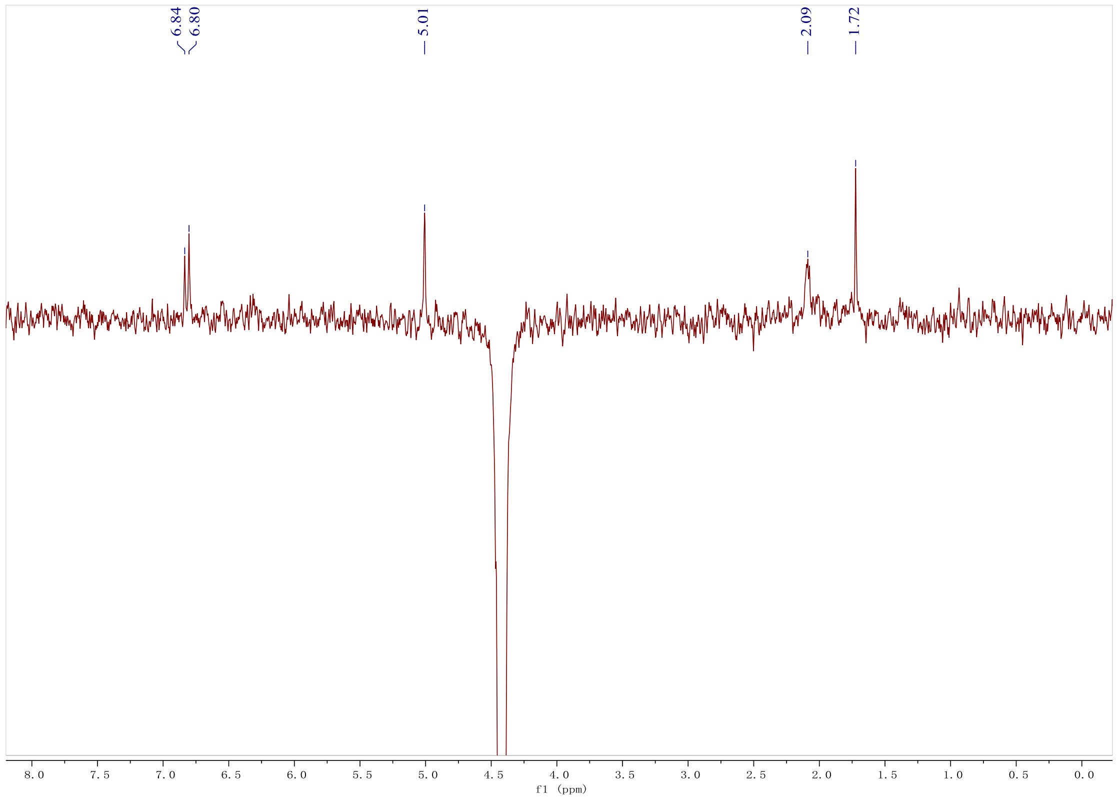

Supplementary Figure 68. NOE (500 MHz, acetone-d_6) spectra of compound **26**

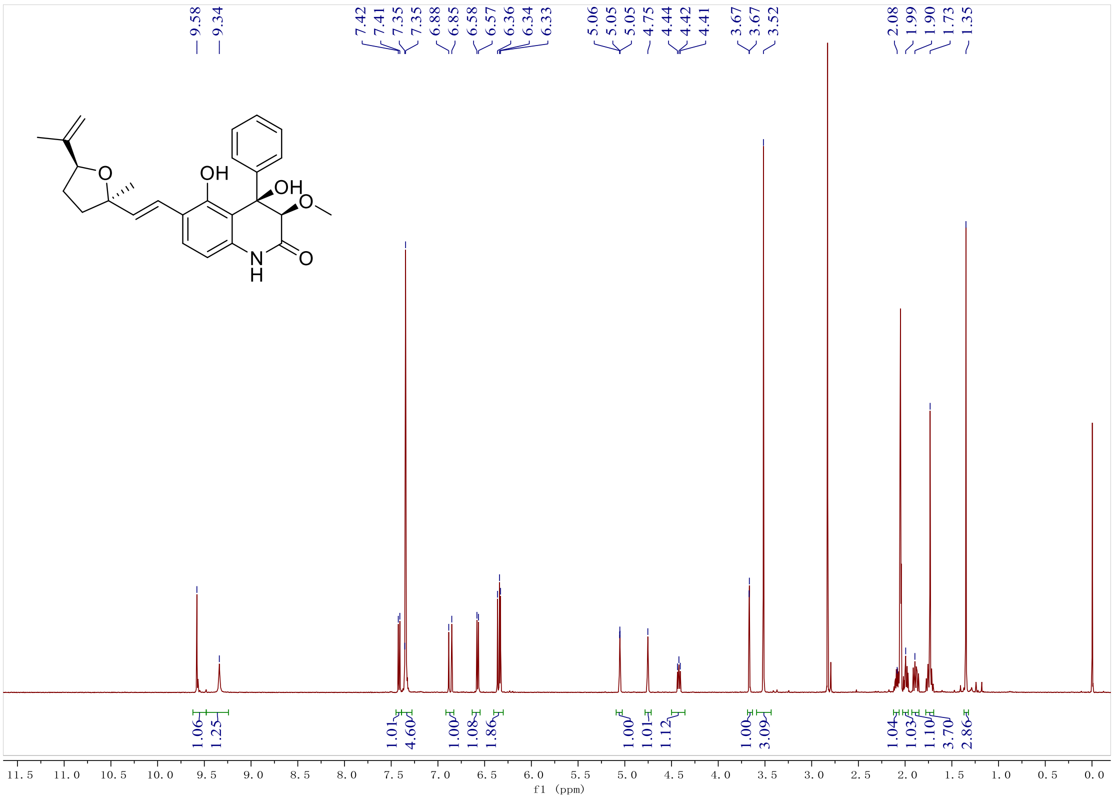

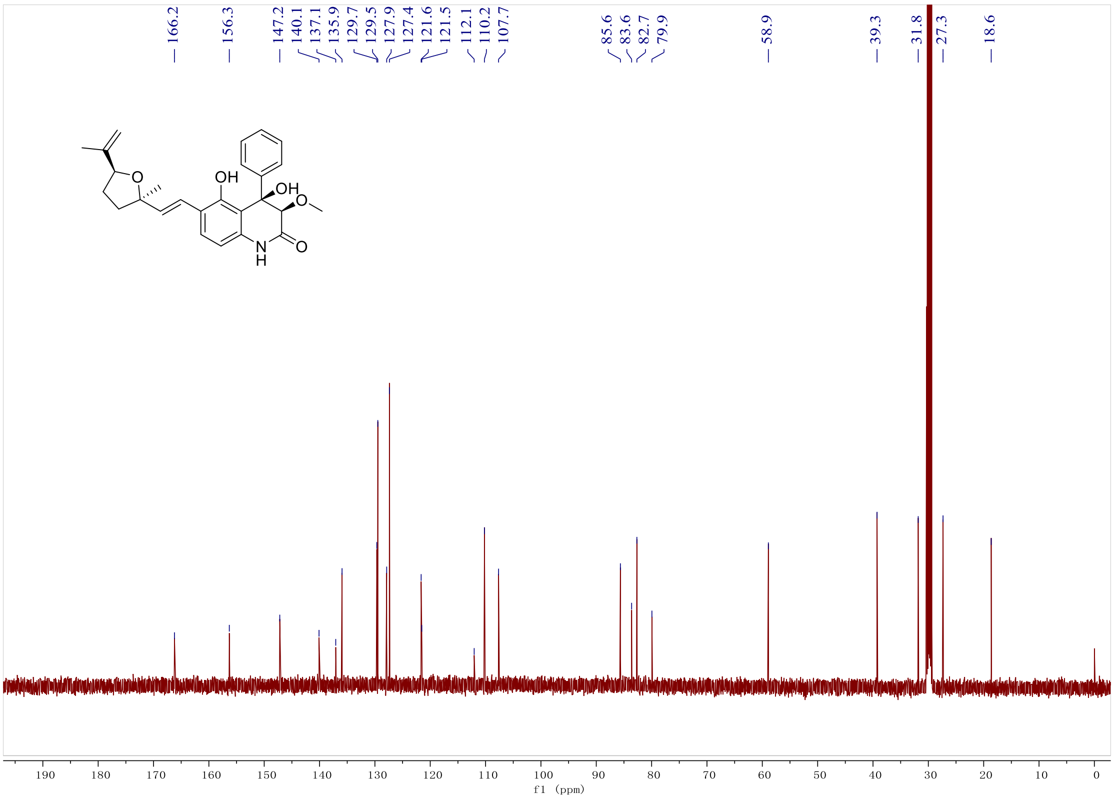

Supplementary Figure 69. ^{1}H NMR (500 MHz, acetone-d_6) and ^{13}C NMR (125 MHz, acetone-d_6) spectra of compound **31**

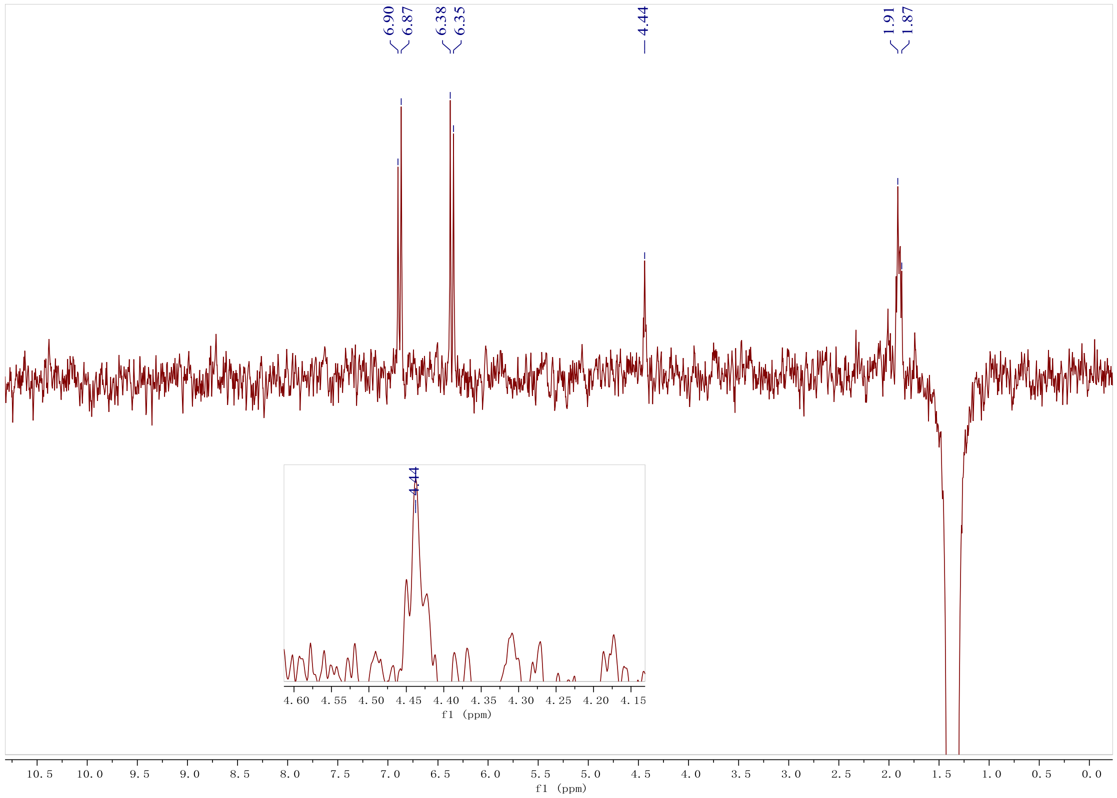

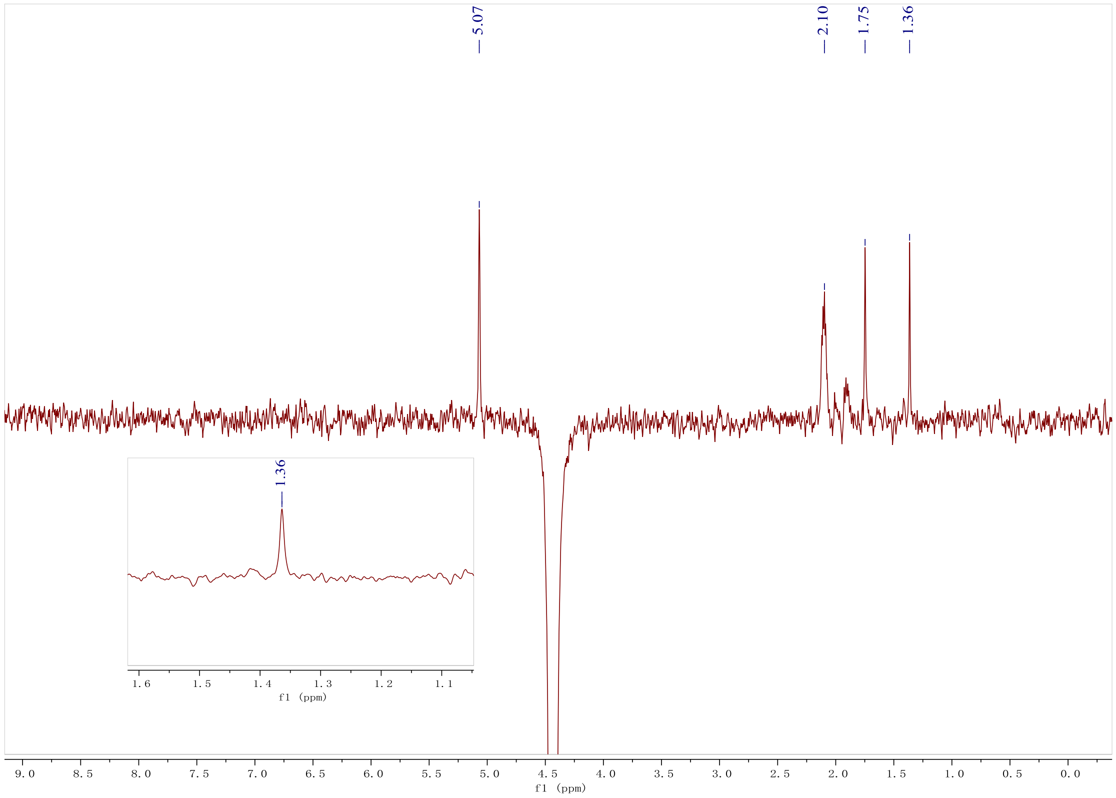

Supplementary Figure 70. NOE (500 MHz, acetone-d_6) spectra of compound **31**

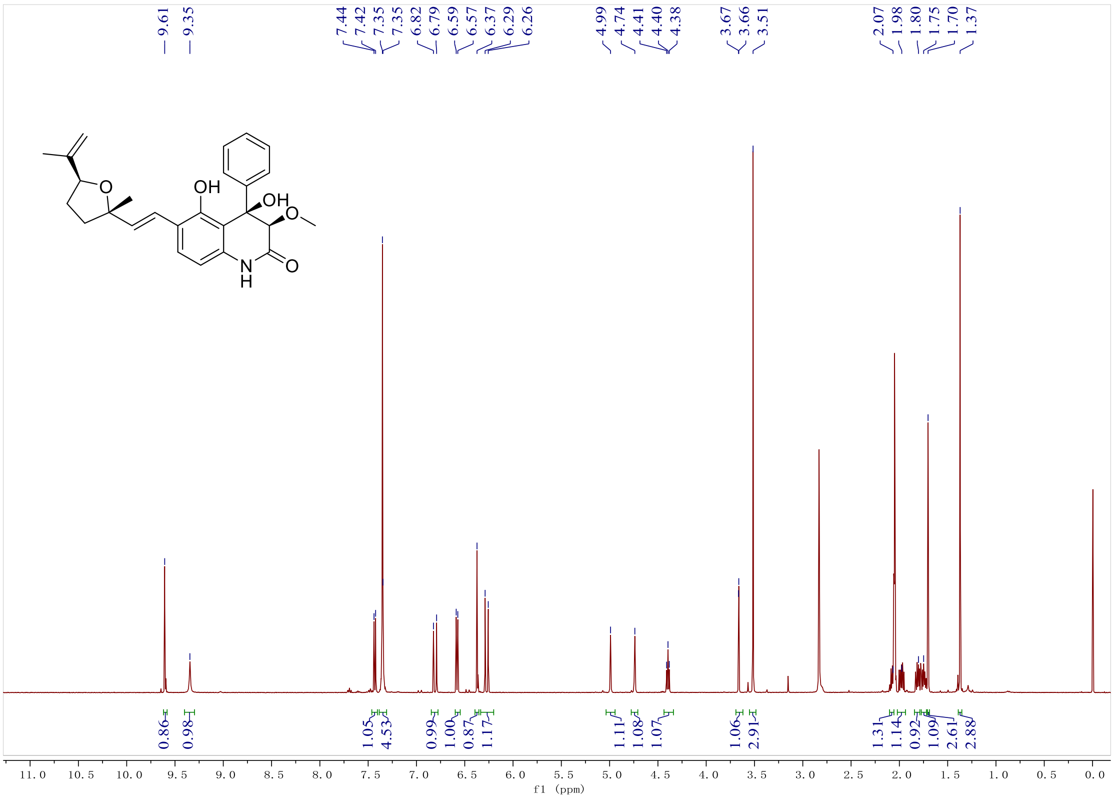

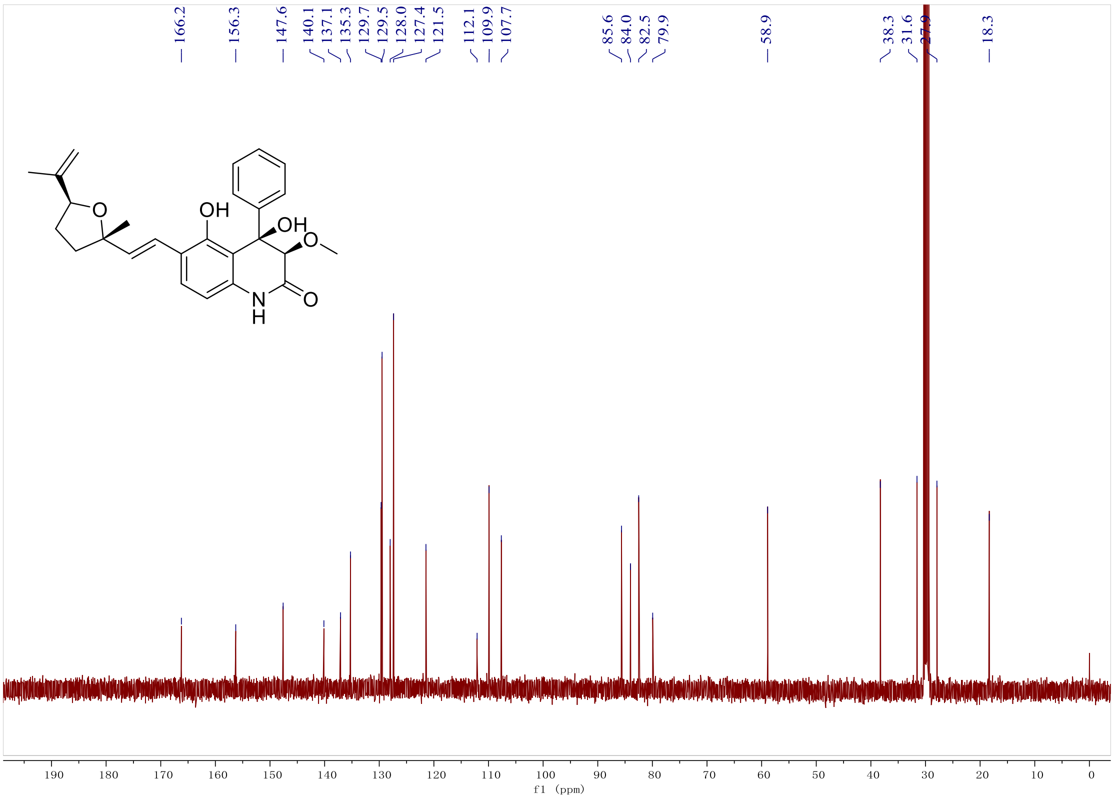

Supplementary Figure 71. ^{1}H NMR (500 MHz, acetone-d_6) and ^{13}C NMR (125 MHz, acetone-d_6) spectra of compound **30**

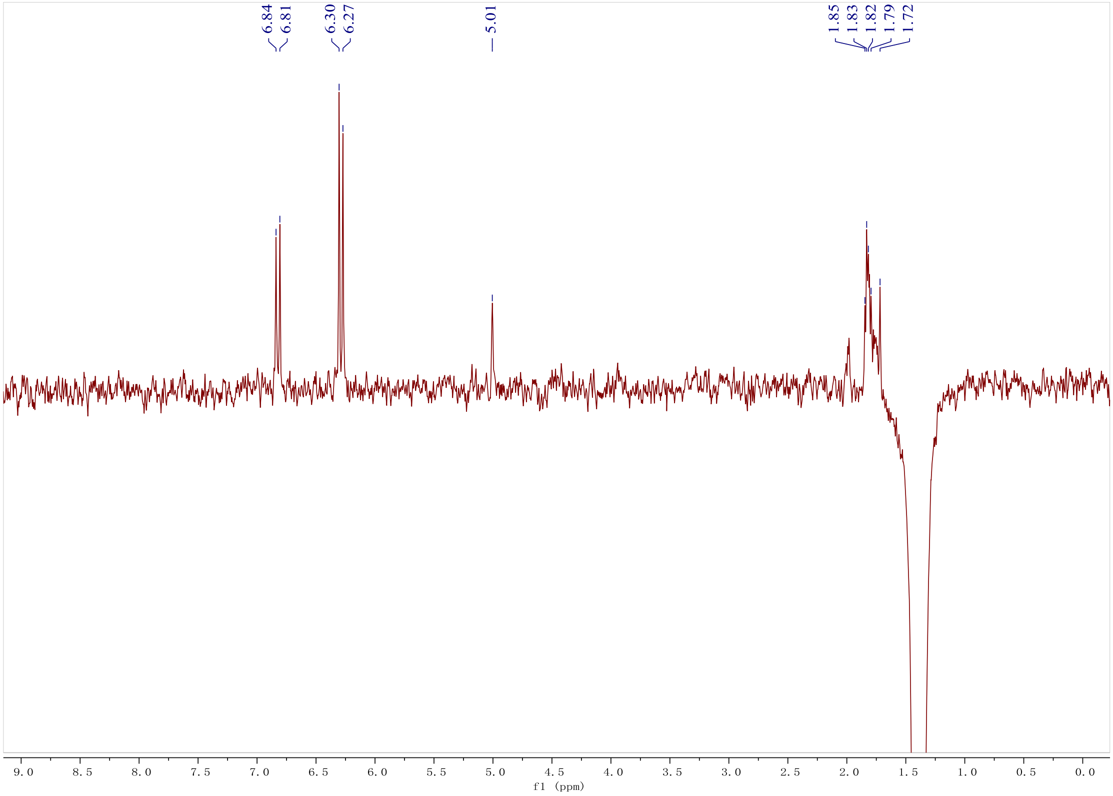

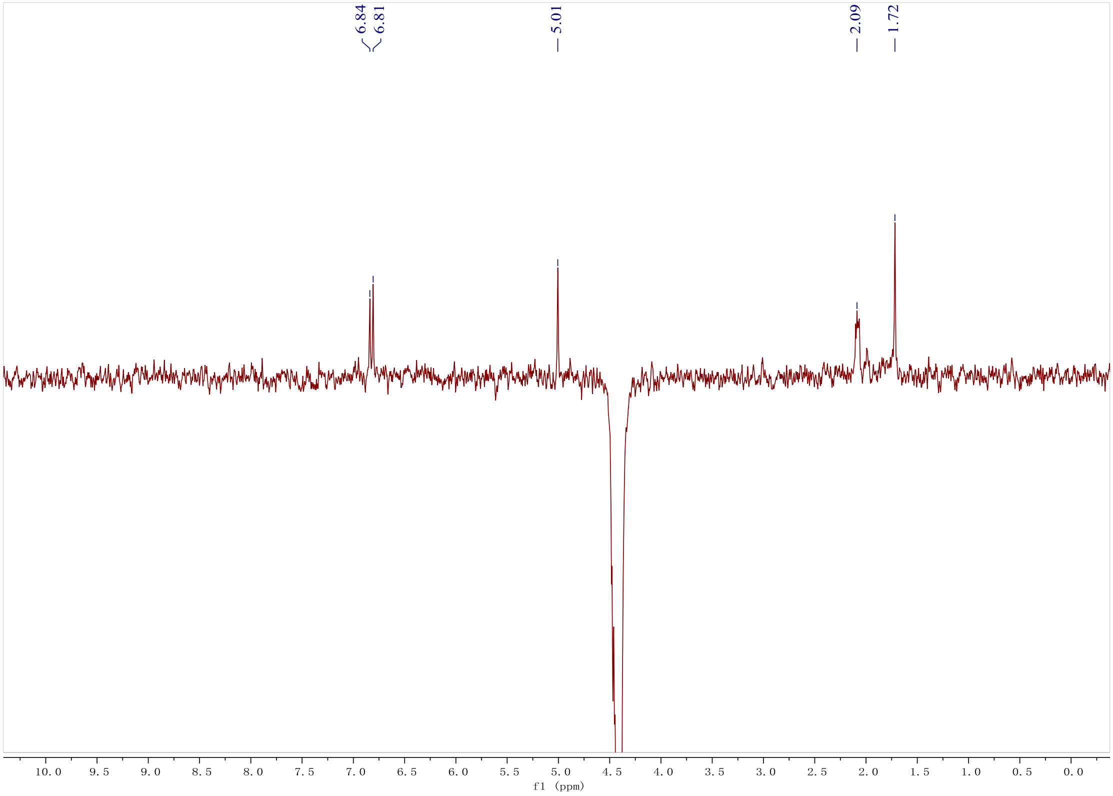

Supplementary Figure 72. NOE (500 MHz, acetone-d_6) spectra of compound **30**

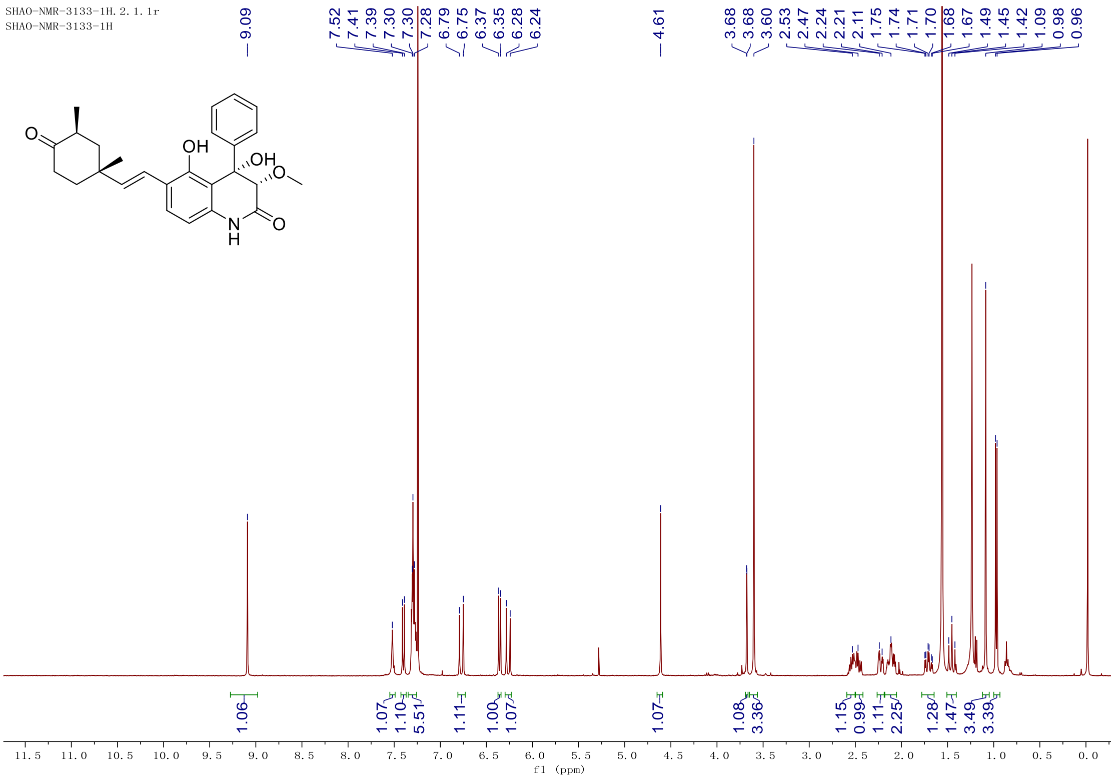

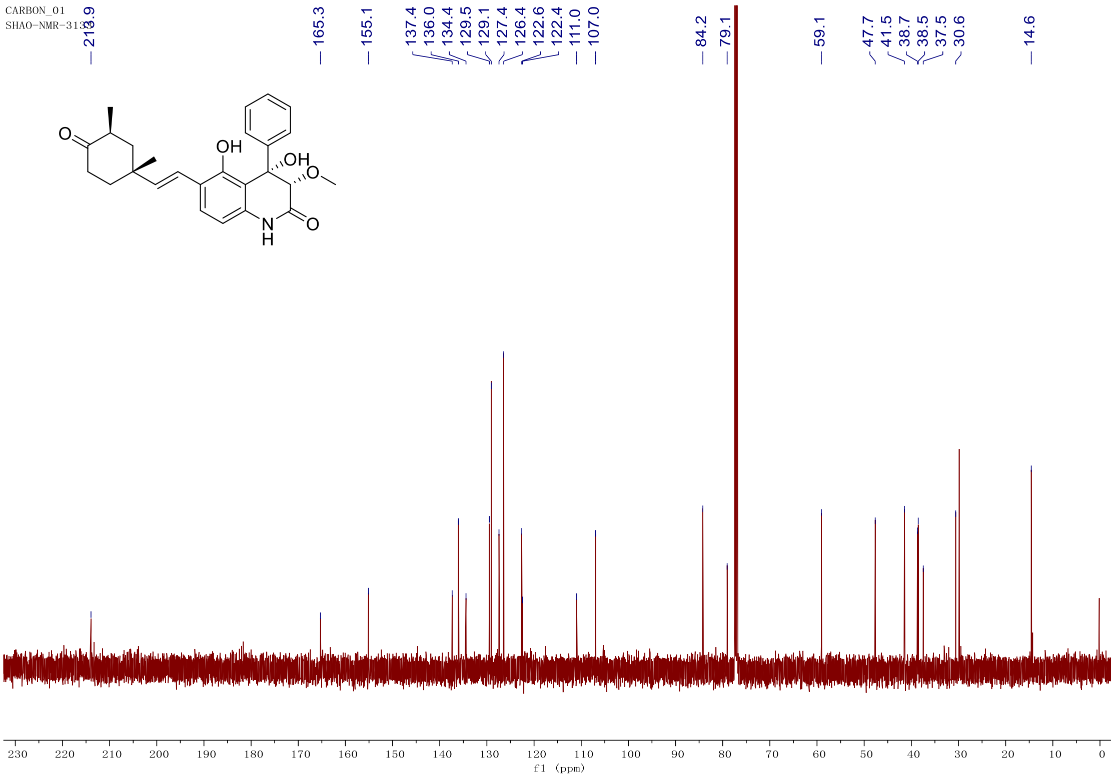

Supplementary Figure 73. ^{1}H NMR (400 MHz, $CDCl_3$) and ^{13}C NMR (100 MHz, $CDCl_3$) spectra of compound **4**

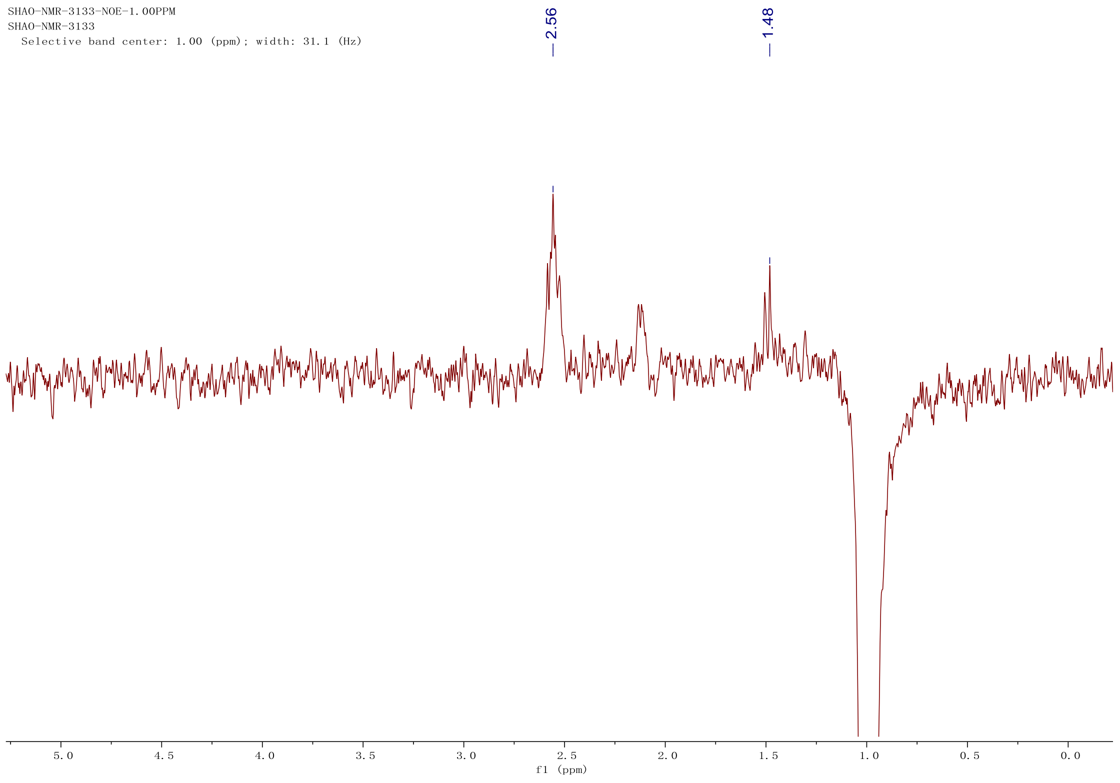

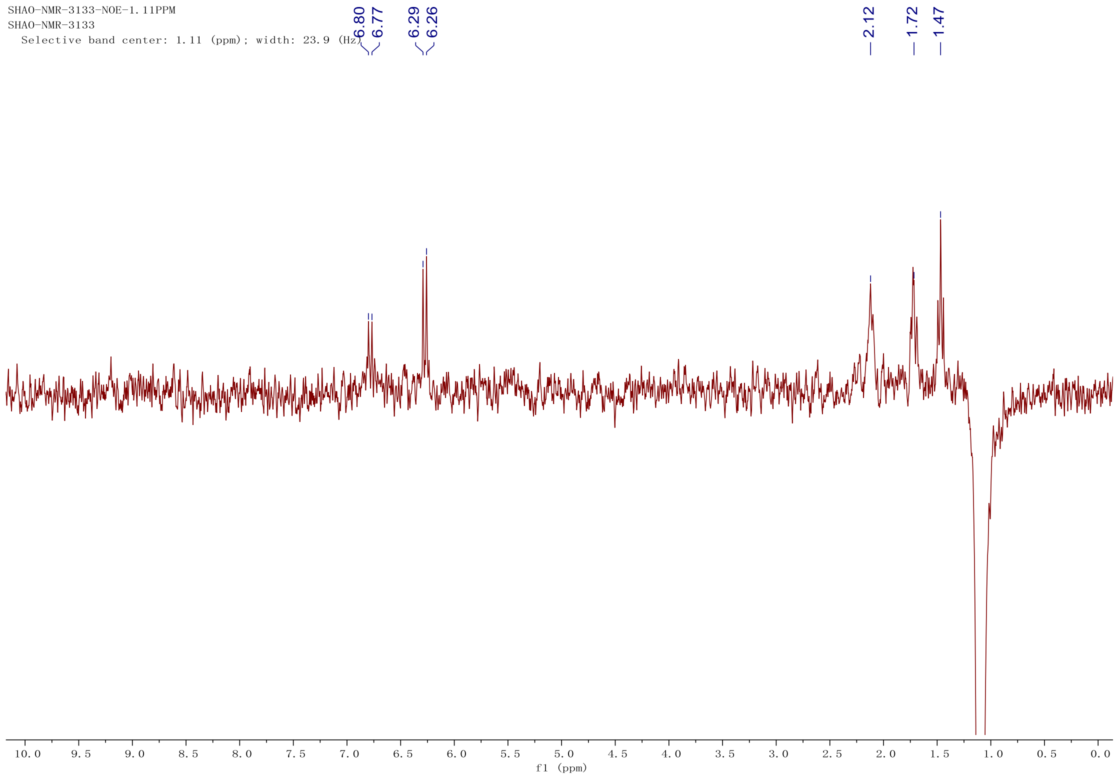

Supplementary Figure 74. NOE (500 MHz, $CDCl_3$) spectra of compound **4**

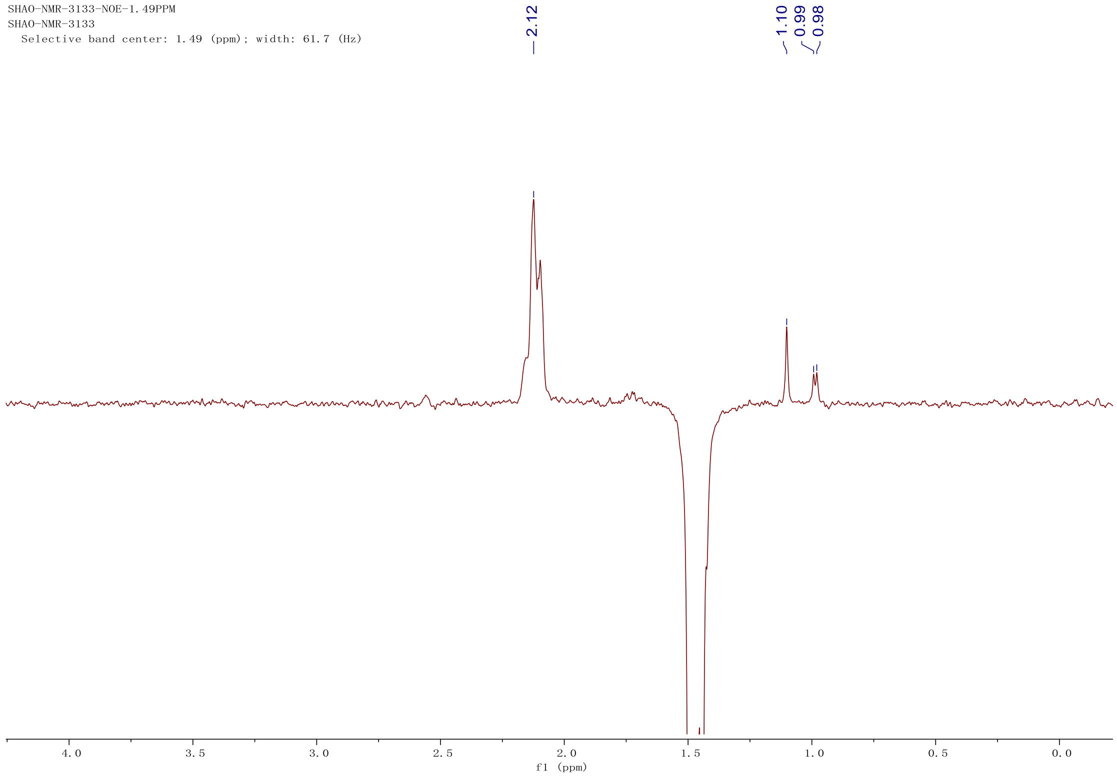

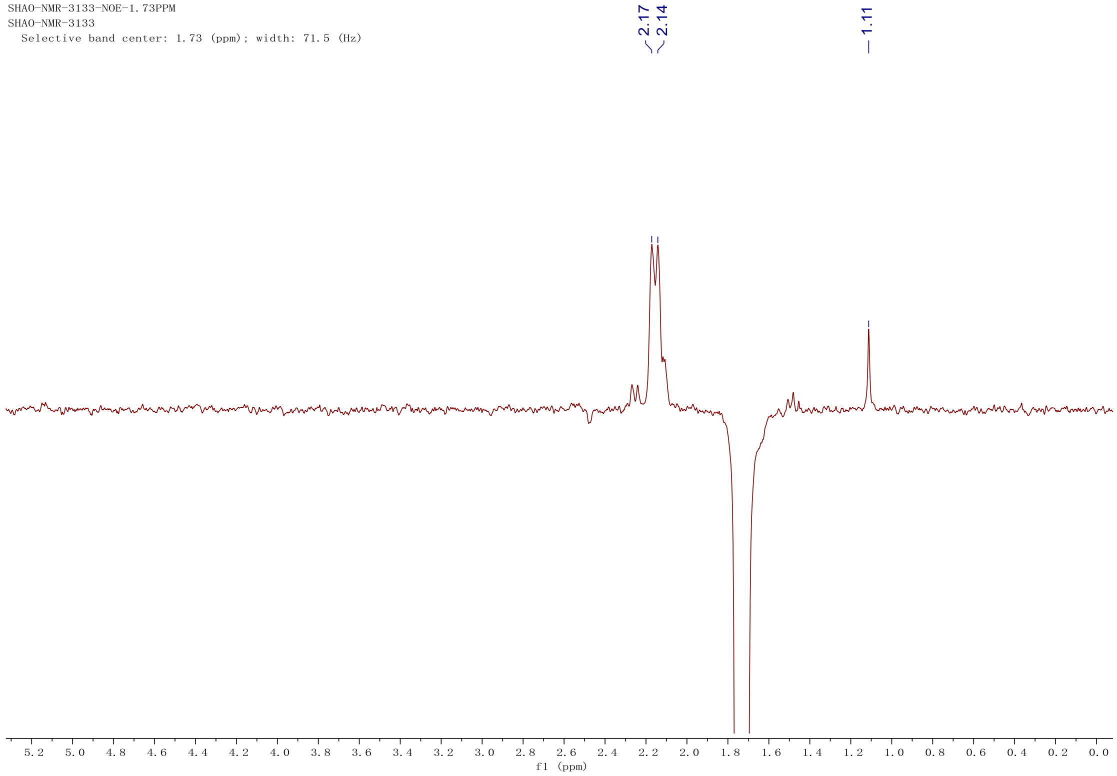

Supplementary Figure 75. NOE (500 MHz, $CDCl_3$) spectra of compound **4**

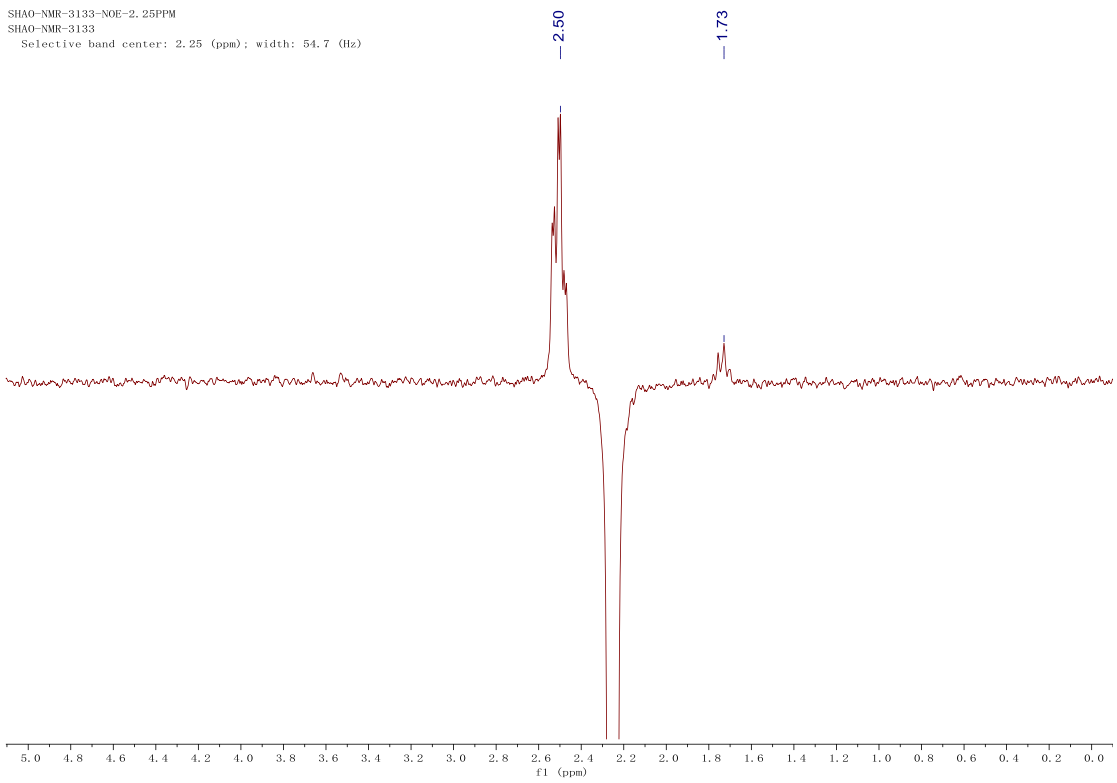

Supplementary Figure 76. NOE (500 MHz, $CDCl_3$) spectra of compound **4**

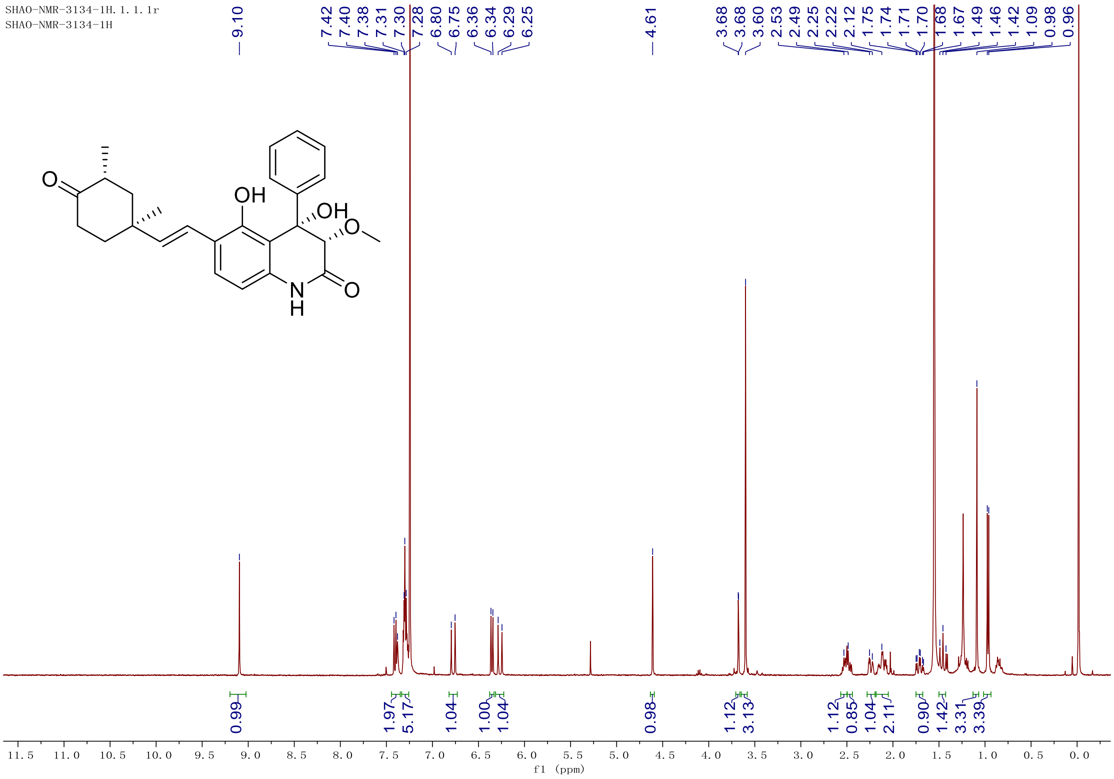

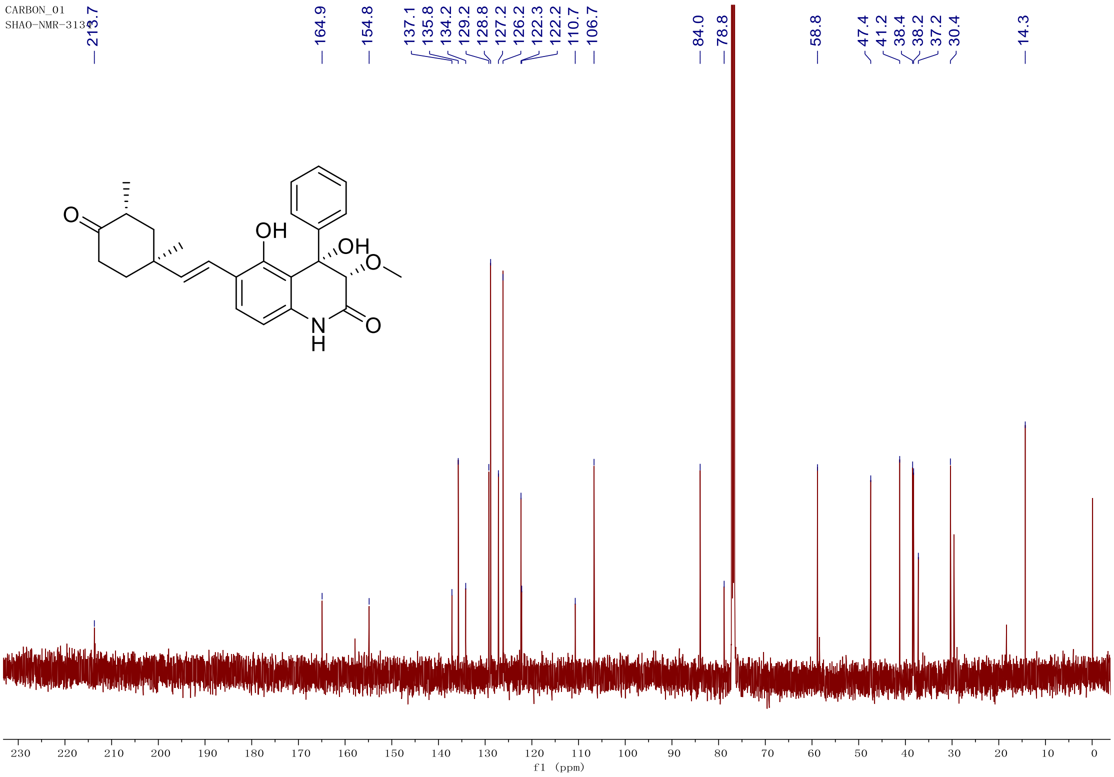

Supplementary Figure 77. ^{1}H NMR (400 MHz, $CDCl_3$) and ^{13}C NMR (100 MHz, $CDCl_3$) spectra of compound **5**

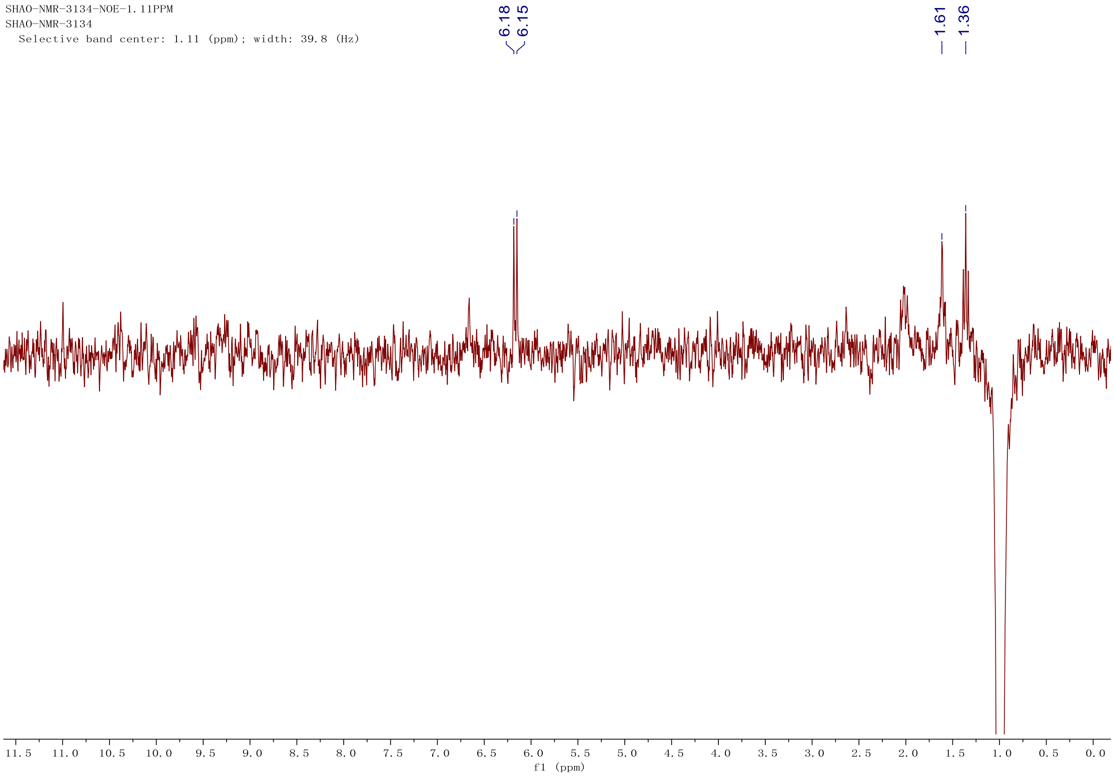

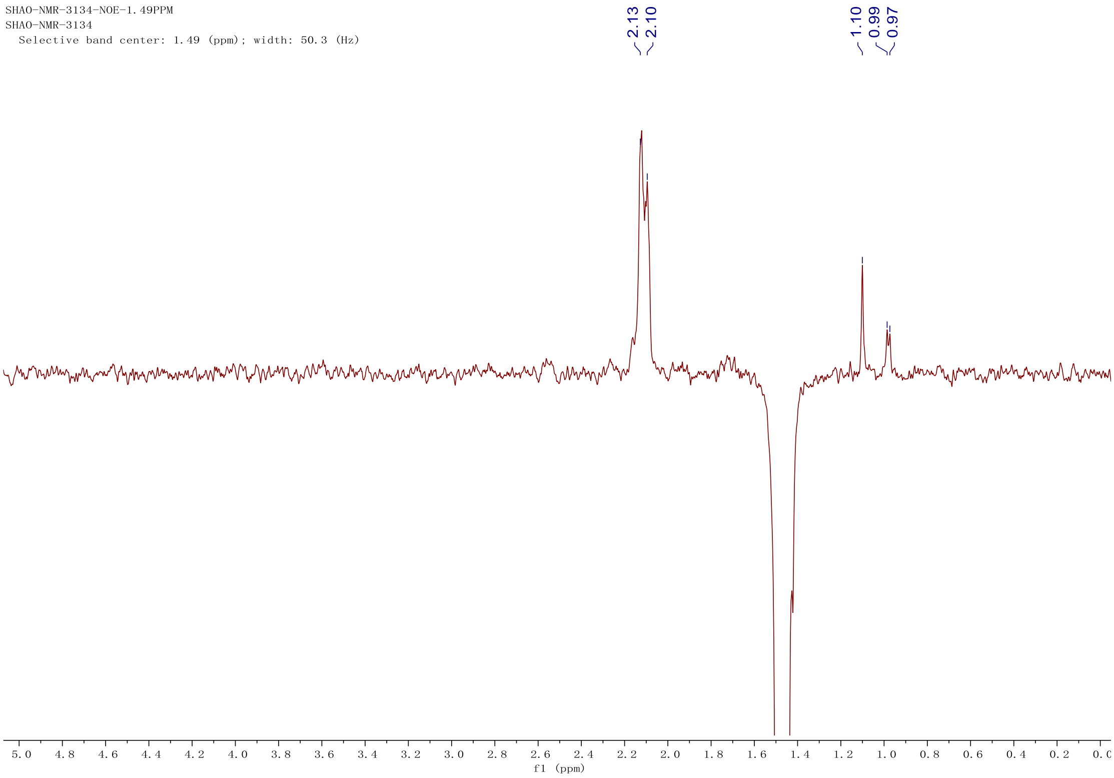

Supplementary Figure 78. NOE (500 MHz, $CDCl_3$) spectra of compound **5**

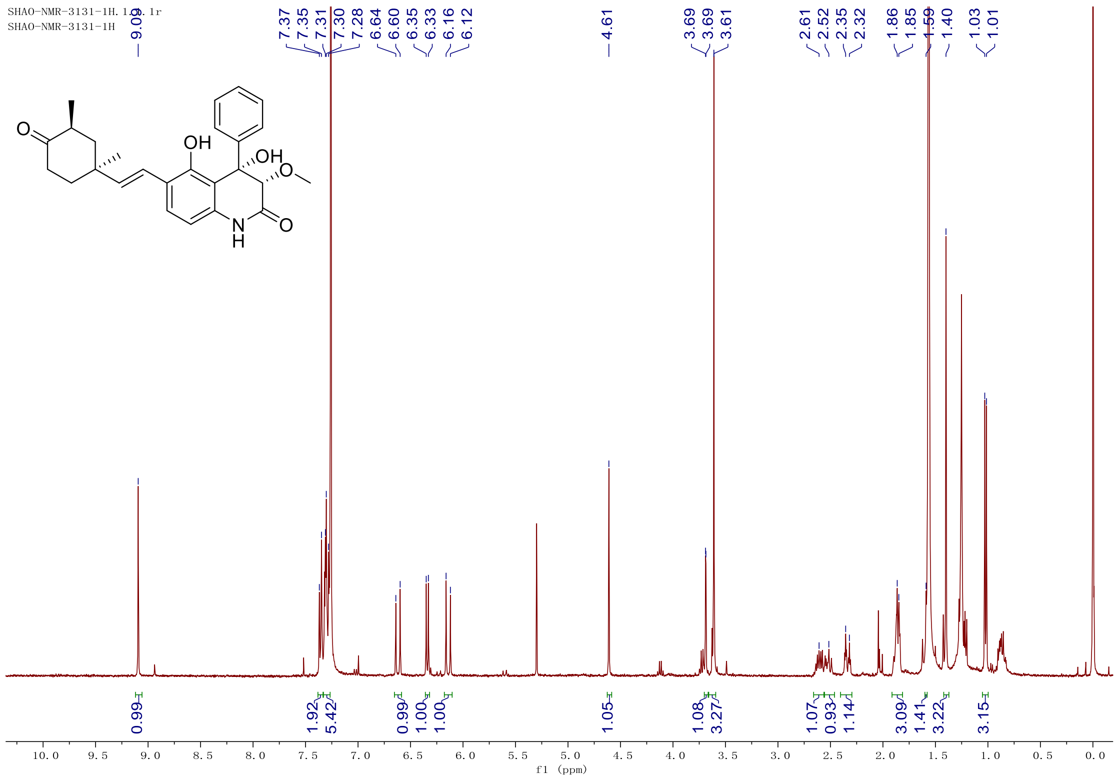

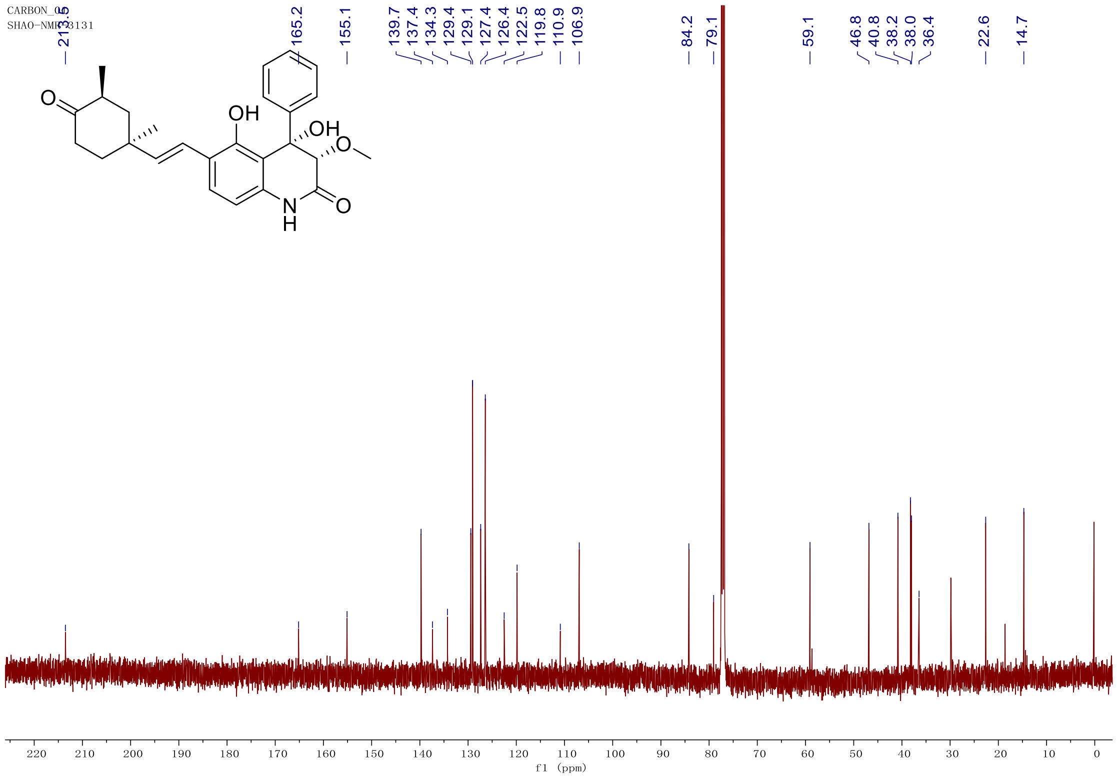

Supplementary Figure 79. ^{1}H NMR (400 MHz, $CDCl_3$) and ^{13}C NMR (100 MHz, $CDCl_3$) spectra of compound **6**

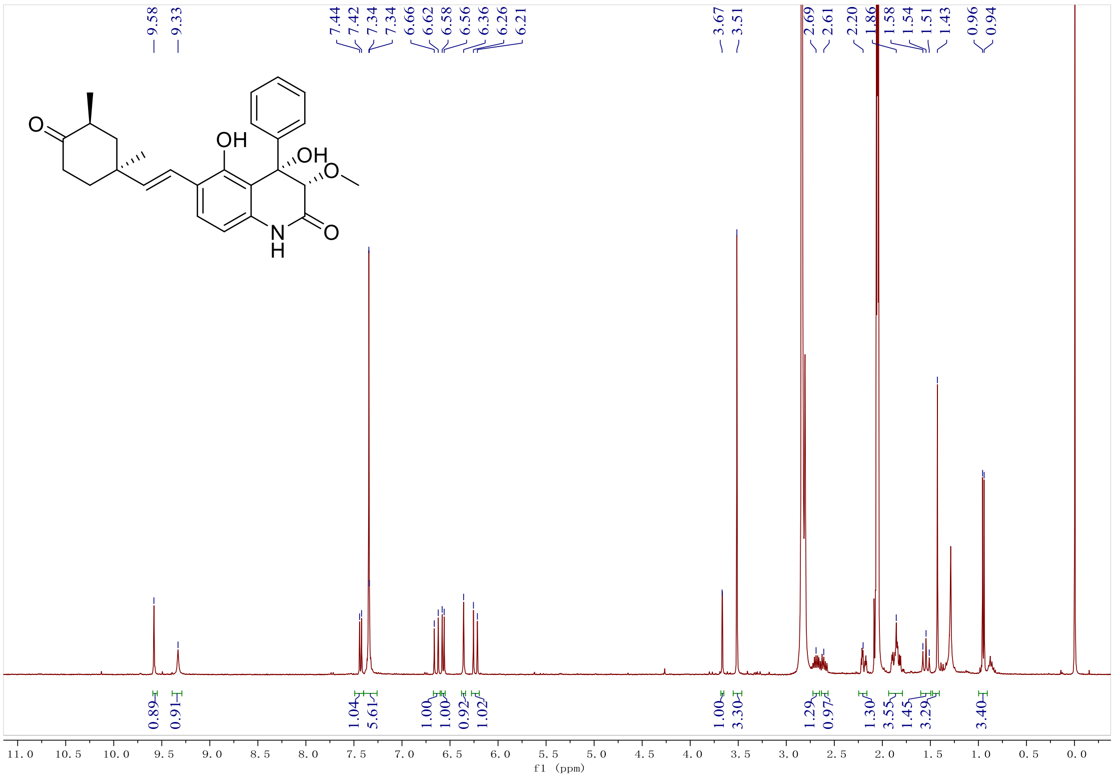

Supplementary Figure 80. ^{1}H NMR (400 MHz, acetone-d_6) spectra of compound **6**

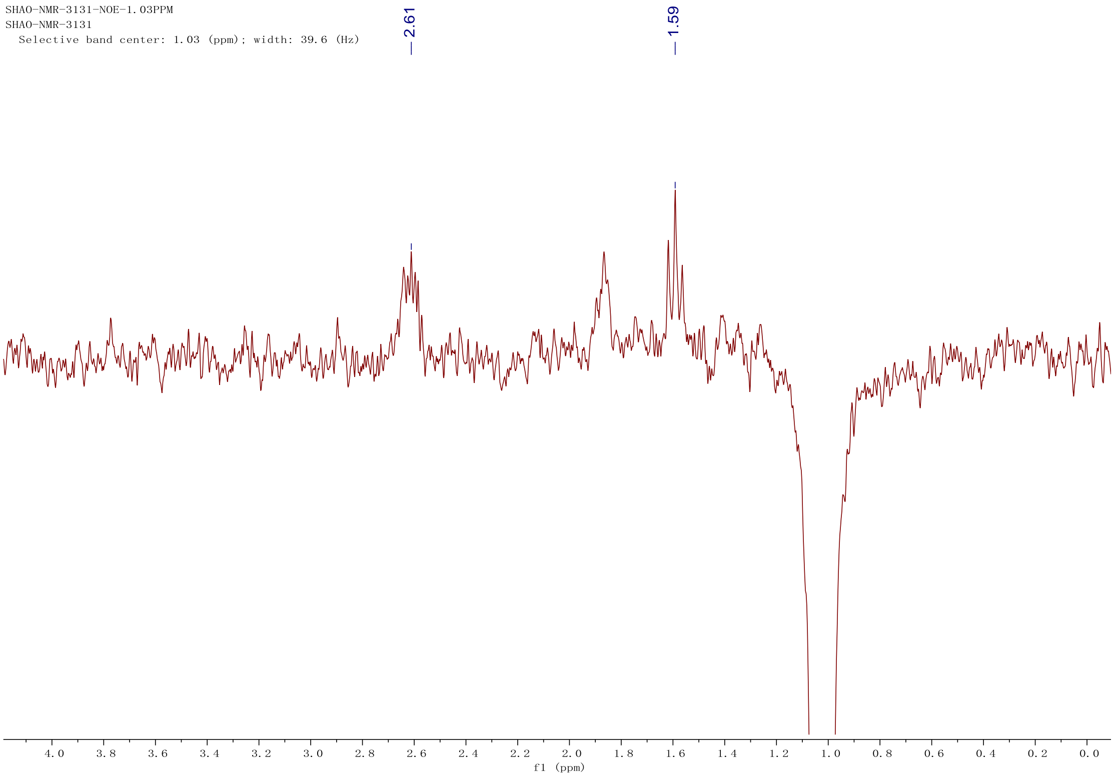

Supplementary Figure 81. NOE (500 MHz, $CDCl_3$) spectra of compound **6**

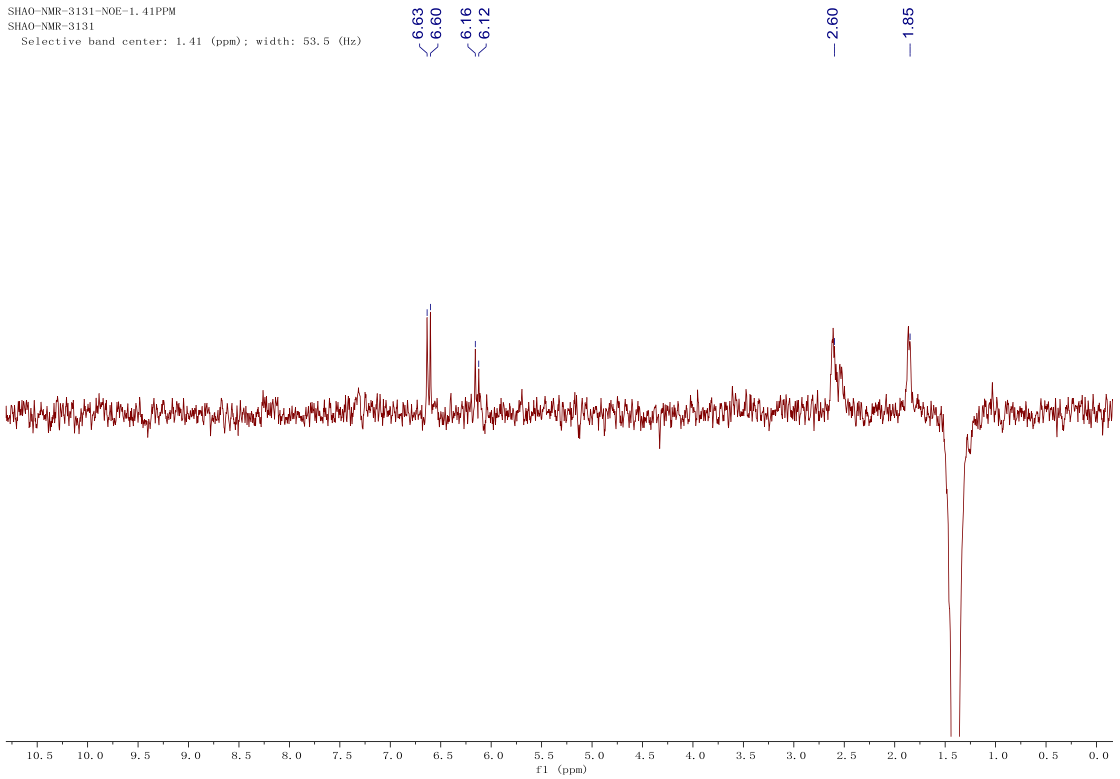

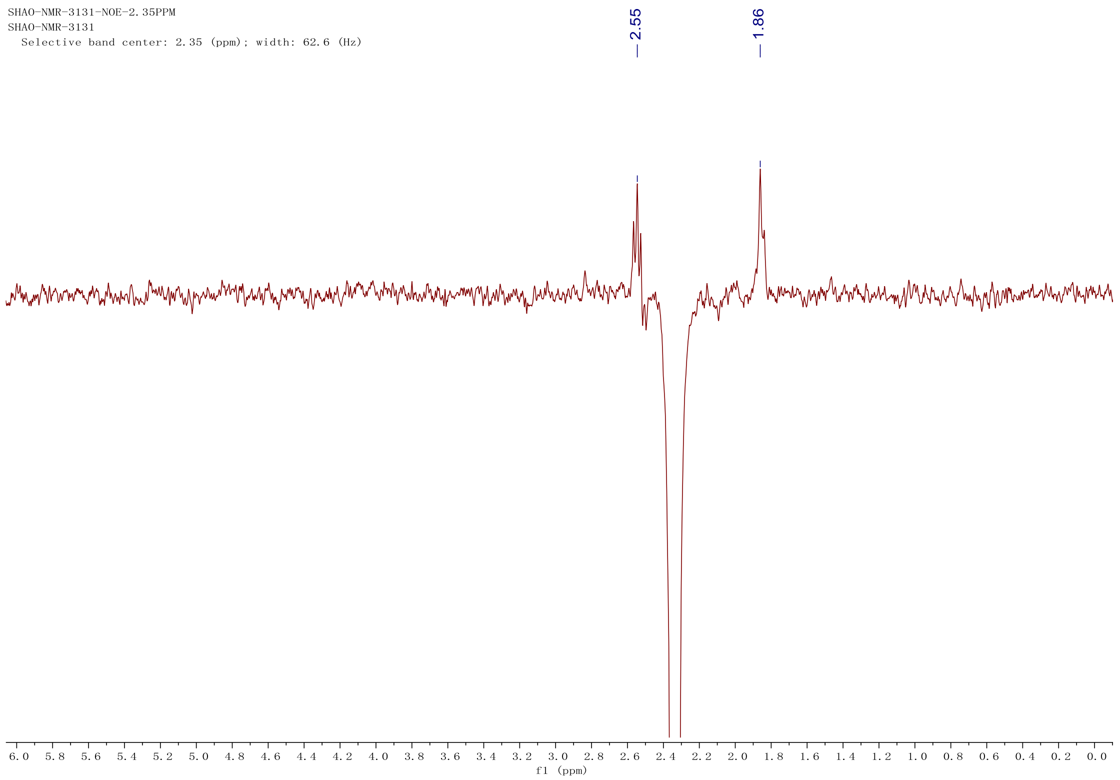

Supplementary Figure 82. NOE (500 MHz, $CDCl_3$) spectra of compound **6**

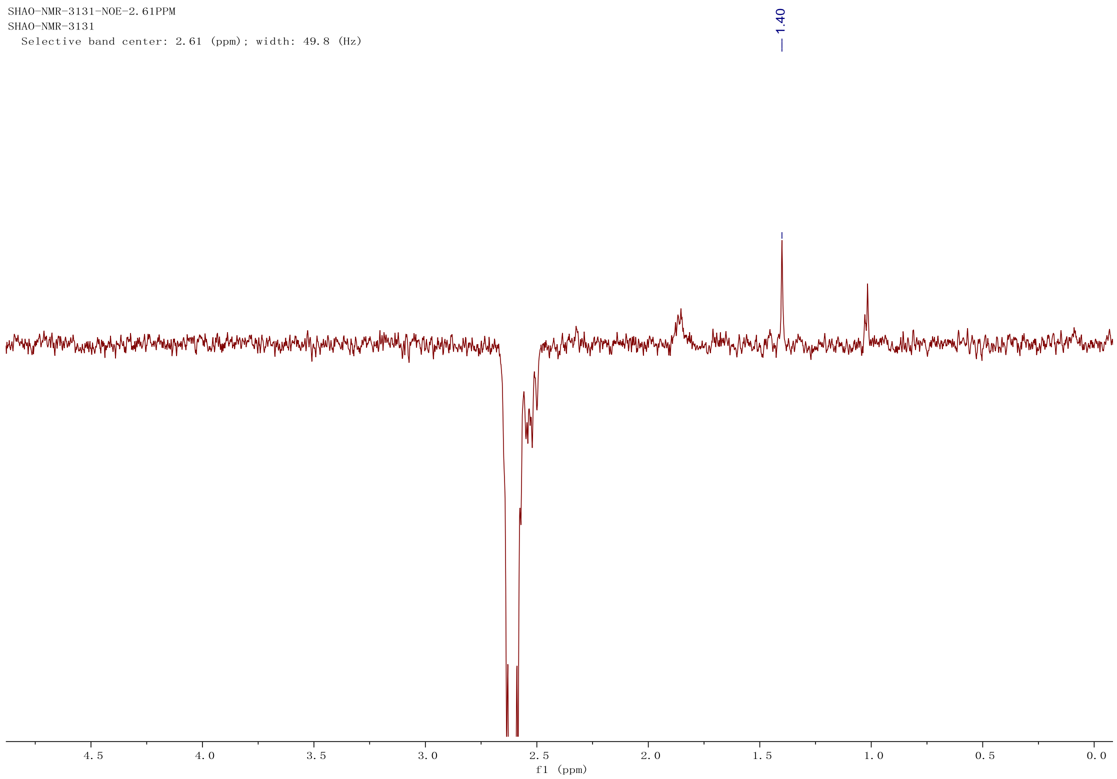

Supplementary Figure 83. NOE (500 MHz, $CDCl_3$) spectra of compound **6**

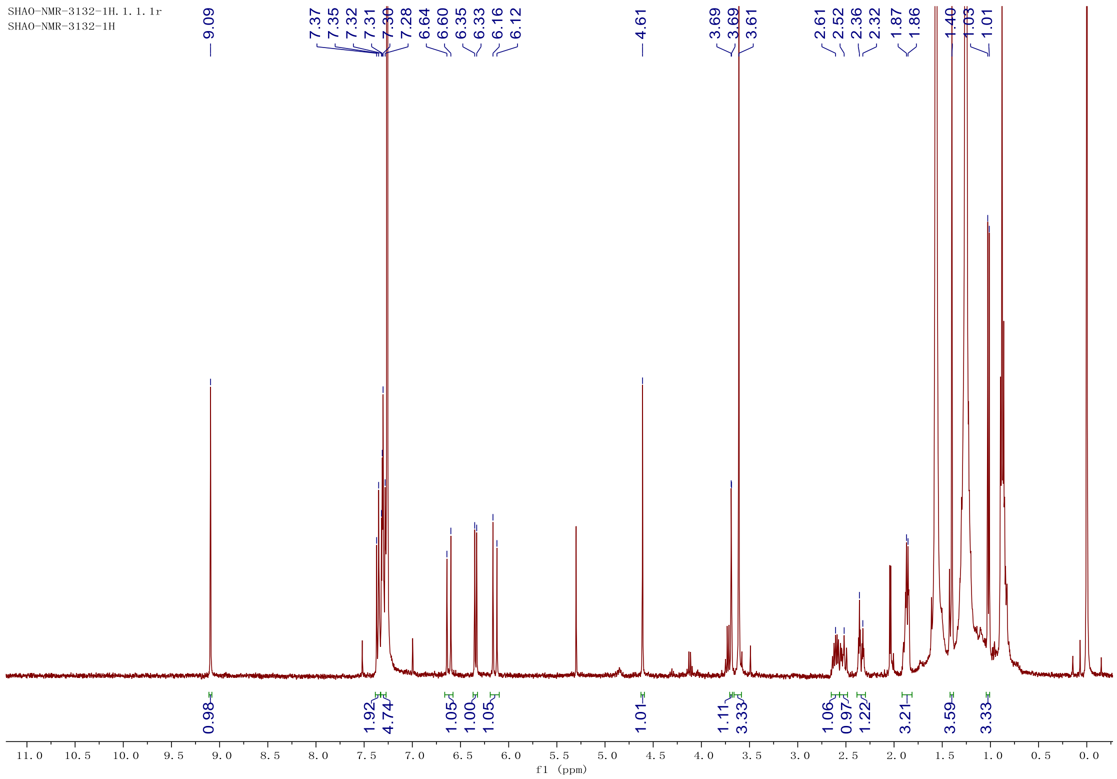

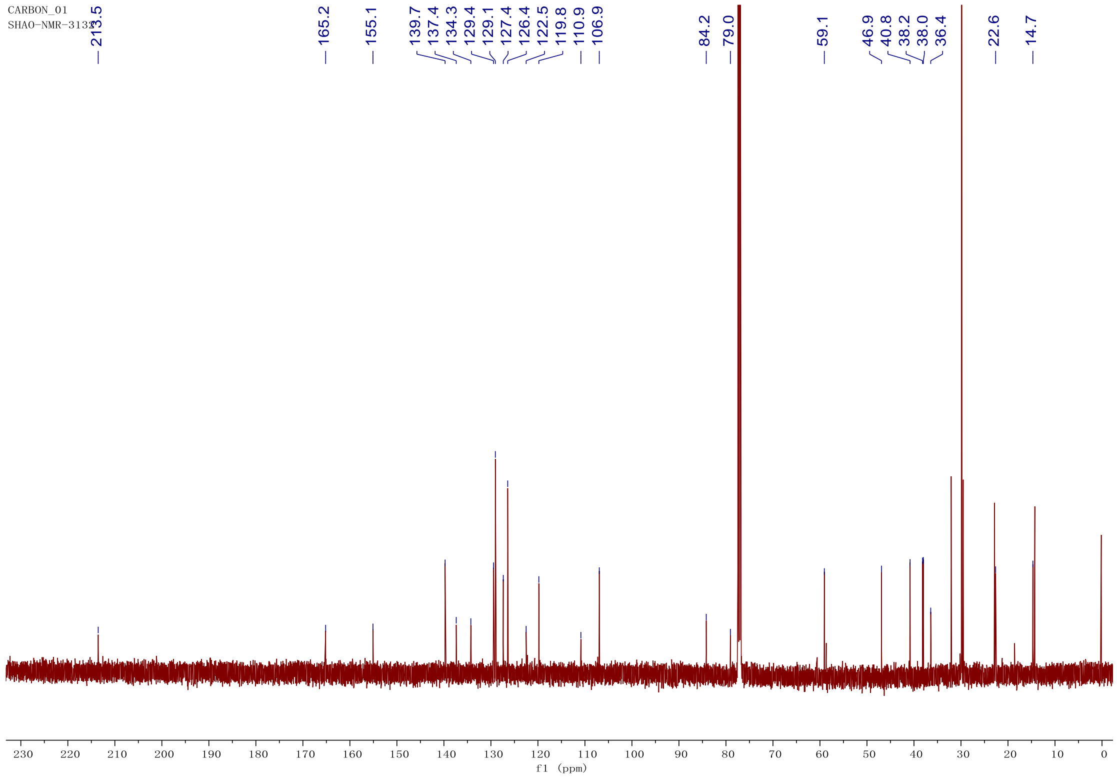

Supplementary Figure 84. ^{1}H NMR (500 MHz, $CDCl_3$) and ^{13}C NMR (125 MHz, $CDCl_3$) spectra of compound **32**

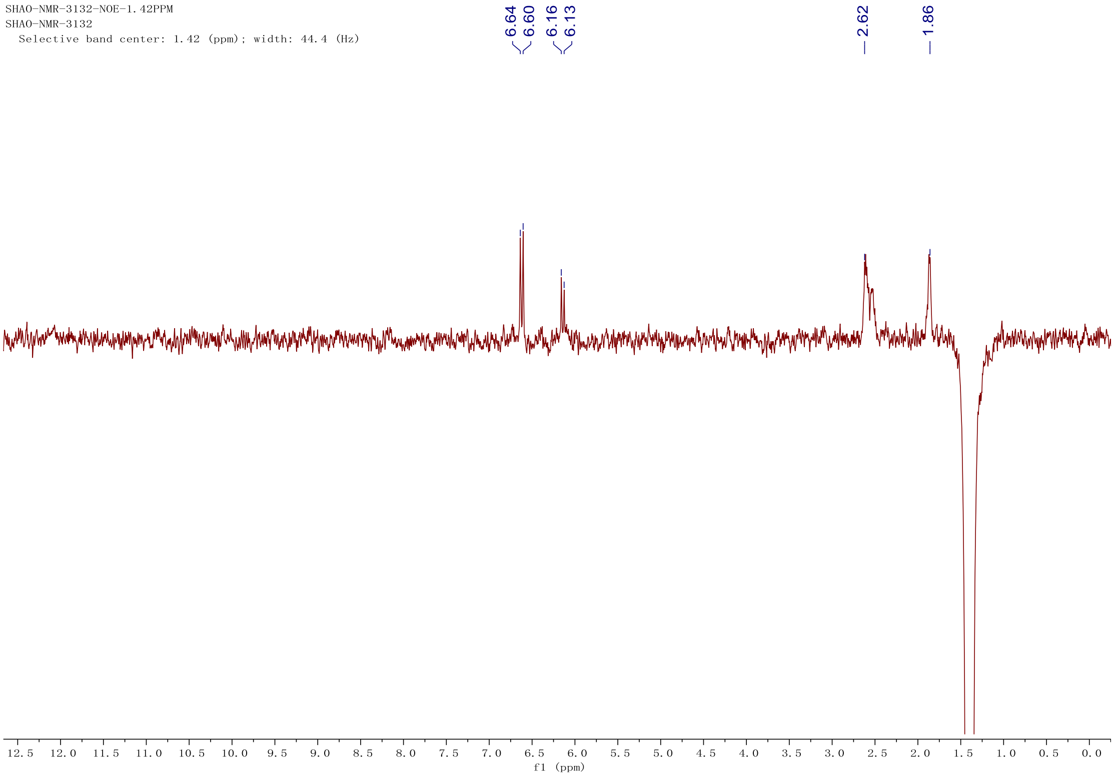

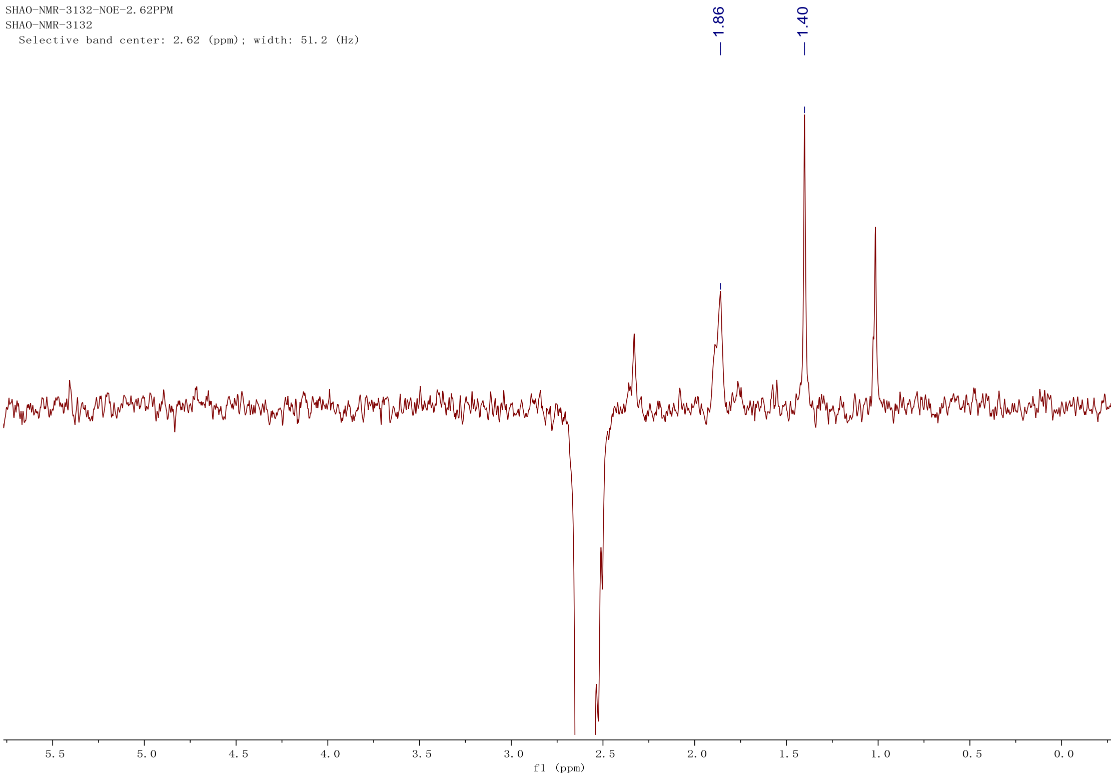

Supplementary Figure 85. NOE (500 MHz, $CDCl_3$) spectra of compound **32**

Supplementary References

1. Chan, C. K., Tsai, Y. L., Chan, Y. L. & Chang, M. Y. Synthesis of Substituted 2,3-Benzodiazepines. *J. Org. Chem.* **81**, 9836-9847 (2016).
2. Kumar, D., Vaya, D. & Chundawat, T. S. Total Synthesis of 6-Hydroxymetatacarboline-D Discovered from *Mycena metata* via the Pictet-Spengler Reaction Followed by the Horner-Wadsworth-Emmons Reaction. *ACS Omega* **6**, 8933-8941 (2021).
